# Solvent-Controlled Reaction Productivity and Mechanistic Pathways in the Multicomponent Synthesis of 2‑Amino-3-cyano‑4*H*‑pyrans

**DOI:** 10.1021/acsorginorgau.6c00059

**Published:** 2026-07-15

**Authors:** Jhonathan Renner Nunes dos Santos, Luciana Machado Ramos, Juliana Gonzaga de Morais Lima, Saulo T. A. Passos, Aline Lima de Oliveira, Fabricio Machado, Brenno A. D. Neto

**Affiliations:** † Laboratory of Medicinal Chemistry and Organic Synthesis, Campus de Ciências Exatas e Tecnológicas, Universidade Estadual de Goiás, Molecular Sciences Graduate Program, Anápolis, Goiás 75132-400, Brazil; ‡ University of Brasília, Chemistry Institute (IQ-UnB), Laboratory of Medicinal and Technological Chemistry, Campus Universitário Darcy Ribeiro, Brasília, Distrito Federal 70910-900, Brazil

**Keywords:** synthetic enzyme, catalysis, multicomponent reaction, mechanism, solvent effect, biological

## Abstract

In this work, we present a detailed mechanistic and solvent-effect study of the multicomponent synthesis of 2-amino-3-cyano-4*H*-pyran (**ACP**) derivatives catalyzed by a water-soluble imidazolium-functionalized polymer (PEI.IL)—a synthetic enzyme (synzyme). Rather than focusing solely on synthetic scope, this study dissects how solvent properties and reaction equilibria govern reaction productivity and pathway selection in this multicomponent transformation. Solvent effects were quantitatively analyzed using the Kamlet-Abboud-Taft model, revealing that hydrogen-bond acidity (α), hydrogen-bond basicity (β), and dipolarity/polarizability (π*) play distinct and competing roles at different stages of the reaction. Control experiments, combined with systematic variation of reaction conditions, demonstrate that product formation proceeds predominantly through a pathway initiated by the reversible condensation between benzaldehyde and malononitrile, followed by enol-mediated a new C–C bond formation. Reaction productivity was treated as a thermodynamically oriented descriptor, allowing correlations between solvent effects, keto–enol equilibria, and overall efficiency to be established. The methodology affords a series of **ACP** derivatives in good to excellent yields under optimized conditions, with the catalyst remaining recyclable over multiple cycles without loss of activity. By integrating solvent-effect analysis, equilibrium considerations, and mechanistic control experiments, this work provides an experimentally grounded framework for understanding and optimizing multicomponent reactions beyond conventional yield-based approaches.

## Graphical abstract

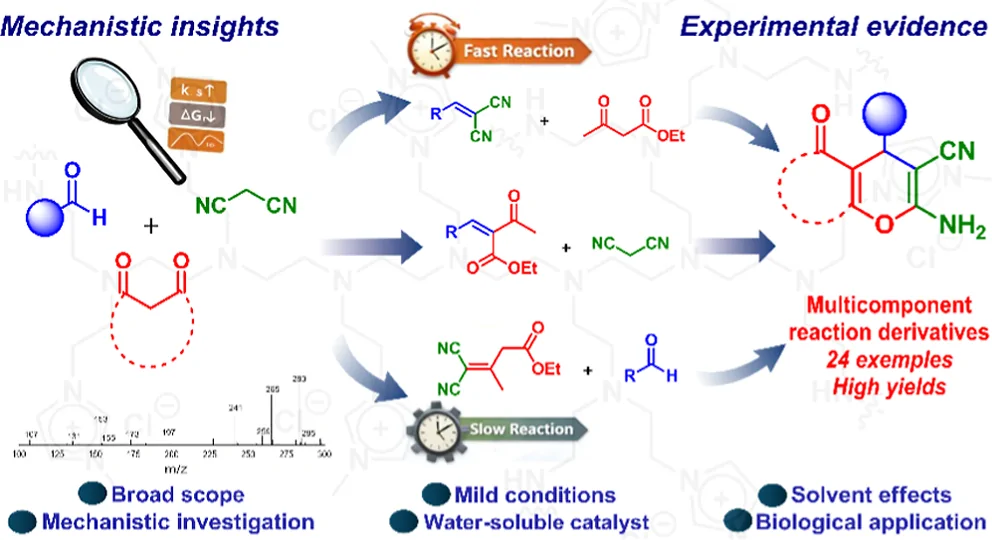

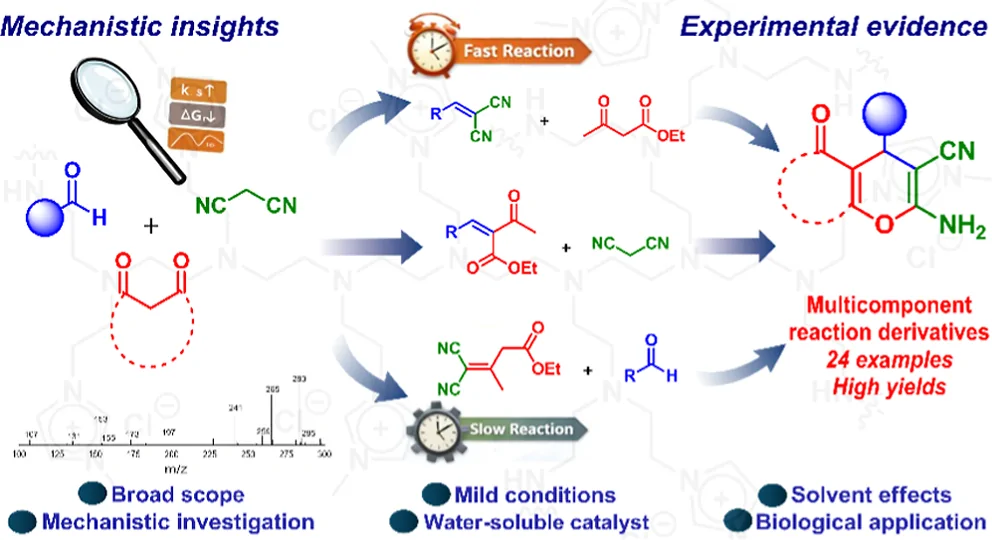

## Introduction

Multicomponent reactions (MCRs)^1–14^ are currently established as a key toolbox in organic chemistry.^15–17^ A large number of important studies on a wide range of different MCRs are found in the scientific literature, highlighting their significance and utility.^17–37^ Despite their undeniable importance to synthetic and medicinal chemistry,^38^ many aspects of MCRs still require urgent attention, particularly investigations and developments aimed at a deeper understanding of these challenging transformations. For example, solvent effects remain one of the most neglected aspects of MCRs.^39^ Mechanistic investigations supported by experimental evidence—rather than merely proposing “plausible mechanisms”—are, unfortunately, exceptions in the MCR literature.^40–42^

Since the breakthrough publication by Sherwood and co-workers,^43^ some groups—including ours—have focused on the application of solvent effect models to better understand multicomponent transformations.^44–52^ These few studies have demonstrated that valuable information can be obtained by examining solvent effects in these truncated multicomponent processes. This aligns with I. Ugi’s assertion^53^ that the mechanisms of any MCR cannot be fully elucidated using conventional methods, although essential mechanistic insights can be gained through a combination of multiple techniques. Still, a high level of detail is not typically expected.^53^

The synthesis of 2-amino-3-cyano-4*H*-pyran (**ACP**) derivatives via MCRs has emerged as an efficient and atom-economical strategy for constructing diverse heterocyclic frameworks with biological relevance (Scheme 1). Typically, these reactions involve a one-pot condensation of aldehydes, malononitrile, and 1,3-dicarbonyl compounds, such as ethyl acetoacetate or dimedone, under mild conditions. A wide range of catalysts has been employed to accelerate this transformation, including Lewis acids, Bronsted acids, ionic liquids, nanomaterials, and organocatalysts. This synthetic route offers significant advantages such as operational simplicity, high atom economy, and rapid access to functionalized pyran systems with good to excellent yields (Scheme 1).^54–57^

**Scheme 1:**
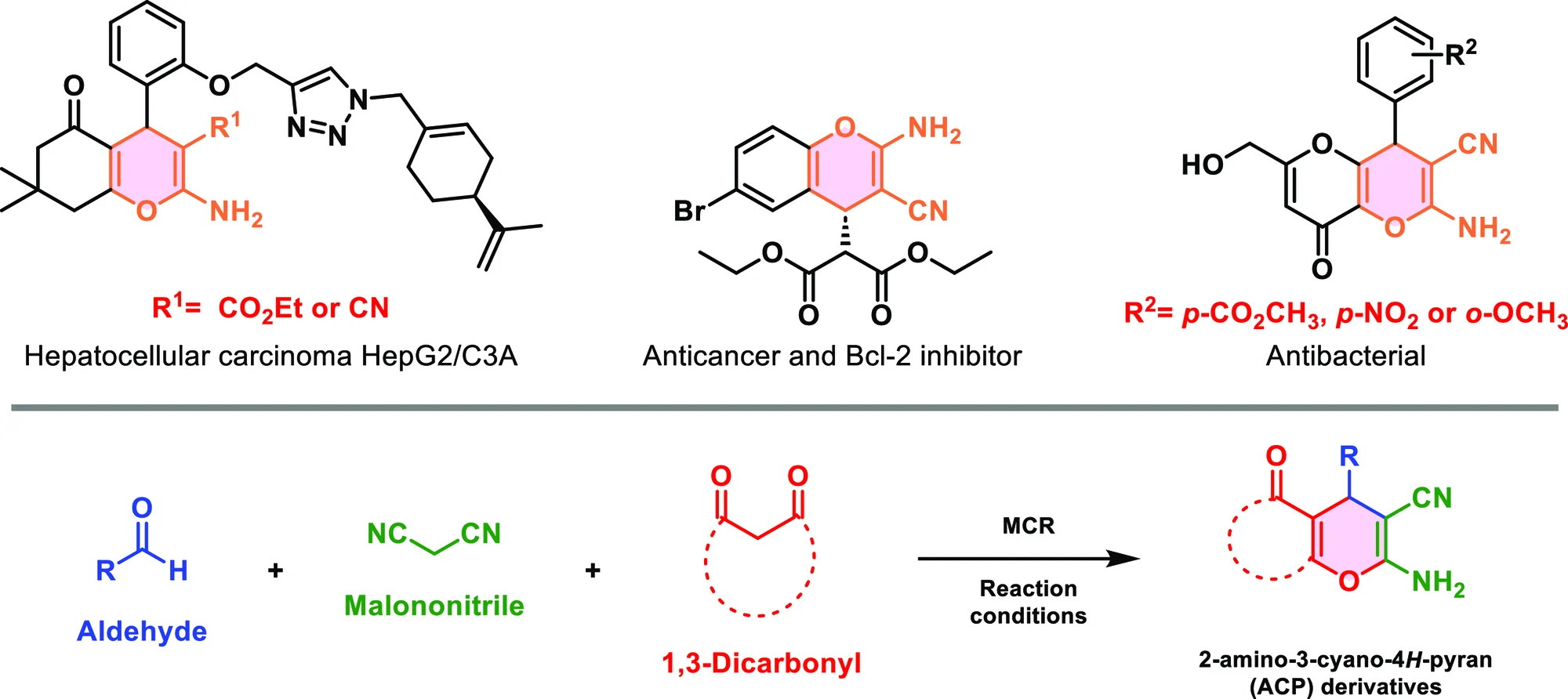
Examples of Bioactive 2-Amino-3-cyano-4*H*-pyran (**ACP**) Derivatives and Their Multicomponent Synthesis

Recent advances have focused on the development of greener and more sustainable catalytic systems.^58–61^ Biopolymer-supported magnetic nanocatalysts, such as Fe_3_O_4_@xanthan gum and Fe_3_O_4_@chitosan-Titriplex V composites, have demonstrated excellent catalytic activity, recyclability, and environmental compatibility under aqueous or ethanol-based conditions.^62,63^ Similarly, plant-derived catalysts, including aqueous extracts of *Elaeagnus angustifolia* leaves and onion peels, have enabled metal-free synthesis of pyran derivatives, highlighting the viability of natural resources in organic synthesis.^64,65^ Additionally, ionic liquids such as [C_4_C_1_Imim]­[BF_4_] have served dual roles as both solvents and reaction media, affording high yields and enabling catalyst recycling over multiple cycles.^66^

In addition, transition metal complexes, especially those of Mo­(VI), V­(IV), Cu­(II), and Ni­(II), have shown promising results in enhancing reaction rates and substrate scope. These catalysts often operate under mild to moderate temperatures and can be recovered from supported materials such as MCM-41, MOF structures, or polystyrene resins.^67–69^ Other examples are available and have succeeded in the multicomponent synthesis of **ACPs**.^70–79^ The topic of **ACPs** has been recently reviewed elsewhere.^80^

Although the articles are relatively rich in synthetic approaches and catalytic advancements, and despite considerable room for improvement, no study published so far has conducted a robust mechanistic investigation using techniques capable of identifying, isolating, or monitoring reaction intermediates with empirical validation. Proposed mechanisms lacking specific experimental confirmation still dominate the literature. This represents a significant research opportunity in the field, especially when combined with investigations on solvent effects and analytical techniques capable of monitoring the reaction transformation. Herein, we present our investigation on this important MCR and disclose our main findings.

## Results and Discussion

We initiate our investigation trying to find the best rection condition to produce **ACP** derivatives (**4**) via a catalyzed MCR approach (see Figure 1 and Table 1). Catalyst type, temperature, time and solvents were evaluated aiming at improving the formation of **ACP 4a** through a model reaction, i.e., mixing benzaldehyde, malononitrile and ethyl acetoacetate as the reagents and using equimolar quantities. A common feature in all MCR approaches to obtain **ACP** derivatives is the critical role of the catalytic system employed. In this context, we tested several catalysts, and the most promising was the so-called PEI.IL (Figure 1), a polyethylenimine (PEI) decorated with multiple imidazolium units bearing a hydrophilic anion to ensure water solubility. We originally developed this catalyst—a synzyme (synthetic enzyme type)—to accelerate aqueous dephosphorylation reactions,^81^ achieving improvements on the order of 2 × 10^6^ (*k*_cat_/*k*_non_). Later, we found that the designed PEI.IL could also be efficiently employed to perform MCRs,^82^ displaying a pronounced ionic liquid effect.^83–91^

**Figure 1:**
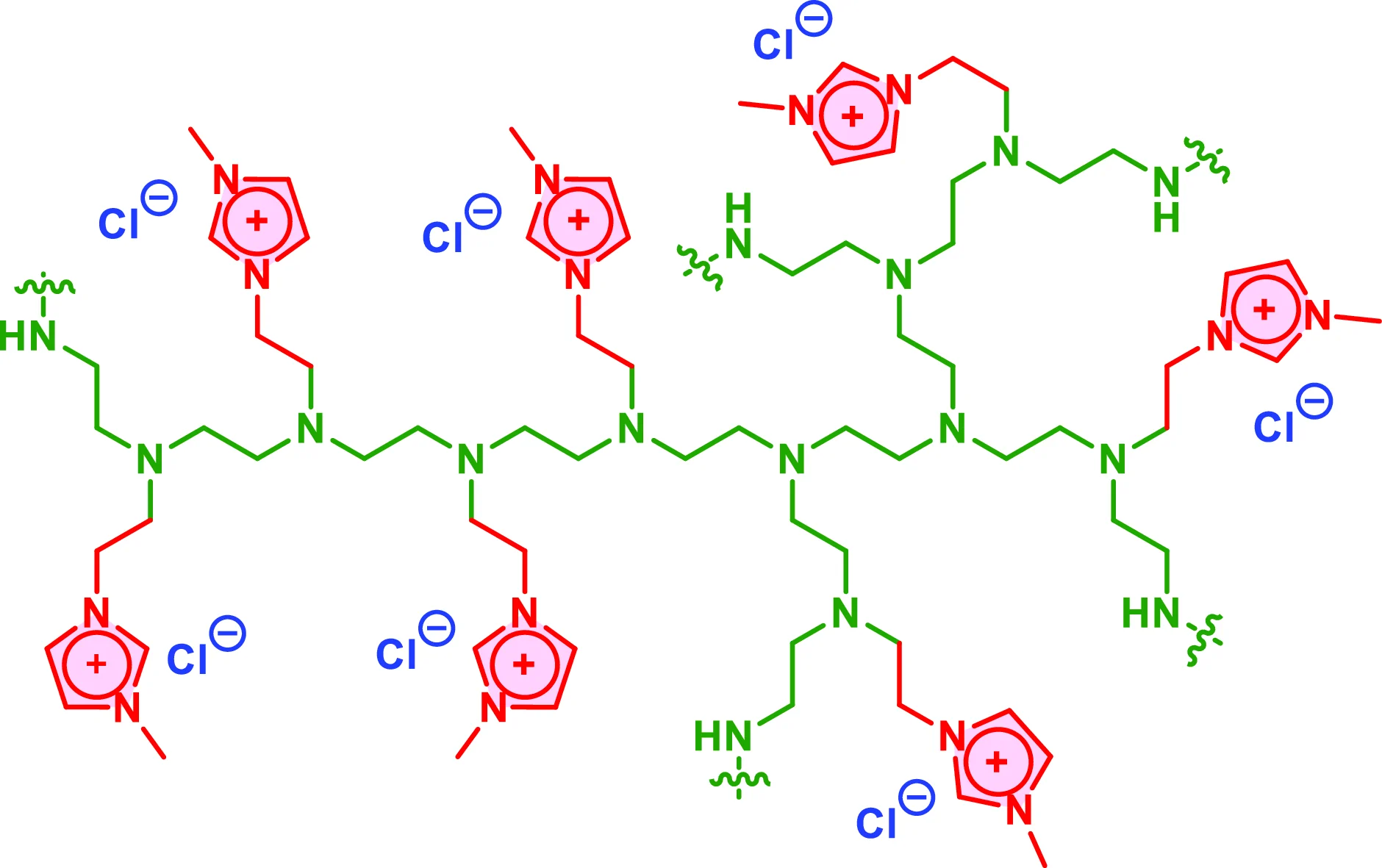
Structure of the water-soluble imidazolium-based catalyst PEI.IL (synthetic enzyme). Adapted from ref 81 Copyright 2015, American Chemical Society.

**Table 1:**
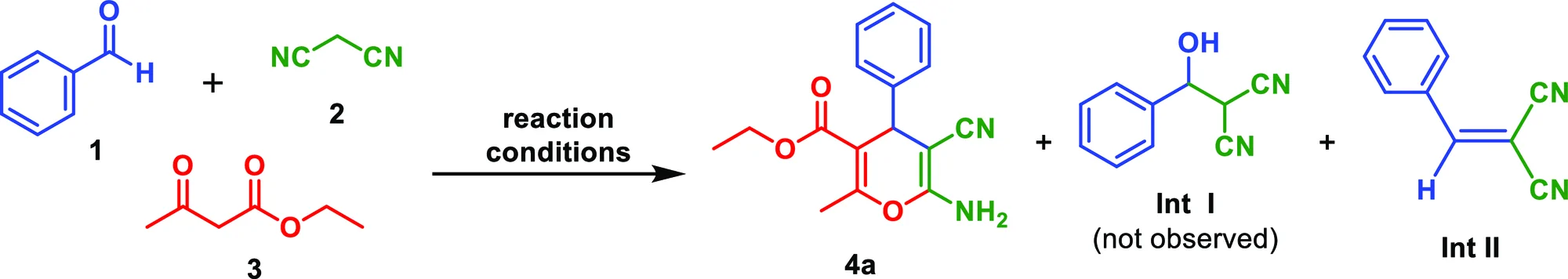

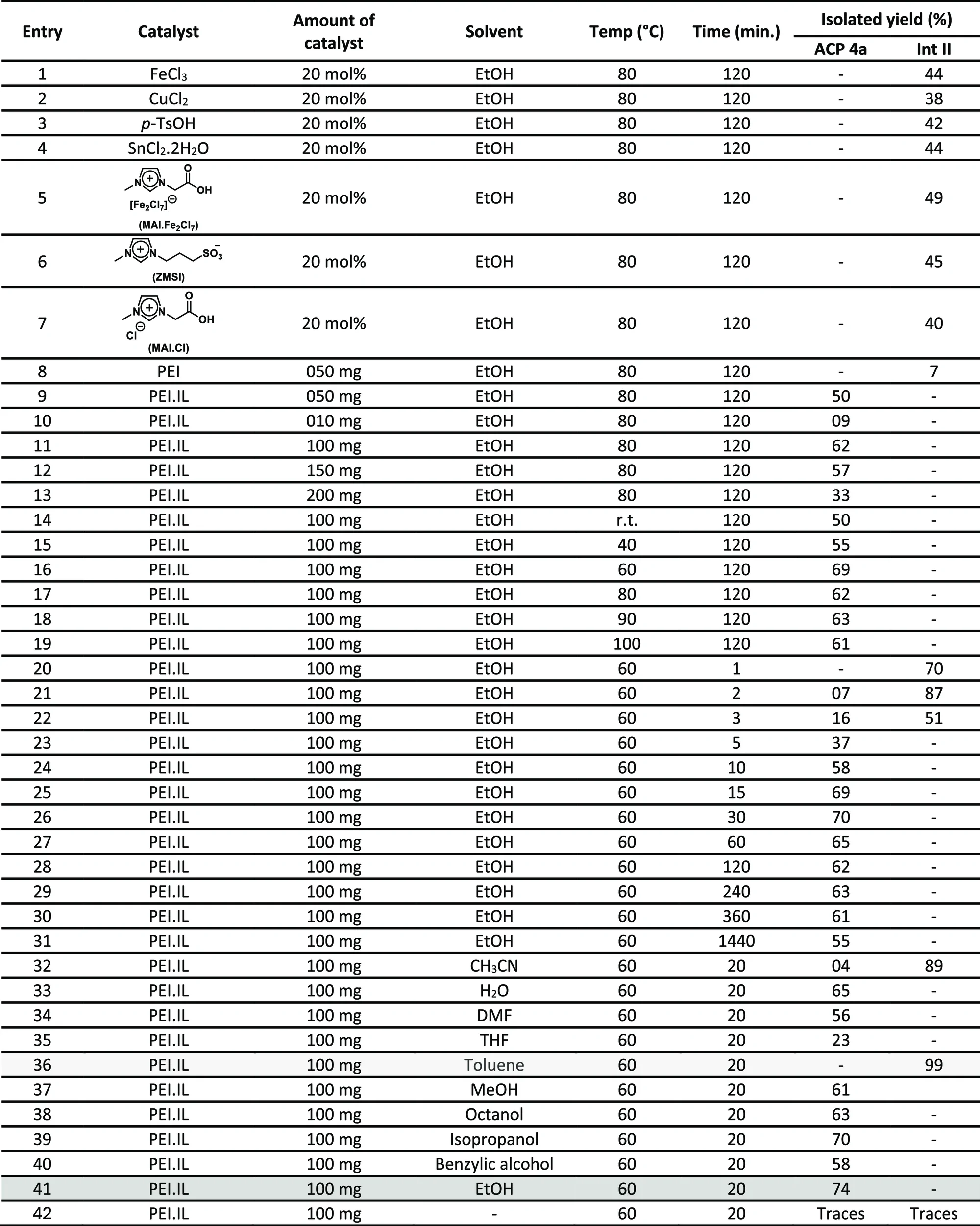
Multicomponent Synthesis of **ACP** derivative **4a** under Various Reaction Conditions^a^

a Reactions performed using 1 mL of solvent and 1.00 mmol of each reagent.

The combination of the designed PEI.IL, a reaction time of 120 min at 60 °C, 1.00 mmol of each reagent in 1 mL of ethanol, and 100 mg of catalyst afforded the desired multicomponent adduct in 74% yield (Table 1, Entry 41). All attempts to vary these conditions—temperature, time, solvent, catalyst type or catalyst amount—did not yield better results for the synthesis of **4a**. The results in Table 1 also reveal an important structural requirement for the catalytic system. PEI alone was unable to promote formation of the final MCR adduct and afforded only a small amount of **Int II**, indicating that the polymeric backbone by itself is insufficient to drive the productive pathway. The imidazolium-based catalysts evaluated in the absence of the PEI support favored formation of **Int II** but did not promote its conversion into **ACP 4a**. These observations indicate that the catalytic activity of PEI.IL does not arise from either component independently, but from a cooperative effect between the PEI scaffold and the appended imidazolium units. In this polyfunctional architecture, the PEI backbone may provide multiple sites and a substrate-organizing environment, whereas the imidazolium units and their counterions create a local ionic-liquid-like microenvironment capable of promoting substrate activation through ionic and hydrogen-bonding interactions. Such cooperative organization appears to be essential for shifting the reaction beyond **Int II** and enabling formation of the final MCR adduct.

A few words before the presentation and discussion of the mechanism are necessary regarding the formation of **Int II** (see Table 1). It is known that, due to the high reactivity of malononitrile, the Knoevenagel condensation between this reagent and the aldehyde—affording **Int II**—can occur under relatively mild temperature conditions^92,93^ and, in some cases, under catalyst-free conditions.^94^ The condition shown in Table 1 (entry 36) allowed the obtention of **Int II** through a Knoevenagel condensation in nearly quantitative yield, which is certainly interesting. The challenge, however, is to shift the complex equilibria evoked for the MCR—which will be discussed in due course—toward the consumption of **Int II** and the formation of **ACP 4a**.

As part of the reaction-condition optimization, we examined how changes in the stoichiometric ratio of the starting materials affected the formation of **ACP 4a**. The results showed that employing an excess of specific reagents consistently increased the reaction yield. Guided by this outcome, we designed a second set of experiments using excess reagent, and the corresponding data are presented in Table 2.

**Table 2:**
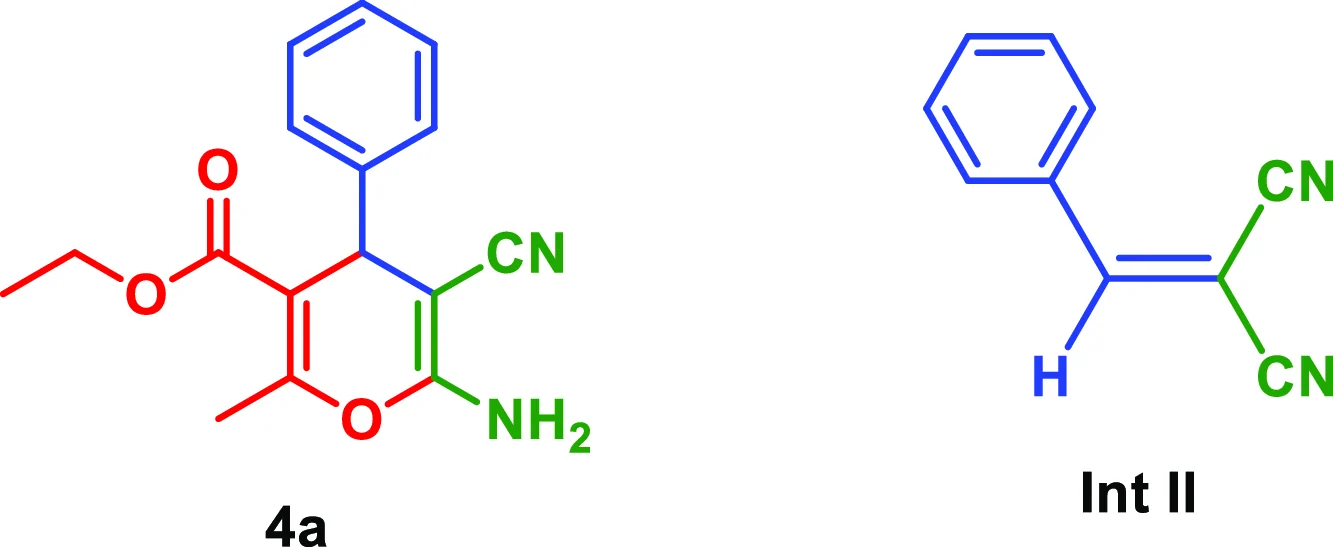
Study of Reaction Time Variation in the Presence of Excess Reagents^a^

		condition A		condition B		condition C	
entry	time (min)	**4a** (%)	int II (%)	**4a** (%)	int II (%)	**4a** (%)	int II (%)
1	1	N.O.	58	N.O.	55	N.O.	13
2	2	47	N.O.	N.O.	58	N.O.	15
3	3	50	N.O.	N.O.	73	N.O.	16
4	5	61	N.O.	N.O.	82	N.O.	52
5	10	83	N.O.	N.O.	92	N.O.	60
6	15	90	N.O.	5	92	N.O.	67
7	20	97	N.O.	30	N.O.	67	N.O.

a All yields refer to isolated compounds and were calculated based on the limiting reagents. N.O. = not observed. Condition A: 1.00 mmol benzaldehyde, 3.00 mmol malononitrile, 1.00 mmol ethyl acetoacetate, 60 °C, 1 mL EtOH, 20 min, PEI.IL (100 mg). Condition B: 3.00 mmol benzaldehyde, 1.00 mmol malononitrile, 1.00 mmol ethyl acetoacetate, 60 °C, 1 mL EtOH, 20 min, PEI.IL (100 mg). Condition C: 1.00 mmol benzaldehyde, 1.00 mmol malononitrile, 3.00 mmol ethyl acetoacetate, 60 °C, 1 mL EtOH, 20 min, PEI.IL (100 mg).

From Table 2, it is evident that malononitrile has the most significant influence on the reaction yields, and a few mechanistic implications will be addressed in due course. Once the optimal condition to favor the formation of **ACP 4** was established, the methodology was directly applied to the synthesis of a series of these compounds (Scheme 2).

**Scheme 2:**
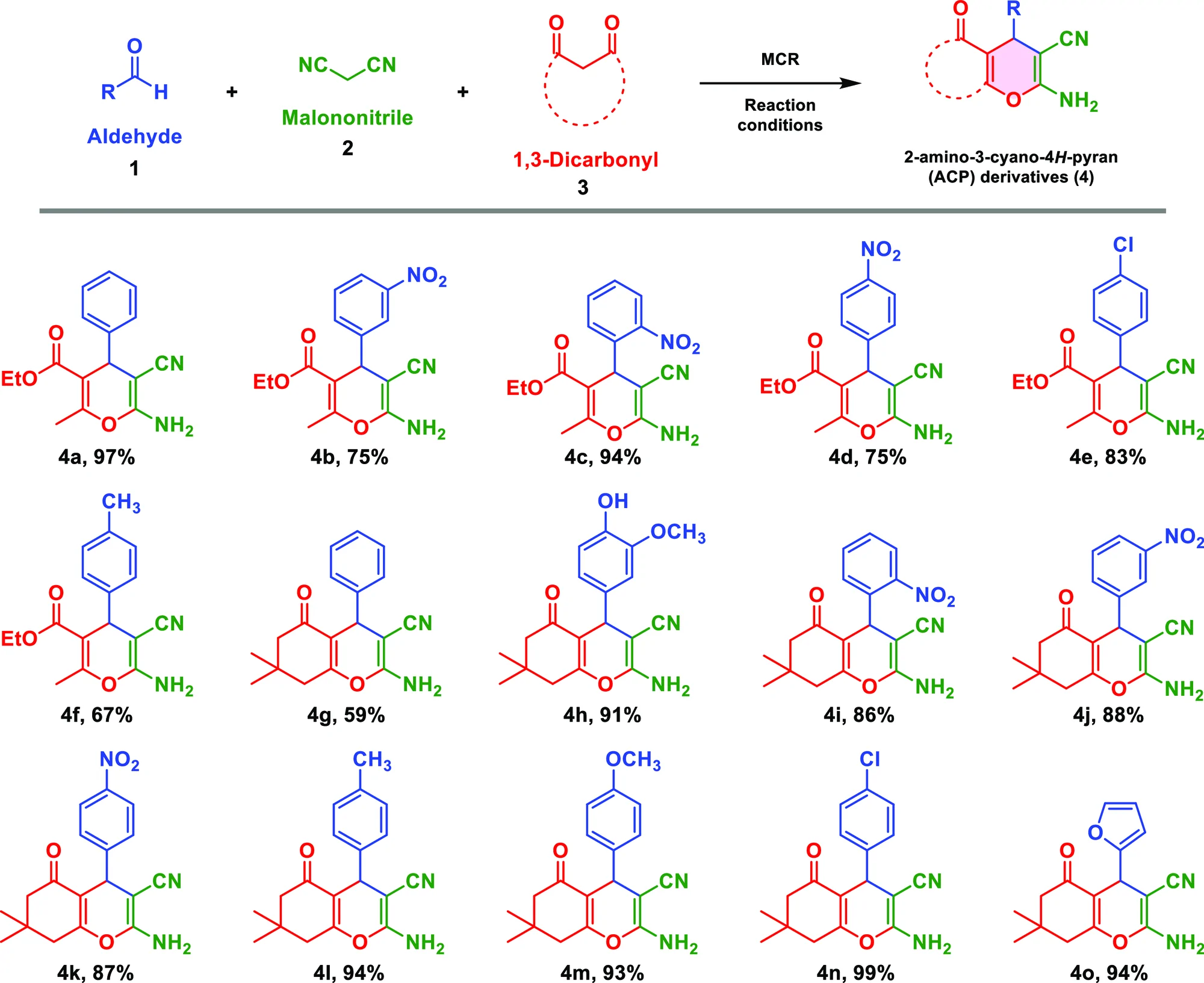
Synthesis of **ACP** Derivatives **4a**–**o** Using the Optimized Reaction Conditions Employing the Ionically-Tagged Synthetic Enzyme (PEI.IL)^a^

a Reaction conditions: 1.00 mmol aldehyde, 3.00 mmol malononitrile, 1.00 mmol 1,3-dicarbonyl derivative, 60 °C, 1 mL EtOH, 20 min, PEI.IL (100 mg).

The reaction performed under optimized conditions also enabled catalyst recycling for at least 11 consecutive cycles without any notable loss of catalytic activity and allowed scale-up to multigram synthesis with excellent yields (details in the Experimental Section). Minor variations in yields (Figure S2) were observed; however, these were not associated with catalyst deactivation but rather with differences in purification efficiency or small losses occurring during product isolation. The successful obtention of several **ACP 4a-o** derivatives with overall good to excellent yields prompted us to investigate the use of a reactive “1,3-dicarbonyl” compound. In this context, ethyl acetoacetate was replaced by 4-hydroxycoumarin to obtain an additional series of **ACP 4** derivatives. The use of the more reactive reagent, which has a permanent “enol” preference, and under optimized conditions, also afforded the desired multicomponent adducts with good to excellent yields (Scheme 3).

**Scheme 3:**
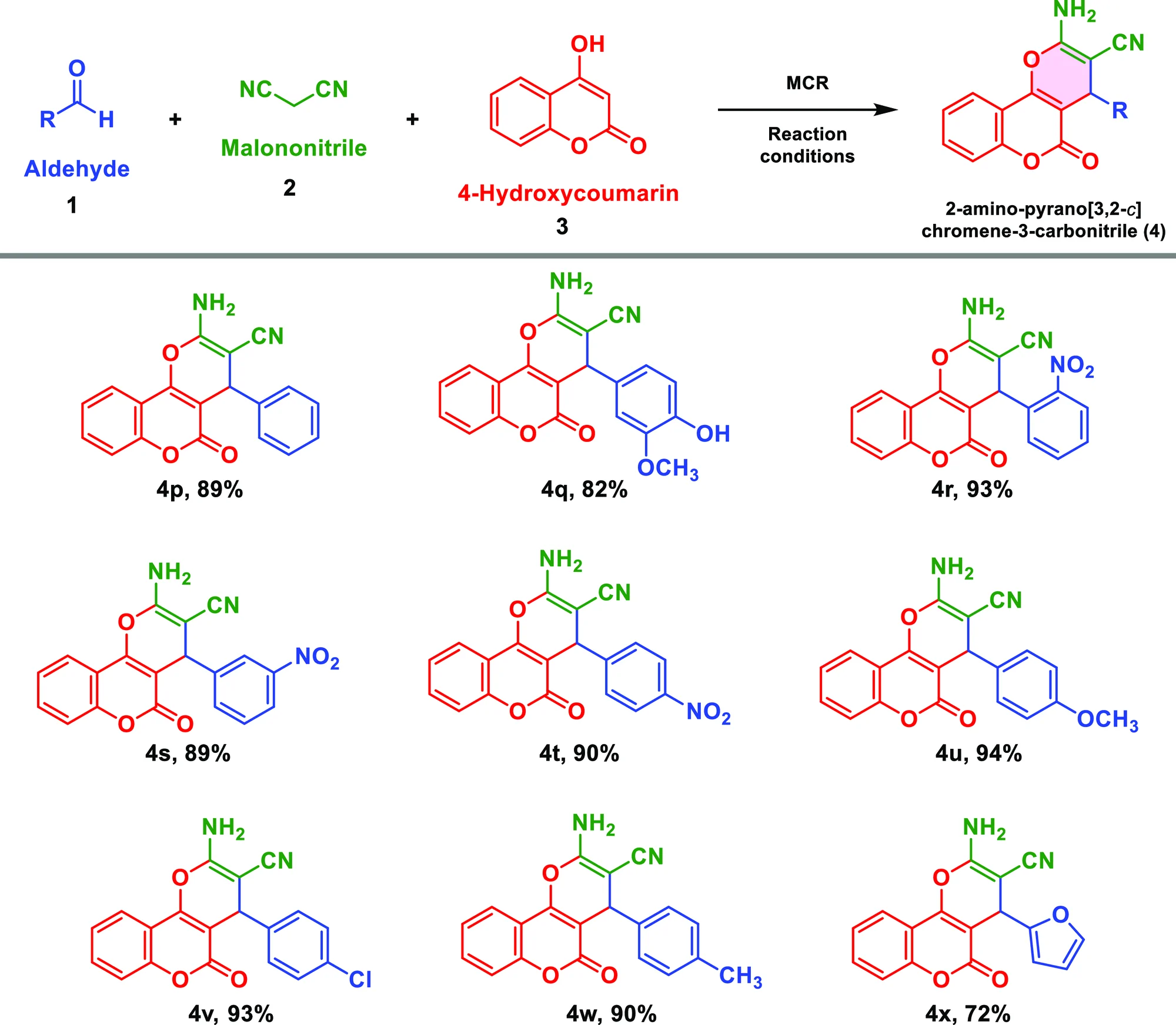
Synthesis of **ACP** Derivatives **4p**–**x** Using the Optimized Reaction Conditions Employing the Ionically-Tagged Synthetic Enzyme (PEI.IL)^a^

a Reaction conditions: 1.00 mmol aldehyde, 3.00 mmol malononitrile, 1.00 mmol 4-hydroxycoumarin, 60 °C, 1 mL EtOH, 20 min, PEI.IL (100 mg).

The solvent clearly influenced the reaction, and depending on the solvent chosen, the reaction could be tuned to afford either the MCR adduct or **Int II** exclusively (see Tables 1 and 2). To determine which parameters were effectively influencing the outcome, we applied the robust Kamlet–Abboud–Taft (KAT)^95–99^ solvent model (Figures 2 and S1). To quantify how changes in solvent translate into overall efficiency, reaction productivity (*P*)^43^ can be treated as a thermodynamically oriented metric defined as the natural logarithm of the molar ratio between the isolated MCR adduct (**ACP 4**) and the unreacted yield-limiting reagent (i.e., malononitrile). In this framework, the reference component is selected based on which reagent effectively constrains product formation, so *P* behaves analogously to an equilibrium-like descriptor and allows yield trends to be interpreted within a multiequilibria reaction network rather than as a purely kinetic output.^43^ For the present **ACP**-forming system, malononitrile is an appropriate reference because it participates in a fast reversible addition with benzaldehyde (affording **Int I**), and its residual amount directly reflects how efficiently the system is driven away from reversible intermediates toward the final MCR product. Additionally, malononitrile was the excess reagent required to achieve improved yields. Accordingly, ln (*P*) provides a single response variable that captures the net driving force of the transformation and enables direct correlation of reaction efficiency with solvent descriptors.

**Figure 2:**
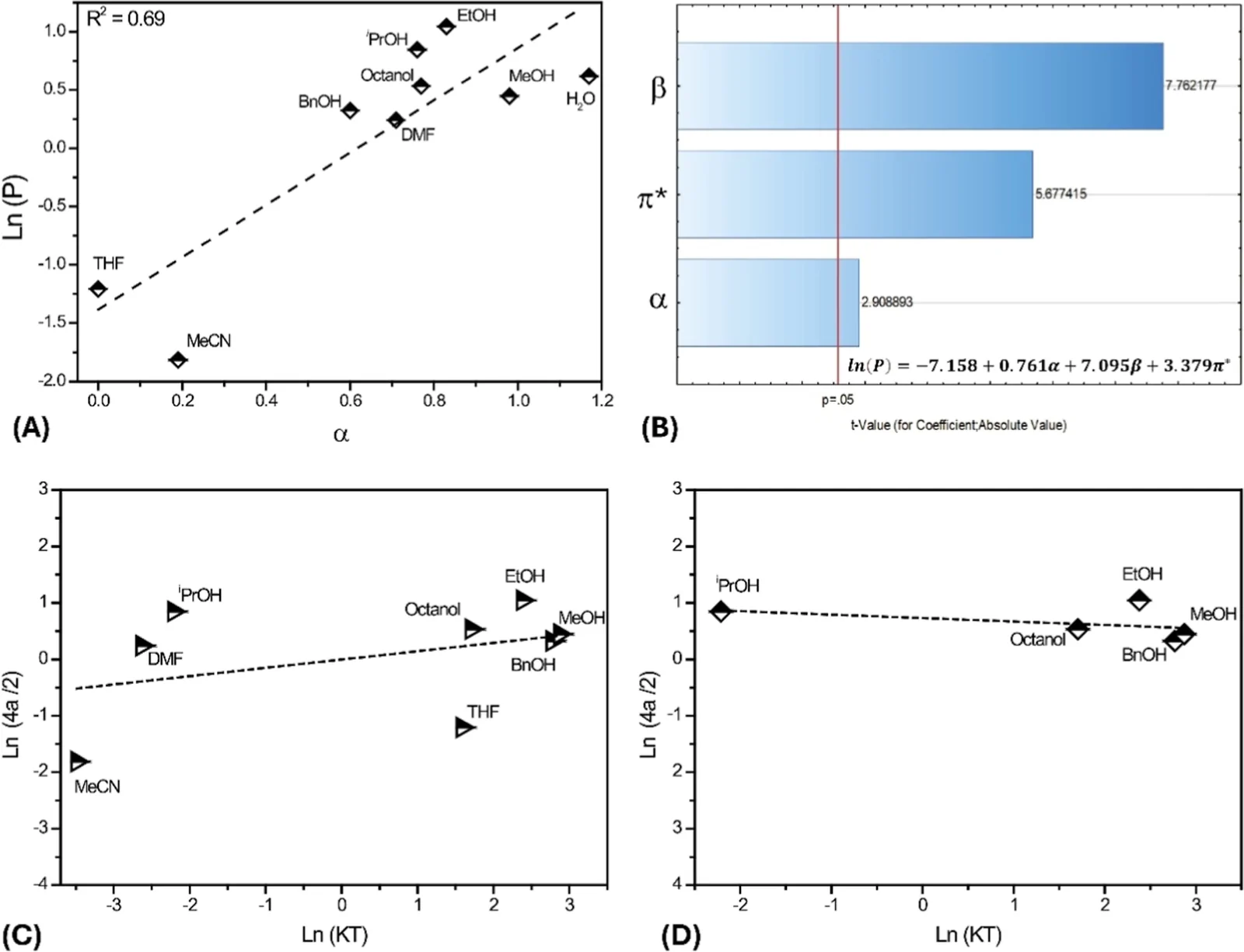
(A) Influence of hydrogen bond acidity (α) on the reaction outcome toward the formation of **4a**. (B) Multivariate analysis based on the KAT descriptors indicating that all three parameters influence reaction productivity. ln (*P*) = −7.158 + 0.761α + 7.095β + 3.379 π*. (C) Catalyzed reactions showing the influence of all tested solvents on the keto–enol equilibrium, ln­(KT), and on the yield of **ACP 4** formation. (D) Catalyzed reactions showing the influence of all tested alcohols, used as solvents, on ln­(KT) and on the yield of **ACP 4** formation. Note: *P* = [**4a**]/[**2**] and KT = [enol]/[keto] for the ethyl acetoacetate reagent.

In this context, the parameter that showed the strongest correlation was hydrogen bond acidity (α), with an *R* ^2^ of 0.69. However, to better understand the effects related to the solvent and the influence of each parameter, a multivariate analysis was conducted. Relationships between the KAT descriptors (independent variables) were examined using a general regression approach, which is fundamental for developing reliable multivariate models capable of accurately predicting reaction productivity (dependent variable). A significance level of *p* = 0.05 was established as the threshold for statistical significance of the regression model parameters.

The simple Knoevenagel condensation has been studied using KAT parameters, and in that case, β had a direct influence on the reaction outcome.^100^ Although toluene has a low bulk β value, the nearly quantitative formation of **Int II** under these conditions should not be attributed to the solvent alone, but rather to the local ionic-liquid-like microenvironment provided by PEI.IL. In this environment, the imidazolium units and their counterions can promote substrate activation and facilitate the Knoevenagel condensation, in accordance with the literature on ionic-liquid-promoted Knoevenagel condensations.^100^ The known KAT values for ethanol are 0.83 (α), 0.77 (β), and 0.62 (π*), respectively. The β value of this alcohol is not negligible and certainly contributes to the formation of **Int II**. However, the elevated α value also influences the reaction by promoting the conversion of this intermediate into the final adduct. In contrast, toluene has an α value of 0.00; therefore, the reaction does not progress from **Int II** to the final product, as observed in Table 1 (entry 36). Therefore, ethanol is an “ideal” solvent, not only for providing sustainable and environmentally friendly reaction conditions, but also because its β descriptor favors intermediate formation, while its α value has a more direct influence on promoting the formation of the final adduct.

The π* parameter also plays a role in the transformation, therefore, its influence should be elucidated and explained in light of the possible mechanism of the multicomponent transformation. The polarity of the reagents and possible intermediates in the transformation already points to the influence of this parameter on the reaction outcome by favoring the stabilization of charged and polar intermediates and transition states.

The combination of solvent effects and the presence of the catalyst PEI.IL also had an important influence on the keto–enol equilibrium (KT), which is a key aspect to be considered in the mechanistic analysis, especially because the enol tautomer has been identified as the reactive species in the addition to the iminium intermediate in the Biginelli MCR.^43^ This equilibrium could be readily determined by ^1^H NMR spectroscopy (Figures S3–S11), and it is well established that the solvent plays a central role by exerting a direct influence on the tautomeric equilibrium.^101^ Pure ethyl acetoacetate (no solvent) in the presence of the catalyst PEI.IL shifts the keto–enol equilibrium toward the keto tautomer, as our group previously demonstrated for a different MCR.^82^ The analysis performed in different solvents (Table 3) clearly showed that the catalyst plays a vital role in shifting this equilibrium toward the keto isomer and, therefore, that enol restoration is intimately linked to solvent effects.

**Table 3:**
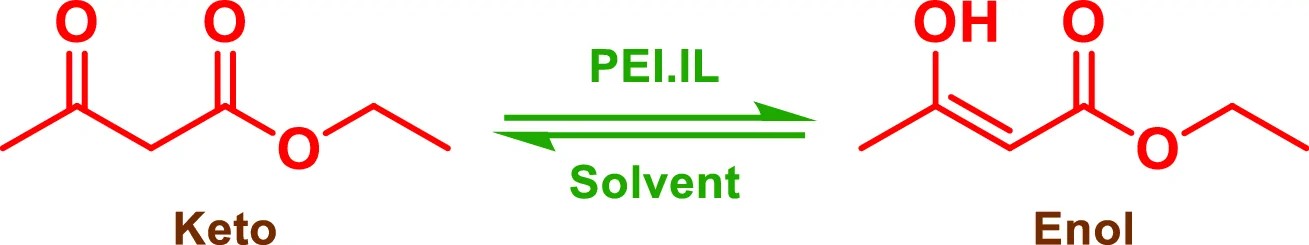
Ethyl Acetoacetate Keto–Enol Equilibrium in Different Solvents and in the Presence of the Catalyst PEI.IL, as Determined by ^1^H NMR

solvent	integral value (%) (enol)	integral value (%) (keto)	KT = $\frac{\left[Enol\right]}{\left[Keto\right]}$	ln (KT)
MeCN	02.97	97.03	00.03	–3.49
DMF	06.72	93.28	00.07	–2.63
THF	83.03	16.97	04.89	1.59
isopropanol	09.87	90.13	00.11	–2.21
benzyl alcohol	94.10	05.90	15.94	2.77
ethanol	91.53	08.47	10.80	2.38
methanol	94.64	05.36	17.65	2.87
octanol	84.62	15.38	05.50	1.71

Based on the data from Table 3, Figure 2C,D were plotted. Analysis of these panels in Figure 2 together with Table 3 shows that benzyl alcohol, ethanol, and methanol are the most effective solvents in favoring the enol tautomer in the presence of the catalyst. It is important to stress that multiple factors can influence this equilibrium and that, under different catalytic conditions, it may shift toward one tautomer or the other. Although these three alcohols favor the enol isomer, the presence of PEI.IL has a marked effect and strongly favors the keto form. Among these three solvents, the advantages of using ethanol are obvious due to safety and environmental considerations. When the 1,3-dicarbonyl reagent is changed from ethyl acetoacetate to 4-hydroxycoumarin, the solvent also plays an important role, as discussed above, but not in favoring the enol tautomer, since the coumarin derivative is “locked” in its enol form regardless of the solvent.

One final aspect of using ethanol arises when the 1,3-dicarbonyl compound is cyclic. When a cyclic 1,3-dicarbonyl component is employed, the role of the solvent changes fundamentally compared with acyclic systems. As demonstrated by Sherwood and co-workers,^43^ the enol form of cyclic diketones is strongly favored as the β increases. This behavior arises because cyclic 1,3-dicarbonyl compounds are conformationally unable to establish stabilizing intramolecular hydrogen bonds. Consequently, enol stabilization becomes highly dependent on solvent–solute hydrogen bonding. Solvents with higher β values, such as ethanol, efficiently stabilize the enol tautomer through external hydrogen-bond interactions, shifting the keto–enol equilibrium decisively toward the reactive enol species. In this work, similar effects were observed, further justifying ethanol as the most suitable solvent among those evaluated for obtaining **ACP** derivatives under the optimized conditions described herein.

Once the solvent influence was clarified and ethanol was selected and justified as the optimal medium for the reaction, we turned our attention to the mechanism of these two MCRs, that is, one considering ethyl acetoacetate as the reagent and the other employing 4-hydroxycoumarin. A mechanistic investigation was necessary to deepen our understanding of this transformation. The cited literature proposes—without any available experimental or theoretical evidence—two possible reaction pathways to generate **ACP 4a** (Scheme 4). However, three competing reactions can be expected to form the first important reaction intermediate: **Int II**, **Int III**, or **Int V**.

**Scheme 4:**
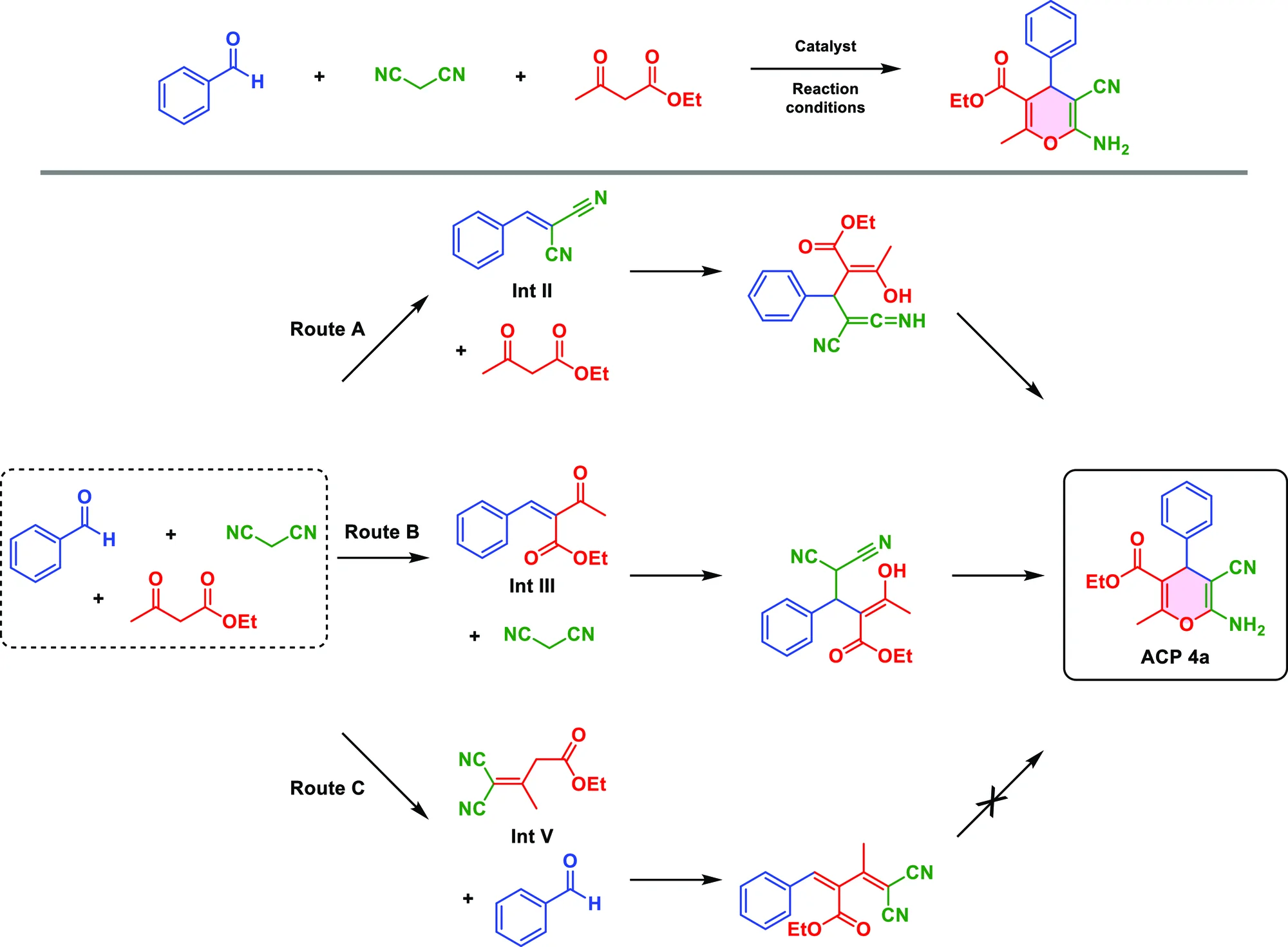
Possible Reaction Pathways for the Formation of **ACP 4a** ^a^

a The literature indicates routes A and B as plausible pathways, although the formation of **Int V** is a possibility not yet discussed.

To further improve our understanding of the mechanism, a series of control experiments was conducted (Scheme 5). With these results in mind, combined with the information obtained from solvent effects (Table 1 and Figure 2), and reaction condition optimization (Table 2), some mechanistic considerations could be drawn. First, the reaction of the two partners, aldehyde and malononitrile (95% yield), is considerably more favored when compared with the other two possible combinations, namely the aldehyde with ethyl acetoacetate (23% yield of **Int III**) or the malononitrile with the 1,3-dicarbonyl derivative (7% yield of **Int v**). The isolated **Int II** could be efficiently transformed into the final MCR adduct in good yield (75%), as noted in Scheme 5. These results point to a faster formation of **Int II** when compared with the other two possible reaction pathways that afford **Int III** or **Int V**, respectively. Once formed, **Int II** is also rapidly transformed into the final desired **ACP 4**.

**Scheme 5:**
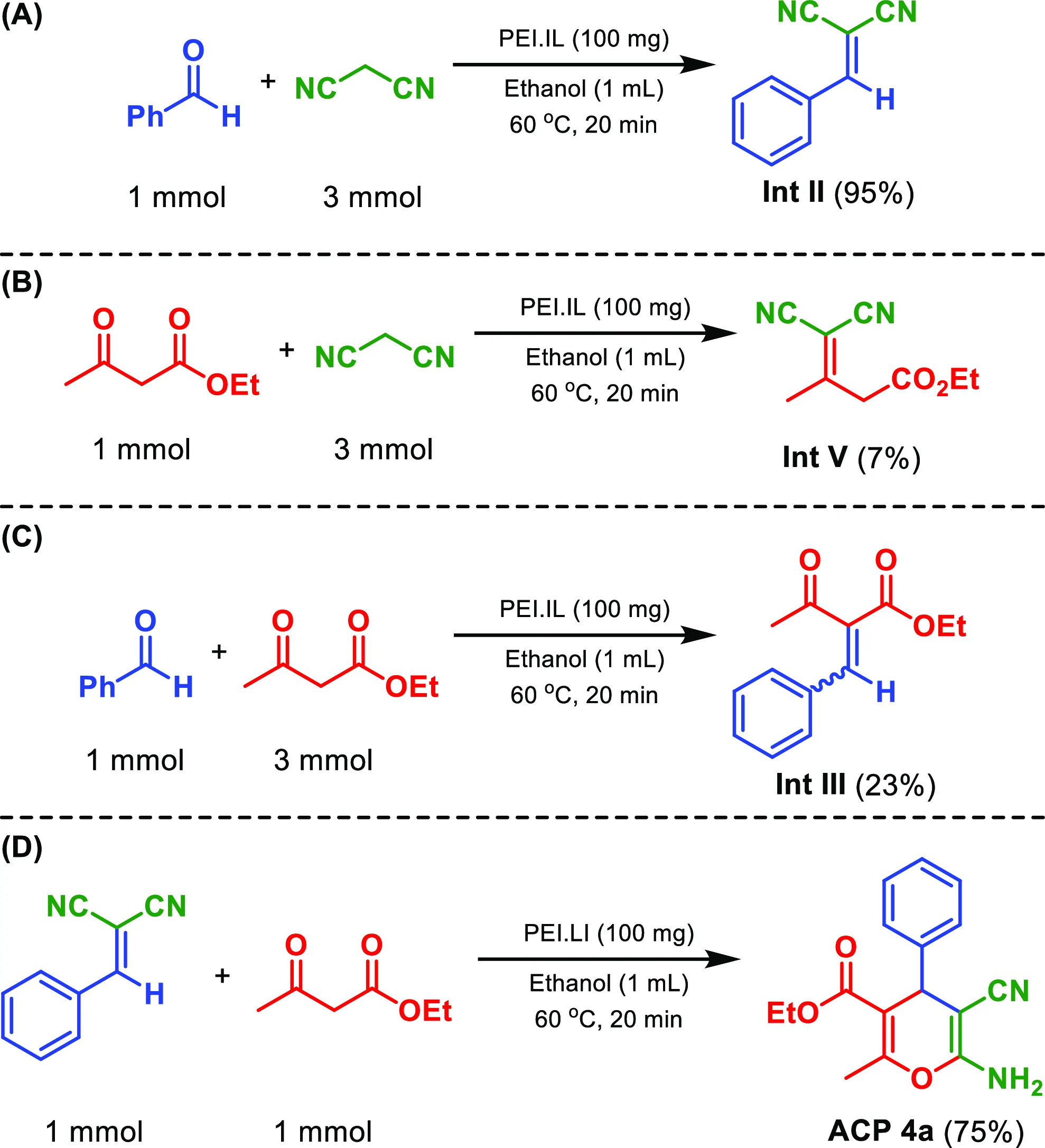
Control Experiments to Elucidate the Preferential Mechanistic Route for Obtaining **ACP 4a**

An in-depth analysis of the results shown in Scheme 5 allows a clear association between solvent effects and the trends observed in this study. The inverse relationship between the rate of carbonyl addition and the β descriptor, a well-known phenomenon,^102,103^ can be rationalized by considering how strongly basic solvents interact with carbonyl substrates. As supported by earlier reports,^102,103^ solvents with higher β values (such as ethanol) act as efficient hydrogen-bond acceptors and preferentially stabilize the carbonyl oxygen in the ground state through strong solvent–solute interactions. This stabilization reduces the effective electrophilicity of the carbonyl carbon and lowers the population of reactive, unsolvated conformations. In contrast, the transition state for nucleophilic addition benefits less from this stabilization, leading to an increased activation free energy. As a consequence, stronger hydrogen-bond basicity selectively stabilizes the reactant state relative to the transition state, resulting in slower rates of carbonyl addition. Taken together, these studies establish β as a critical solvent descriptor that modulates carbonyl reactivity by governing ground-state stabilization rather than transition-state acceleration.

The reaction between malononitrile and benzaldehyde under the optimized conditions (Scheme 5A) afforded the desired **Int II** in 95% yield in ethanol (β = 0.77), but reagent excess was required. By contrast, inspection of Table 1 (entry 36) shows that when toluene was used as the solvent (β = 0.11), **Int II** was formed in nearly quantitative yield without requiring reagent excess. In this context, the higher β value of ethanol helps to explain the need for excess malononitrile to efficiently promote the Knoevenagel condensation. Analysis of Scheme 5B makes this ethanol effect even clearer. Although the presence of PEI.IL favors the keto tautomer, which would in principle promote the addition of excess malononitrile to the C=O group of the ketone, this process is strongly hindered by solvent effects. As a result, formation of the desired **Int V** occurs only in very low yield.

Scheme 5C is somewhat paradoxical, displaying contrasting effects on each reagent. While ethyl acetoacetate shows a preference for the keto isomer in the presence of PEI.IL, ethanol also facilitates formation of the enol tautomer, which in turn reacts with the C=O group of the aldehyde, an addition that is not favored in ethanol due to its high β value. In this specific reaction, however, the solvent also exerts a beneficial effect by promoting formation of the reactive enol isomer, but a detrimental effect on the nucleophilic addition of the enol to the electrophilic aldehyde.

In Scheme 5D, ethanol favors enol formation and, consequently, formation of the final **ACP 4a** in good yields. In this step, α and π*, as discussed above, also influence the reaction outcome and favor formation of the desired MCR adduct. Considered together, the solvent effects discussed herein, combined with the control experiments (Scheme 5), already indicate that r—oute A (see Scheme 4) is the favored reaction pathway. However, additional evidence was sought to unequivocally confirm this preference.

To gain insights into the reaction mechanism, we decided to monitor the multicomponent transformation using electrospray (tandem) mass spectrometry—ESI–MS­(/MS).^104–107^ This technique is known for its efficient “fishing” of intact ions from solution and the gentle transfer of those ions to the gas phase.^108–110^ The fast and high-performing ESI analysis allows the detection and characterization, via MS/MS, of transient species,^111–113^ allowing effective monitoring of the transformation and providing compelling evidence of the reaction mechanism.^114–116^ As we have pointed out, ESI–MS­(/MS) is the most promising tool to elucidate MCR mechanisms.^42^

In this context, we monitored by ESI–MS­(/MS) two different reactions under the optimized conditions: one obtained by mixing benzaldehyde, malononitrile, and ethyl acetoacetate, and the other by replacing the 1,3-dicarbonyl reagent with the 4-hydroxycoumarin derivative. The results are presented in Figures 3 and 4, respectively. The MS signals were not considered as absolute concentrations, since different intermediates and products may display different ionization efficiencies under ESI conditions. Instead, extracted ion intensities for the assigned species were normalized and used as relative time-dependent descriptors of the reaction composition. Thus, the kinetic treatment should be regarded as an apparent kinetic model based on normalized MS response profiles, aimed at comparing the relative evolution of intermediates and products and at distinguishing productive from nonproductive pathways. This approach allowed the experimental MS trends to be fitted to the proposed differential models and provided quantitative support for pathway selection without assuming identical response factors for all detected ions, as noted in many reports.^117–119^

**Figure 3:**
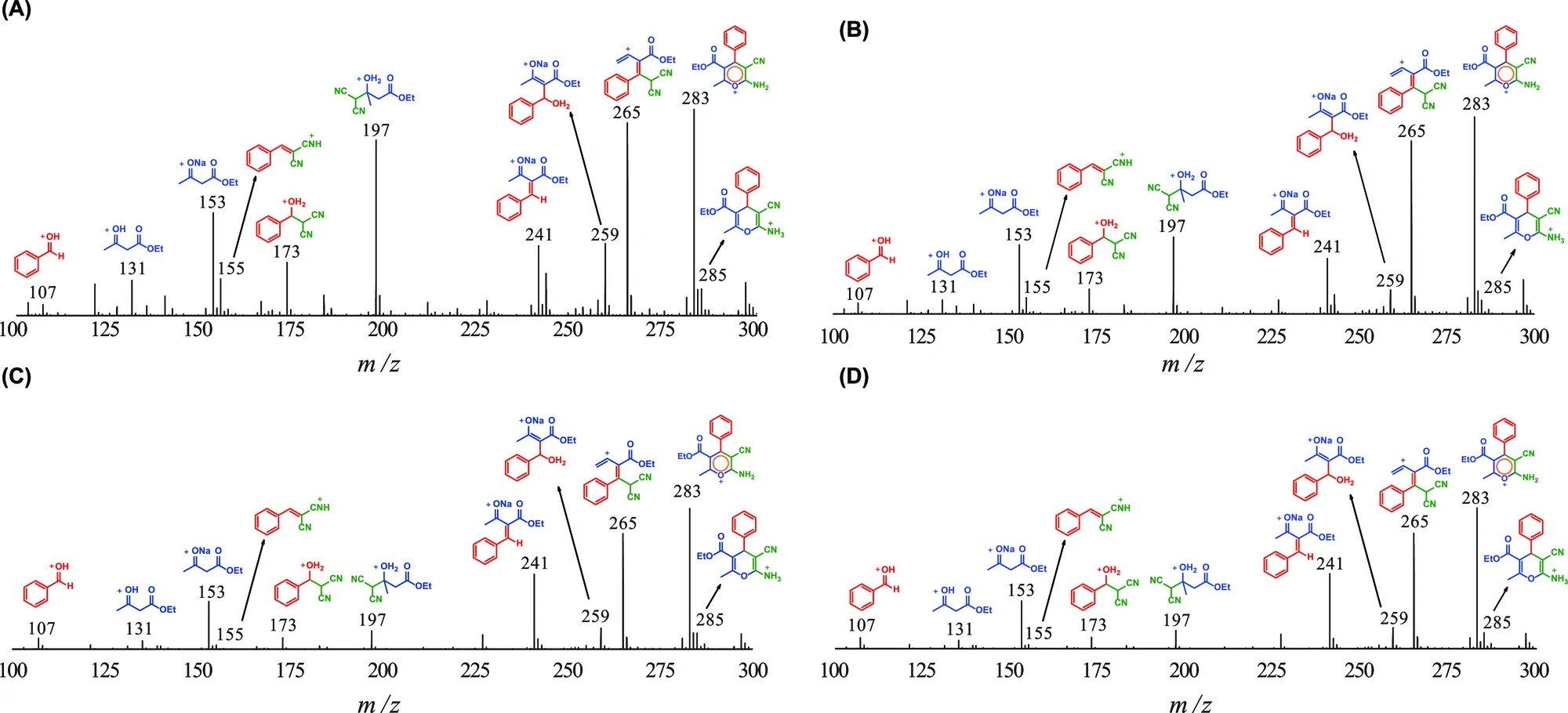
ESI­(+)–MS­(/MS) monitoring of the reaction mixture containing benzaldehyde, malononitrile, and ethyl acetoacetate over time: (a) 5 min, (b) 30 min, (c) 60 min, and (d) 120 min.

**Figure 4:**
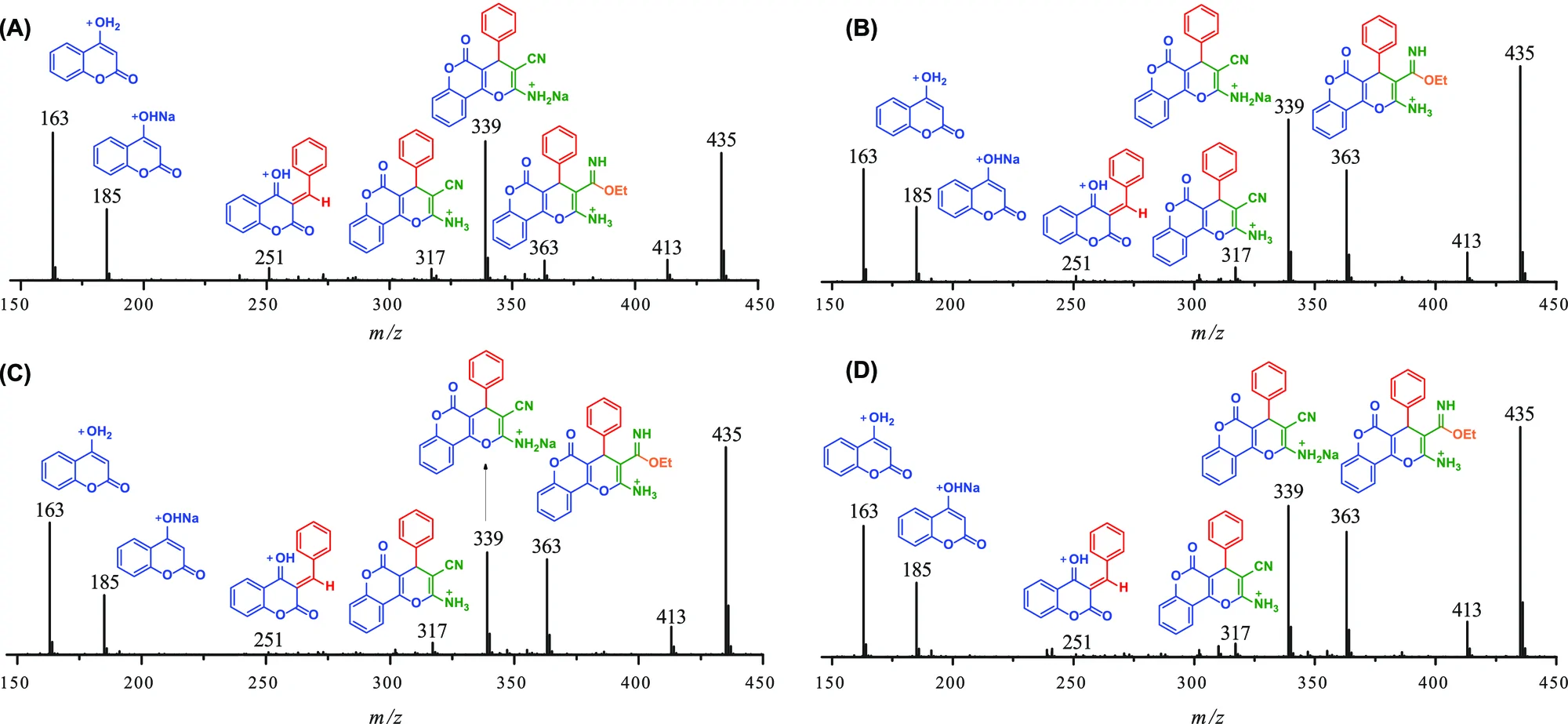
ESI­(+)–MS­(/MS) monitoring of the reaction mixture containing benzaldehyde, malononitrile, and 4-hydroxycoumarin over time: (a) 5 min, (b) 30 min, (c) 60 min, and (d) 120 min.

The monitoring of these two MCRs allowed the interception and structural characterization, via MS/MS, of key intermediates and products over time (Figures S12–S29). Additionally, both reactions proceeded as expected but with significant kinetic differences, as explored below. These differences are expected since the coumarin reagent has a permanent and locked “enol” preference,^120–122^ whereas the enol tautomer in ethyl acetoacetate is solvent dependent.^39,43,49^ At first glance, the reaction monitoring over time does not provide an immediate understanding of the multicomponent transformation, but plotting and analyzing the intensities against time (Figure 5) returned an elucidative interpretation and also allowed a kinetic model to be proposed (Scheme 6). As noted in Scheme 6, all intermediates and products could be efficiently detected during the monitoring. The kinetic models also contemplated both the possible reaction pathways and side reactions predicted for these two multicomponent transformations.

**Figure 5:**
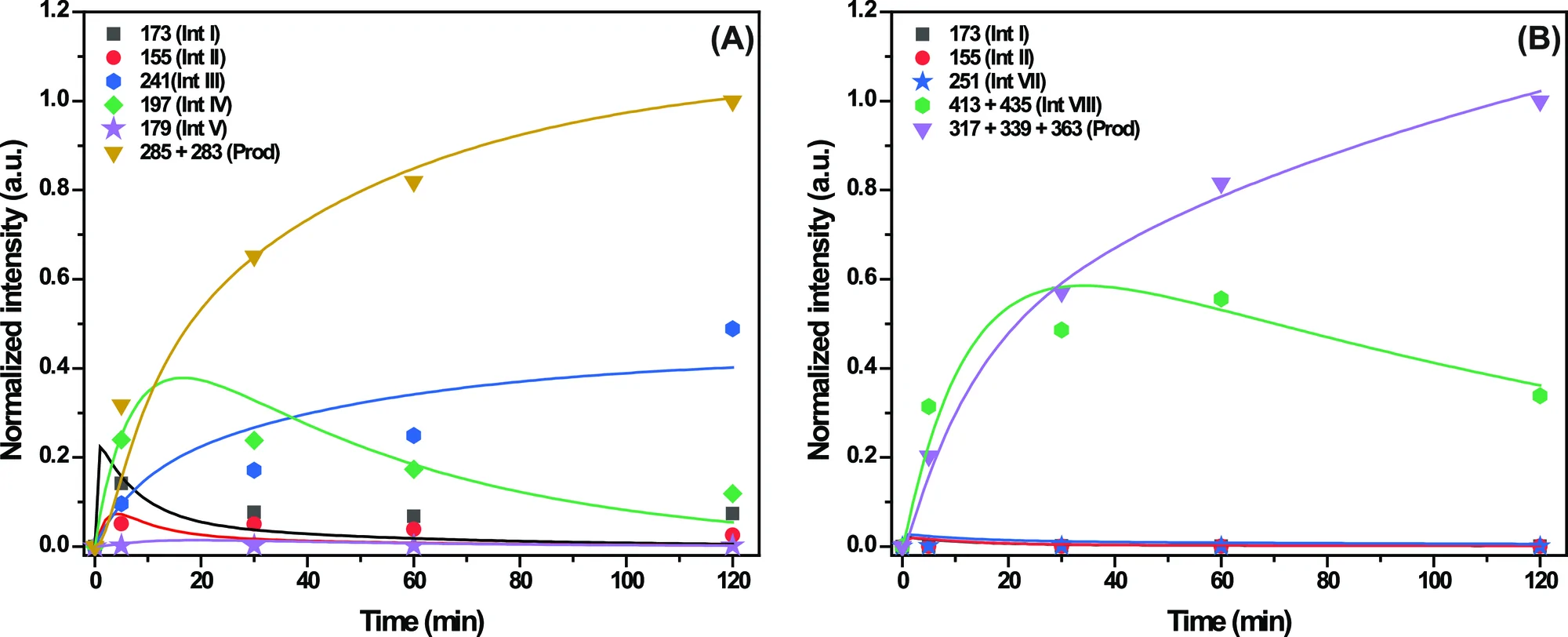
Experimental data of the reaction monitoring over time. (a) Ethyl acetoacetate as the reagent and (b) 4-hydroxycoumarin as the reagent. Predictions of the differential reaction model using the set of kinetic parameters estimated through a sequential optimization procedure. The solid lines represent the model predictions of the normalized intensity of the components in the reaction system, while the symbols denote the normalized experimental data from mass spectrometry signals.

**Scheme 6:**
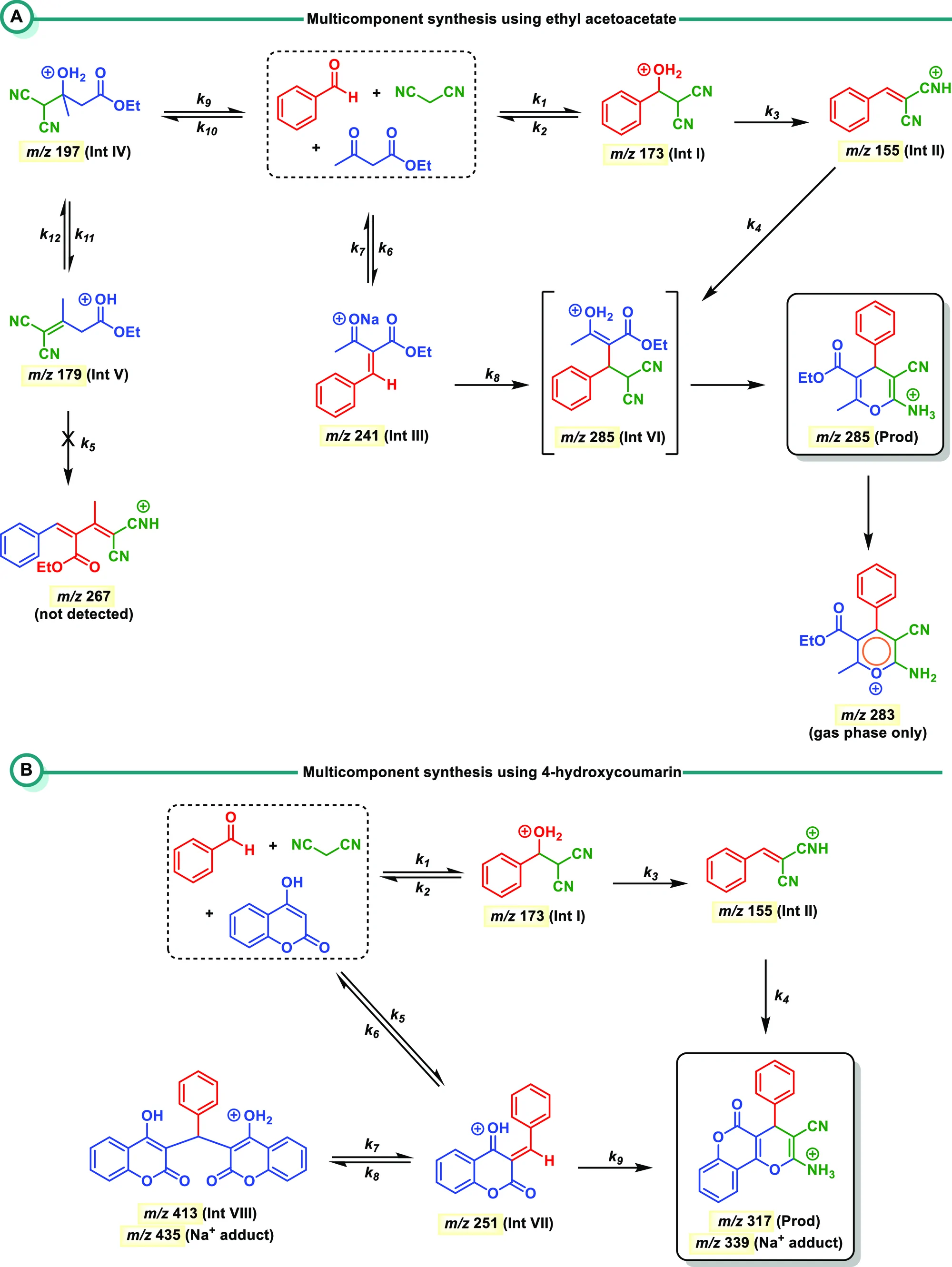
Proposed Kinetic Models Based on ESI–MS^a^

a Note that the use of ethyl acetoacetate (A) afforded kinetics completely different from those obtained when 4-hydroxycoumarin (B) is used.

A set of differential mass balance equations for all reaction components is proposed, based on the kinetic mechanism (Scheme 6A), to predict the dynamic behavior of the main reactive species in the reaction medium, as given by eqs 1–7. According to eqs 1–7, *y*_*i*_ is related to the normalized intensity of component *i* from MS experiments. The subscript *i*, ranging from 1 to 7, denotes: (1) the combined contribution of benzaldehyde, malononitrile, and ethyl acetoacetate; (2) **Int I**; (3) **Int II**; (4) **Int III**; (5) **Int IV**; (6) **Int V**; and (7) the product of the multicomponent reaction. *k*_*i*_ stands for the kinetic constants, with *i* ranging from 1 to 12.

1 $$\frac{dy_{1}}{dt}=-\left(k_{1}+k_{6}+k_{10}\right)y_{1}+k_{2}y_{2}+k_{7}y_{4}+k_{9}y_{5}$$

2 $$\frac{dy_{2}}{dt}=-\left(k_{2}+k_{3}\right)y_{2}+k_{1}y_{1}$$

3 $$\frac{dy_{3}}{dt}=-k_{4}y_{3}+k_{3}y_{2}$$

4 $$\frac{dy_{4}}{dt}=-\left(k_{7}+k_{8}\right)y_{4}+k_{6}y_{1}$$

5 $$\frac{dy_{5}}{dt}=-\left(k_{9}+k_{11}\right)y_{5}+k_{10}y_{1}+k_{12}y_{6}$$

6 $$\frac{dy_{6}}{dt}=-k_{12}y_{6}+k_{11}y_{5}$$

7 $$\frac{dy_{7}}{dt}=k_{4}y_{3}+k_{8}y_{4}$$

For the MCR of benzaldehyde, malononitrile, and 4-hydroxycoumarin, the complete set of model equations, in accordance with Scheme 6B, is expressed by eqs 8–13. In this case, the normalized intensity (*y*_*i*_) of the main reactive species *i* (with *i* ranging from 1 to 6) in the reaction medium represents: (1) the combined contribution of benzaldehyde, malononitrile, and 4-hydroxycoumarin; (2) **Int I**; (3) **Int II**; (4) Int **VII**; (5) **Int VIII**; and (6) the product of the multicomponent reaction. The kinetic constant is denoted by *k*_*i*_, with *i* ranging from 1 to 9.

8 $$\frac{dy_{1}}{dt}=-\left(k_{1}+k_{5}\right)y_{1}+k_{2}y_{2}+k_{6}y_{4}$$

9 $$\frac{dy_{2}}{dt}=-\left(k_{2}+k_{3}\right)y_{2}+k_{1}y_{1}$$

10 $$\frac{dy_{3}}{dt}=-k_{4}y_{3}+k_{3}y_{2}$$

11 $$\frac{dy_{4}}{dt}=-\left(k_{6}+k_{8}+k_{9}\right)y_{4}+k_{5}y_{1}+k_{7}y_{5}$$

12 $$\frac{dy_{5}}{dt}=-k_{7}y_{5}+k_{8}y_{4}$$

13 $$\frac{dy_{6}}{dt}=k_{4}y_{3}+k_{9}y_{4}$$

All kinetic constants (kinetic mechanism represented by eqs 1–7 in Scheme 6A, and eqs 8–13 in Scheme 6B) were determined through a sequential optimization procedure (details in the Experimental Section). Based on this methodology, all constants could be obtained, as expressed in Table 4.

**Table 4:** Estimated Kinetic Constants Based on Parameter Estimation Approach

	parameter value	
kinetic constant (min^–1^)	Scheme 6A	Scheme 6B
*k*_1_	0.5393	0.0553
*k*_2_	2.4794	1.7422
*k*_3_	0.2410	1.9539
*k*_4_	0.5604	1.9436
*k*_5_	-	0.0768
*k*_6_	0.0190	1.8723
*k*_7_	0.0000	0.0389
*k*_8_	0.0000	2.0500
*k*_9_	0.0500	0.0000
*k*_10_	0.0581	-
*k*_11_	0.0172	-
*k*_12_	0.4517	-

From Table 4, one can note that in Scheme 6A, the reaction of benzaldehyde with malononitrile occurs in equilibrium to form **Int I**, but the fitted rate constants indicate that the reverse process is substantially faster (*k*_2_ = 2.4794 min^–1^) than the forward step (*k*_1_ = 0.5393 min^–1^). This behavior implies limited steady-state accumulation of **Int I** and a strong tendency for the system to regenerate benzaldehyde and malononitrile. Under these conditions, productive conversion requires rapid consumption of **Int I** through subsequent steps rather than relying on its formation. Once **Int I** is formed, the kinetic constants support a productive sequence in which **Int I** proceeds to **Int II** (*k*_3_ = 0.2410 min^–1^), followed by formation of the final intermediate and, as a consequence, of the MCR adduct (*k*_4_ = 0.5604 min^–1^). Because *k*_4_ exceeds *k*_3_, conversion of **Int I** into **Int II** is slower within this productive sequence, whereas conversion of **Int II** into product is faster. Therefore, the overall flux to product in Scheme 6A is particularly sensitive to the rate at which **Int I** is driven forward toward **Int II**, competing against the rapid backward equilibrium that restores the starting materials. For the success of the reaction, the solvent is once more essential in further directing the reaction toward the final MCR adduct.

Alternative routes involving ethyl acetoacetate are not supported as productive channels under the fitted model. The direct equilibrium between benzaldehyde and ethyl acetoacetate leading to **Int III** is slow (*k*_6_ = 0.0190 min^–1^), and no detectable contribution from **Int III** to product formation is required (*k*_8_ = 0). In addition, the interaction of malononitrile with ethyl acetoacetate forms **Int IV** in a slow reversible process (*k*_9_ = 0.0500 min^–1^; *k*_10_ = 0.0581 min^–1^), while the subsequent interconversion between **Int IV** and **Int V** is strongly biased toward **Int IV** (*k*_11_ = 0.0172 min^–1^; *k*_12_ = 0.4517 min^–1^). These values indicate that this subnetwork mainly recycles back to precursors rather than feeding product formation.

In Scheme 6B (see also Table 4), the reaction of benzaldehyde with malononitrile is also reversible, but the equilibrium is markedly shifted toward the starting materials, as evidenced by a much smaller forward rate constant (*k*_1_ = 0.0553 min^–1^) relative to the reverse step (*k*_2_ = 1.7422 min^–1^). Despite this unfavorable equilibrium, once **Int I** is generated it is rapidly converted onward, since both **Int I** → **Int II** (*k*_3_ = 1.9539 min^–1^) and **Int II** → product (*k*_4_ = 1.9436 min^–1^) are fast and comparable in magnitude. These values indicate that, in Scheme 6B, the overall formation of product through this channel is expected to be limited primarily by the net formation of **Int I** rather than by downstream chemistry.

The kinetic scheme also includes equilibria involving 4-hydroxycoumarin, in which benzaldehyde reacts reversibly with this “persistent enol” to form **Int VII** (*k*_5_ = 0.0768 min^–1^; *k*_6_ = 1.8723 min^–1^), indicating a strongly reversible and weakly populated adduct. A second addition of 4-hydroxycoumarin to afford **Int VIII** is also strongly reversible (*k*_7_ = 0.0389 min^–1^; *k*_8_ = 2.0500 min^–1^), further disfavoring accumulation of higher adducts. Consistently, the fitted model does not support direct conversion of **Int VII** to product under these conditions (*k*_9_ = 0), indicating that this branch does not provide a productive pathway to the final adduct.

The overall net result is that the replacement of ethyl acetoacetate with 4-hydroxycoumarin shifts the kinetic profile from a system constrained by the forward consumption of **Int I** (Scheme 6A, where *k*_3_ is relatively small) to a system constrained by the net formation of **Int I** itself (Scheme 6B, where *k*_1_ is very small but *k*_3_ and *k*_4_ are large). In both cases, the kinetic parameters support product formation predominantly through the pathway initiated by the reversible formation of **Int I** from benzaldehyde and malononitrile, followed by rapid conversion to **Int II** and then to the final adduct.

Compounds bearing the **ACP** scaffold have also been reported to display relevant antimicrobial potential.^123,124^ In this context, Table 5 summarizes the minimum inhibitory concentration (MIC) and minimum bactericidal concentration (MBC) values obtained for compounds **4a**–**4x** against *Staphylococcus aureus* (ATCC 29213) and *Pseudomonas aeruginosa* (ATCC 27853), determined by the broth microdilution assay. For interpretation of the antimicrobial data, MIC values ≤100 μg mL^–1^ were classified as indicative of good activity, values between 100 and 500 μg mL^–1^ as moderate activity, values from 500 to 1000 μg mL^–1^ as weak activity, and values above 1000 μg mL^–1^ as inactive.^125^

**Table 5:** Evaluation of the Minimum Inhibitory Concentration and Minimum Bactericidal Concentration of Compounds **4a**–**4x** using the Broth Microdilution Method

	*S. aureus* (ATCC 29213)		*P. aeruginosa* (ATCC 27853)	
compound^a^ ^,^ ^b^	MIC (μg mL^–1^)	MBC (μg mL^–1^)	MIC (μg mL^–1^)	MBC (μg mL^–1^)
**4a**	62.5	>1000	250	>1000
**4b**	62.5	>1000	250	>1000
**4c**	62.5	>1000	62.5	>1000
**4d**	62.5	>1000	62.5	>1000
**4e**	125	1000	31.2	>1000
**4f**	125	1000	500	>1000
**4g**	1000	>1000	250	>1000
**4h**	62.5	>1000	1000	>1000
**4i**	250	>1000	500	>1000
**4j**	500	>1000	500	>1000
**4k**	500	>1000	125	>1000
**4l**	500	>1000	250	>1000
**4m**	500	>1000	1000	>1000
**4n**	1000	>1000	1000	>1000
**4o**	500	>1000	1000	>1000
**4p**	250	1000	>1000	>1000
**4q**	125	1000	1000	>1000
**4r**	1000	>1000	>1000	>1000
**4s**	1000	>1000	>1000	>1000
**4t**	250	>1000	>1000	>1000
**4u**	>1000	>1000	>1000	>1000
**4v**	>1000	>1000	>1000	>1000
**4w**	500	>1000	>1000	>1000
**4x**	250	>1000	>1000	>1000

a Compounds used as reference standards: chloramphenicol with MIC of 4 μg mL^–1^ and gentamicin with MIC of 0.5 μg mL^–1^.

b Compounds **4a**–**4x** were also evaluated by the broth microdilution assay against *Candida albicans* (ATCC 10231) and *Candida tropicalis* (ATCC 13803) and showed MIC and MFC values ≥1000 μg mL^–1^.

Based on these criteria, three compounds, namely **4c**, **4d**, and **4e**, exhibited good antibacterial activity against *P. aeruginosa*, a pathogen recognized for posing a significant risk to hospitalized patients. In parallel, compounds **4a**, **4b**, **4c**, **4d**, and **4h** displayed good activity against *S. aureus*. A moderate antibacterial profile was observed for 13 compounds against *S. aureus*, whereas eight derivatives showed moderate activity against *P. aeruginosa*. Weak antibacterial activity was identified for four compounds against *S. aureus* and for five compounds against *P. aeruginosa*. Complete lack of inhibitory activity was observed for compounds **4u** and **4v** against *S. aureus*, while eight compounds were inactive against *P. aeruginosa* under the tested conditions.

Previous studies have reported optically active pyranocoumarins and 2-amino-4*H*-chromene derivatives whose antibacterial activity was evaluated using the broth microdilution method.^126^ These heterocycles exhibited activity primarily against *S. aureus*, supporting the notion that this structural class represents a promising platform for the design of new antibacterial agents. A higher number of inactive compounds was observed against *P. aeruginosa* when compared with *S. aureus*. This behavior is consistent with the Gram-negative nature of *P. aeruginosa*, whose outer membrane presents increased chemical complexity and acts as an efficient permeability barrier, thereby conferring enhanced resistance to antibacterial agents that are unable to effectively traverse this structure.^127^

Overall, most of the evaluated compounds exhibited a predominantly bacteriostatic profile, characterized by inhibition of bacterial growth. A pronounced bactericidal effect was not observed at the concentrations investigated, as reflected by the generally high MBC values.

## Conclusions

A comprehensive mechanistic and solvent-effect investigation of the multicomponent synthesis of **ACP** derivatives has been presented. By integrating solvent descriptor analysis, control experiments, and reaction productivity as an oriented metric, this study demonstrates that reaction efficiency in this system is governed by a delicate interplay between reversible equilibria and solvent-controlled pathway selection rather than by isolated kinetic steps. The results establish that product formation proceeds predominantly through a pathway initiated by the reversible condensation between benzaldehyde and malononitrile, followed by enol-mediated bond formation, while alternative routes involving direct participation of the 1,3-dicarbonyl component are kinetically disfavored.

Solvent effects were shown to play distinct and stage-dependent roles: hydrogen-bond basicity (β) modulates carbonyl reactivity and Knoevenagel efficiency, hydrogen-bond acidity (α) promotes conversion of reactive intermediates into the final adduct, and dipolarity/polarizability (π*) influences reaction productivity by stabilizing polarized intermediates. The combined influence of these parameters explains the superior performance of ethanol, which balances enol availability, intermediate stabilization, and overall reaction driving force. The catalytic system PEI.IL further modulates the keto–enol equilibrium and enables efficient catalyst recycling without loss of activity.

Beyond providing an efficient and sustainable synthetic protocol, this work highlights the importance of experimentally validated mechanistic analysis in MCRs and illustrates how reaction productivity can serve as a powerful descriptor to rationalize solvent and equilibrium effects in complex reaction networks. These findings offer general insights applicable to the rational design and optimization of multicomponent transformations well beyond the specific **ACP** framework studied herein.

## Experimental Section

### General Information

Liquid reagents and solvents were distilled before use, and solid reagents were crystallized to ensure purity. Vibrational absorption spectroscopy in the infrared region (FT-IR) was performed using KBr pellets, with spectra recorded in the 4000–400 cm^–1^ range. ^1^H and ^13^C­{^1^H} nuclear magnetic resonance (NMR) spectra were recorded using standard instrumentation with 5 mm probes. Measurements were conducted at room temperature and at 45 °C. The operating frequencies were 400 MHz (or 500, or 600 MHz) for ^1^H NMR and 100 MHz (or 125, or 150 MHz) for ^13^C­{^1^H} NMR. Chemical shifts (δ) were referenced to deuterated chloroform (CDCl_3_) or deuterated dimethyl sulfoxide (DMSO-*d*_6_), using tetramethylsilane (TMS) as the internal standard. NMR multiplicities are reported as s (singlet), d (doublet), dd (doublet of doublets), t (triplet), q (quartet), quin (quintet), and m (multiplet). High-resolution mass spectra (HRMS) were obtained by electrospray ionization (ESI). ESI–MS­(/MS) analyses were performed in the positive ion mode over an *m*/*z* range of 50–1000 Da. Ions were detected as protonated molecules and/or sodiated adducts. Melting points were determined using a digital melting point apparatus. Reactions that required heating were carried out using a silicone oil bath.

### Kinetics

All kinetic constants (kinetic mechanism represented by eqs 1–7 in Scheme 6A, and eqs 8–13 in Scheme 6B) were determined through a sequential optimization procedure. For this purpose, a numerical approach was implemented in Python, combining the heuristic particle swarm optimization (PSO) algorithm with an ordinary differential equations solver based on the Adams/BDF multistep method.^128–131^ This sequential strategy allows efficient parameter estimation, accurately describing the reaction dynamics. For a comprehensive discussion of the numerical procedures used in parameter estimation and in solving the set of ordinary differential equations, readers are referred to a previously described work.^82^

### Antimicrobial Activity Evaluation

Standard bacterial strains obtained from the American Type Culture Collection (ATCC) were used, namely *P. aeruginosa* (ATCC 27853), *S. aureus* (ATCC 29213), *Candida albicans* (ATCC 10231) and *Candida tropicalis* (ATCC 13803). Bacterial inocula were prepared by suspending colonies grown on agar plates after 24 h of incubation at 37 °C in sterile saline solution (0.9% NaCl). Turbidity was adjusted to the 0.5 McFarland standard, corresponding to approximately 1–2 × 10^8^ colony-forming units (CFU) mL^–1^. Minimum inhibitory concentrations (MICs) were determined by dissolving the synthesized compounds in 5% DMSO and diluting them in Mueller–Hinton broth to final concentrations ranging from 1000 to 15.6 μg mL^–1^. Each well of the microplate was inoculated with 7 μL of the bacterial suspension. All assays were performed in triplicate and included controls for bacterial growth, solvent (DMSO), and medium sterility. Chloramphenicol (64–2 μg mL^–1^) and gentamicin (8–0.5 μg mL^–1^) were used as reference antibiotics. The microplates were incubated at 35 °C for 24 h, after which MIC values were determined by visual inspection and confirmed using a 0.01% resazurin solution. The MIC was defined as the lowest concentration of the compounds (**4a**–**x**) that completely inhibited visible bacterial growth. Minimum bactericidal concentrations (MBCs) were determined by subculturing 100 μL aliquots from wells showing no visible growth onto nutrient agar plates using a Drigalski loop. The plates were incubated at 36 °C for 24 h, and the MBC was defined as the lowest concentration resulting in no bacterial growth. All MBC determinations were performed in duplicate.

### General Procedure for the Synthesis of Intermediate 2-Benzylidenemalononitrile (Int II)

Malononitrile (1 mmol, 66 mg), benzaldehyde (1 mmol, 101.1 μL), PEI.IL (100 mg), and EtOH (1 mL) were added to a sealed tube. The reaction mixture was stirred at 60 °C for 20 min. After completion, the crude product was purified by flash column chromatography using ethyl acetate/hexane (2:8) as the mobile phase. A white solid was obtained in 52% yield (111.5 mg).

^1^H NMR (400 MHz, CDCl_3_): δ 7.94–7.87 (m, 2H), 7.78 (s, 1H), 7.69–7.60 (m, 1H), 7.60–7.50 (m, 2H) ppm. ^13^C­{^1^H} NMR (100 MHz, CDCl_3_): δ 159.9, 134.6, 130.9, 130.7, 129.9, 113.7, 112.5, 82.8 ppm.

### General Procedure for the Synthesis of Intermediate Ethyl Acetobenzylidene Acetate (Int IV)

Ethyl acetoacetate (1 mmol, 126.4 μL), benzaldehyde (1 mmol, 101.1 μL), PEI.IL (100 mg), and EtOH (1 mL) were added to a sealed tube. The reaction mixture was stirred at 60 °C for 20 min. After completion, purification was performed by flash column chromatography using ethyl acetate/hexane (1:9) as the mobile phase. A yellow oil was obtained as a mixture of isomers: (*Z*)-ethyl acetobenzylidene acetate (52% yield, 112 mg) and (*E*)-ethyl acetobenzylidene acetate (7% yield, 15 mg).

#### (*Z*)-Ethyl Acetobenzylidene Acetate

^1^H NMR (400 MHz, CDCl_3_): δ 7.50 (s, 1H), 7.42–7.35 (m, 2H), 7.34–7.29 (m, 3H), 4.26 (q, *J* = 7.2 Hz, 2H), 2.35 (s, 3H), 1.20 (t, *J* = 7.2 Hz, 3H) ppm. ^13^C­{^1^H} NMR (100 MHz, CDCl_3_): δ 194.6, 167.8, 141.2, 134.6, 133.0, 130.7, 129.5, 128.8, 61.7, 26.5, 13.8 ppm. These values attributed to this isomer are in accordance with the previous literature.^132^

#### (*E*)-Ethyl Acetobenzylidene Acetate

^1^H NMR (400 MHz, CDCl_3_): δ 7.59 (s, 1H), 7.49–7.46 (m, 2H), 7.44–7.39 (m, 3H), 4.35 (q, *J* = 6.9 Hz, 2H), 2.45 (s, 3H), 1.29 (t, *J* = 7.1 Hz, 3H). ^13^C­{^1^H} NMR (100 MHz, CDCl_3_): δ 203.4, 164.4, 140.5, 134.1, 133.0, 130.4, 129.7, 129.0, 61.6, 31.2, 14.1 ppm. These values attributed to this isomer are in accordance with the previous literature.^132^

### General Procedure for the Synthesis of Intermediate Ethyl 4,4-Dicyano-3-methyl-2-butenoate (Int V)

Ethyl acetoacetate (1 mmol, 126.4 μL), malononitrile (3 mmol, 198 mg), PEI.IL (100 mg), and EtOH (1 mL) were added to a sealed vial. The reaction mixture was stirred at 60 °C for 20 min. After completion, purification was carried out by flash column chromatography using ethyl acetate/hexane (2:9) as the mobile phase. A white oil was obtained in 61% yield (109 mg).

^1^H NMR (400 MHz, CDCl_3_): δ 4.22 (q, *J* = 7.1 Hz, 2H), 3.60 (s, 2H), 2.37 (s, 3H), 1.30 (t, *J* = 7.1 Hz, 3H) ppm. ^13^C­{^1^H} NMR (100 MHz, CDCl_3_): δ 172.5, 166.3, 111.2, 111.1, 89.3, 62.3, 42.4, 23.2, 14.0 ppm.

### General Procedure for the Synthesis of 2-Amino-3-cyano-4*H*-pyran Derivatives

In a round-bottom flask equipped with a reflux condenser, EtOH (1 mL), an aromatic or heteroaromatic aldehyde (**1a–i**, 1 mmol), malononitrile (**2**, 3 mmol, 198 mg), a 1,3-dicarbonyl compound (ethyl acetoacetate (**3a**), 5,5-dimethyl-1,3-cyclohexadione (**3b**), or 4-hydroxycoumarin (**3c**), (1 mmol), and PEI.IL (100 mg) were combined. The reaction mixture was heated at 60 °C for 20 min. After completion, the products were purified by recrystallization from EtOH.

### Scale-Up (Multigram) Experiment

Benzaldehyde (15 mmol, 1.57 mL), malononitrile (45 mmol, 2.97 g), ethyl acetoacetate (15 mmol, 1.89 mL), PEI.IL (1.5 g) and 15 mL of EtOH were placed in a sealed flask. The reaction mixture was kept at a temperature of 60 °C under constant stirring for 20 min. At the end of the reaction period, a mixture of H_2_O/EtOH was added to the crude reaction mixture, causing the product of interest to precipitate as a yellow solid. This was then collected by vacuum filtration. Subsequently, this solid was dried at reduced pressure. In this perspective, product **4a** was obtained in the form of a yellow solid with a 90% yield (3.83 g, 13.45 mmol).

### Recycling of the PEI.IL Catalyst

After the synthesis of the **ACP 4a–4x** derivatives, the reusability of the ionically tagged catalyst used in the reaction medium was evaluated. For this study, the **ACP 4n** derivative was selected as the model substrate. Thus, in a round-bottom flask, 1 mmol of the aldehyde, 3 mmol of malononitrile, 1 mmol of dimedone, 1 mL of EtOH, and 100 mg of PEI.IL were mixed and stirred at 60 °C for 20 min. The product (solid) was isolated by vacuum filtration and washed with ethanol. After filtration, the mother liquor was rotary-evaporated to remove the washing solvent, and a new reaction was carried out (see Figure S2).

#### Ethyl 6-Amino-5-cyano-2-methyl-4-phenyl-4*H*-pyran-3-carboxylate (**4a**)

The product was obtained as a yellow solid (0.97 mmol, 276 mg, 97%); mp 197–199 °C; FT-IR (KBr, cm^–1^): 3404, 3328, 3216, 3032, 2988, 2190, 1739, 1684, 1653, 1607, 1253, 845, 822. ^1^H NMR (CDCl_3_, 500 MHz): δ 7.32–7.27 (m, 2H), 7.24–7.16 (m, 3H), 4.50–4.41 (m, 3H), 4.08–3.96 (m, 2H), 2.37 (d, *J* = 1.1 Hz, 3H), 1.09 (t, *J* = 7.1 Hz, 3H) ppm. ^13^C­{^1^H} NMR (CDCl_3_, 125 MHz): δ 165.8, 157.4, 156.8, 143.7, 128.6, 127.5, 127.2, 118.9, 108.0, 62.5, 60.6, 38.7, 18.4, 13.8 ppm. HRMS (ESI) *m*/*z*: [M + H]^+^, 285.1234 calcd for C_16_H_16_N_2_O_3_; found, 285.1240; [M + Na]^+^, 307.1053 calcd; found, 307.1063.

#### Ethyl 6-Amino-5-cyano-2-methyl-4-(3-nitrophenyl)-4*H*-pyran-3-carboxylate (**4b**)

The product was obtained as a yellow solid (0.75 mmol, 246 mg, 75%). mp 166–168 °C; FT-IR (KBr, cm^–1^): 3397, 3325, 3220, 2987, 2195, 1686, 1666, 1644, 1601, 1531, 1341, 1368, 1270, 1178, 789.; ^1^H NMR (CDCl_3_, 500 MHz): δ 8.12 (dt, *J* = 6.9 and 2.3 Hz, 1H), 7.98 (t, *J* = 1.6 Hz, 1H), 7.70–7.62 (m, 2H), 7.10 (s, 2H), 4.53 (d, *J* = 1.2 Hz, 1H), 4.02–3.95 (m, 2H), 2.35 (d, *J* = 1.0 Hz, 3H), 1.03 (t, *J* = 7.1 Hz, 3H) ppm. ^13^C {^1^H} NMR (DMSO-*d*_6_, 125 MHz): δ 165.6, 159.1, 158.3, 148.2, 147.84, 134.7, 130.7, 122.4, 122.2, 119.9, 106.7, 60.8, 56.7, 39.0, 18.8, 14.1 ppm. HRMS (ESI) *m*/*z*: [M + H]^+^, 330.1084 calcd for C_16_H_15_N_3_O_5_; found, 330.1079, [M + Na]^+^ 352.0904 calcd; found, 352.0898.

#### Ethyl 6-Amino-5-cyano-2-methyl-4-(2-nitrophenyl)-4*H*-pyran-3-carboxylate (**4c**)

The product was obtained as a yellow solid (0.94 mmol, 309 mg, 94%); mp 143–145 °C; FT-IR (KBr, cm^–1^): 3453, 3325, 3205, 2973, 2209, 1729, 1679, 1648, 1602, 1531, 1348, 1221, 1171, 775. ^1^H NMR (CDCl_3_, 500 MHz): δ 7.81 (dd, *J* = 8.4 and 1.3 Hz, 1H), 7.56 (td, *J* = 7.5 and 1.4 Hz, 1H), 7.40–7.32 (m, 2H), 5.26 (d, *J* = 1.1 Hz, 1H), 4.62 (s, 2H), 3.99–3.92 (m, 2H), 2.40 (d, *J* = 1.1 Hz, 3H), 0.98 (t, *J* = 7.1 Hz, 3H) ppm. ^13^C {^1^H} NMR (CDCl_3_, 125 MHz): δ 165.0, 158.2, 158.0, 149.1, 139.0, 133.2, 130.6, 127.9, 124.1, 118.1, 107.3, 61.2, 60.9, 33.0, 18.4, 13.6 ppm. HRMS (ESI) *m*/*z*: [M + H]^+^, 330.1084 calcd for C_16_H_15_N_3_O_5_; found, 330.1078, [M + Na]^+^, 352.0904 calcd; found, 352.0889.

#### Ethyl 6-Amino-5-cyano-2-methyl-4-(4-nitrophenyl)-4*H*-pyran-3-carboxylate (**4d**)

The product was obtained as an orange solid (0.75 mmol, 246 mg, 75%); mp 144–145 °C; FT-IR (KBr, cm^–1^): 3410, 3333, 3198, 2980, 2195, 1686, 1679, 1644, 1606, 1510, 1348, 1270, 1171, 818. ^1^H NMR (CDCl_3_, 500 MHz): δ 8.22–8.14 (m, 2H), 7.42–7.34 (m, 2H), 4.62 (s, 2H), 4.56 (d, *J* = 1.2 Hz, 1H), 4.07–4.01 (m, 2H), 2.42 (d, *J* = 1.0 Hz, 3H), 1.10 (t, *J* = 7.1 Hz, 3H) ppm. ^13^C {^1^H} NMR (CDCl_3_, 125 MHz): δ 165.2, 158.0, 157.6, 151.0, 147.0, 128.4, 124.0, 118.3, 106.7, 61.0, 60.9, 38.7, 18.6, 13.9 ppm. HRMS (ESI) *m*/*z*: [M + H]^+^, 330.1084 calcd for C_16_H_15_N_3_O_5_; found, 330.1081, [M + Na]^+^, 352.0904 calcd; found, 352.0906.

#### Ethyl 6-Amino-4-(4-chlorophenyl)-5-cyano-2-methyl-4*H*-pyran-3-carboxylate (**4e**)

The product was obtained as an orange solid (0.83 mmol, 264 mg, 83%); mp 146–147 °C; FT-IR (KBr, cm^–1^): 3347, 3333, 2987, 2209, 1708, 1651, 1558, 1496, 1404, 1263, 1185, 1086, 839, 507. ^1^H NMR (DMSO-*d*_6_, 500 MHz): δ 7.40–7.35 (m, 2H), 7.19–7.14 (m, 2H), 6.94 (s, 2H), 4.31 (d, *J* = 1.2 Hz, 1H), 4.04–3.90 (m, 2H), 2.31 (d, *J* = 1.0 Hz, 3H), 1.04 (t, *J* = 7.1 Hz, 3H) ppm. ^13^C {^1^H} NMR (DMSO-*d*_6_, 125 MHz): δ 165.8, 158.9, 157.4, 144.4, 131.8, 129.6, 129.4, 128.9, 120.0, 107.2, 60.7, 38.7, 18.7, 14.2 ppm. HRMS (ESI) *m*/*z*: [M + H]^+^, 319.0844 calcd for C_16_H_15_ClN_2_O_3_; found, 319.0844, [M + Na]^+^, 341.0663 calcd; found, 341.0659.

#### Ethyl 6-Amino-5-cyano-2-methyl-4-(4-methylphenyl)-4*H*-pyran-3-carboxylate (**4f**)

The product was obtained as an orange solid (0.67 mmol, 199 mg, 67%); mp 164–165 °C; FT-IR (KBr, cm^–1^): 3417, 3334, 3234, 2980, 2209, 1708, 1657, 1609, 1581, 1510, 1404, 1248, 1178, 853. ^1^H NMR (CDCl_3_, 500 MHz): δ 7.37–7.33 (m, 2H), 7.26 (d, *J* = 8.0 Hz, 4H), 7.14 (s, 1H), 6.09 (dd, *J* = 2.6 and 1.0 Hz, 1H), 5.56 (s, 1H), 4.19–4.10 (m, 2H), 2.39 (s, 2H), 1.28 (t, *J* = 7.1 Hz, 3H) ppm. ^13^C {^1^H} NMR (CDCl_3_, 125 MHz): δ 166.0, 147.5, 143.6, 140.1, 131.2, 130.0, 128.4, 128.2, 114.0, 111.4, 110.3, 60.3, 45.9, 27.7, 21.2, 14.2 ppm of HRMS (ESI) *m*/*z*: [M + H]^+^, 299.1390 calcd for C_17_H_18_N_2_O_3_; found, 299.1386, [M + Na]^+^, 321.1210 calcd; found, 321.1213.

#### 2-Amino-7,7-dimethyl-5-oxo-4-phenyl-5,6,7,8-tetrahydro-4*H*-chromene-3-carbonitrile (**4g**)

The product was obtained as a yellow solid (0.59 mmol, 174 mg, 59%); mp 234–236 °C; FT-IR (KBr, cm^–1^): 3396, 3325, 3213, 2958, 2880, 2195, 1686, 1659, 1602, 1418, 1368, 1248, 1213, 698. ^1^H NMR (DMSO-*d*_6_, 500 MHz): δ 7.28 (t, *J* = 7.6 Hz, 2H), 7.21–7.11 (m, 3H), 7.00 (s, 2H), 4.17 (s, 1H), 2.57–2.51 (m, 2H), 2.25 (d, *J* = 16.0 Hz, 1H), 2.10 (d, *J* = 16.0 Hz, 1H), 1.04 (s, 3H), 0.95 (s, 3H) ppm. ^13^C {^1^H} NMR (DMSO-*d*_6_, 125 MHz): δ 196.2, 163.0, 158.9, 145.2, 128.8, 127.6, 127.0, 120.2, 113.1, 58.7, 50.4, 36.1, 36.0, 32.3, 28.9, 28.8, 27.3, 27.2 ppm. HRMS (ESI) *m*/*z*: [M + H]^+^, 295.1441 calcd for C_18_H_18_N_2_O_2_ found 295.1438, [M + Na]^+^, 317.1260 calcd; found, 317.1252.

#### 2-Amino-4-(4-hydroxy-3-methoxyphenyl)-7,7-dimethyl-5-oxo-5,6,7,8-tetrahydro-4*H*-chromene-3-carbonitrile (**4h**)

The product was obtained as a yellow solid (0.91 mmol, 310 mg, 91%); mp 218–219 °C; FT-IR (KBr, cm^–1^): 3496–3220, 3403, 3325, 3018, 2965, 2874, 2188, 1679, 1651, 1588, 1503, 1429, 1206, 1036, 867, 648. ^1^H NMR (DMSO-*d*_6_, 500 MHz): δ 8.84 (s, 1H), 6.93 (s, 2H), 6.69 (d, *J* = 8.1 Hz, 1H), 6.66 (d, *J* = 2.1 Hz, 1H), 6.53 (dd, *J* = 8.1 and 2.1 Hz, 1H), 4.09 (d, *J* = 1.3 Hz, 1H), 3.72 (s, 3H), 2.58–2.44 (m, 2H), 2.27 (d, *J* = 16.1 Hz, 1H), 2.12 (dd, *J* = 16.0 and 1.1 Hz, 1H), 1.05 (s, 3H), 0.98 (s, 3H) ppm. ^13^C {^1^H} NMR (DMSO-*d*_6_, 125 MHz): δ 196.2, 162.6, 158.8, 147.7, 145.7, 136.30, 120.3, 119.8, 115.8, 113.5, 111.9, 59.2, 56.0, 50.5, 35.4, 32.2, 29.0, 27.0 ppm. HRMS (ESI) *m*/*z*: [M + H]^+^, 341.1496 calcd for C_19_H_20_N_2_O_4_; found, 341.1491, [M + Na]^+^, 363.1315 calcd; found, 363.1321.

#### 2-Amino-7,7-dimethyl-4-(2-nitrophenyl)-5-oxo-5,6,7,8-tetrahydro-4*H*-chromene-3-carbonitrile (**4i**)

The product was obtained as a brown solid (0.86 mmol, 292 mg, 86%); mp 209–210 °C; FT-IR (KBr, cm^–1^): 3404, 3333, 3206, 2965, 2871, 2188, 1694, 1666, 1595, 1524, 1357, 1256, 1143. ^1^H NMR (DMSO-*d*_6_, 500 MHz): δ 7.81 (dd, *J* = 8.1 and 1.3 Hz, 1H), 7.66 (td, *J* = 7.6 and 1.3 Hz, 1H), 7.48–7.39 (m, 1H), 7.35 (dd, *J* = 7.9 and 1.4 Hz, 1H), 7.17 (s, 2H), 4.94 (d, *J* = 1.4 Hz, 1H), 2.57–2.41 (m, 2H), 2.20 (d, *J* = 16.2 Hz, 1H), 2.01 (dd, *J* = 16.0 and 1.1 Hz, 1H), 1.01 (s, 3H), 0.88 (s, 3H) ppm. ^13^C­{^1^H} NMR (DMSO-*d*_6_, 125 MHz): δ 196.3, 163.2, 159.7, 149.4, 139.4, 133.8, 130.7, 128.3, 124.2, 119.5, 112.8, 56.9, 50.0, 32.3, 30.4, 28.8, 27.2 ppm. HRMS (ESI) *m*/*z*: [M + H]^+^, 340.1292 calcd for C_18_H_17_N_3_O_4_; found 340.1288, [M + Na]^+^, 362.1111 calcd; found, 362.1109.

#### 2-Amino-7,7-dimethyl-4-(3-nitrophenyl)-5-oxo-5,6,7,8-tetrahydro-4*H*-chromene-3-carbonitrile (**4j**)

The product was obtained as a yellow solid (0.88 mmol, 299 mg, 88%); mp 209–210 °C; FT-IR (KBr, cm^–1^): 3424, 3333, 3205, 2958, 2874, 2195, 1686, 1659, 1595, 1411, 1524, 1354, 1376, 1270, 1171, 825. ^1^H NMR (DMSO-*d*_6_, 500 MHz): δ 8.13–8.04 (m, 1H), 7.98 (t, *J* = 2.0 Hz, 1H), 7.71–7.57 (m, 2H), 7.19 (s, 2H), 4.42 (s, 1H), 2.55 (d, *J* = 2.0 Hz, 2H), 2.27 (d, *J* = 16.1 Hz, 1H), 2.11 (d, *J* = 16.1 Hz, 1H), 1.04 (s, 3H), 0.95 (s, 3H) ppm. ^13^C­{^1^H} NMR (DMSO-*d*_6_, 125 MHz): δ 196.3, 163.6, 159.1, 148.2, 147.5, 134.7, 130.5, 122.3, 122.1, 119.8, 112.2, 57.6, 50.3, 35.9, 32.3, 28.8, 27.2 ppm. HRMS (ESI) *m*/*z*: [M + H]^+^, 340.1297 calcd for C_18_H_17_N_3_O_4_; found, 340.1285, [M + Na]^+^, 362.1111 calcd; found, 362.1107.

#### 2-Amino-7,7-dimethyl-4-(4-nitrophenyl)-5-oxo-5,6,7,8-tetrahydro-4*H*-chromene-3-carbonitrile (**4k**)

The product was obtained as a yellow solid (0.87 mmol, 295 mg, 87%); mp 178–179 °C; FT-IR (KBr, cm^–1^): 3446, 3368, 3213, 2958, 2874, 2195, 1686, 1667, 1591, 1517, 1469, 1341, 1256, 1206, 825. ^1^H NMR (DMSO-*d*_6_, 500 MHz): δ 8.17 (d, *J* = 8.4 Hz, 2H), 7.44 (d, *J* = 8.6 Hz, 2H), 7.18 (s, 2H), 4.37 (s, 1H), 2.54 (d, *J* = 3.7 Hz, 2H), 2.26 (d, *J* = 16.1 Hz, 1H), 2.11 (d, *J* = 16.1 Hz, 1H), 1.04 (s, 3H), 0.96 (s, 3H) ppm. ^13^C {^1^H} NMR (DMSO-*d*_6_, 125 MHz): δ 196.2, 163.6, 159.0, 152.8, 146.7, 129.1, 124.2, 119.8, 112.2, 57.4, 50.3, 36.1, 32.3, 28.7, 27.4 ppm. HRMS (ESI) *m*/*z*: [M + H]^+^, 340.1297 calcd for C_18_H_17_N_3_O_4_; found, 340.1288, [M + Na]^+^, 362.1111 calcd; found, 362.1109.

#### 2-Amino-7,7-dimethyl-5-oxo-4-(*p*-tolyl)-5,6,7,8-tetrahydro-4*H*-chromene-3-carbonitrile (**4l**)

The product was obtained as a yellow solid (0.94 mmol, 290 mg, 94%); mp 218–219 °C; FT-IR (KBr, cm^–1^): 3425, 3340, 3234, 2965, 2919, 2188, 1672, 1634, 1602, 1510, 1361, 1256, 1199, 839. ^1^H NMR (DMSO-*d*_6_, 500 MHz): δ 7.09 (d, *J* = 7.7 Hz, 2H), 7.02 (d, *J* = 7.6 Hz, 2H), 6.97 (s, 2H), 4.13 (s, 1H), 2.55–2.47 (overlapped with DMSO-*d*_6_, 2H), 2.26–2.23 (m, 4H), 2.09 (d, *J* = 16.1 Hz, 1H), 1.04 (s, 3H), 0.95 (s, 3H) ppm. ^13^C {^1^H} NMR (DMSO-*d*_6_, 125 MHz): δ 196.2, 162.8, 158.9, 142.3, 136.1, 129.4, 127.5, 120.3, 113.3, 58.8, 50.4, 35.7, 35.6, 32.3, 28.9, 27.2, 21.1 ppm. HRMS (ESI) *m*/*z*: [M + H]^+^, 309.1598 calcd for C_19_H_20_N_2_O_2_; found, 309.1596, [M + Na]^+^, 331.1417 calcd; found, 331.1412.

#### 2-Amino-7,7-dimethyl-4-(4-methoxyphenyl)-5-oxo-5,6,7,8-tetrahydro-4*H*-chromene-3-carbonitrile (**4m**)

The product was obtained as a yellow solid (0.93 mmol, 302 mg, 93%); mp 200–201 °C; FT-IR (KBr, cm^–1^): 3368, 3329, 3185, 2965, 2917, 2195, 1686, 1651, 1602, 1510, 1368, 1248, 1143, 845. ^1^H NMR (DMSO-*d*_6_, 500 MHz): δ 7.10–7.02 (m, 2H), 6.97 (s, 2H), 6.90–6.78 (m, 2H), 4.12 (d, *J* = 1.3 Hz, 1H), 3.72 (s, 3H), 2.52–2.50 (overlap with DMSO-*d*_6_, 2H), 2.25 (d, *J* = 16.1 Hz, 1H), 2.10 (dd, *J* = 16.0, 1.0 Hz, 1H), 1.04 (s, 3H), 0.95 (s, 3H) ppm. ^13^C­{^1^H} NMR (DMSO-*d*_6_, 125 MHz): δ 196.1, 162.6, 158.9, 158.4, 137.3, 128.7, 120.3, 114.1, 113.4, 59.0, 55.5, 50.5, 35.2, 32.3, 29.0, 27.2 ppm. HRMS (ESI) *m*/*z*: [M + H]^+^, 325.1547 calcd for C_19_H_20_N_2_O_3_; found, 325.1542; [M + Na]^+^, 347.1366 calcd; found, 347.1359.

#### 2-Amino-7,7-dimethyl-4-(4-chlorophenyl)-5-oxo-5,6,7,8-tetrahydro-4*H*-chromene-3- carbonitrile (**4n**)

The product was obtained as a yellow solid (0.99 mmol, 326 mg, 99%); mp 212–213 °C; FT-IR (KBr, cm^–1^): 3383, 3315, 3192, 2951, 2181, 1672, 1644, 1602, 1481, 1354, 1213, 1143, 860. ^1^H NMR (DMSO-*d*_6_, 500 MHz): δ 7.37–7.32 (m, 2H), 7.20–7.13 (m, 2H), 7.07 (s, 2H), 4.19 (d, *J* = 1.2 Hz, 1H), 2.56–2.50 (m, 2H), 2.25 (d, *J* = 16.0 Hz, 1H), 2.17–2.04 (m, 1H), 1.03 (s, 3H), 0.95 (s, 3H). ^13^C {^1^H} NMR (DMSO-*d*_6_, 125 MHz): δ 196.2, 163.1, 159.0, 144.2, 131.6, 129.6, 128.8, 120.0, 112.8, 58.2, 50.4, 35.6, 32.3, 28.8, 27.3 ppm. HRMS (ESI) *m*/*z*: [M + H]^+^, 329.1051 calcd for C_18_H_17_ClN_2_O_2_; found, 329.1050, [M + Na]^+^, 351.0871 calcd; found, 351.0867.

#### 2-Amino-7,7-dimethyl-4-(furan-2-yl)-5-oxo-5,6,7,8-tetrahydro-4*H*-chromene-3- carbonitrile (**4o**)

The product was obtained as a yellow solid (0.94 mmol, 267 mg, 94%); mp 230–231 °C; FT-IR (KBr, cm^–1^): 3403, 3325, 3220, 2973, 2924, 2195, 1672, 1659, 1602, 1348, 1224, 1136, 718. ^1^H NMR (DMSO-*d*_6_, 500 MHz): δ 7.48 (dd, *J* = 1.8 and 0.9 Hz, 1H), 7.08 (s, 2H), 6.32 (dd, *J* = 3.2 and 1.8 Hz, 1H), 6.05 (dd, *J* = 3.3 and 0.9 Hz, 1H), 4.32 (d, *J* = 1.3 Hz, 1H), 2.52 (dd, *J* = 17.7 and 1.6 Hz, 1H), 2.44 (d, *J* = 17.7 Hz, 1H), 2.28 (d, *J* = 16.1 Hz, 1H), 2.17 (dd, *J* = 16.1 and 1.2 Hz, 1H), 1.04 (s, 3H), 0.98 (s, 3H) ppm. ^13^C {^1^H} NMR (DMSO-*d*_6_, 125 MHz): δ 195.9, 163.7, 159.8, 156.2, 142.2, 120.0, 110.9, 110.8, 105.5, 55.9, 50.4, 40.2, 32.3, 29.4, 28.9, 27.0 ppm. HRMS (ESI) *m*/*z*: [M + H]^+^, 285.1234 calcd for C_16_H_16_N_2_O_3_; found, 285.1226 [M + Na]^+^, 307.1053 calcd; found, 307.1055.

#### 2-Amino-5-oxo-4-phenyl-4,5-dihydropyrano­[3,2-*c*]­chromene-3-carbonitrile (**4p**)

The product was obtained as a yellow solid (0.89 mmol, 281 mg, 89%); mp 258–261 °C; FT-IR (KBr, cm^–1^): 3379, 3280, 3187, 2201, 1716, 1668, 1644, 1601, 1487, 1209, 1166, 758. ^1^H NMR (DMSO-*d*_6_, 500 MHz): δ 7.91 (dd, *J* = 7.9 and 1.6 Hz, 1H), 7.75–7.66 (m, 1H), 7.54–7.44 (m, 2H), 7.38 (s, 2H), 7.35–7.29 (m, 2H), 7.29–7.20 (m, 3H), 4.45 (s, 1H) ppm. ^13^C {^1^H} NMR (DMSO-*d*_6_, 125 MHz): δ 160.0, 158.5, 153.9, 152.6, 143.8, 133.4, 129.0, 128.1, 127.6, 125.2, 123.0, 119.7, 117.0, 113.4, 104.5, 58.5, 37.4 ppm. HRMS (ESI) *m*/*z*: [M + Na]^+^, 339.0740 calcd for C_19_H_12_N_2_O_3_; found, 339.0738.

#### 2-Amino-4-(4-hydroxi-3-methoxyphenyl)-5-oxo-4,5-dihydropyrano­[3,2-*c*]­chromene-3- carbonitrile (**4q**)

The product was obtained as a yellow solid (0.82 mmol, 297 mg, 82%); mp 249–250 °C; FT-IR (KBr, cm^–1^): 3571, 3151, 3389, 3308, 2217, 1711, 1666, 1616, 1556, 1503, 1291, 1185, 754. ^1^H NMR (DMSO-*d*_6_, 500 MHz): δ 8.92 (s, 1H), 7.90 (dd, *J* = 7.9 and 1.6 Hz, 1H), 7.80–7.66 (m, 1H), 7.58–7.42 (m, 2H), 7.32 (s, 2H), 6.82 (d, *J* = 2.1 Hz, 1H), 6.71 (d, *J* = 8.1 Hz, 1H), 6.62 (dd, *J* = 8.1, 2.1 Hz, 1H), 4.37 (s, 1H), 3.74 (s, 3H) ppm. ^13^C {^1^H} NMR (DMSO-*d*_6_, 125 MHz): δ 160.0, 158.4, 153.5, 152.5, 147.8, 146.2, 134.8, 133.3, 125.1, 122.9, 120.4, 119.8, 117.0, 116.0, 113.5, 112.6, 104.8, 58.8, 56.2, 37.0 ppm. HRMS (ESI) *m*/*z*: [M + Na]^+^, 385.0795 calcd for C_20_H_14_N_2_O_5_; found, 385.0793.

#### 2-Amino-4-(2-nitrophenyl)-5-oxo-4,5-dihydropyrano­[3,2-*c*]­chromene-3-carbonitrile (**4r**)

The product was obtained as a yellow solid (0.93 mmol, 336 mg, 93%); mp 241–243 °C; FT-IR (KBr, cm^–1^): 3304, 3305, 3178, 2188, 1701, 1672, 1595, 1601, 1487, 1531, 1376, 1224, 1167, 768. ^1^H NMR (DMSO-*d*_6_, 500 MHz): δ 7.91–7.89 (m, 2H), 7.73–7.71 (m, 1H), 7.69–7.63 (m, 1H), 7.55–7.54 (m, 3H), 7.51–7.49 (m, 2H), 7.47–7.44 (m, 1H), 5.25 (s, 1H) ppm. ^13^C {^1^H} NMR (DMSO-*d*_6_, 125 MHz): δ 160.2, 159.1, 154.1, 152.6, 152.7, 149.7, 134.1, 133.6, 131.6, 128.9, 125.2, 124.4, 123.0, 119.1, 117.1, 113.3, 103.8, 56.6, 32.0 ppm. HRMS (ESI) *m*/*z*: [M + H]^+^, 362.0771 calcd for C_19_H_11_N_3_O_5_; found, 362.0779; *m*/*z* [M + Na]^+^, 384.0591 calcd; found, 384.0604.

#### 2-Amino-4-(3-nitrophenyl)-5-oxo-4*H*,5*H*-dihydropyrano­[3,2-*c*]­chromene-3-carbonitrile (**4s**)

The product was obtained as a yellow solid (0.89 mmol, 321 mg, 89%); mp 262–264 °C; FT-IR (KBr, cm^–1^): 3403, 3312, 3185, 2202, 1708, 1666, 1595, 1524, 1368, 1260, 1174, 754. ^1^H NMR (DMSO-*d*_6_, 500 MHz): δ 8.13 (d, *J* = 7.9 Hz, 2H), 7.93 (dd, *J* = 7.9 and 1.6 Hz, 1H), 7.86–7.78 (m, 1H), 7.78–7.68 (m, 1H), 7.64 (t, *J* = 7.8 Hz, 1H), 7.56–7.43 (m, 4H), 4.74 (s, 1H) ppm. ^13^C­{^1^H} NMR (DMSO-*d*_6_, 125 MHz): δ 160.1, 158.6, 154.4, 152.8, 148.4, 146.0, 135.2, 133.6, 130.5, 125.2, 123.1, 122.9, 122.7, 119.4, 117.1, 113.4, 103.3, 57.4, 37.1 ppm. HRMS (ESI) *m*/*z*: [M + H]^+^, 362.0771 calcd for C_19_H_11_N_3_O_5_; found, 362.0784; *m*/*z* [M + Na]^+^, 384.0591 calcd; found, 384.0596.

#### 2-Amino-4-(4-nitrophenyl)-5-oxo-4,5-dihydropyrano­[3,2-*c*]­chromene-3-carbonitrile (**4t**)

The product was obtained as a yellow solid (0.90 mmol, 325 mg, 90%); mp 269–270 °C; FT-IR (KBr, cm^–1^): 3481, 3425, 3185, 2195, 1715, 1659, 1609, 1496, 1361, 1260, 1124, 761. ^1^H NMR (DMSO-*d*_6_, 500 MHz): δ 8.22–8.11 (m, 2H), 7.92 (dd, *J* = 7.9 and 1.6 Hz, 1H), 7.73 (ddd, *J* = 8.7, 7.5, and 1.7 Hz, 1H), 7.63–7.57 (m, 2H), 7.57–7.41 (m, 4H), 4.67 (s, 1H) ppm. ^13^C {^1^H} NMR (DMSO-*d*_6_, 125 MHz): δ 160.0, 158.5, 154.4, 152.8, 151.2, 147.10, 133.6, 129.6, 125.2, 124.2, 123.0, 119.3, 117.1, 113.4, 103.3, 57.3, 37.3 ppm. HRMS (ESI) *m*/*z*: [M + H]^+^, 362.0771 calcd for C_19_H_11_N_3_O_5_; found, 362.0773; *m*/*z* [M + Na]^+^, 384.0591 calcd; found, 384.0587.

#### 2-Amino-4-(4-metoxyphenyl)-5-oxo-4,5-dihydropyrano­[3,2-*c*]­chromene-3-carbonitrile (**4u**)

The product was obtained as a yellow solid (0.94 mmol, 325 mg, 94%); mp 220–222 °C; FT-IR (KBr, cm^–1^): 3383, 3325, 3192, 2195, 1701, 1666, 1595, 1496, 1383, 1270, 1185, 825. ^1^H NMR (DMSO-*d*_6_, 500 MHz): δ 7.90 (dd, *J* = 7.9 and 1.6 Hz, 1H), 7.71 (ddd, *J* = 8.8, 7.5, and 1.6 Hz, 1H), 7.55–7.42 (m, 2H), 7.34 (s, 2H), 7.19–7.13 (m, 2H), 6.90–6.78 (m, 2H), 4.40 (s, 1H), 3.72 (s, 3H) ppm. ^13^C {^1^H} NMR (DMSO-*d*_6_, 125 MHz): δ 160.0, 158.8, 158.4, 153.6, 152.6, 135.9, 133.3, 129.2, 125.1, 122.9, 119.7, 117.0, 114.3, 113.5, 104.8, 58.7, 55.5, 36.65 ppm. HRMS (ESI) *m*/*z*: [M + Na]^+^, 369.0846 calcd for [C_20_H_14_N_2_O_4_ + Na]^+^; found, 369.0842.

#### 2-Amino-4-(4-chlorophenyl)-5-oxo-4,5-dihydropyrano­[3,2-*c*]­chromene-3-carbonitrile (**4v**)

The product was obtained as a yellow solid (0.93 mmol, 329 mg, 93%); mp 249–251 °C; FT-IR (KBr, cm^–1^): 3389, 3325, 3178, 2188, 1715, 1679, 1609, 1481, 1270, 1185, 754. ^1^H NMR (DMSO-*d*_6_, 500 MHz): δ 7.91 (dd, *J* = 8.0 and 1.6 Hz, 1H), 7.72 (ddd, *J* = 8.6, 7.5, and 1.6 Hz, 1H), 7.56–7.40 (m, 4H), 7.40–7.28 (m, 4H), 4.49 (s, 1H) ppm. ^13^C­{^1^H} NMR (DMSO-*d*_6_, 125 MHz): δ 160.0, 158.4, 154.0, 152.7, 142.8, 133.5, 132.2, 130.1, 128.9, 125.2, 123.0, 119.5, 117.1, 113.4, 104.0, 58.0, 36.9 ppm. HRMS (ESI) *m*/*z*: [M + H]^+^, 351.0531 calcd for C_19_H_11_ClN_2_O_3_; found, 351.0529; *m*/*z* [M + Na]^+^, 373.0350 calcd; found, 373.0346.

#### 2-Amino-4-(*p*-tolyl)-5-oxo-4,5-dihydropyrano­[3,2-*c*]­chromene-3-carbonitrile (**4w**)

The product was obtained as a yellow solid (0.90 mmol, 300 mg, 90%); mp 245–247 °C; FT-IR (KBr, cm^–1^): 3383, 3315, 3194, 2191, 1707, 1672, 1601, 1579, 1373, 747. ^1^H NMR (DMSO-*d*_6_, 500 MHz): δ 7.91 (dd, *J* = 7.9, 1.6 Hz, 1H), 7.71 (ddd, *J* = 8.6, 7.4, 1.7 Hz, 1H), 7.53–7.44 (m, 2H), 7.35 (s, 2H), 7.20–7.04 (m, 4H), 4.40 (s, 1H), 2.26 (s, 3H) ppm. ^13^C­{^1^H} NMR (DMSO-*d*_6_, 125 MHz): δ 160.0, 158.4, 153.7, 152.6, 140.9, 136.7, 133.4, 129.5, 128.0, 125.1, 123.0, 119.7, 117.0, 113.5, 104.6, 58.6, 37.0, 21.0 ppm. HRMS (ESI) *m*/*z*: [M + H]^+^, 331.1077 calcd for C_20_H_14_N_2_O_3_; found, 331.1071; *m*/*z* [M + Na]^+^, 353.0897 calcd; found, 353.0897.

#### 2-Amino-4-(furan-2-yl)-5-oxo-4,5-dihydropyrano­[3,2-*c*]­chromene-3-carbonitrile (**4x**)

The product was obtained as a yellow solid (0.72 mmol, 220 mg, 72%); mp 253–255 °C; FT-IR (KBr, cm^–1^): 3376, 3163, 2195, 1701, 1679, 1595, 1454, 754. ^1^H NMR (DMSO-*d*_6_, 500 MHz): δ 7.90 (dd, *J* = 7.9 and 1.6 Hz, 1H), 7.74 (td, *J* = 7.8 and 1.6 Hz, 1H), 7.54–7.42 (m, 5H), 6.39–6.38 (m, 1H), 6.28 (d, *J* = 3.2 Hz, 1H), 4.63 (s, 1H) ppm. ^13^C­{^1^H} NMR (DMSO-*d*_6_, 125 MHz): δ 159.3, 158.6, 154.4, 153.9, 153.8, 152.0, 142.3, 133.0, 124.6, 122.2, 118.8, 116.5, 112.7, 110.5, 106.3, 101.5, 30.4 ppm. HRMS (ESI) *m*/*z*: [M + Na]^+^, 329.0533 calcd for [C_17_H_10_N_2_O_4_ + Na]^+^; found, 329.0527.

## Supplementary Material



## Data Availability

The data underlying this study are available in the published article and its Supporting Information.

## Notes

The data that support the findings of this study are available from the corresponding authors upon reasonable request. Copies of ^1^H and ^13^C NMR, HR-MS (PDF)
