# Supplementary material for: Solvent-Controlled Reaction Productivity and Mechanistic Pathways in the Multicomponent Synthesis of 2‑Amino-3-cyano‑4H‑pyrans
Source: ACS Org Inorg Au. 2026 Jul 15;6(5):654–74. doi: 10.1021/acsorginorgau.6c00059 (PMC13650957; doi:10.1021/acsorginorgau.6c00059)
Supplement: Supplementary file 1 [file gg6c00059_si_001.pdf]

Supporting Information for

Solvent-Controlled Reaction Productivity and Mechanistic Pathways in the Multicomponent Synthesis of 2-Amino-3-cyano-4*H*-pyrans

Jhonathan Renner Nunes dos Santos,[a] Luciana Machado Ramos,[a]* Juliana Gonzaga de Morais Lima,[a] Saulo T. A. Passos,[b] Aline Lima de Oliveira,[b] Fabricio Machado,[b] Brenno A. D. Neto,[a,b]*

[a] Laboratory of Medicinal Chemistry and Organic Synthesis, Campus de Ciências Exatas e Tecnológicas, Universidade Estadual de Goiás, Molecular Sciences Graduate Program, Anápolis, GO 75132-400, Brazil.

[b] University of Brasília, Chemistry Institute (IQ-UnB), Laboratory of Medicinal and Technological Chemistry, Campus Universitário Darcy Ribeiro, Brasília, Distrito Federal 70910-900, Brazil.

Summary

Figure S1____**Page S2**

Figure S2____**Page S2**

NMR data of the keto-enol equilibrium____**Page S3-S5**

HR-ESI(+)-MS(/MS) of the reaction monitoring____**Page S6-S14**

NMR data of Intermediates (Int) II, IV, and V____**Page S15-S18**

IR, NMR, and HR-ESI(+)-MS(/MS) of MCR products ACP 4a-4x____________________________**Pages S19-S66**

Multilinear regression model

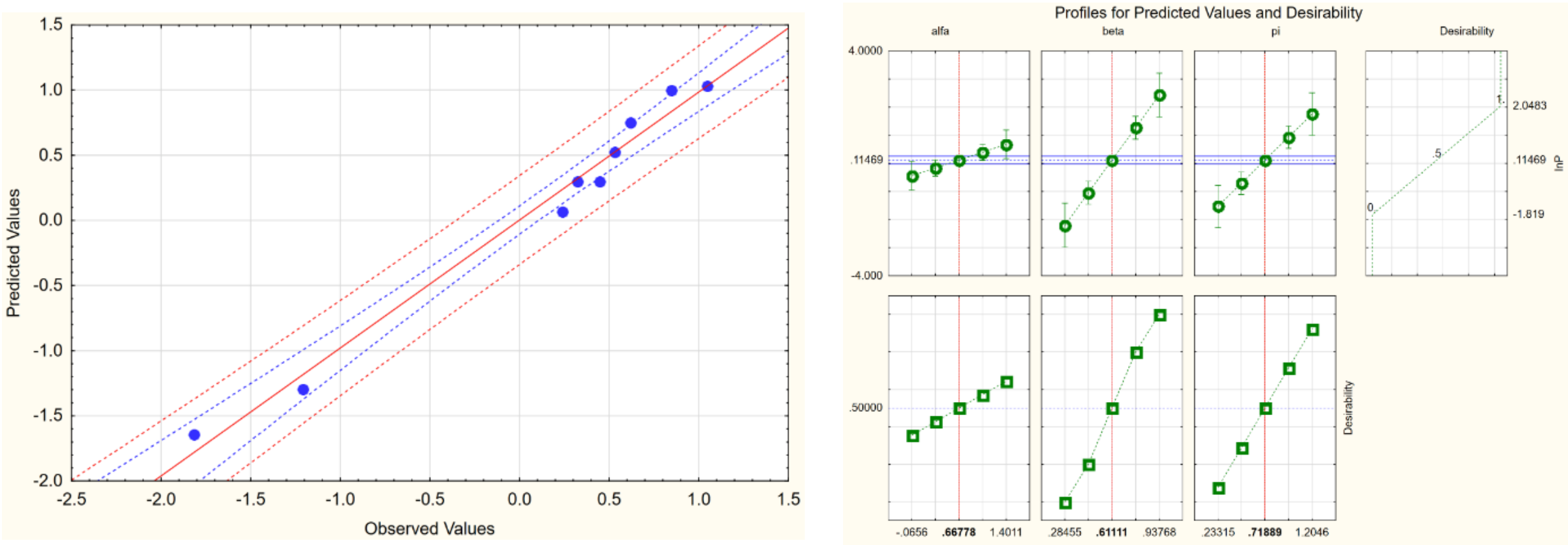

Figure S1. (Left) Comparison between predicted and observed values. The dashed lines indicate the regression bands constructed with a confidence level of 95%. (Right) Profile for predicted values and desirability.

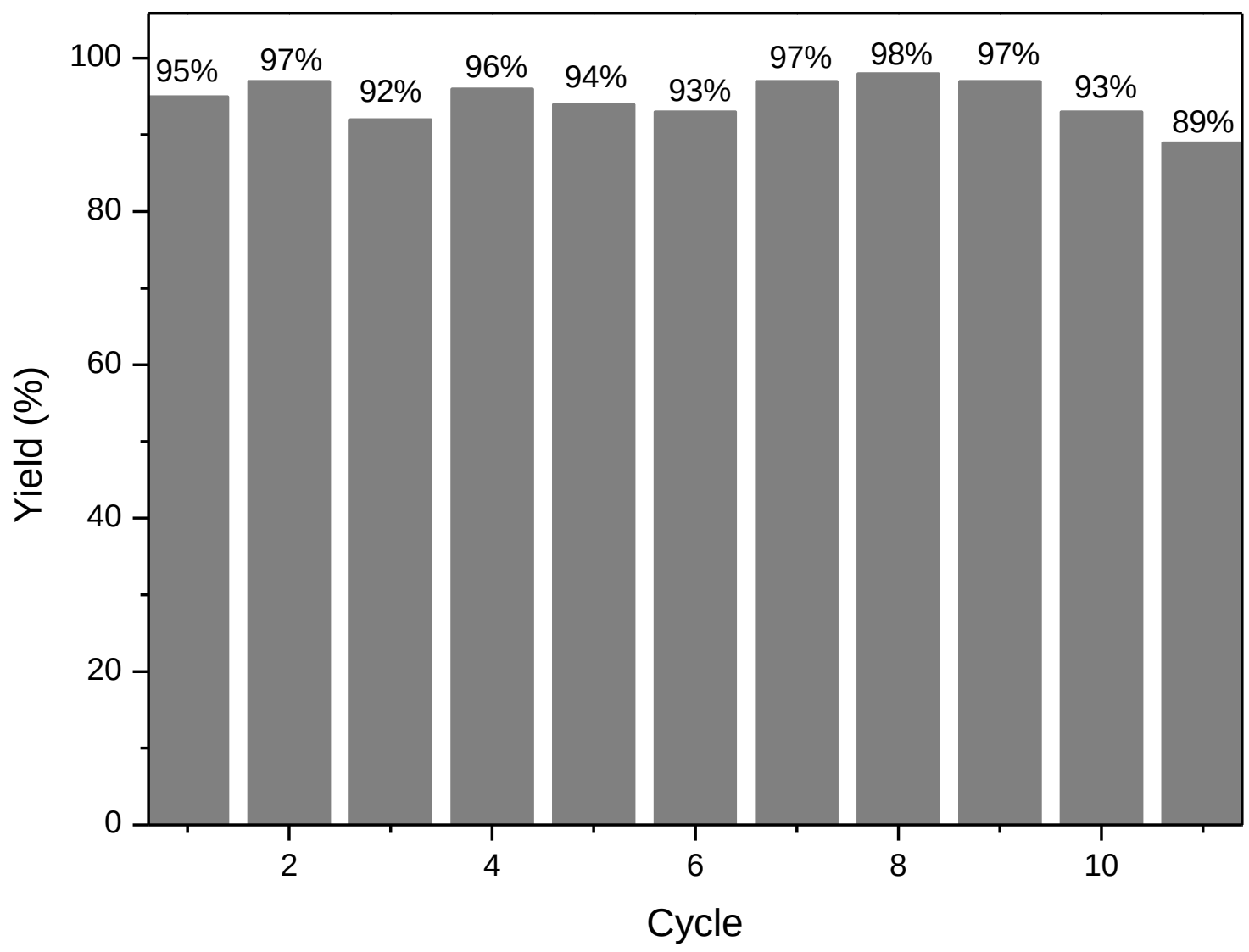

Figure S2. Catalyst recycling experiments.

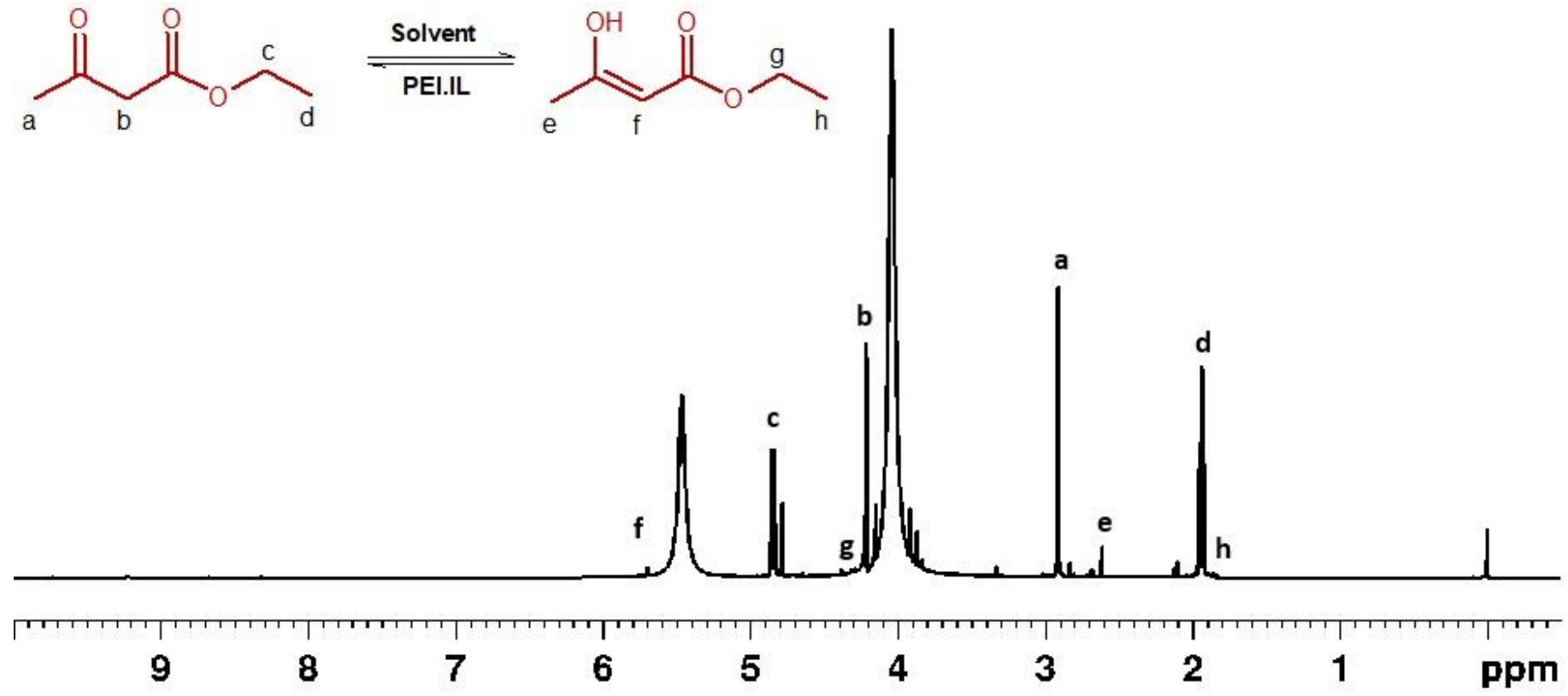

Figure S3. ^{1}H NMR (600 MHz, D_2O) of a mixture of methanol, ethyl acetoacetate and the catalyst. A sealed capillary tube filled with D_2O/TMSP was used to adjust the equipment and to set the scale (0.0 ppm).

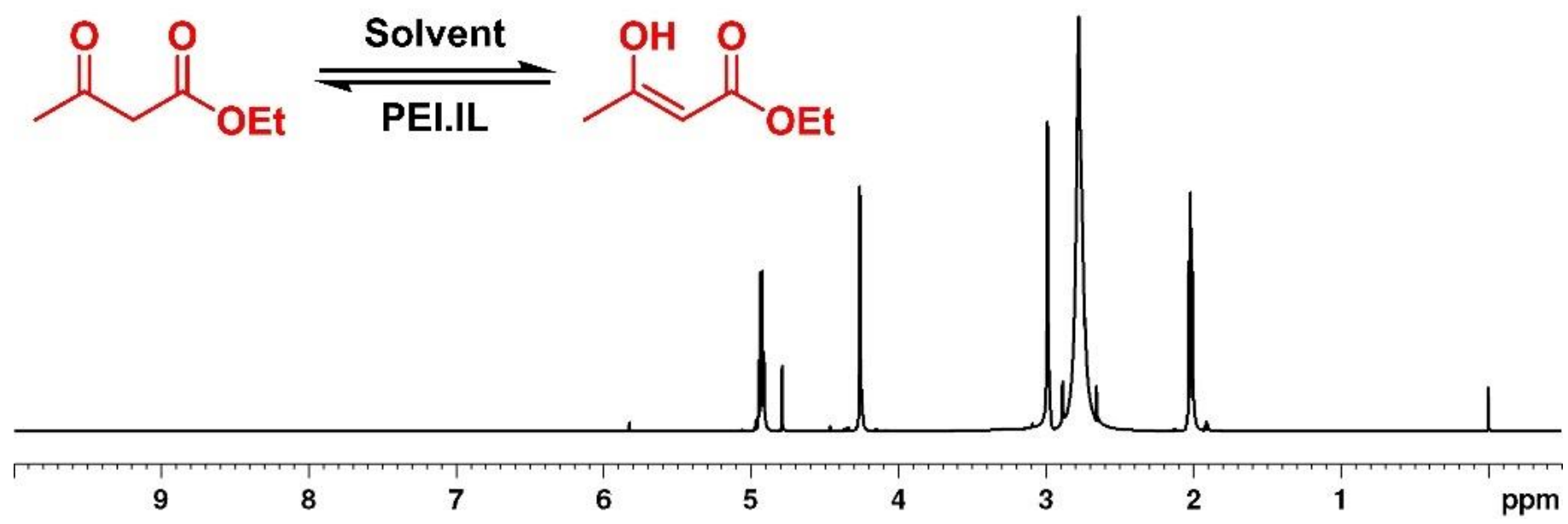

Figure S4. ^{1}H NMR (600 MHz, D_2O) of a mixture of acetonitrile, ethyl acetoacetate and the catalyst. A sealed capillary tube filled with D_2O/TMSP was used to adjust the equipment and to set the scale (0.0 ppm).

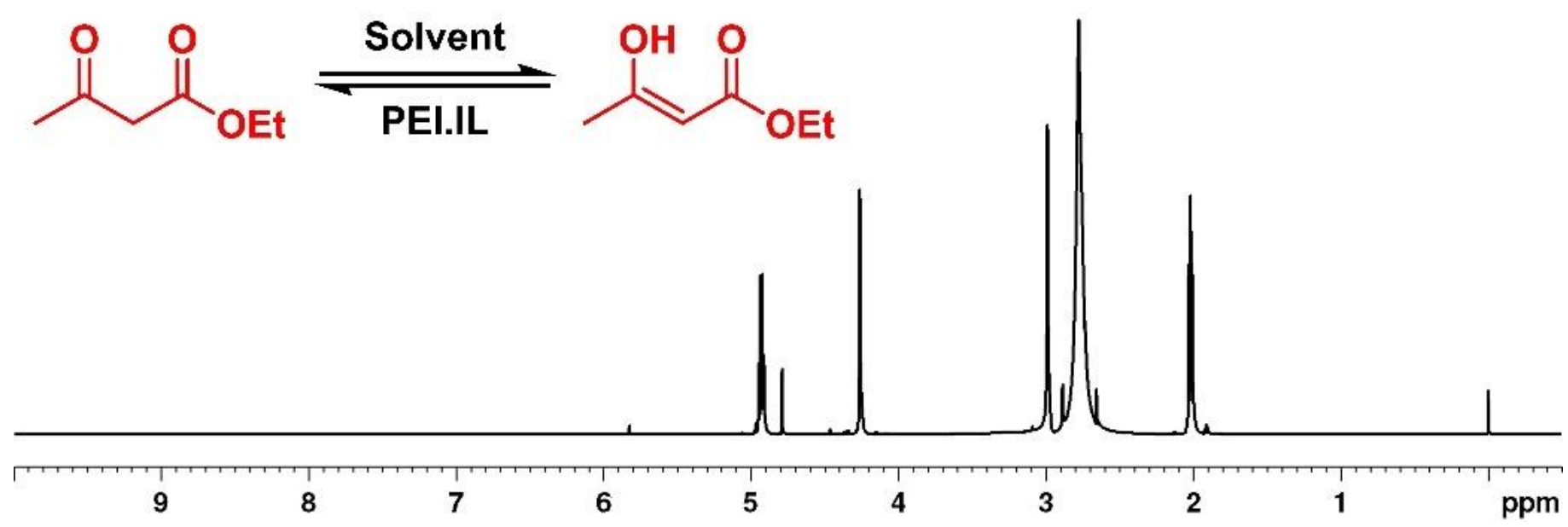

Figure S5. ^{1}H NMR (600 MHz, D_2O) of a mixture of dimethylformamide, ethyl acetoacetate and the catalyst. A sealed capillary tube filled with D_2O/TMSP was used to adjust the equipment and to set the scale (0.0 ppm).

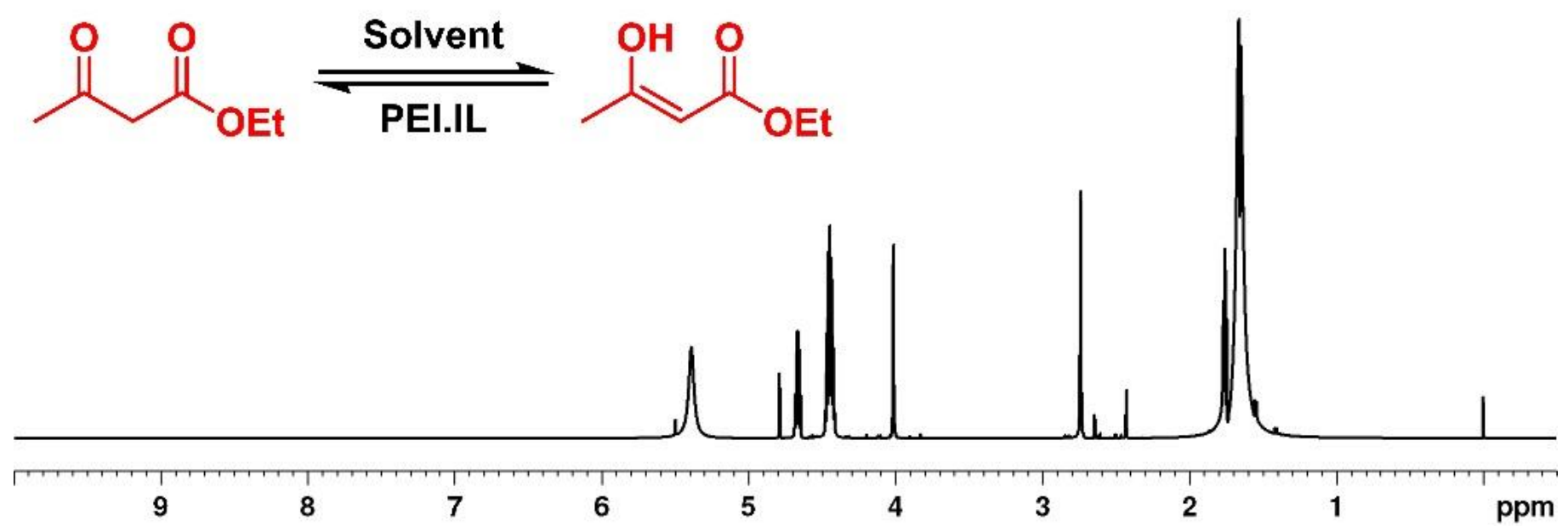

Figure S6. ^{1}H NMR (600 MHz, D_2O) of a mixture of isopropanol, ethyl acetoacetate and the catalyst. A sealed capillary tube filled with D_2O/TMSP was used to adjust the equipment and to set the scale (0.0 ppm).

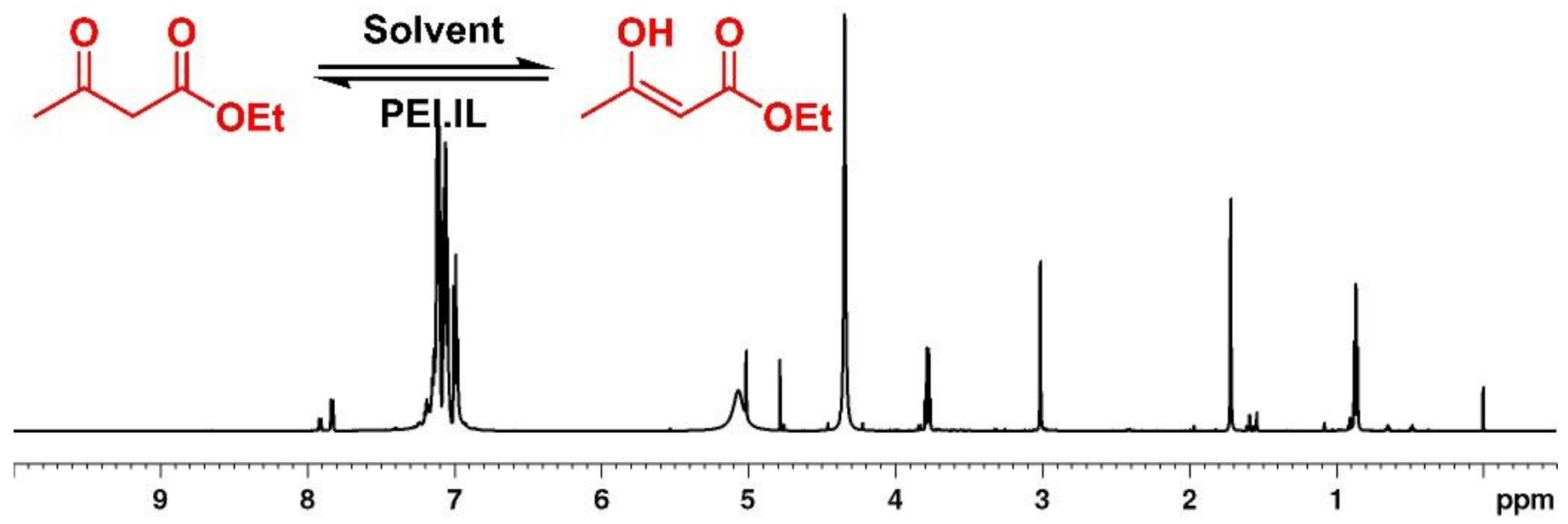

Figure S7. ^{1}H NMR (600 MHz, D_2O) of a mixture of benzyl alcohol, ethyl acetoacetate and the catalyst. A sealed capillary tube filled with D_2O/TMSP was used to adjust the equipment and to set the scale (0.0 ppm).

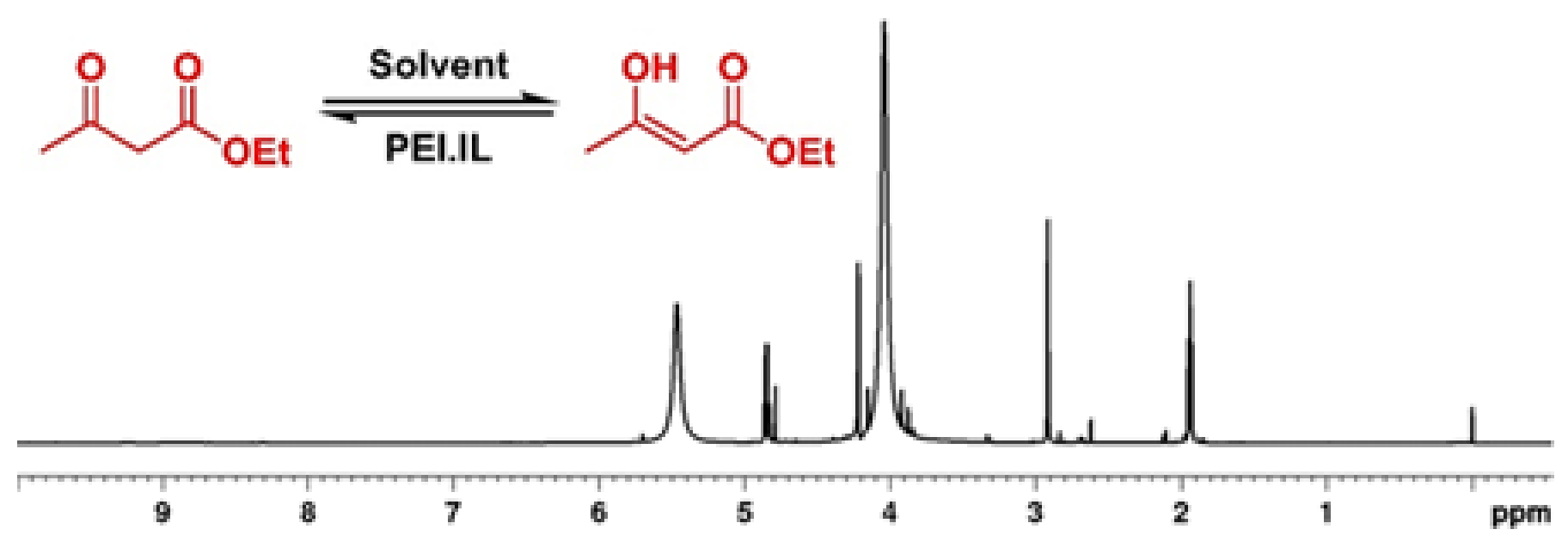

Figure S8. ^{1}H NMR (600 MHz, D_2O) of a mixture of tetrahydrofuran, ethyl acetoacetate and the catalyst. A sealed capillary tube filled with D_2O/TMSP was used to adjust the equipment and to set the scale (0.0 ppm).

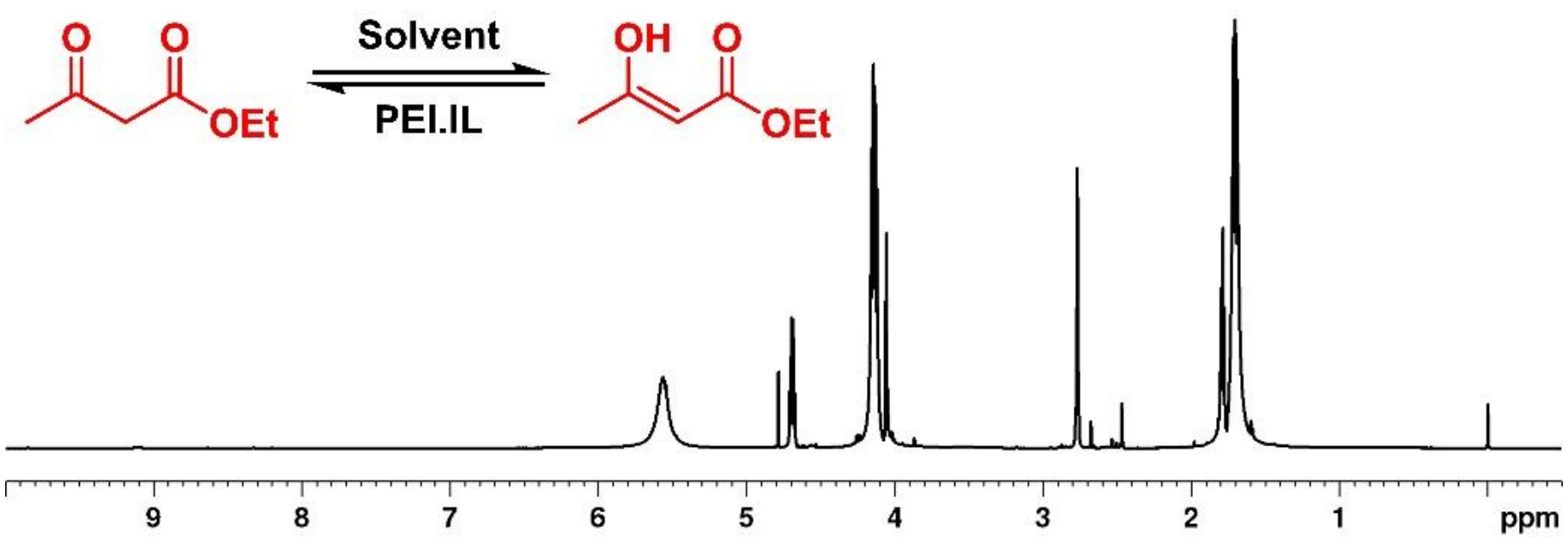

Figure S9. ^{1}H NMR (600 MHz, D_2O) of a mixture of ethanol, ethyl acetoacetate and the catalyst. A sealed capillary tube filled with D_2O/TMSP was used to adjust the equipment and to set the scale (0.0 ppm).

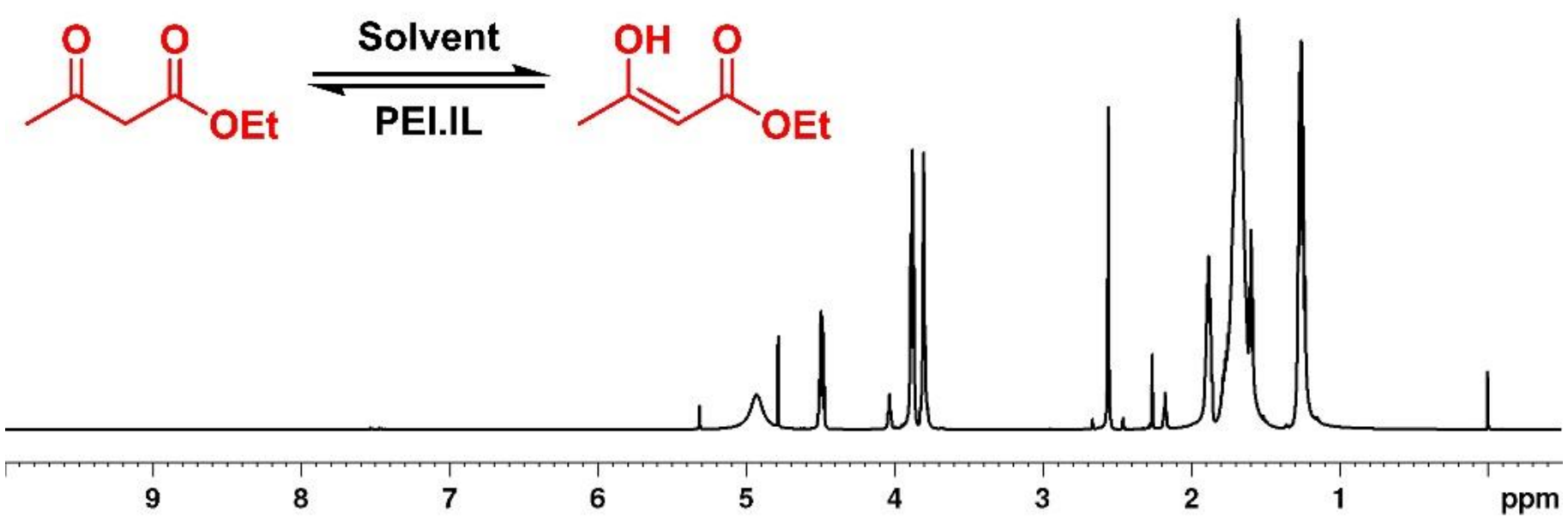

Figure S10. ^{1}H NMR (600 MHz, D_2O) of a mixture of octanol, ethyl acetoacetate and the catalyst. A sealed capillary tube filled with D_2O/TMSP was used to adjust the equipment and to set the scale (0.0 ppm).

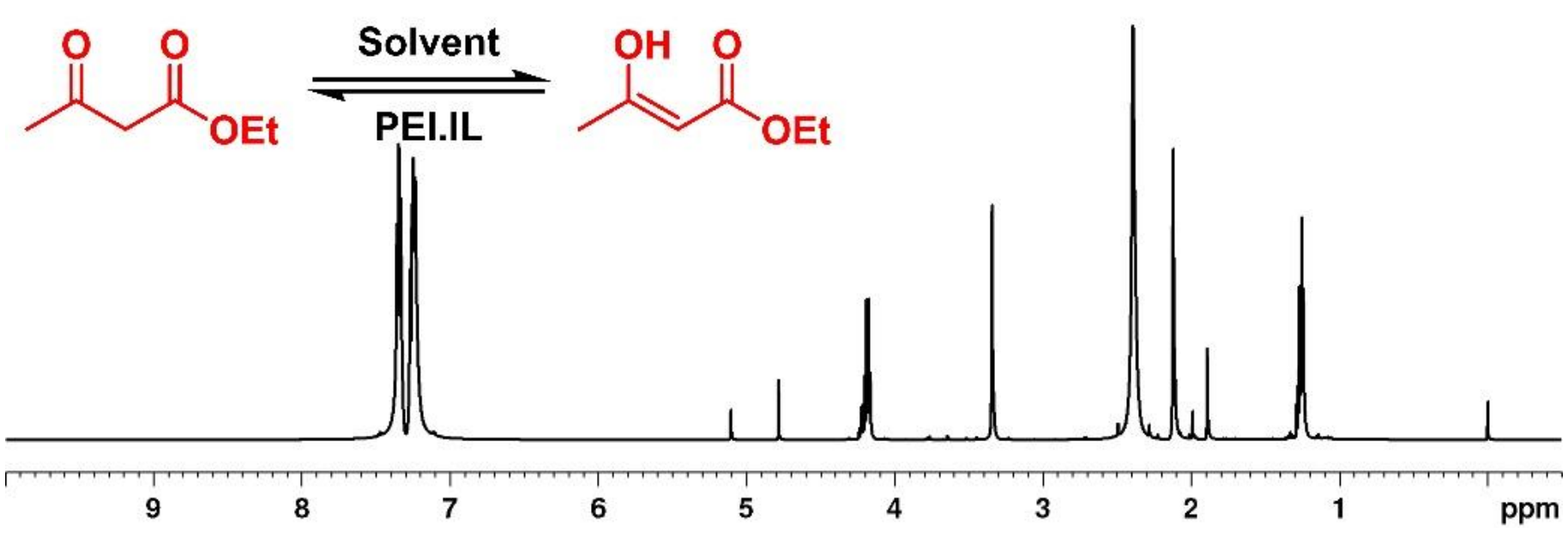

Figure S11. ^{1}H NMR (600 MHz, D_2O) of a mixture of toluene, ethyl acetoacetate and the catalyst. A sealed capillary tube filled with D_2O/TMSP was used to adjust the equipment and to set the scale (0.0 ppm).

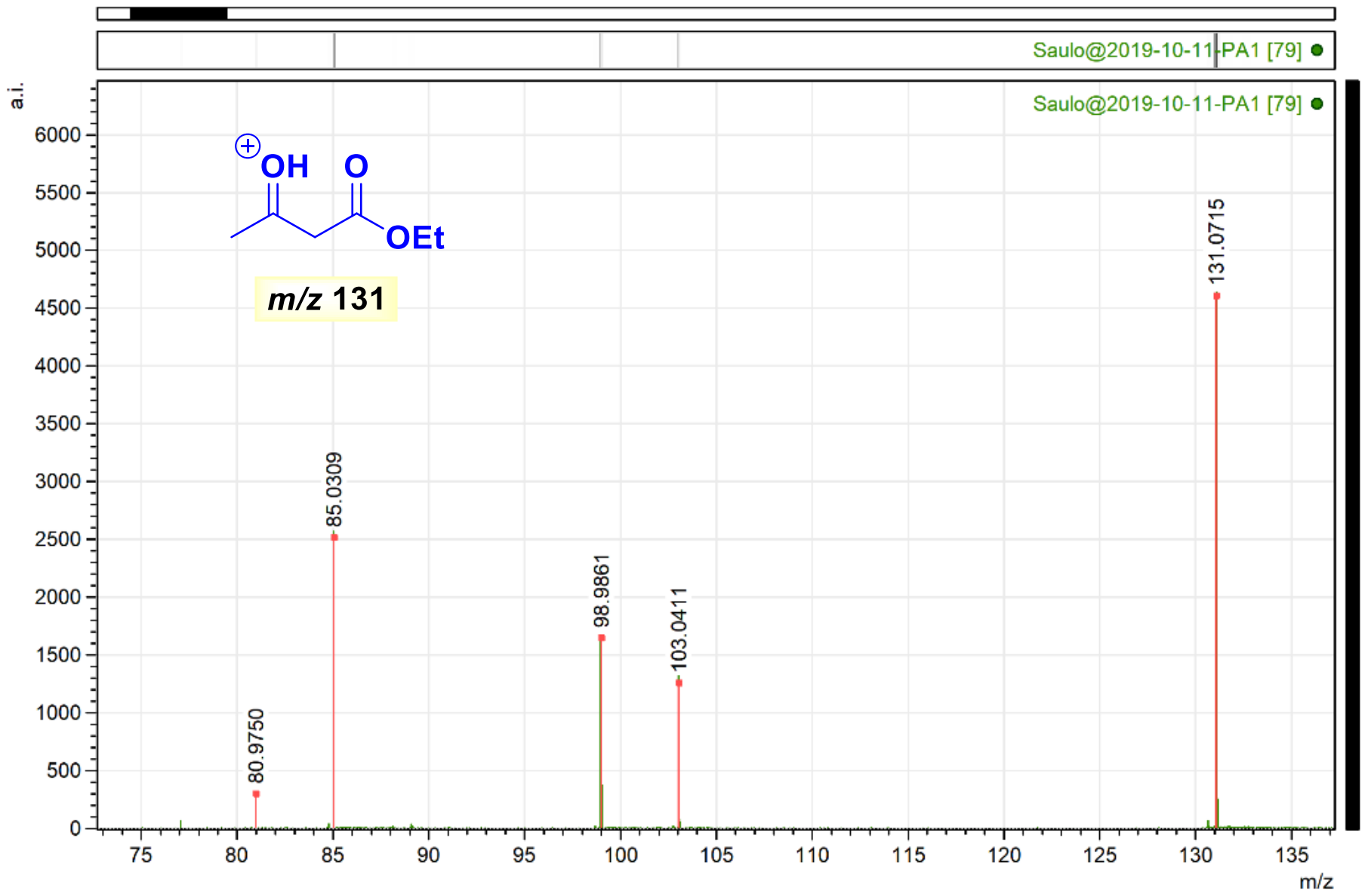

Figure S12. ESI(+)-MS/MS of the signal of *m/z* 131.

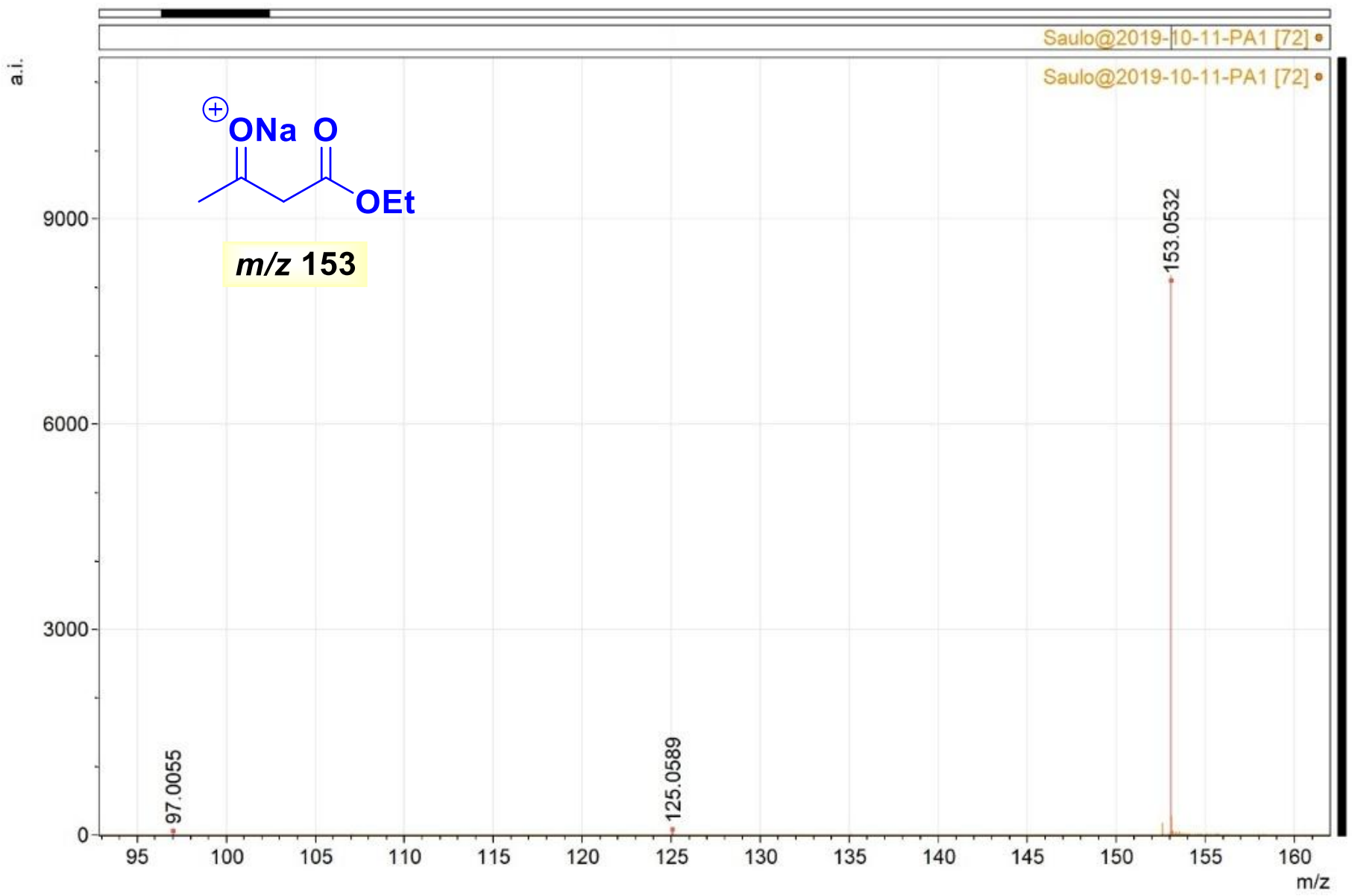

Figure S13. ESI(+)-MS/MS of the signal of *m/z* 153.

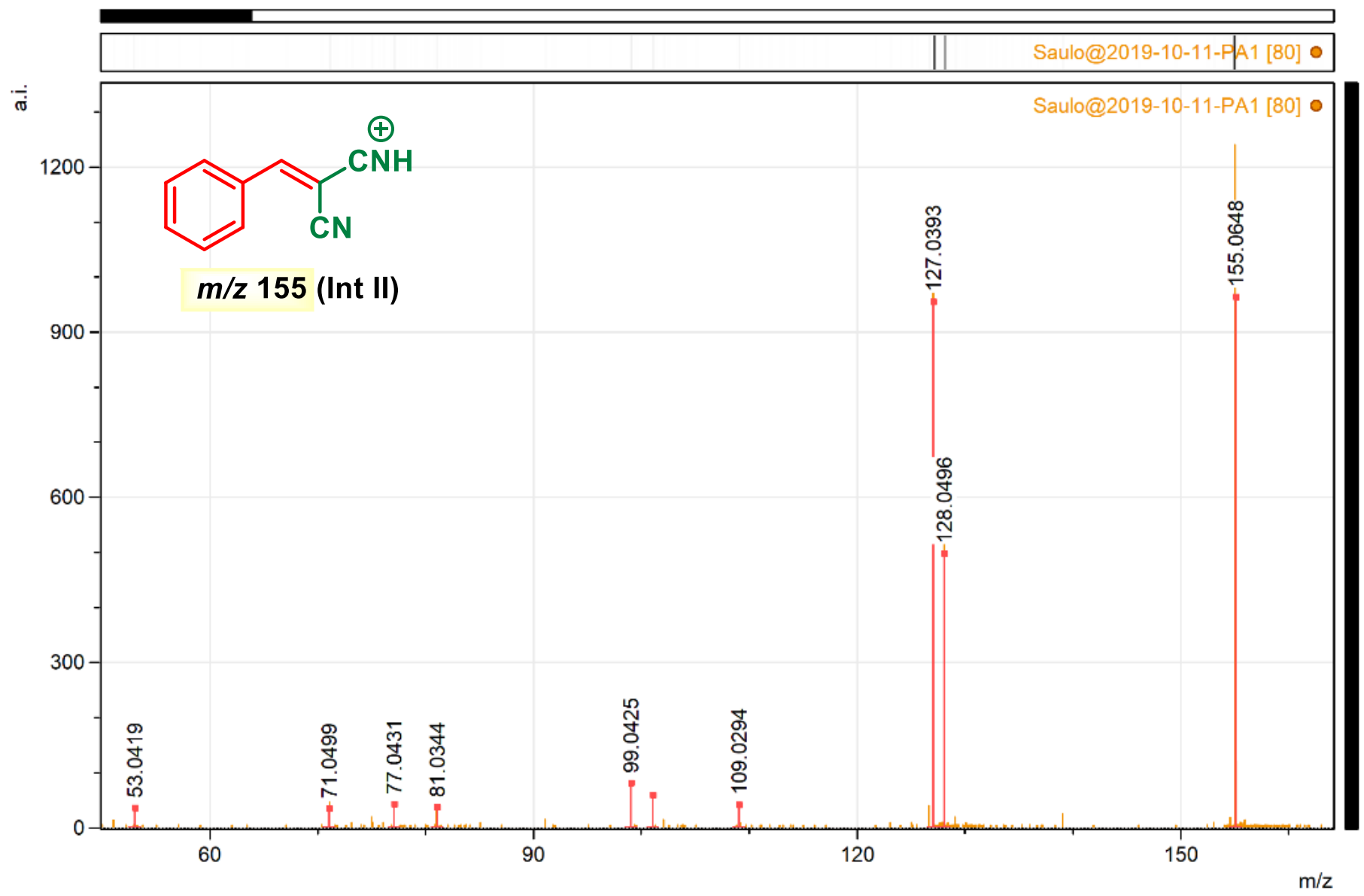

Figure S14. ESI(+)-MS/MS of the signal of *m/z* 155.

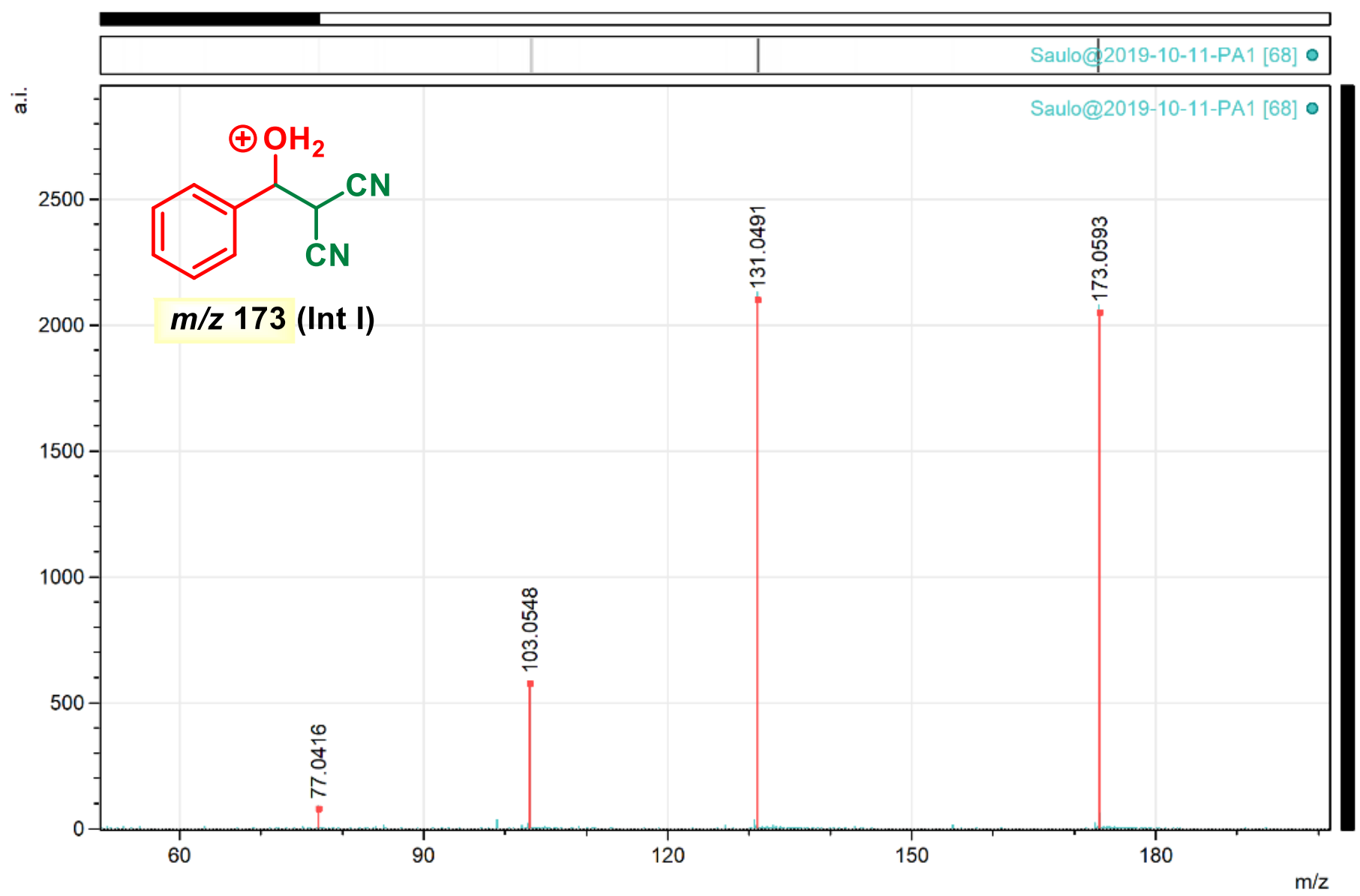

Figure S15. ESI(+)-MS/MS of the signal of *m/z* 173.

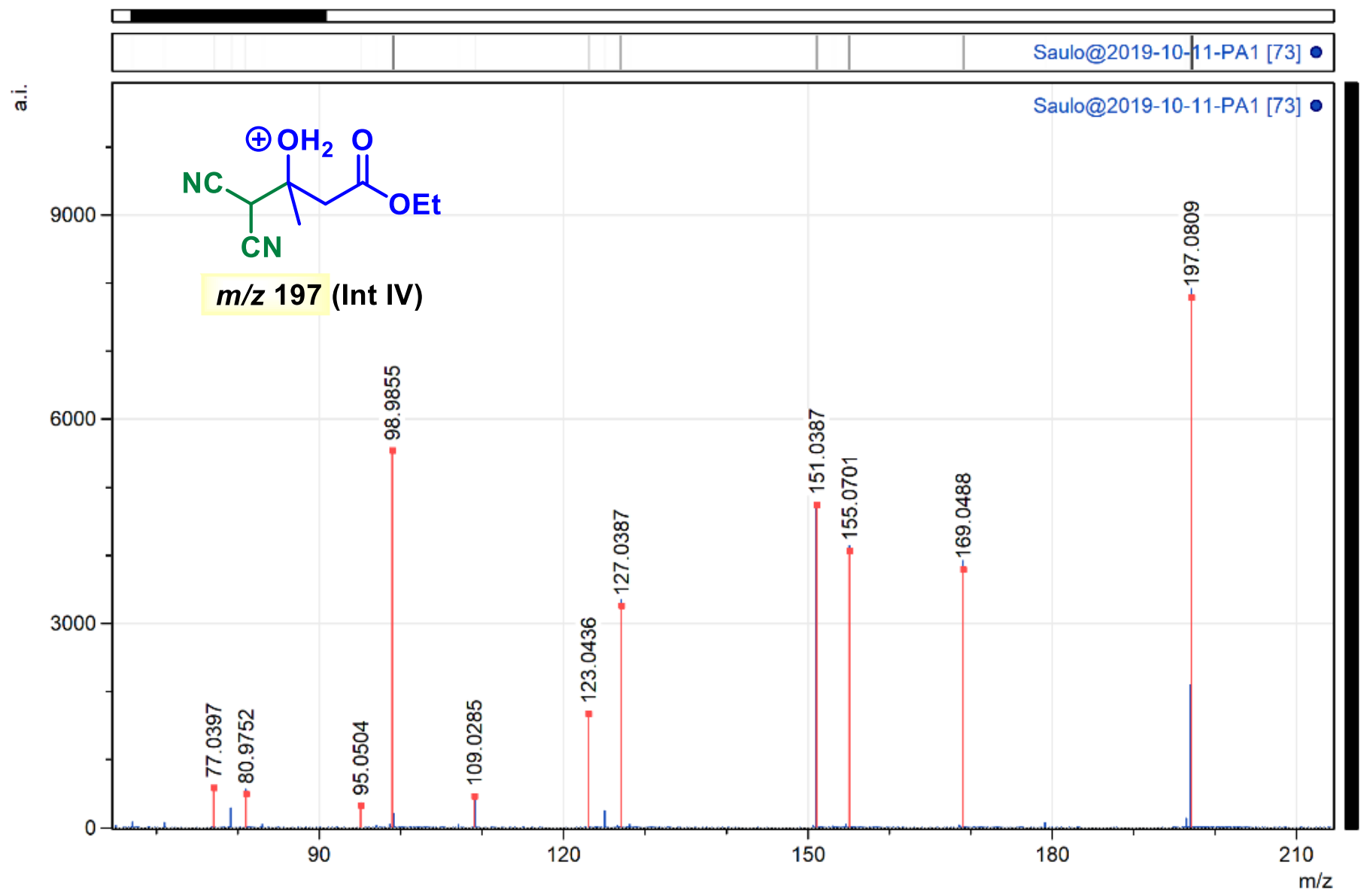

Figure S16. ESI(+)-MS/MS of the signal of *m/z* 197.

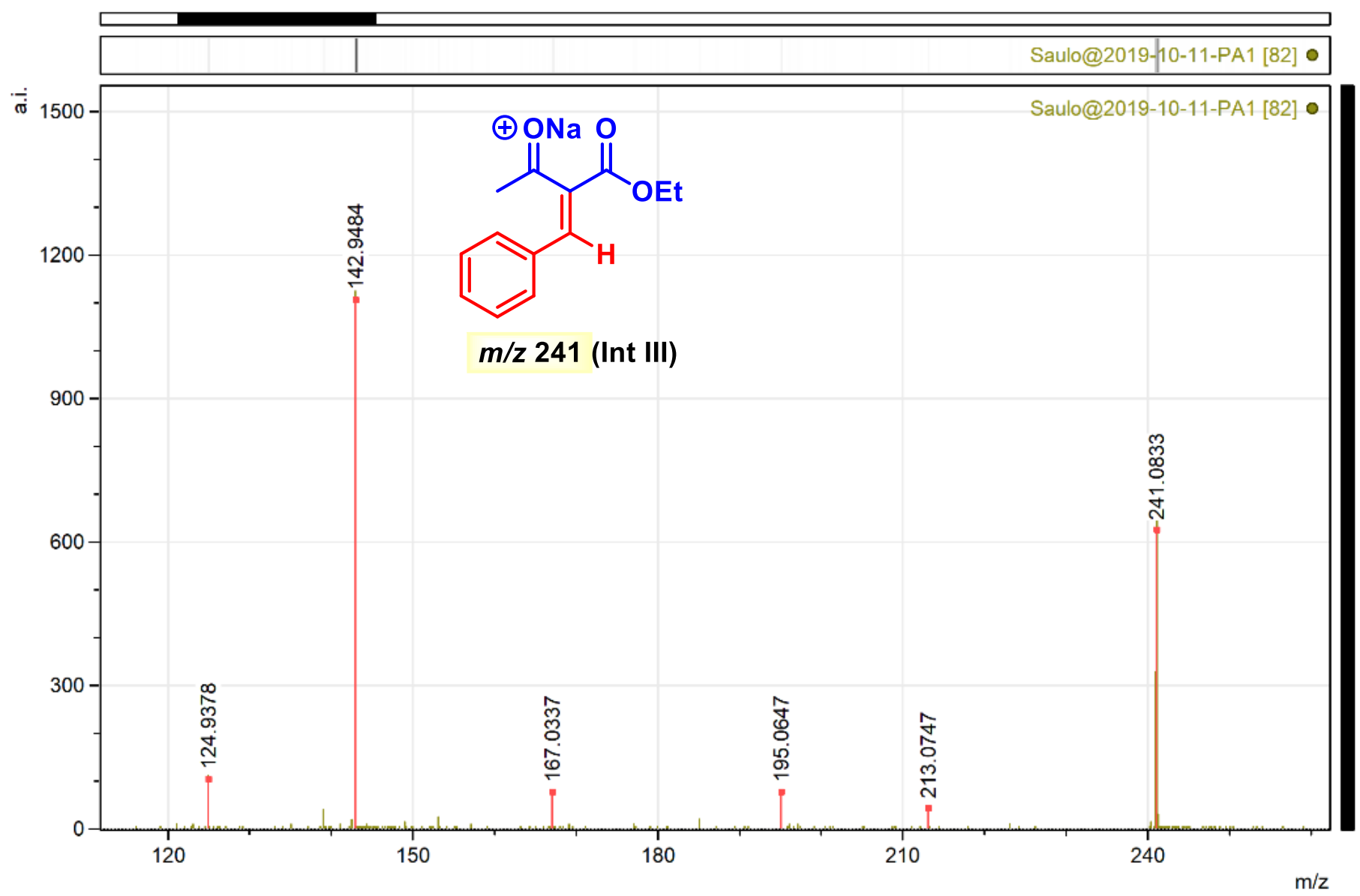

Figure S17. ESI(+)-MS/MS of the signal of *m/z* 241.

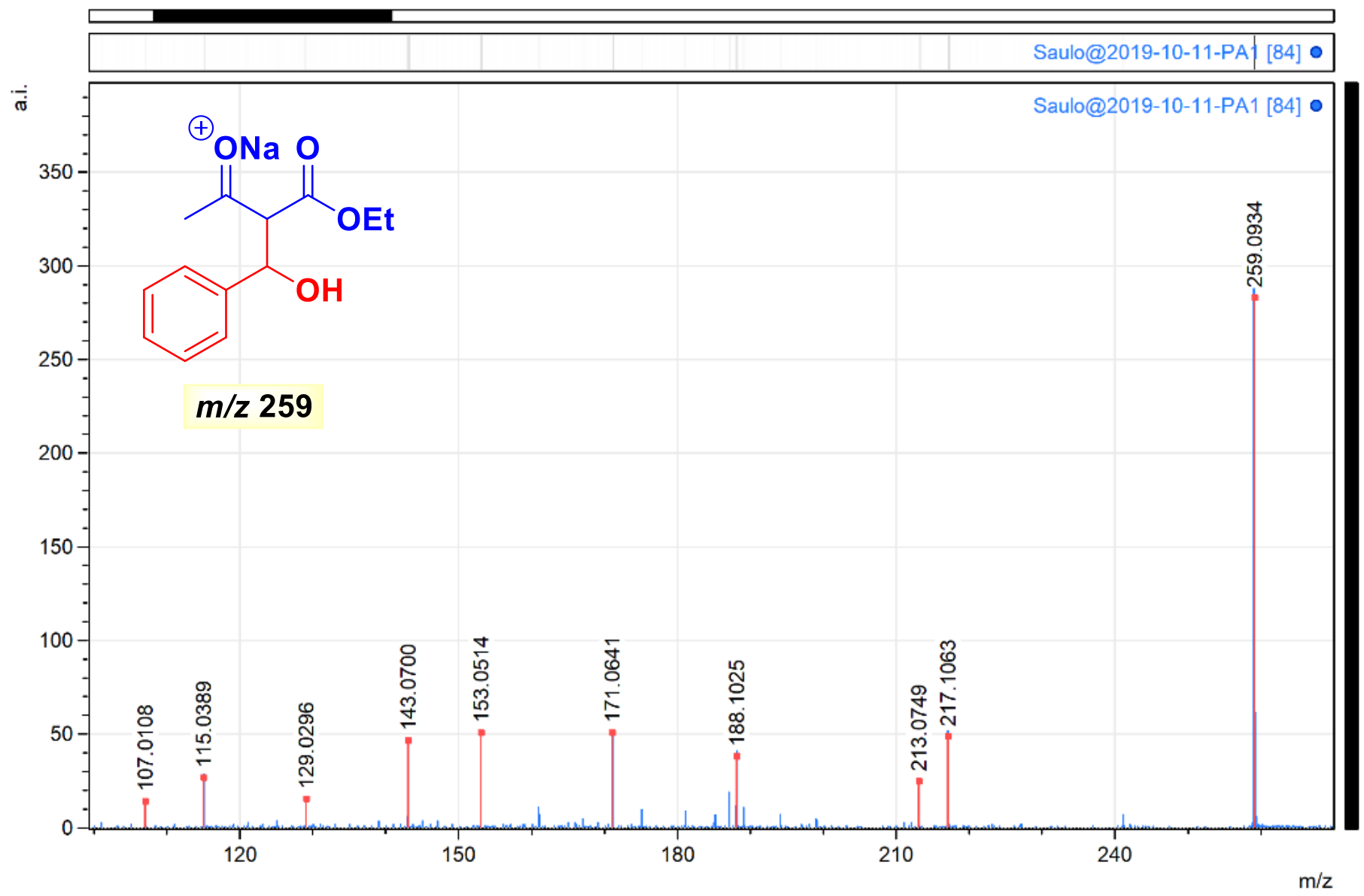

Figure S18. ESI(+)-MS/MS of the signal of *m/z* 259.

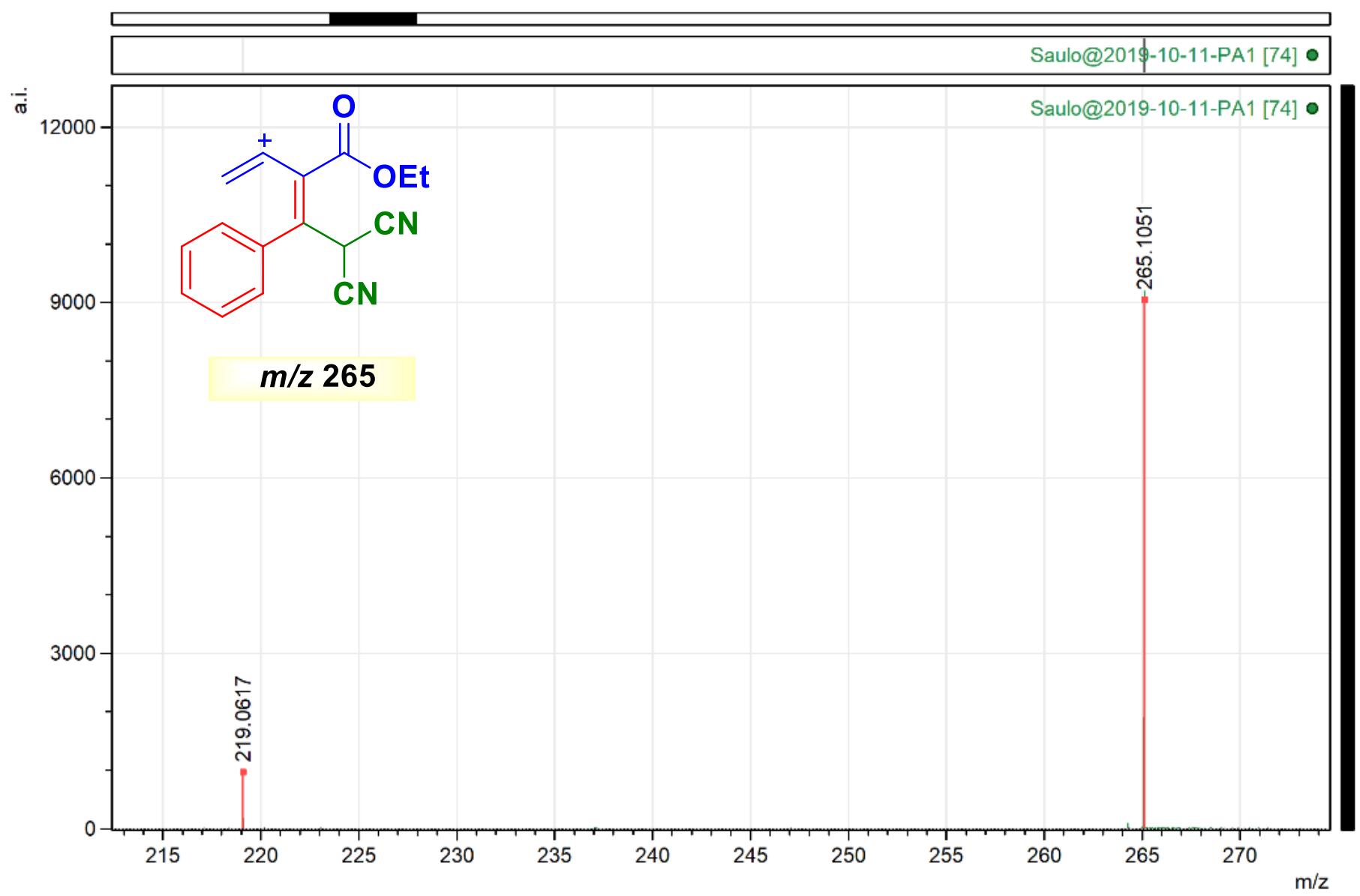

Figure S19. ESI(+)-MS/MS of the signal of *m/z* 265.

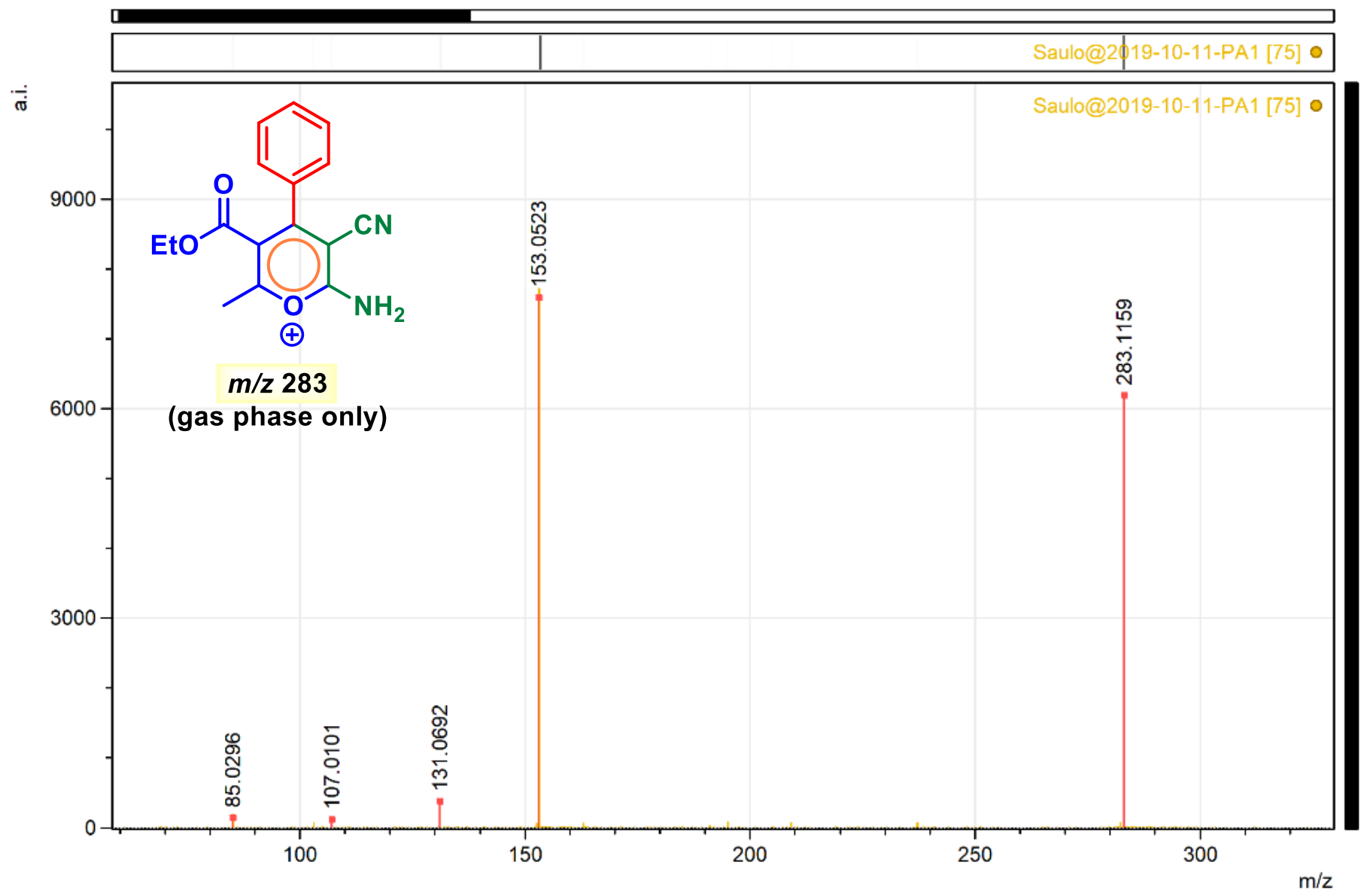

Figure S20. ESI(+)-MS/MS of the signal of *m/z* 283.

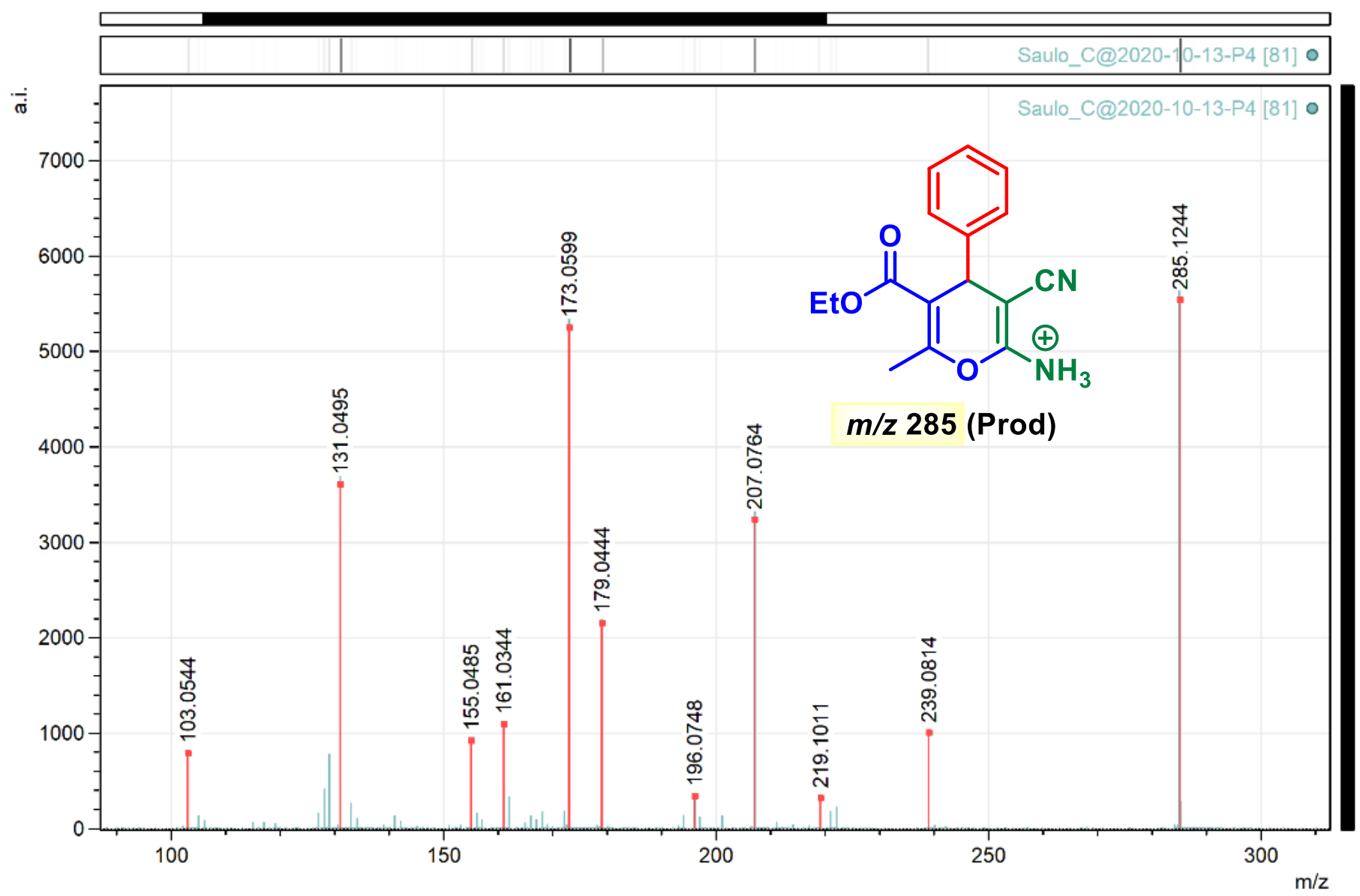

Figure S21. ESI(+)-MS/MS of the signal of *m/z* 285.

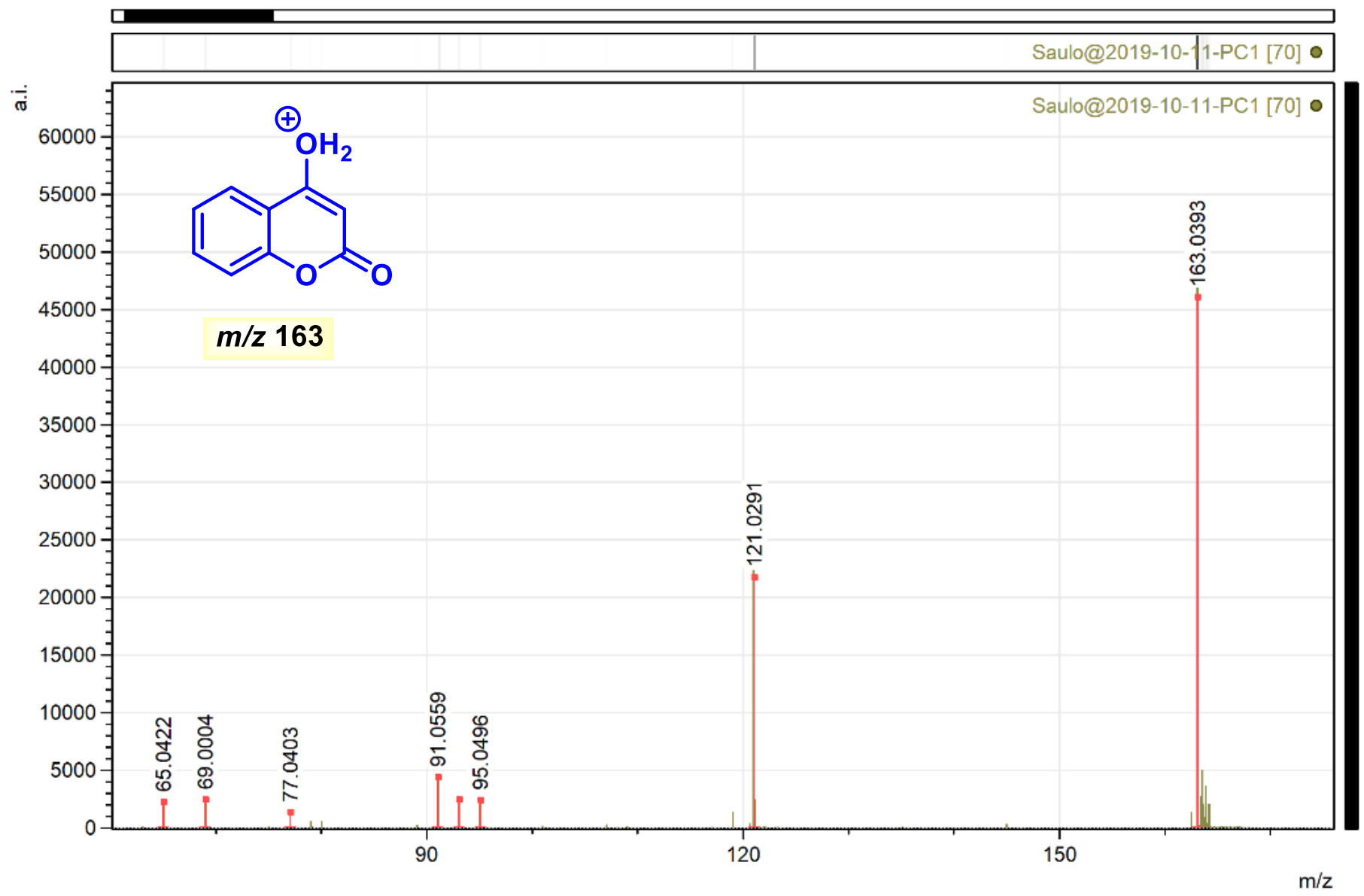

Figure S22. ESI(+)-MS/MS of the signal of *m/z* 163.

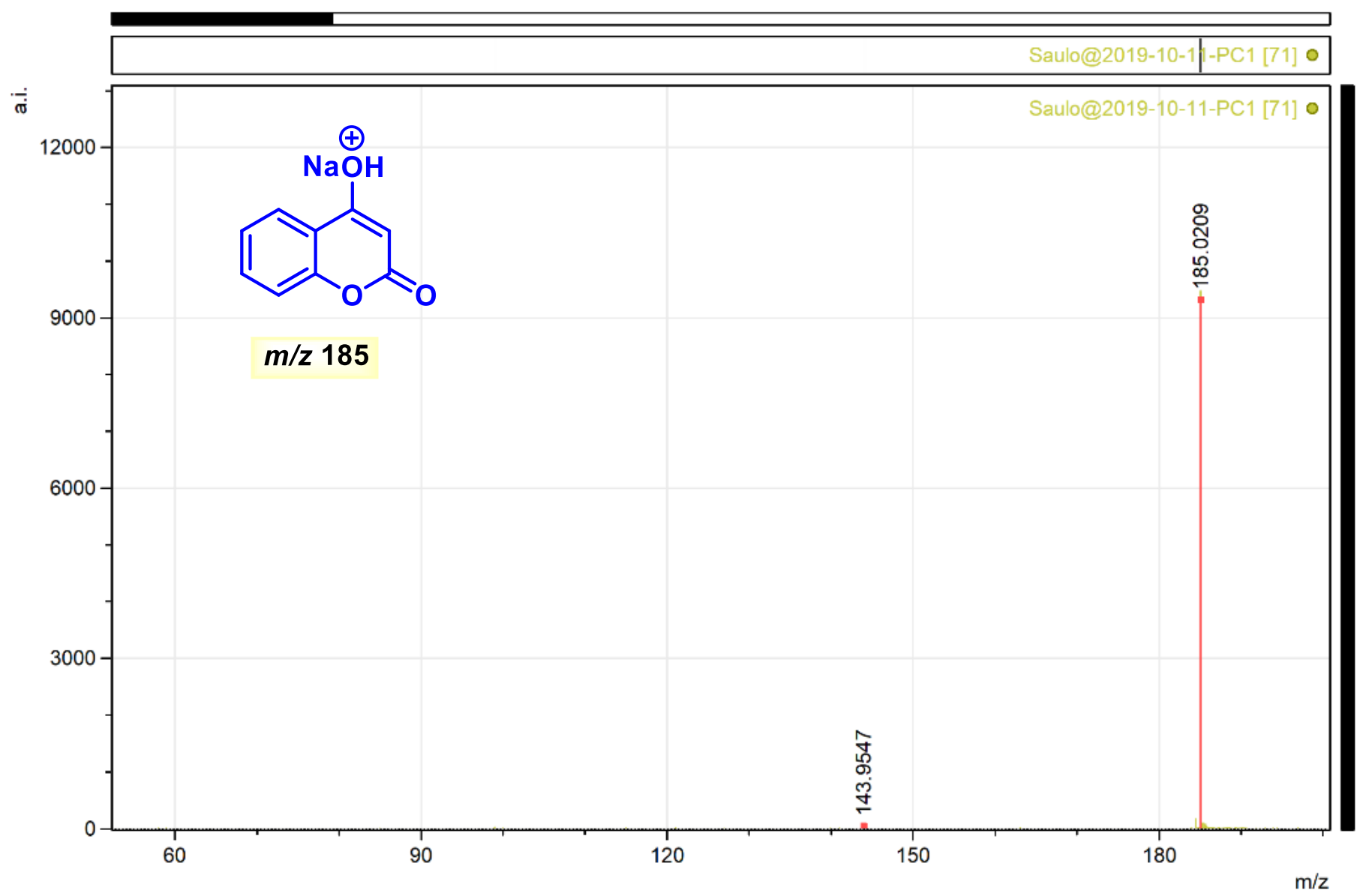

Figure S23. ESI(+)-MS/MS of the signal of *m/z* 185.

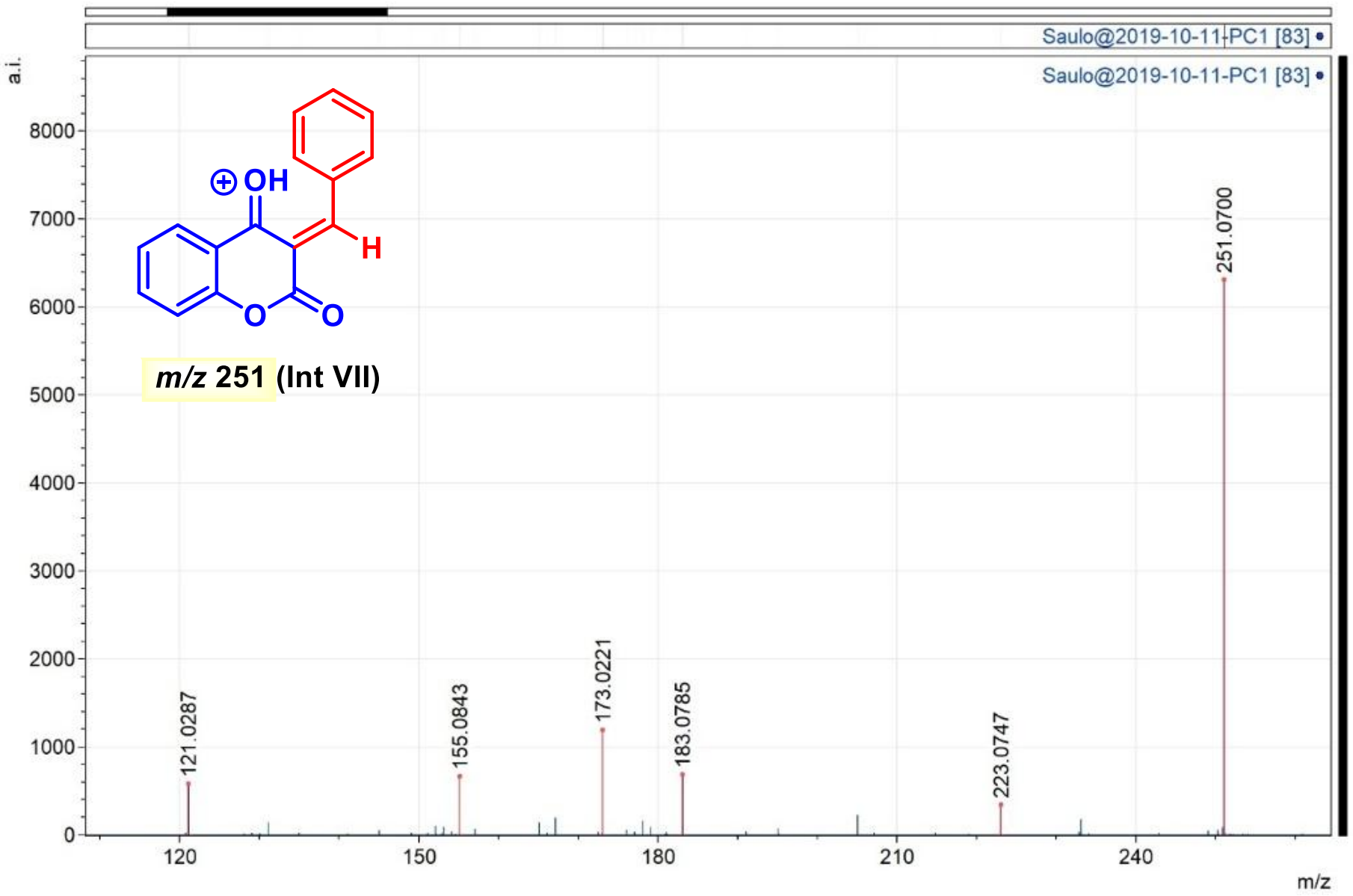

Figure S24. ESI(+)-MS/MS of the signal of *m/z* 251.

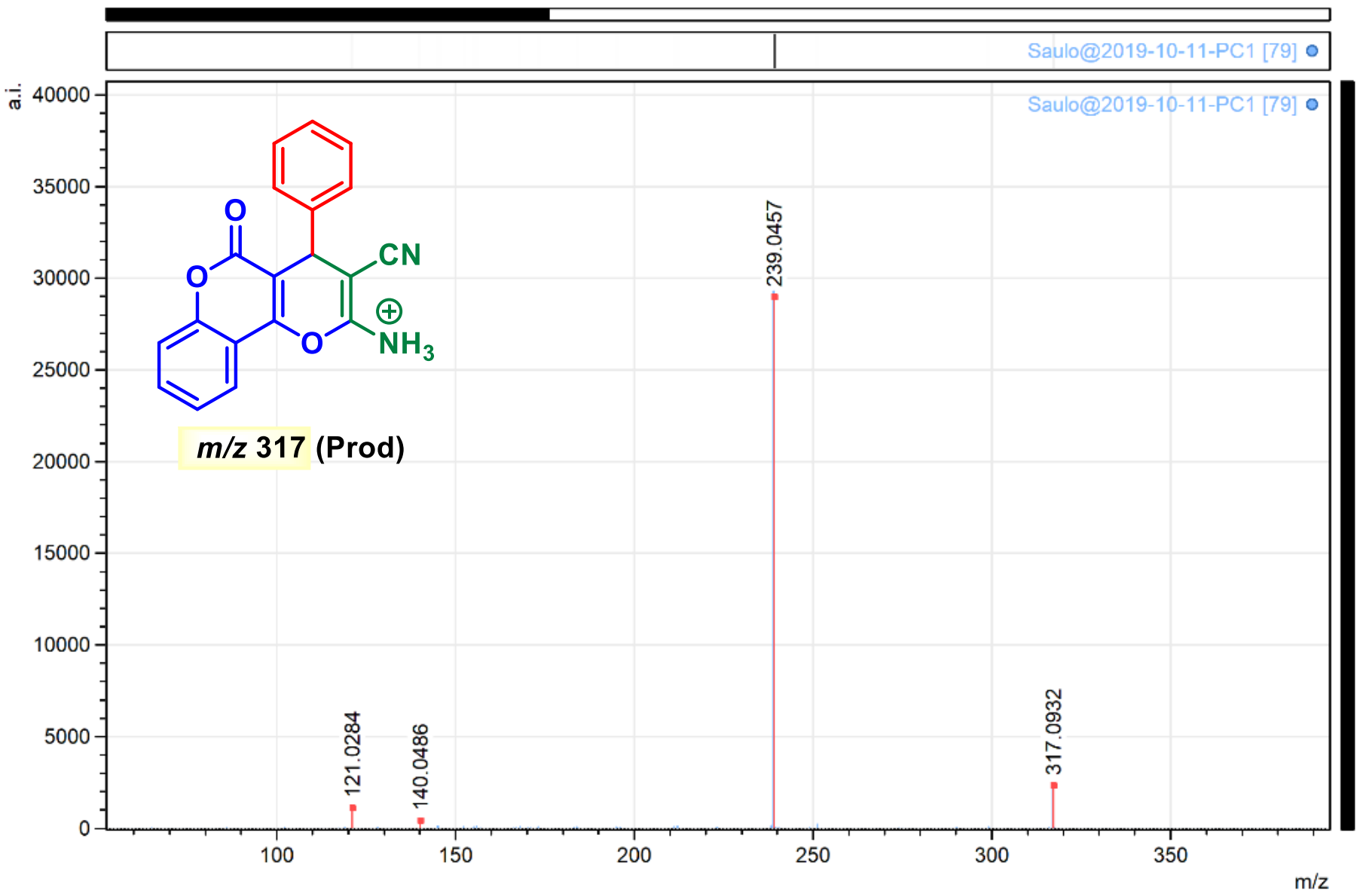

Figure S25. ESI(+)-MS/MS of the signal of *m/z* 317.

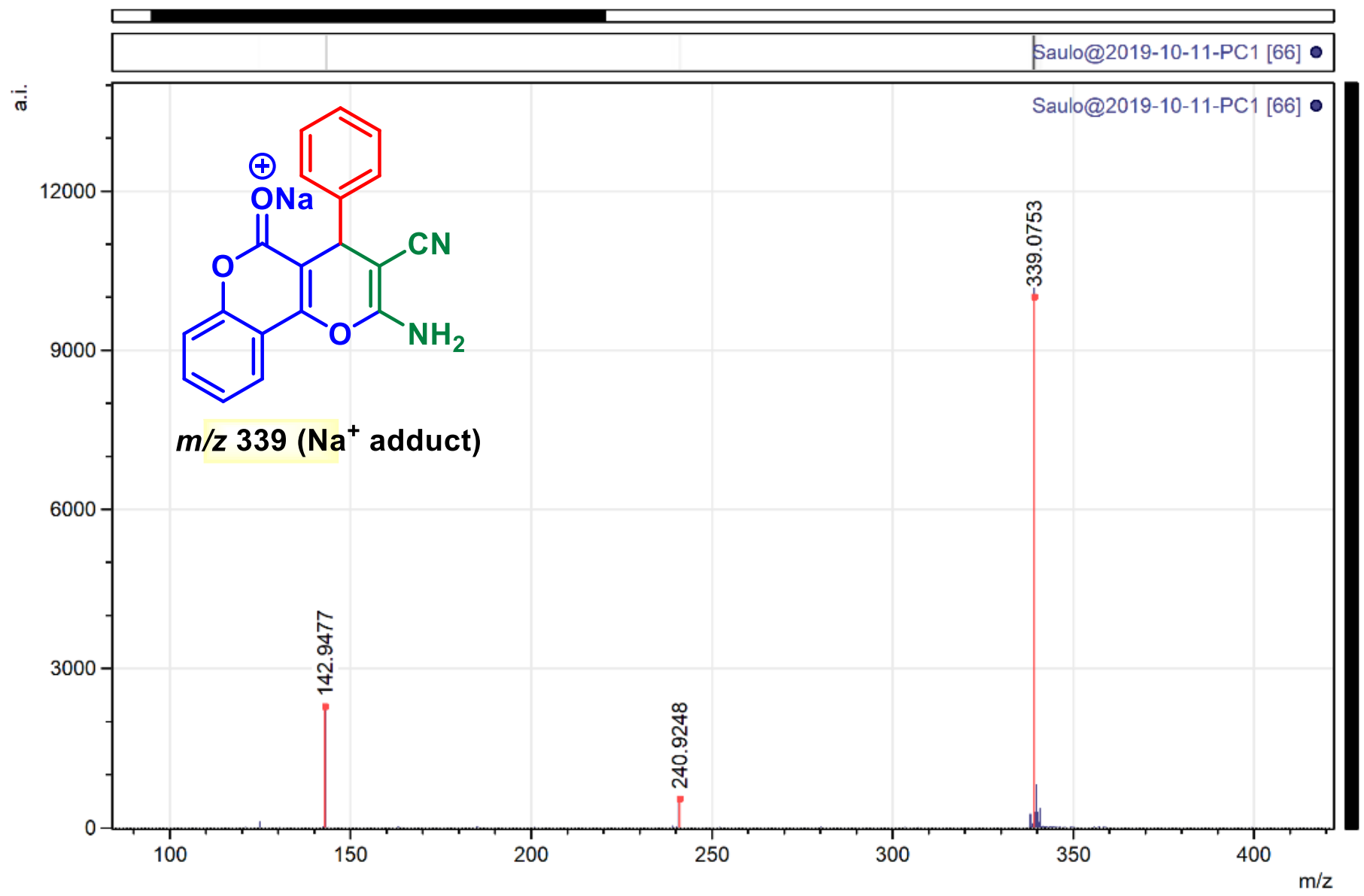

Figure S26. ESI(+)-MS/MS of the signal of *m/z* 339.

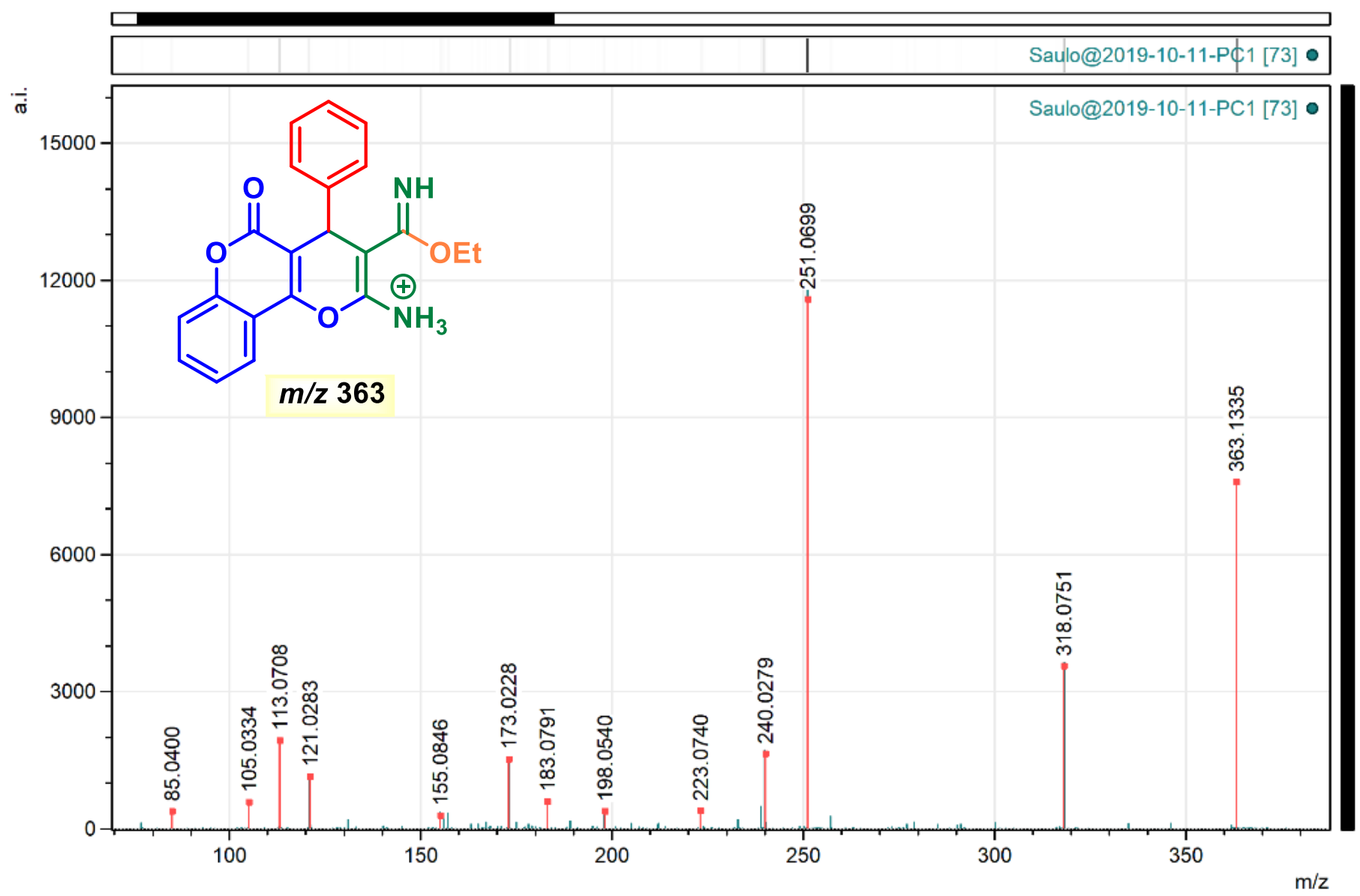

Figure S27. ESI(+)-MS/MS of the signal of *m/z* 363.

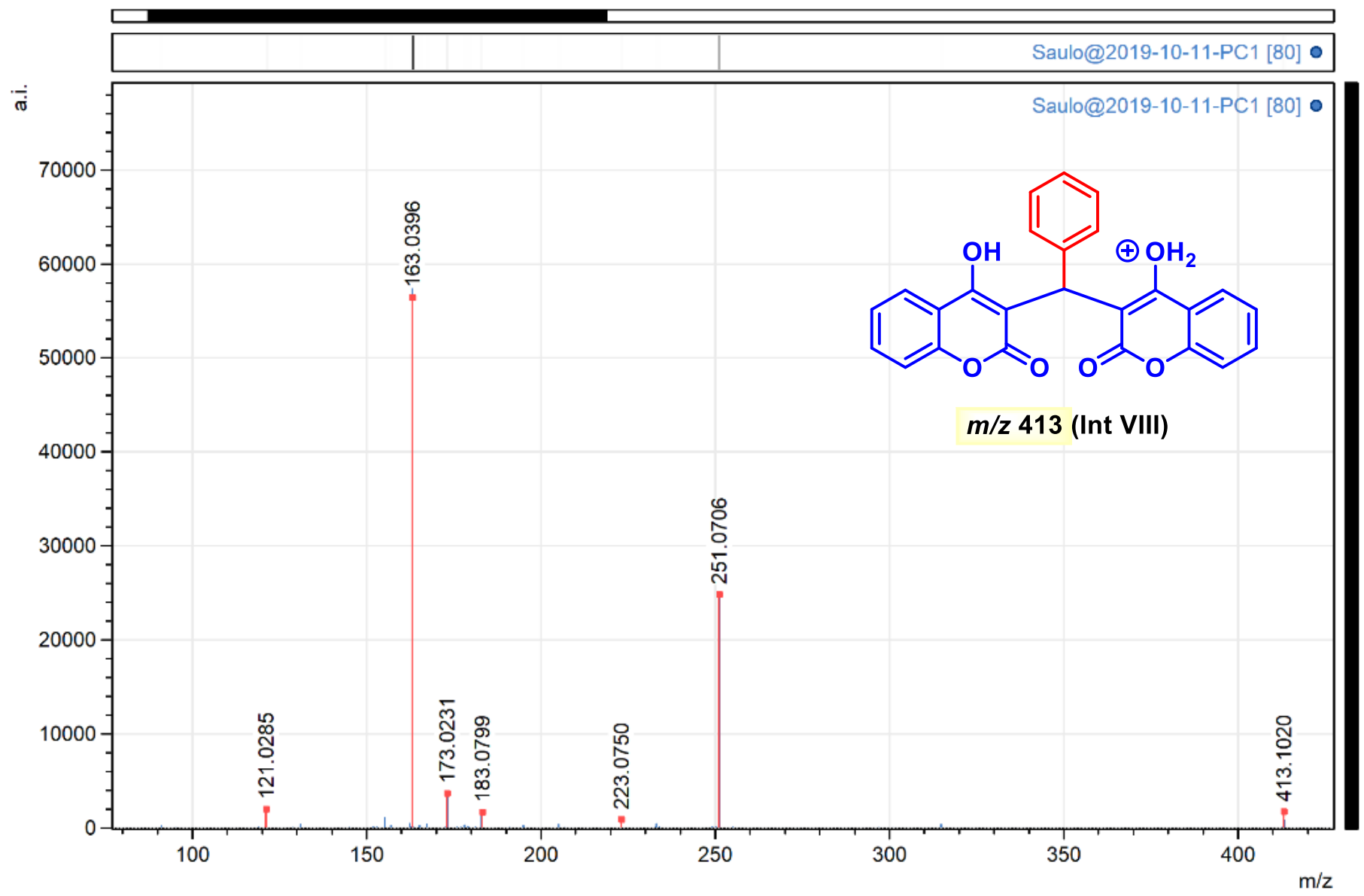

Figure S28. ESI(+)-MS/MS of the signal of *m/z* 413.

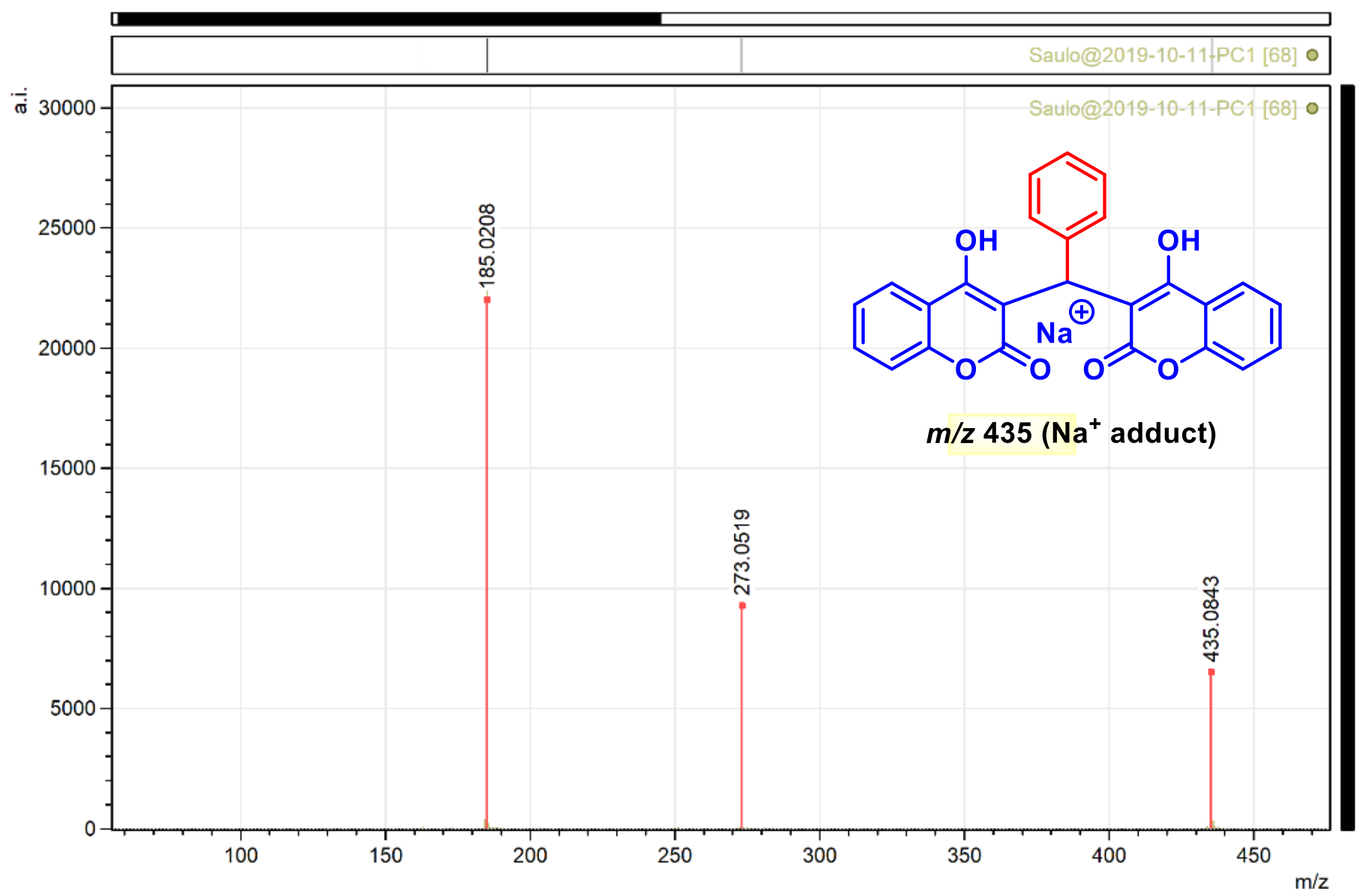

Figure S29. ESI(+)-MS/MS of the signal of *m/z* 435.

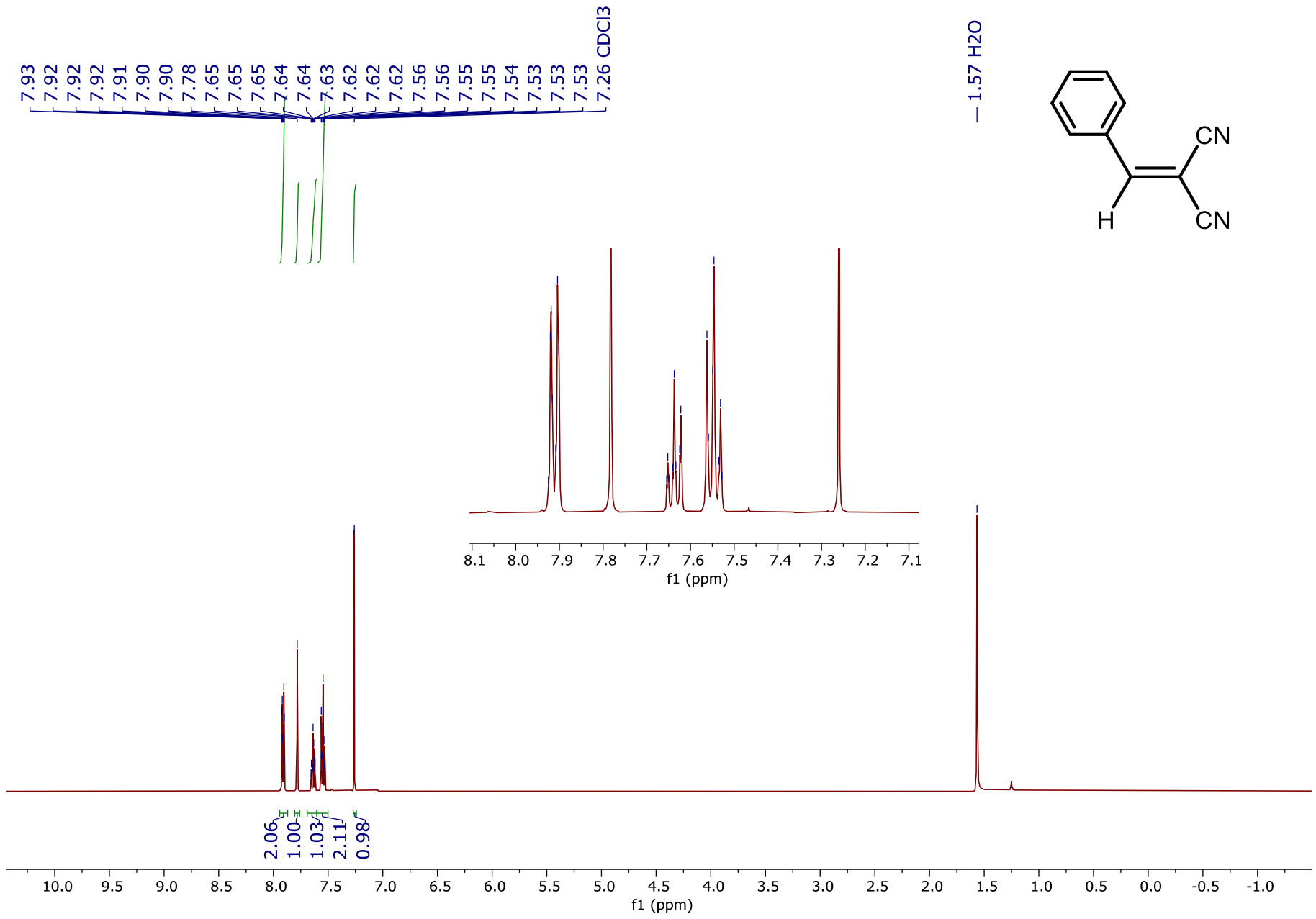

Figure S30. ^{1}H NMR spectrum (400 MHz, $CDCl_3$) of **Int II**.

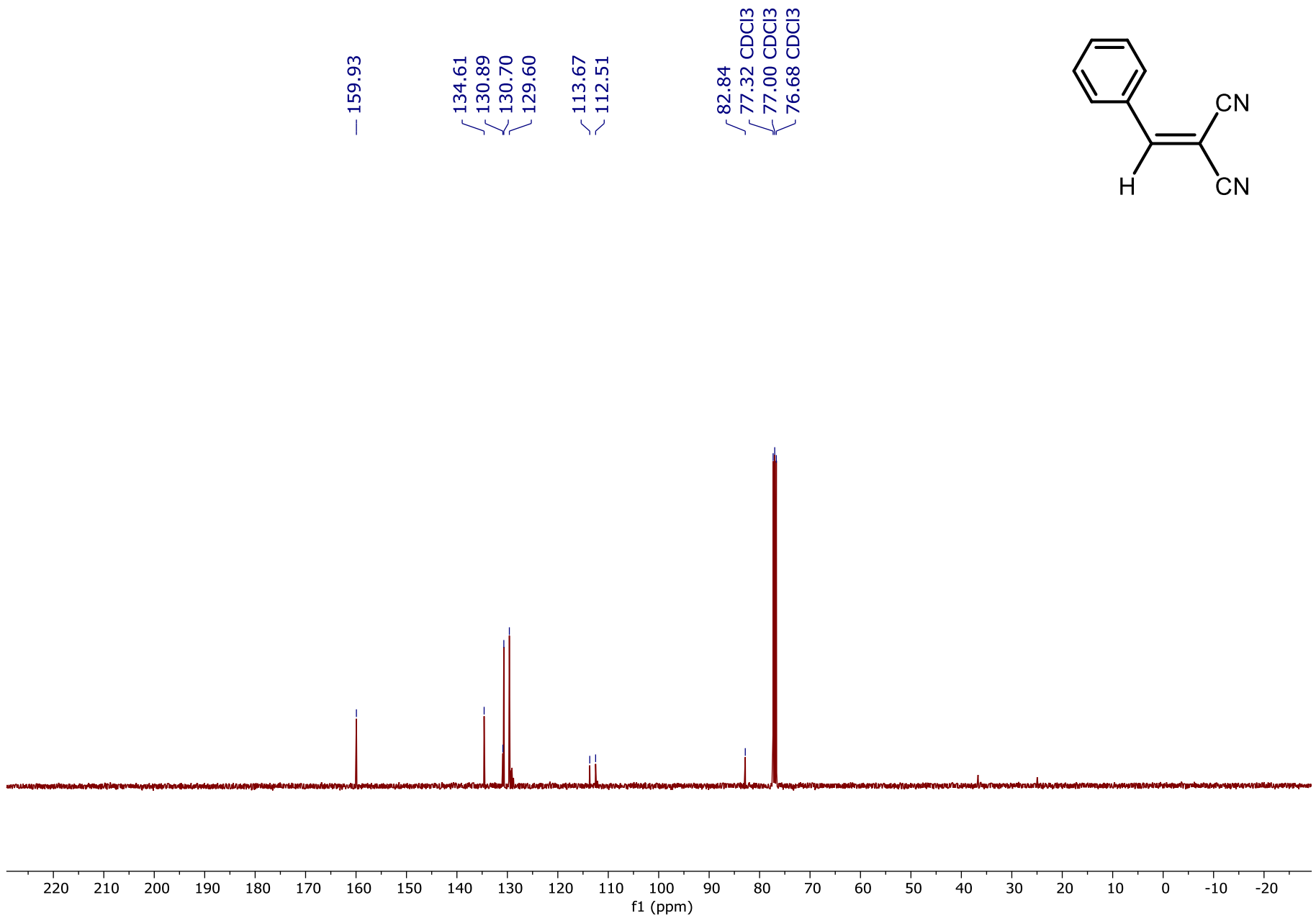

Figure S31. ^{13}C{^{1}H} NMR spectrum (100 MHz, $CDCl_3$) of **Int II**.

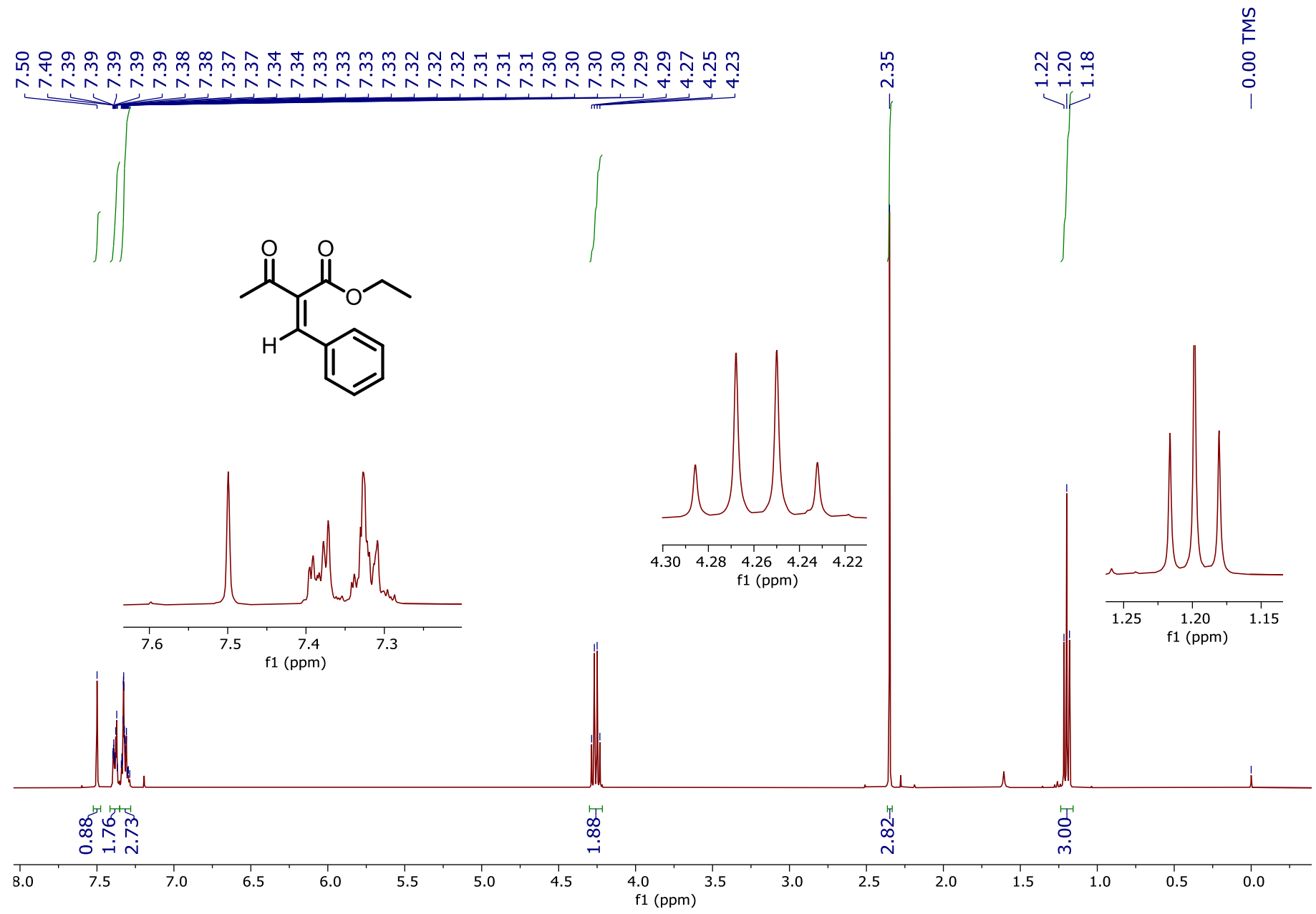

Figure S32. ^{1}H NMR spectrum (400 MHz, $CDCl_3$) of the intermediate (*Z*)-ethyl acetobenzylidene acetate (**Int IV**).

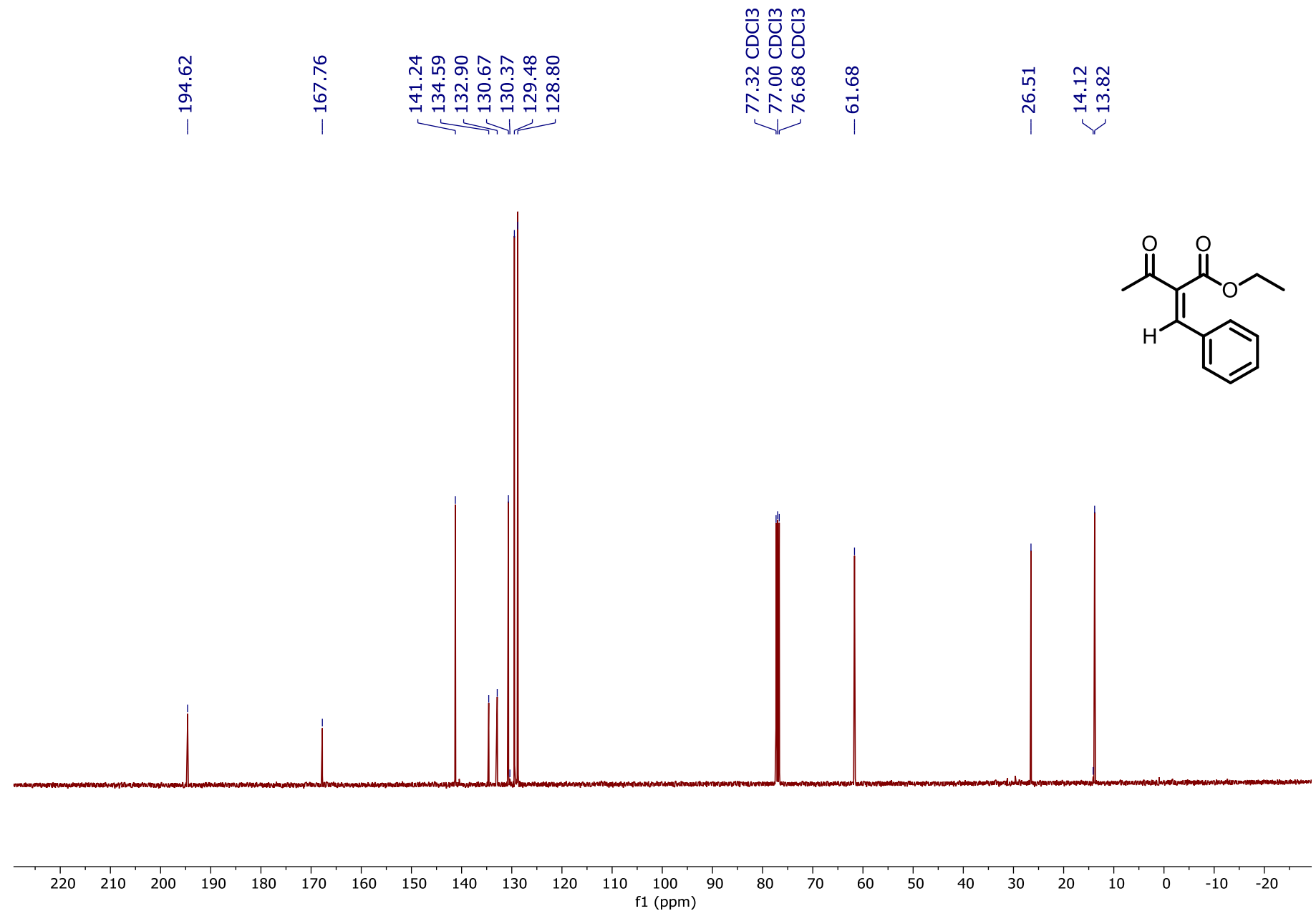

Figure S33. $^{13}C\{^{1}H\}$ NMR spectrum (100 MHz, $CDCl_3$) of the intermediate (*Z*)-ethyl acetobenzylidene acetate (**Int IV**).

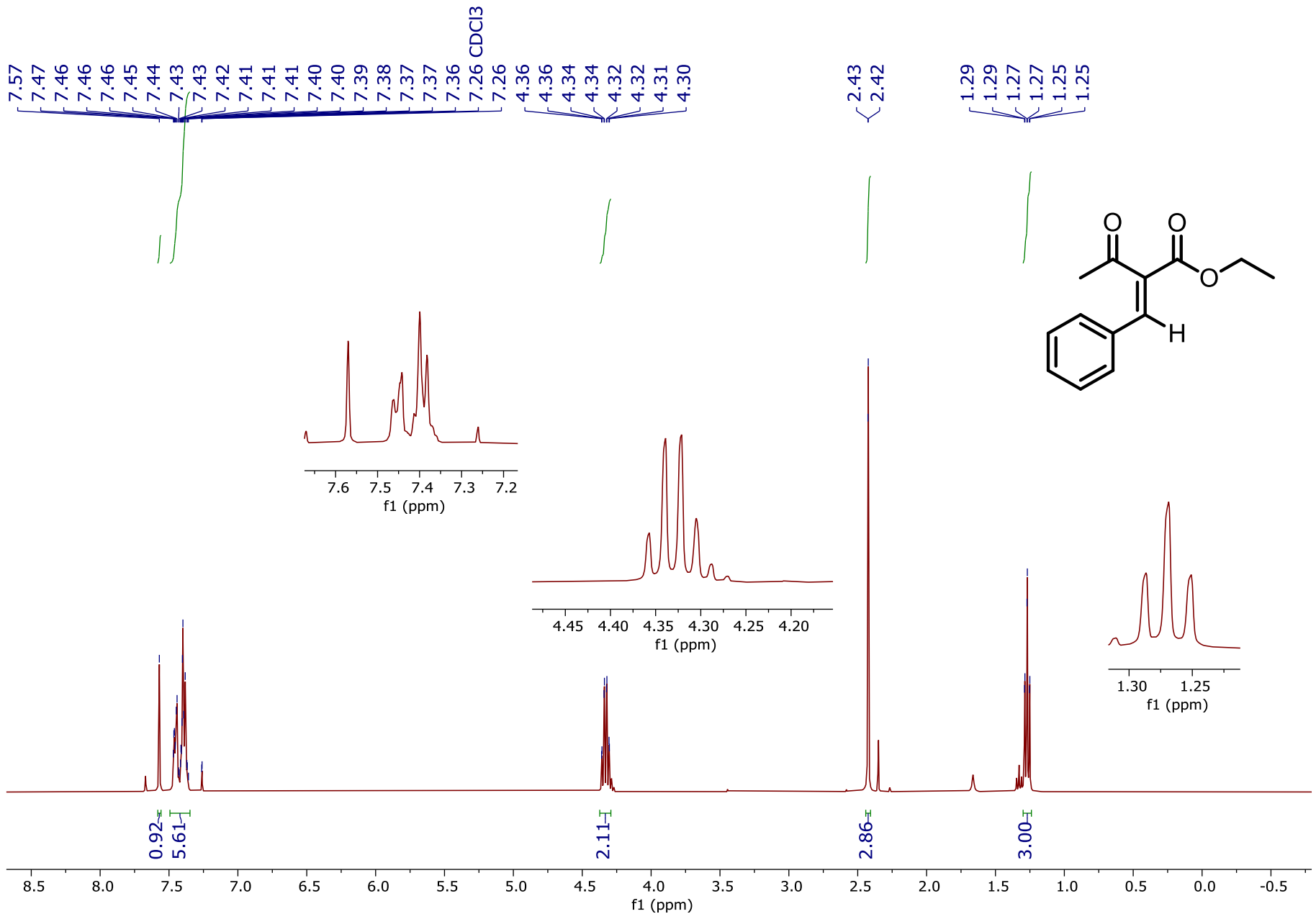

Figure S34. ^{1}H NMR spectrum (400 MHz, $CDCl_3$) of the intermediate (*E*)-ethyl acetobenzylidene acetate (**Int IV**).

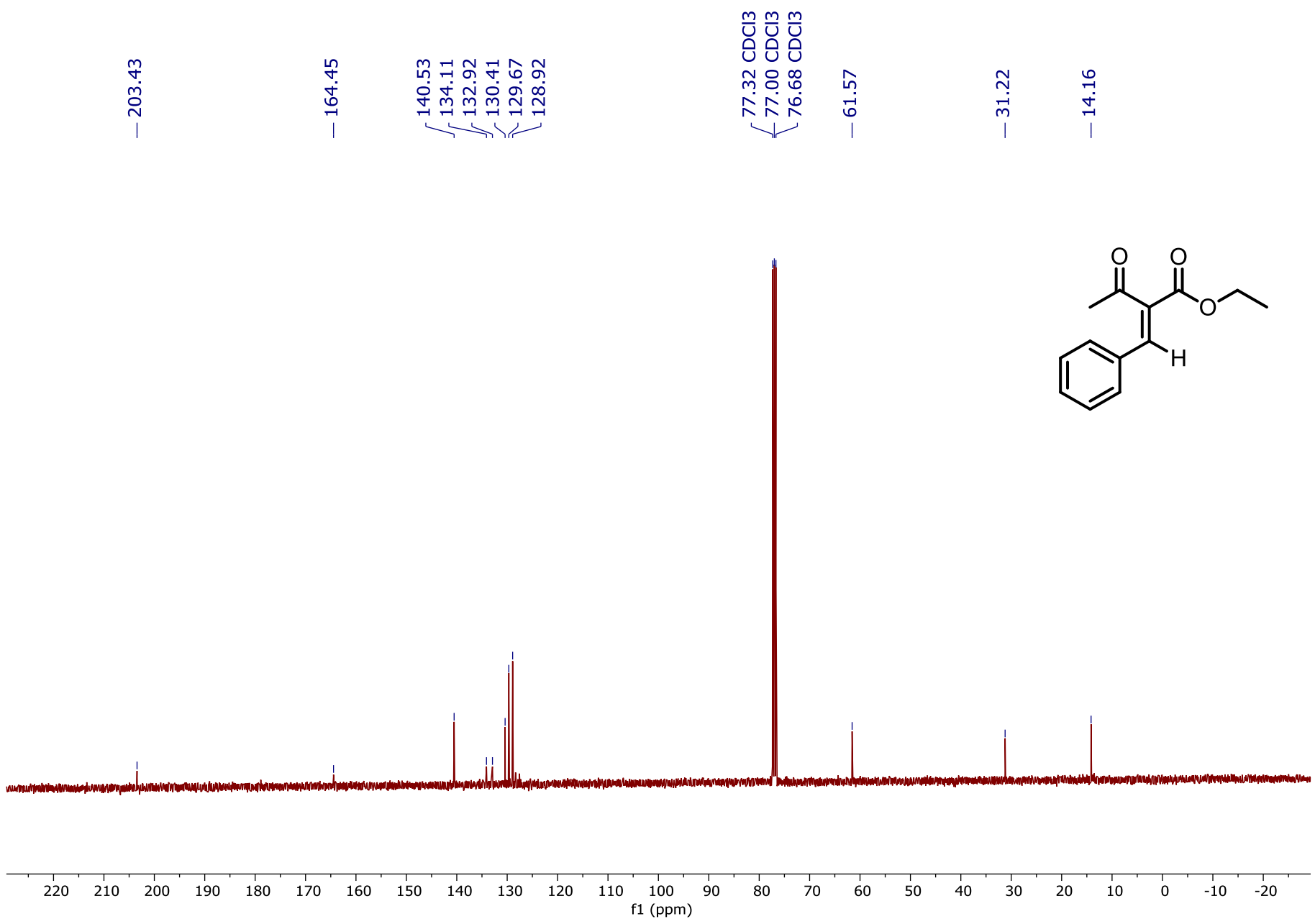

Figure S35. $^{13}C\{^{1}H\}$ NMR spectrum (100 MHz, $CDCl_3$) of the intermediate (*E*)-ethyl acetobenzylidene acetate (**Int IV**).

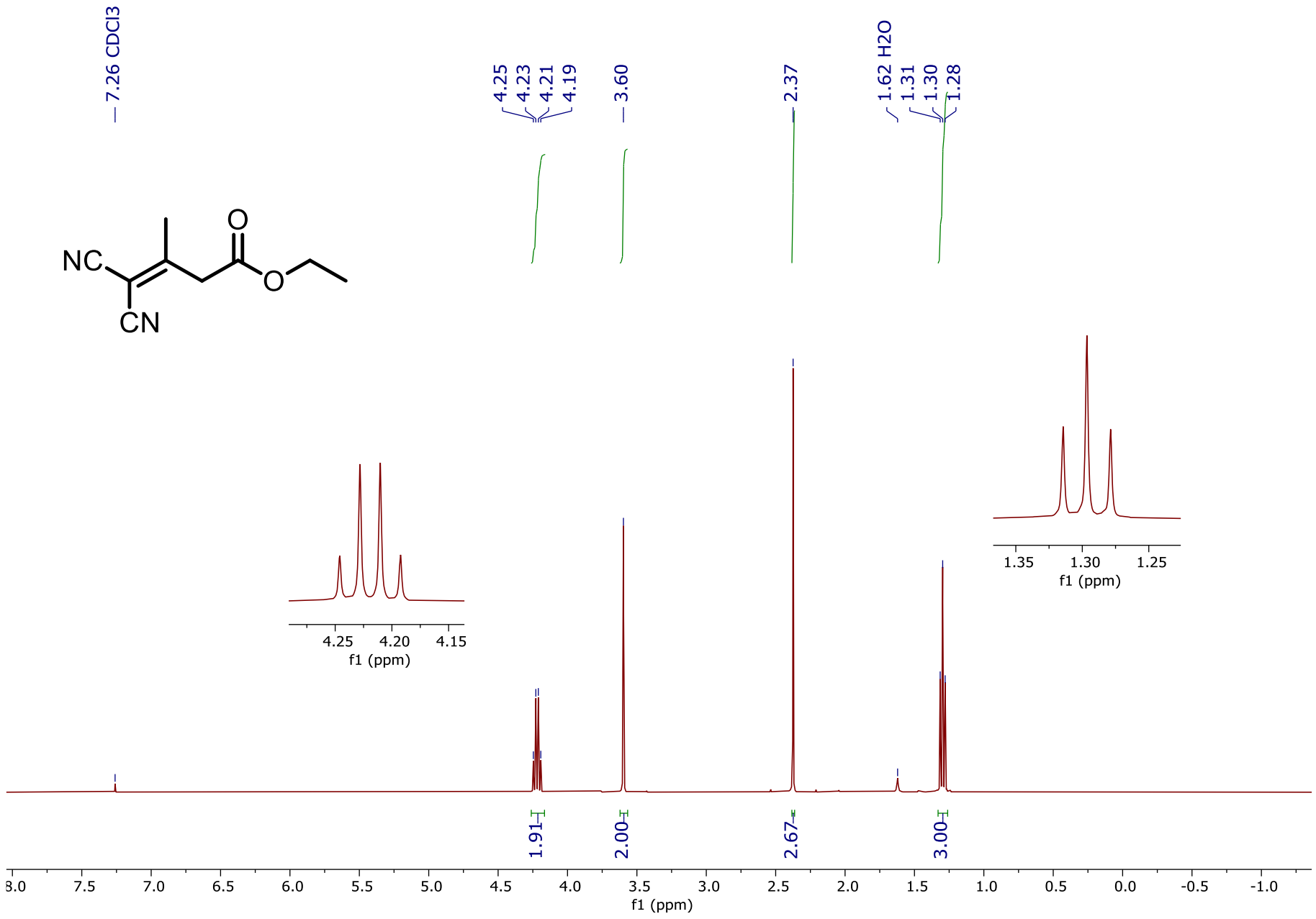

Figure S36. ^{1}H NMR spectrum (400 MHz, $CDCl_3$) of **Int V**.

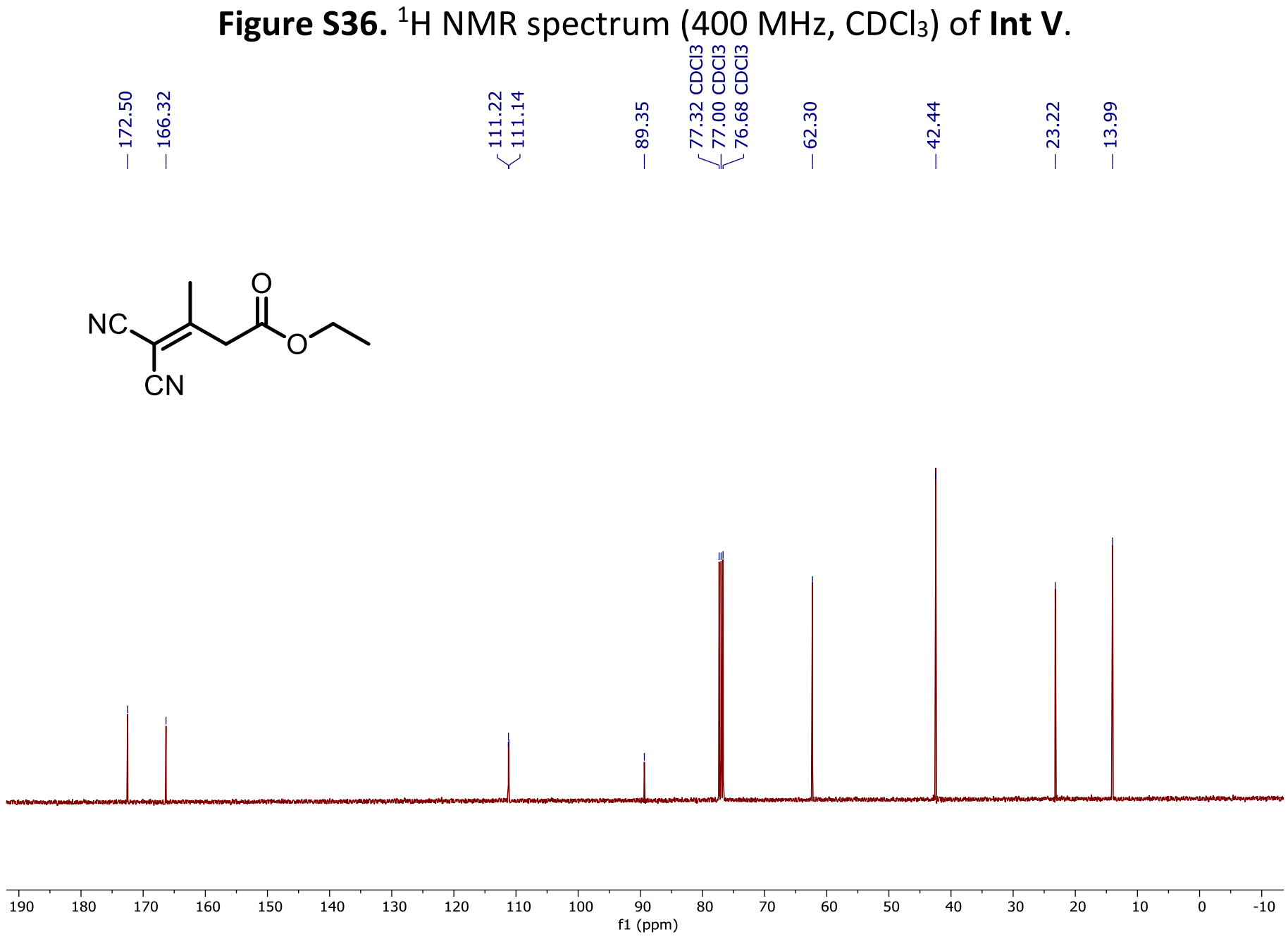

Figure S37. $^{13}C\{^{1}H\}$ NMR spectrum (100 MHz, $CDCl_3$) of **Int V**.

Spectral data for compounds ACP 4a-4x.

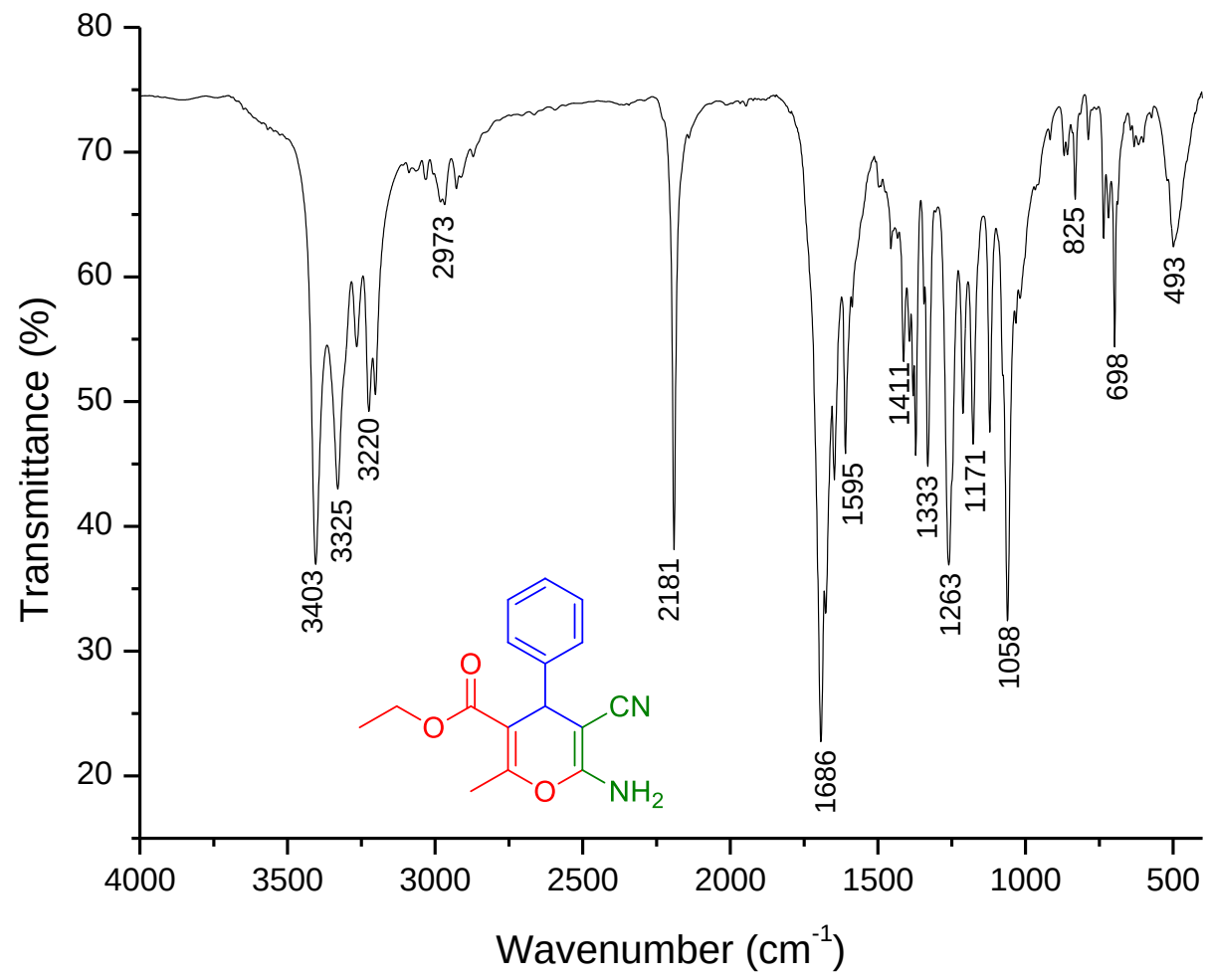

Figure S38. FT-IR (KBr) spectrum of compound **4a**.

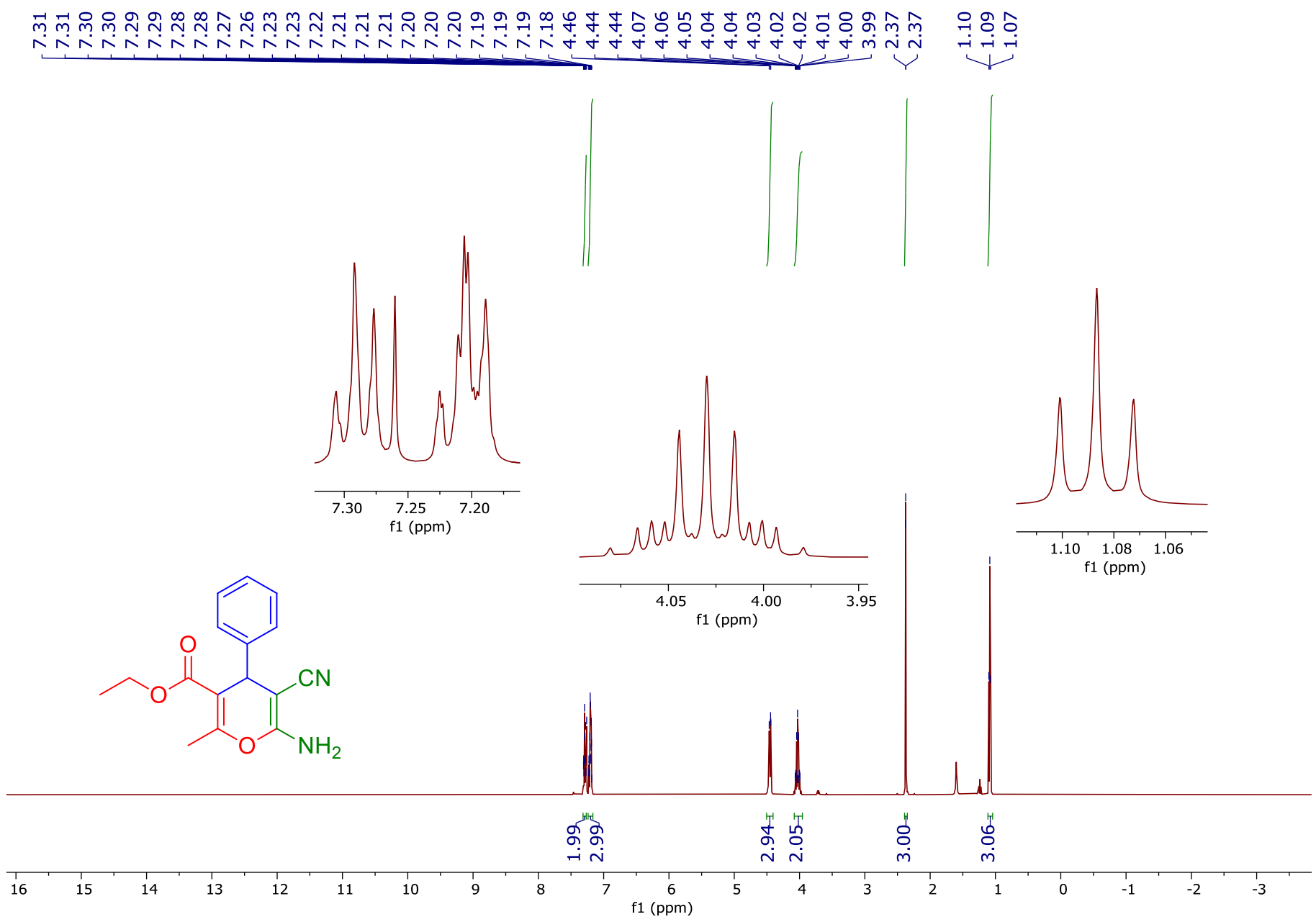

Figure S39. ^{1}H NMR spectrum (500 MHz, $CDCl_3$) of **4a**.

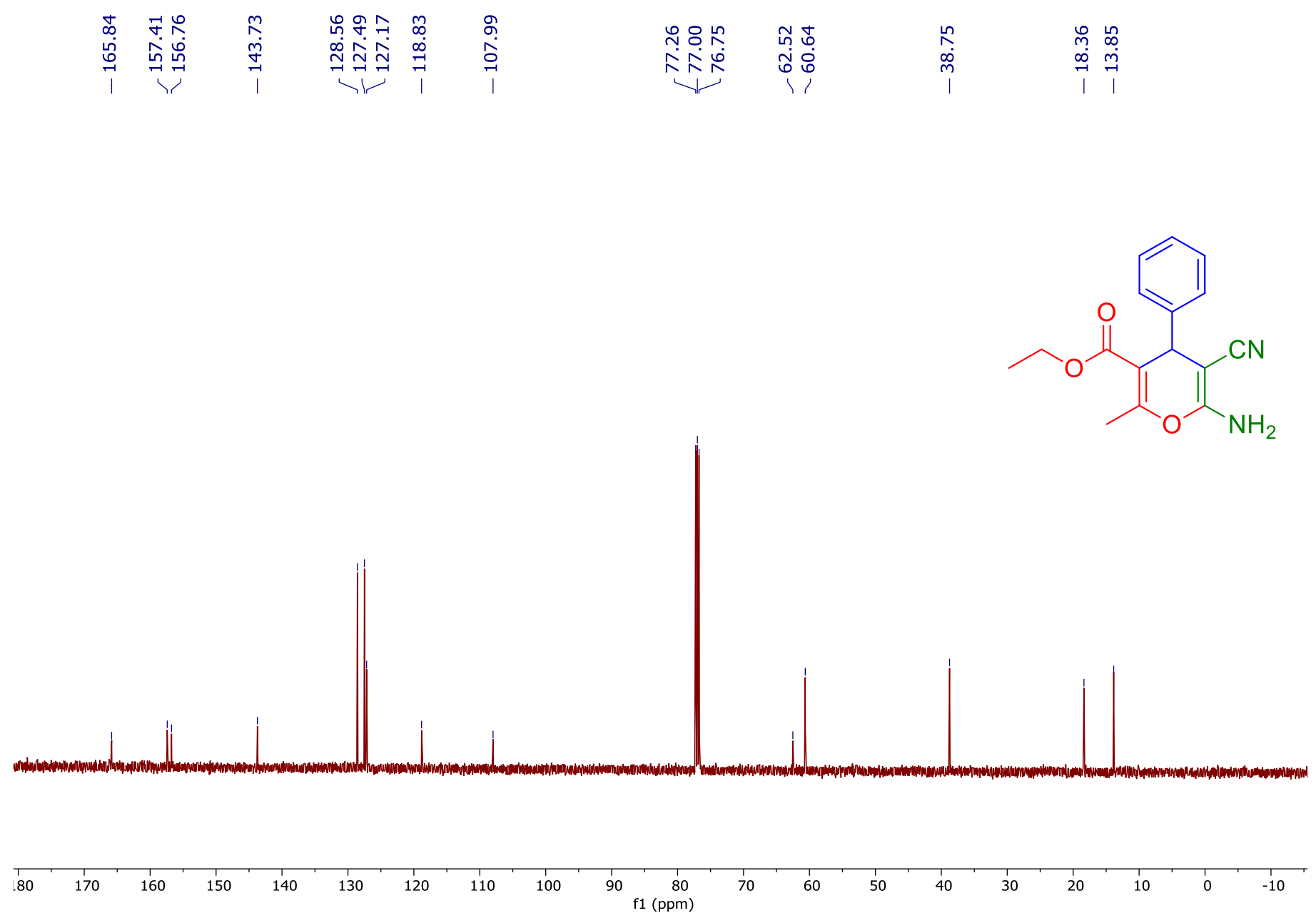

Figure S40. ^{13}C {^{1}H} NMR spectrum (125 MHz, $CDCl_3$) of **4a**.

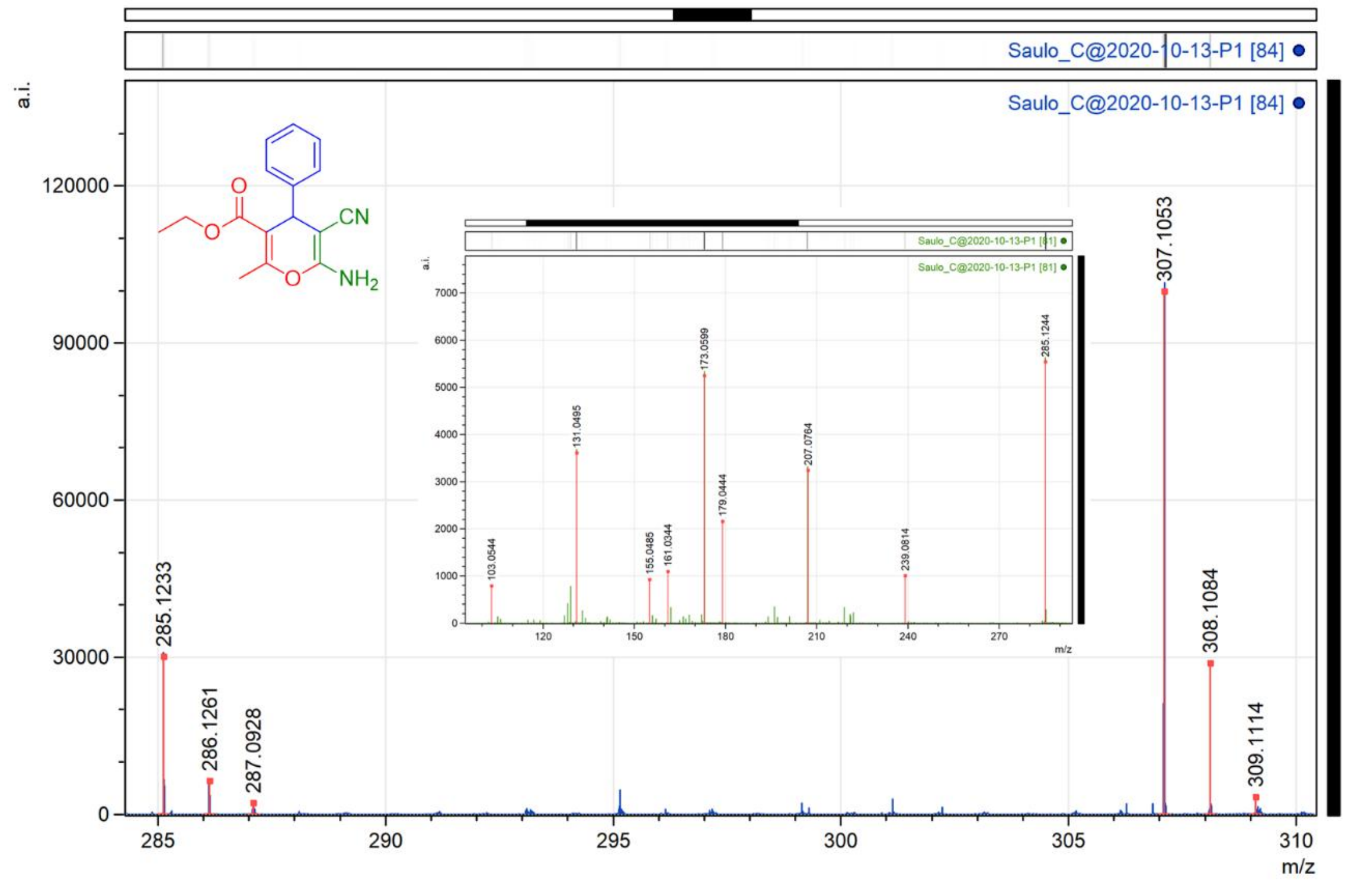

Figure S41. High-resolution ESI(+)-MS of **4a** showing its isotopic pattern. The inset is the ESI(+)-MS/MS from the $[M+H]^+$ isotopologue.

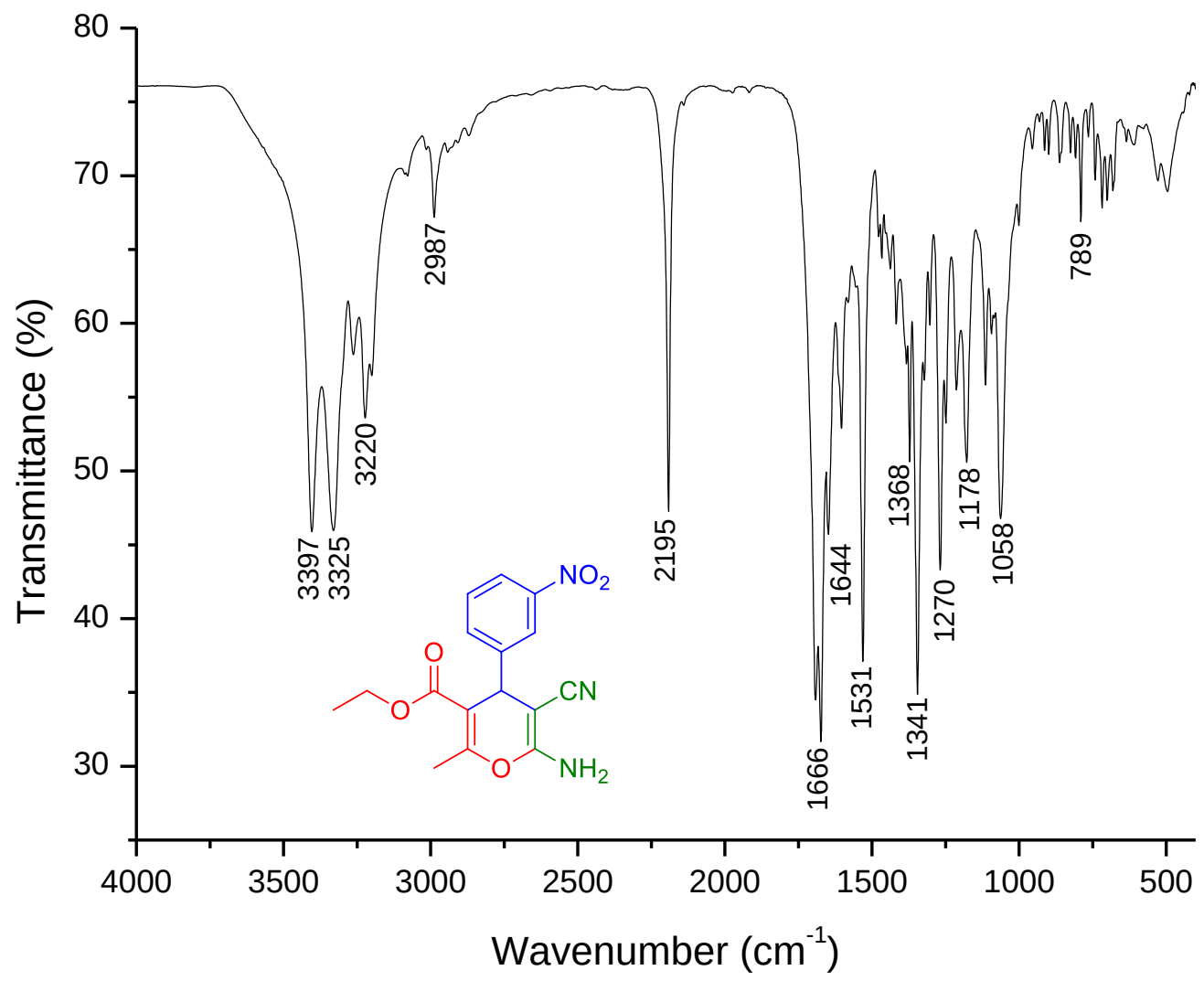

Figure S42. FT-IR (KBr) spectrum of compound **4b**.

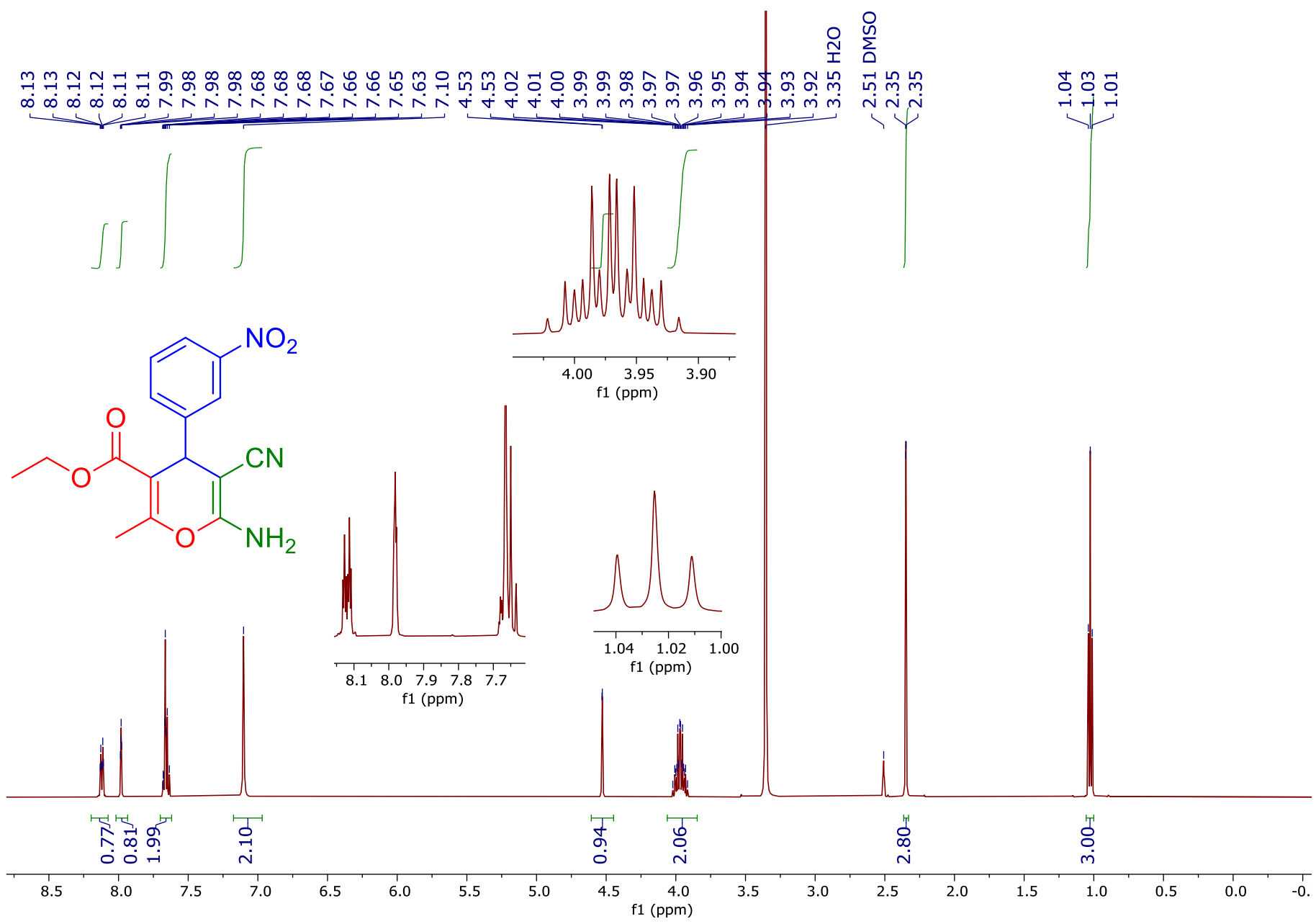

Figure S43. ^{1}H NMR spectrum (500 MHz, $CDCl_3$) of **4b**.

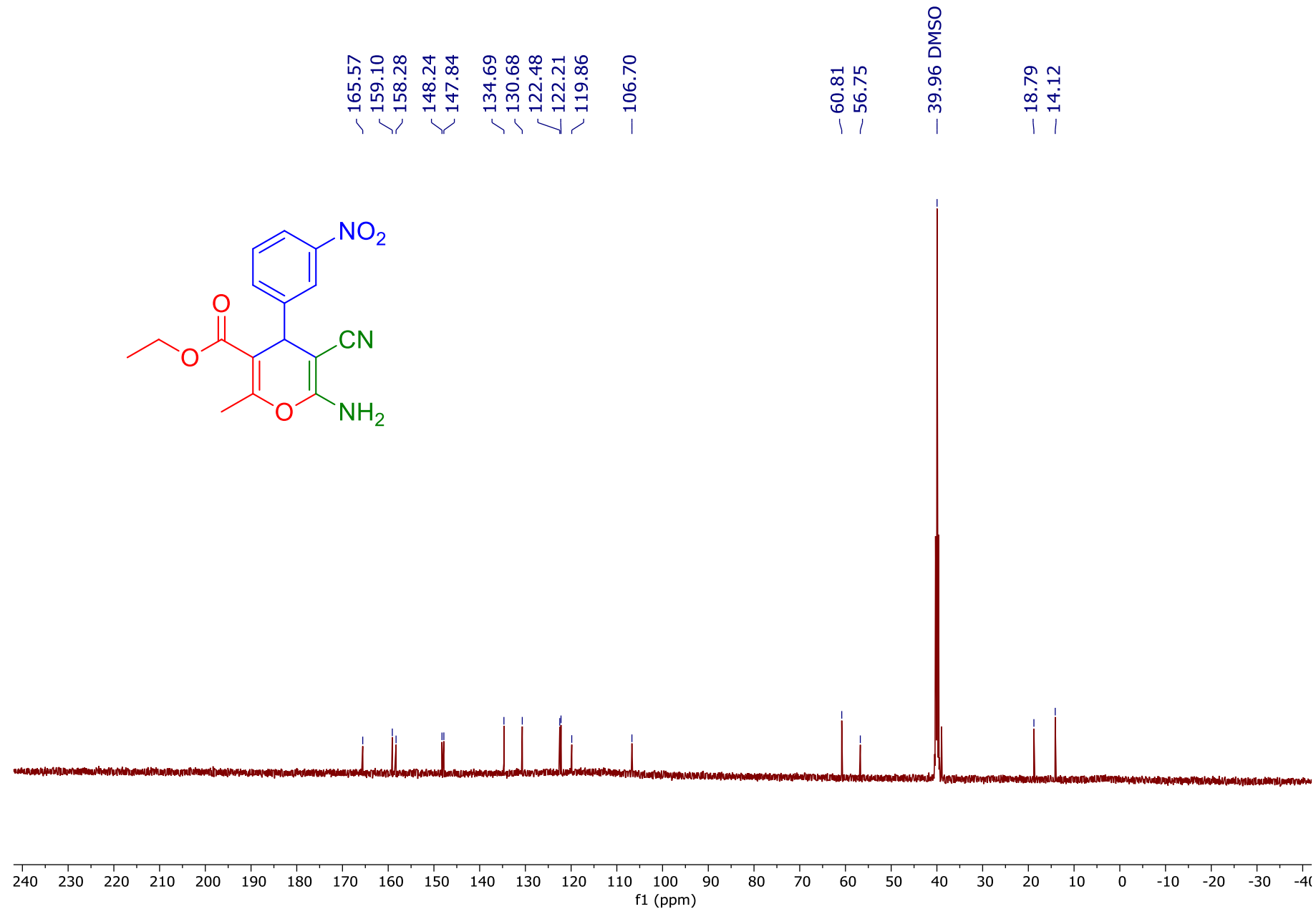

Figure S44. $^{13}C\ \{^{1}H\}$ NMR spectrum (125 MHz, DMSO-d$_6$) of **4b**.

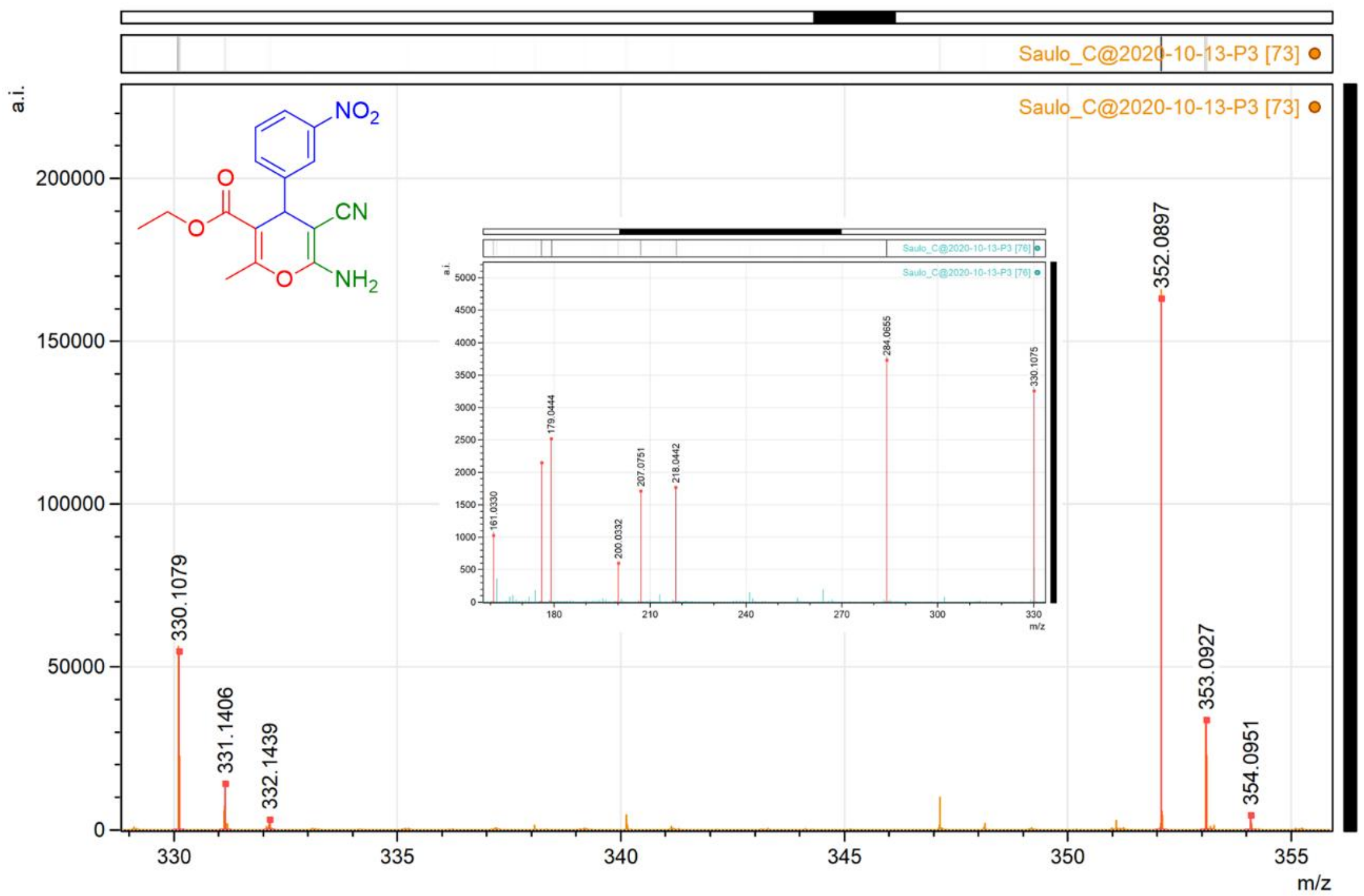

Figure S45. High-resolution ESI(+)-MS of **4b** showing its isotopic pattern. The inset is the ESI(+)-MS/MS from the [M+H]$^+$ isotopologue.

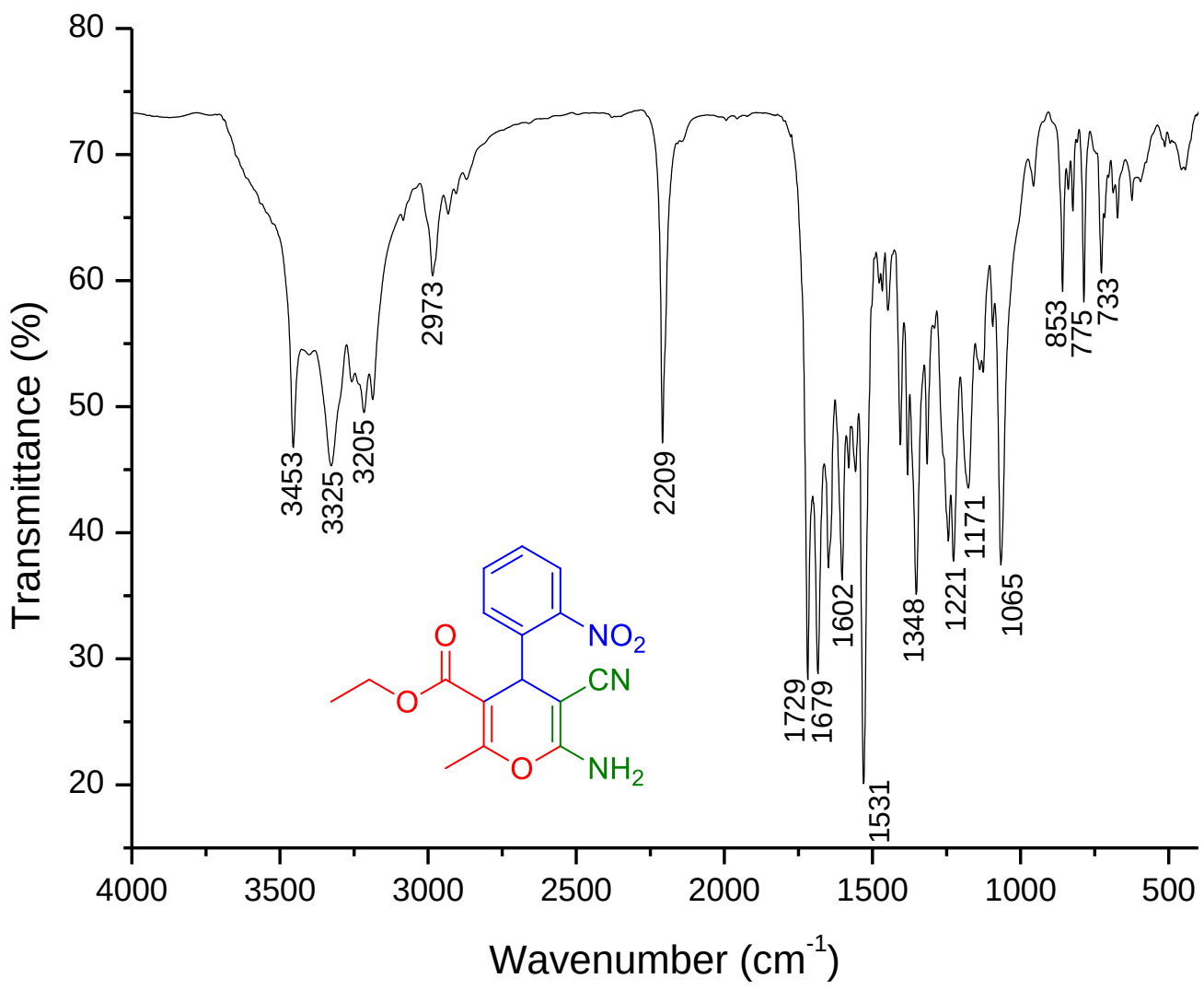

Figure S46. FT-IR (KBr) spectrum of compound **4c**.

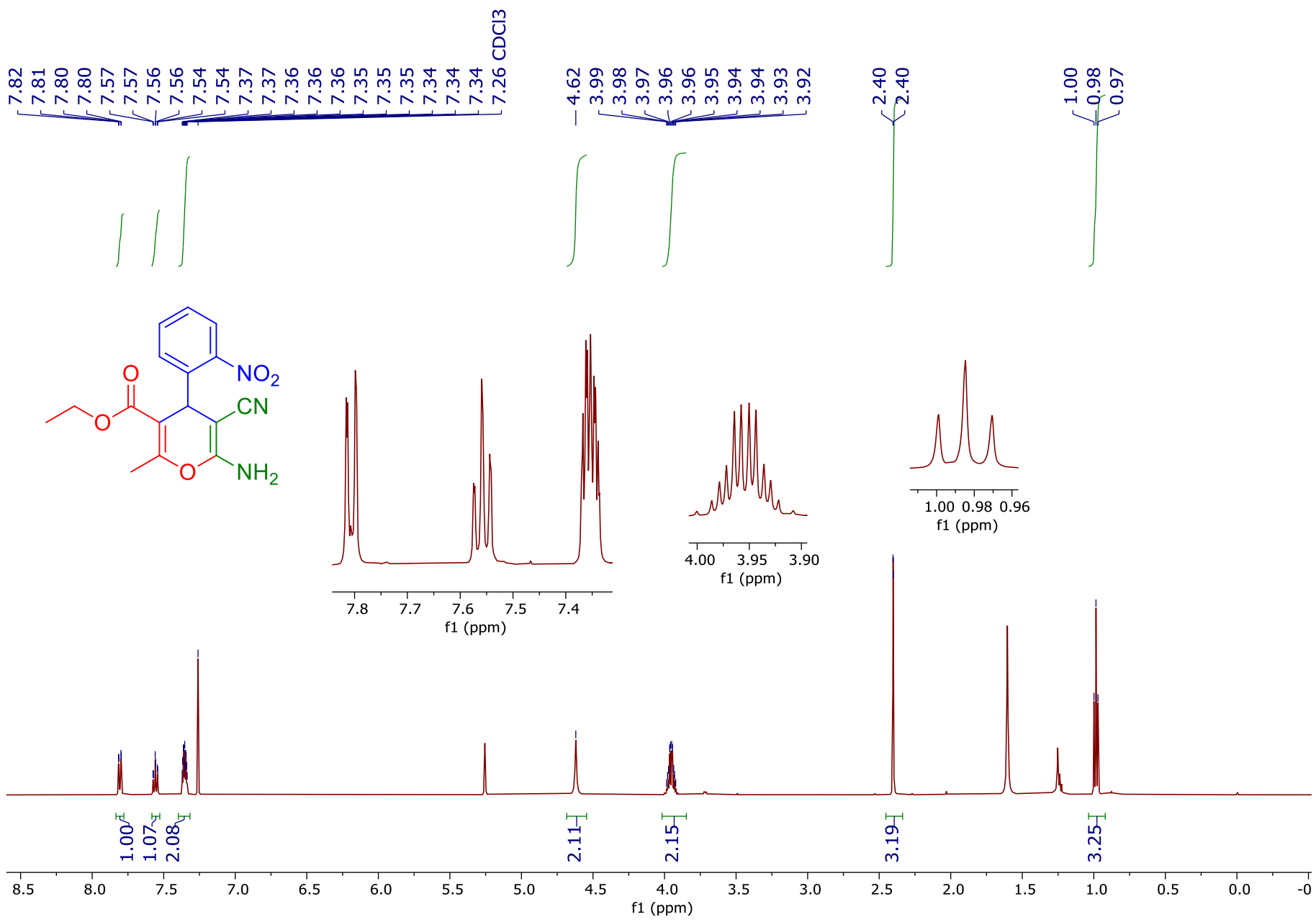

Figure S47. ^{1}H NMR spectrum (500 MHz, CDCl₃) of **4c**.

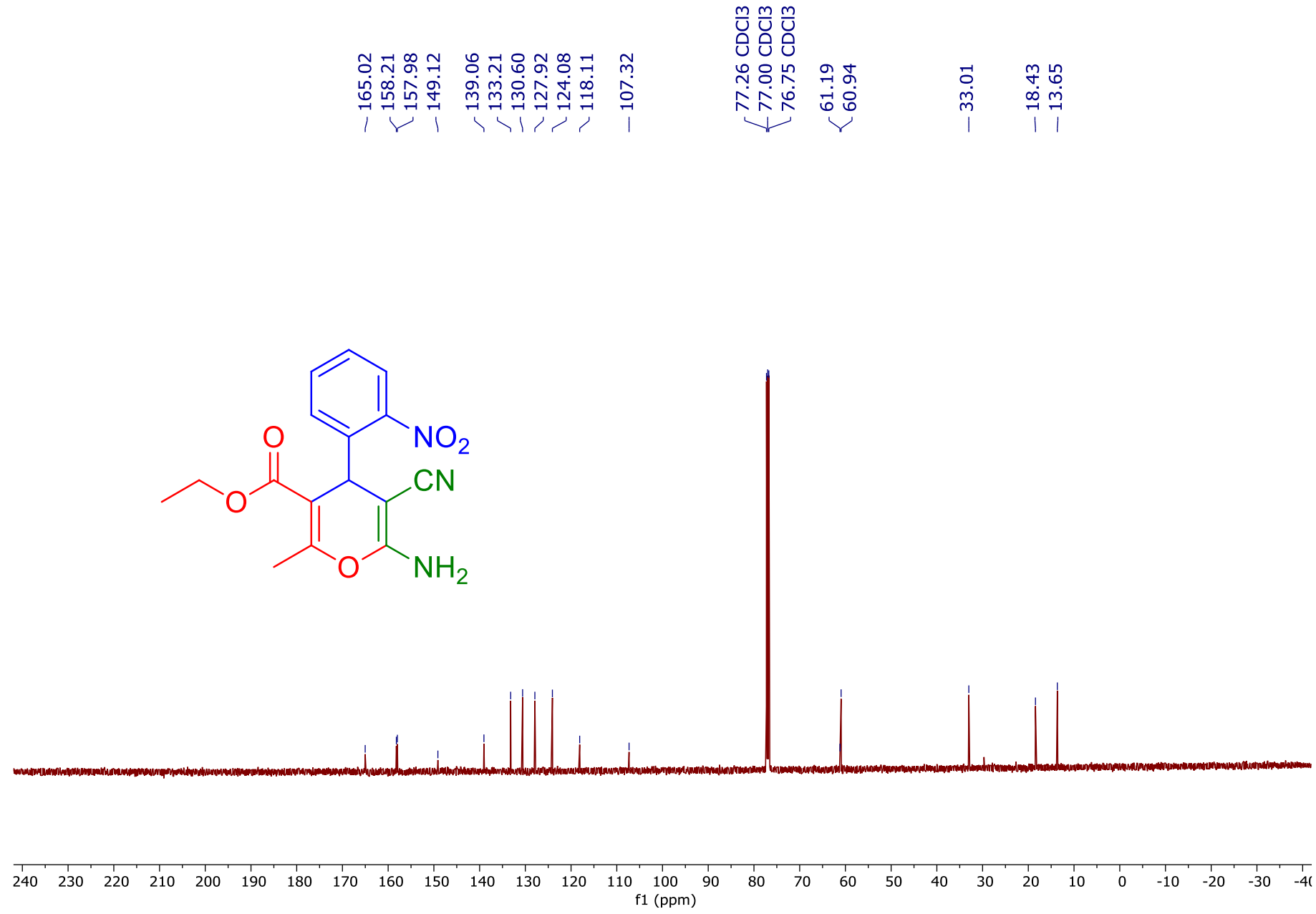

Figure S48. $^{13}C\{^1H\}$ NMR spectrum (125 MHz, $CDCl_3$) of **4c**. One carbon signal overlaps with the solvent signals.

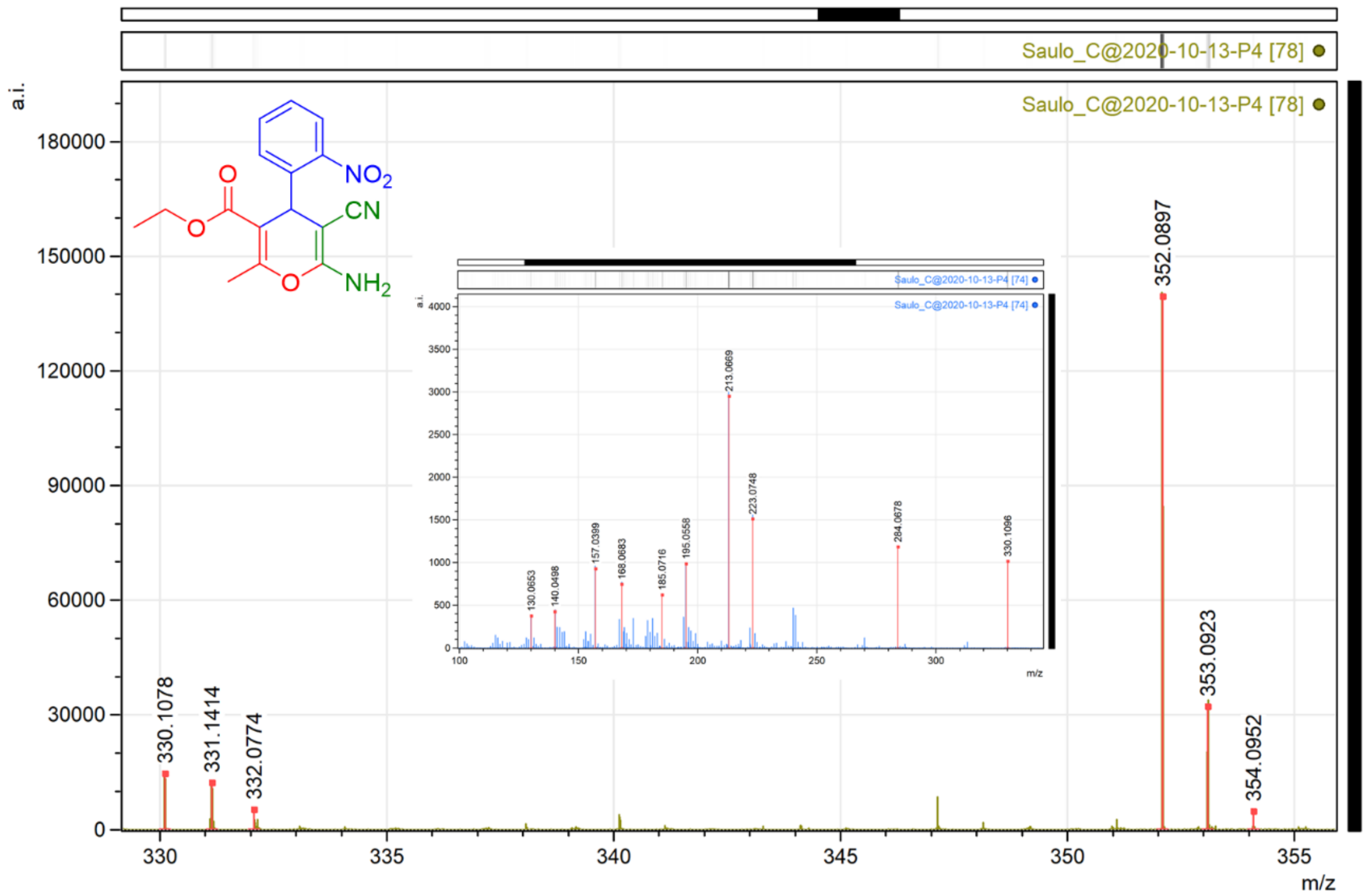

Figure S49. High-resolution ESI(+)-MS of **4c** showing its isotopic pattern. The inset is the ESI(+)-MS/MS from the $[M+H]^+$ isotopologue.

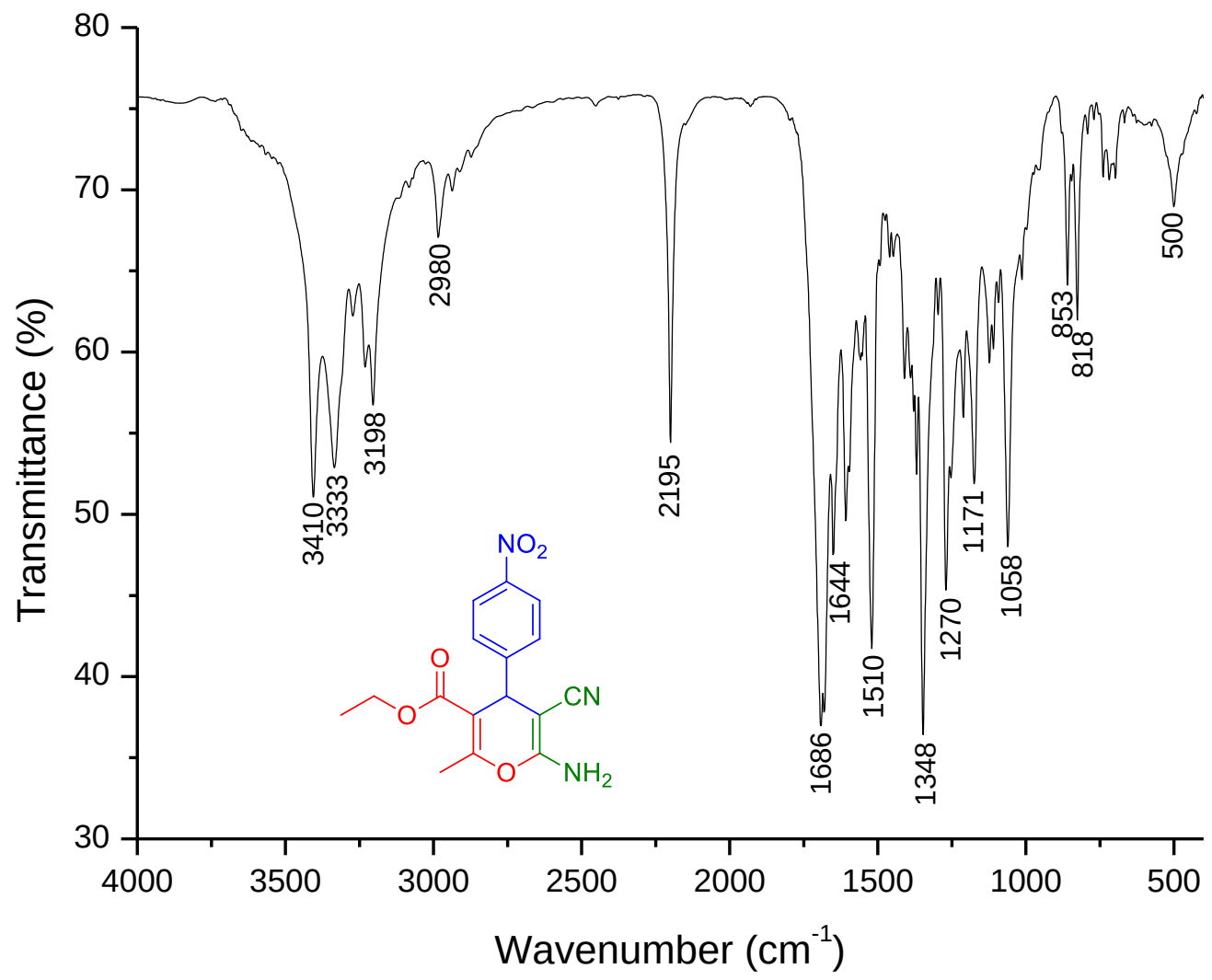

Figure S50. FT-IR (KBr) spectrum of compound **4d**.

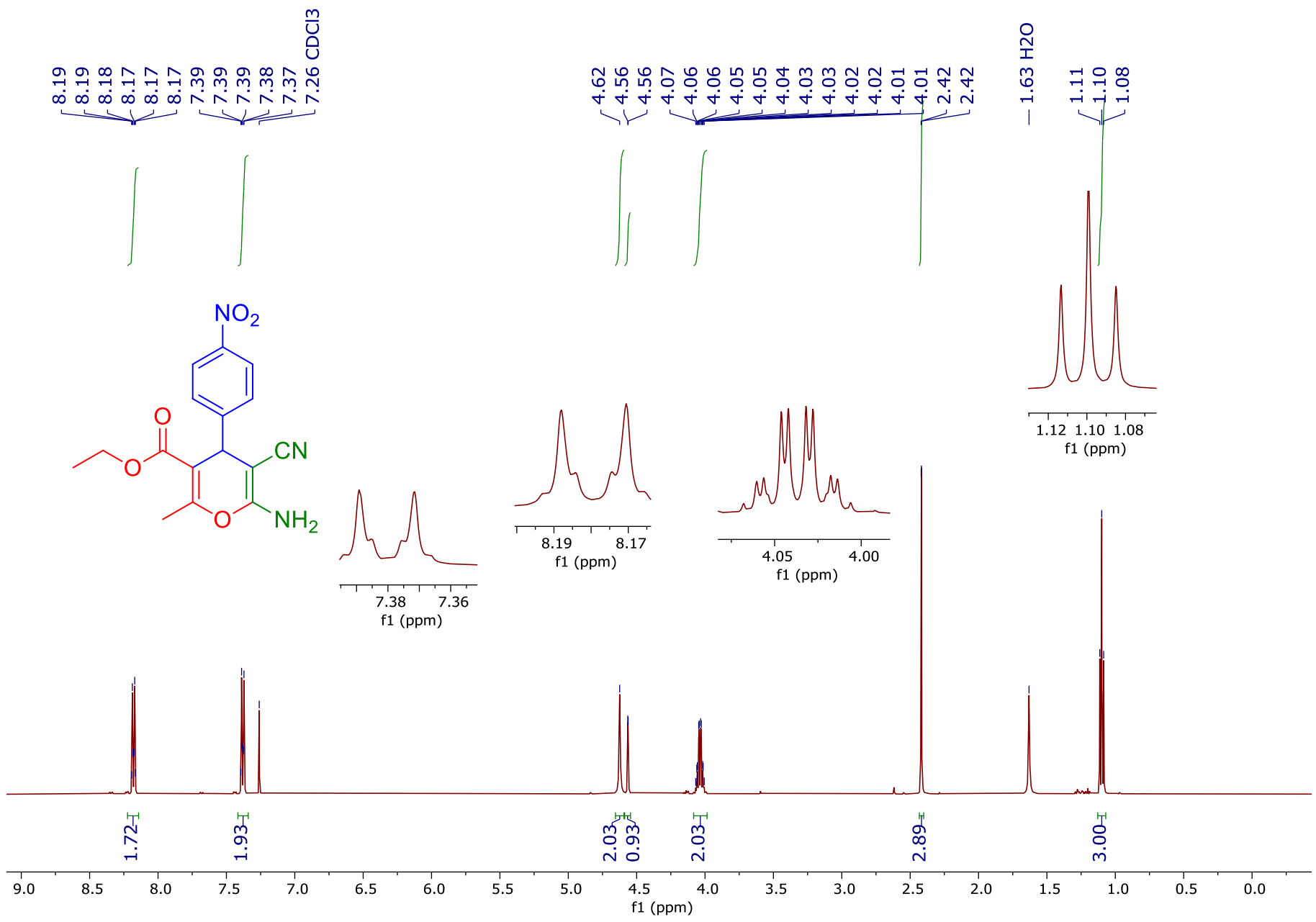

Figure S51. ¹H NMR spectrum (500 MHz, $CDCl_3$) of **4d**.

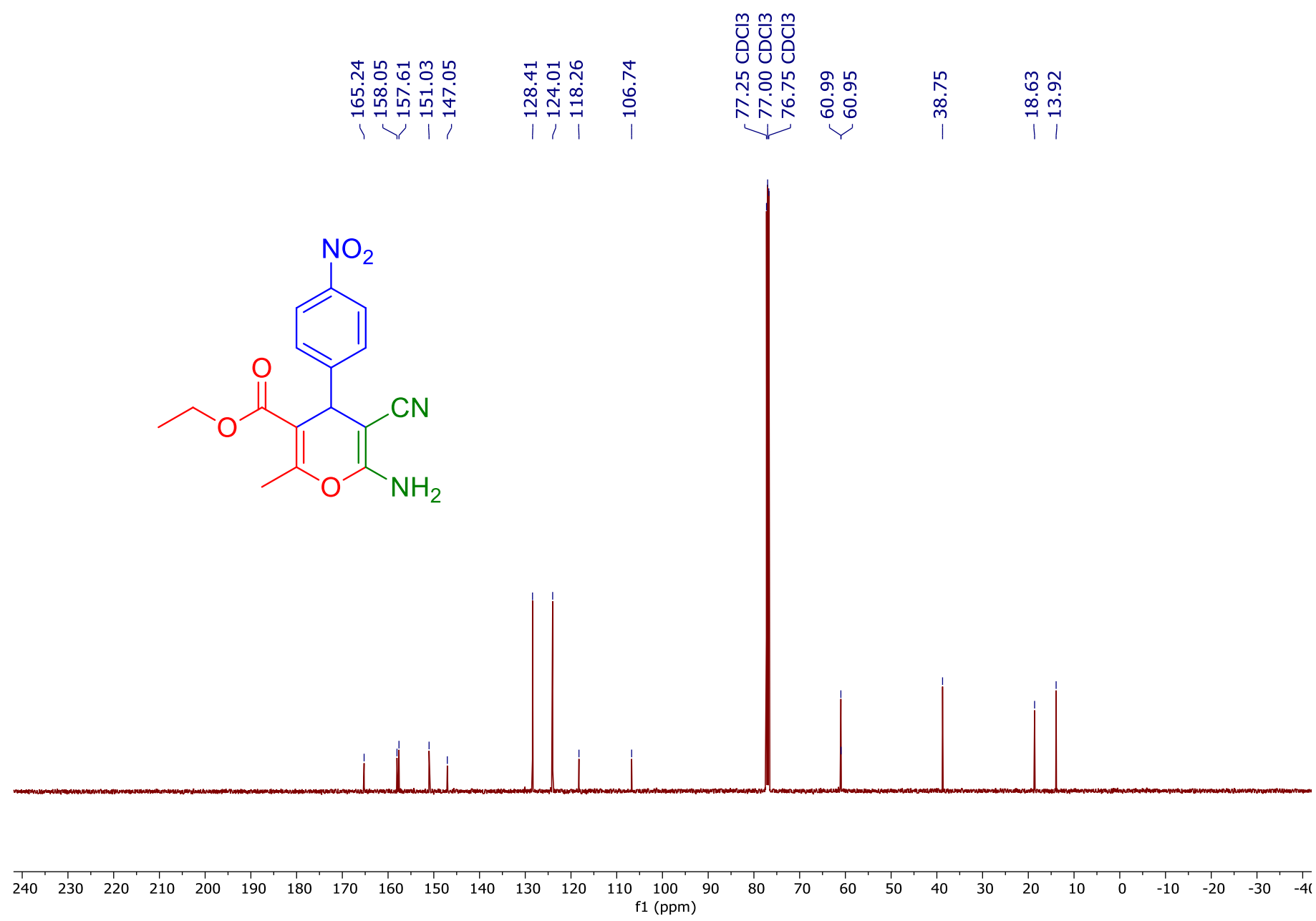

Figure S52. ^{13}C {^{1}H} NMR spectrum (125 MHz, $CDCl_3$) of **4d**.

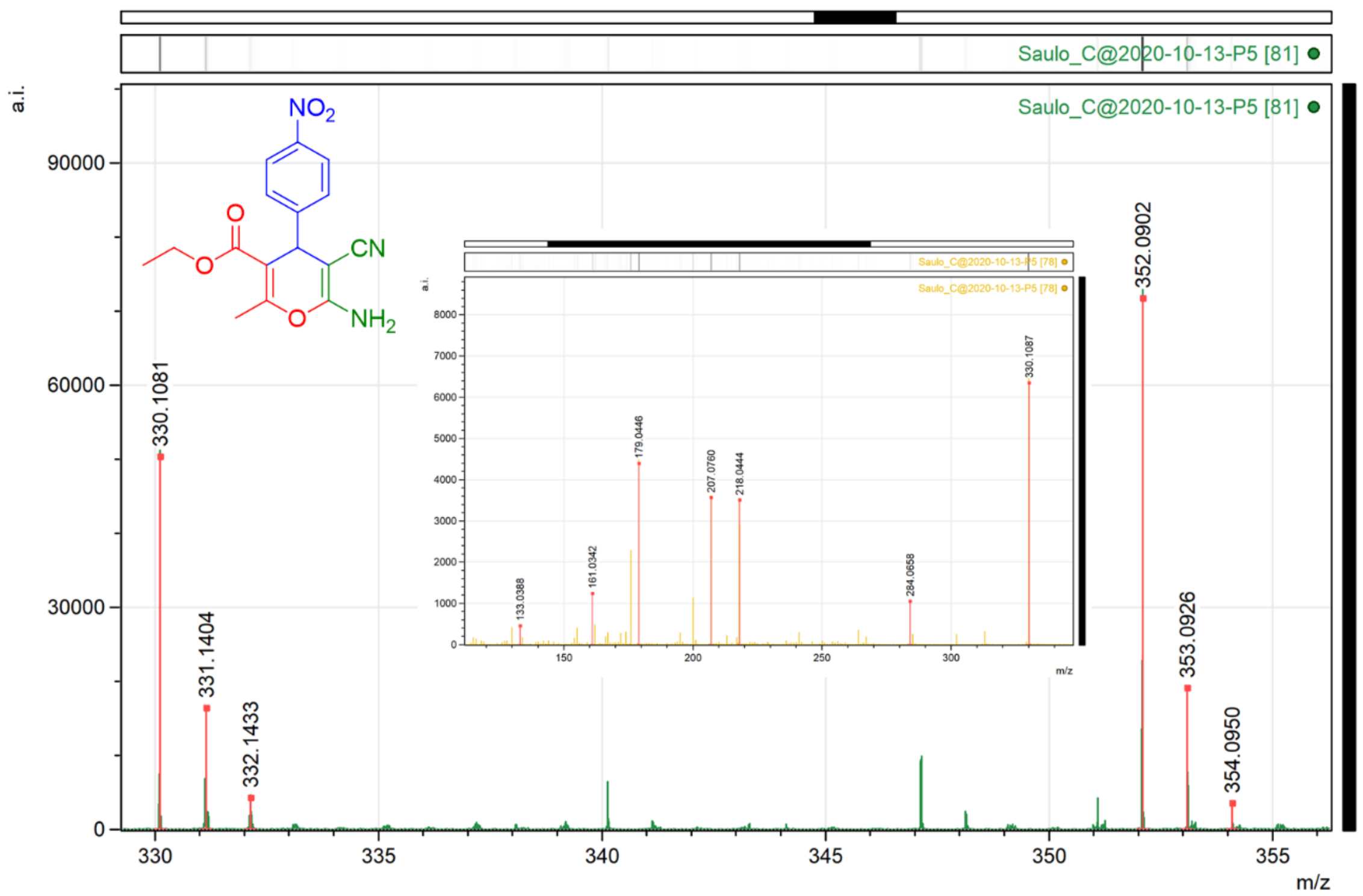

Figure S53. High-resolution ESI(+)-MS of **4d** showing its isotopic pattern. The inset is the ESI(+)-MS/MS from the $[M+H]^+$ isotopologue.

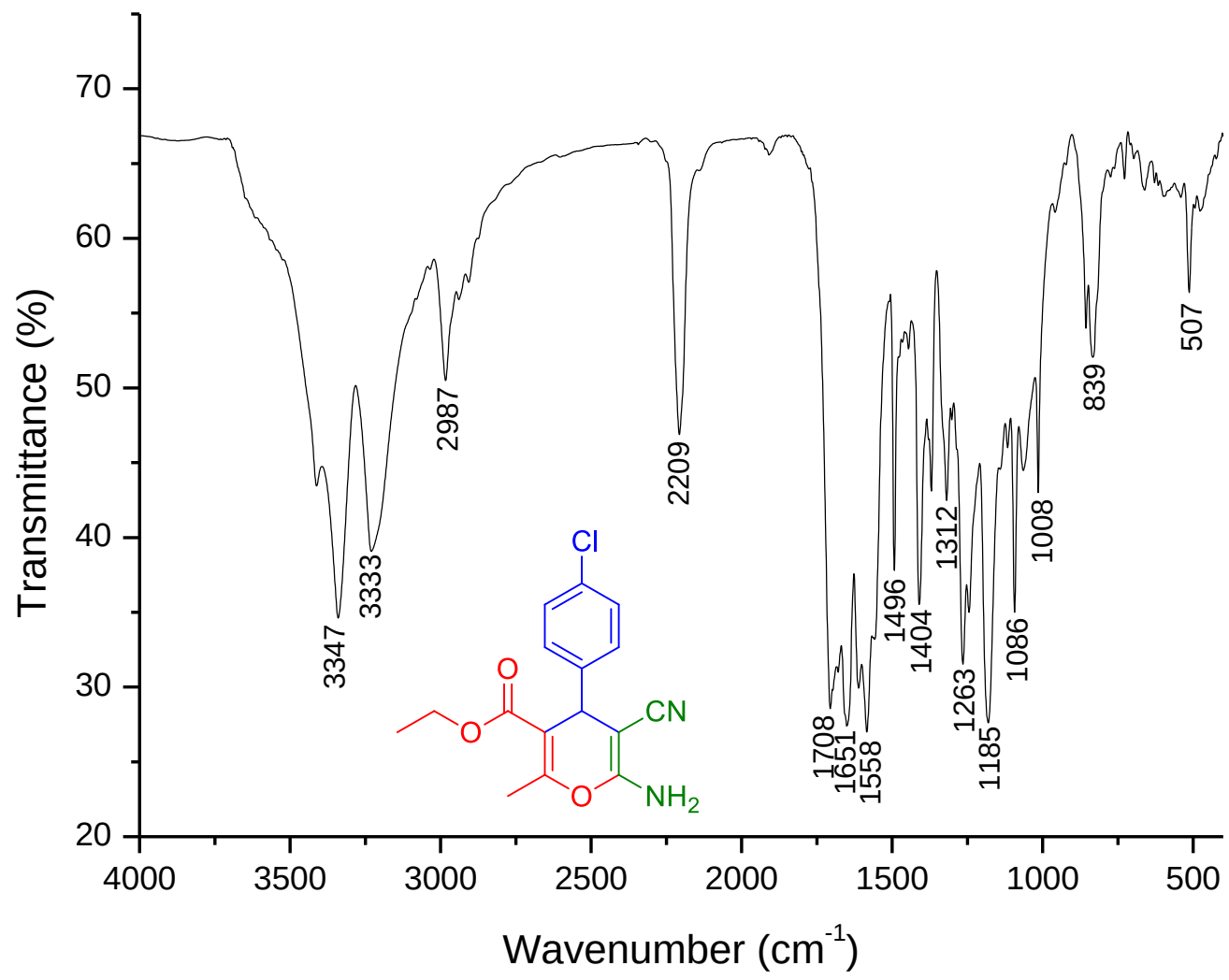

Figure S54. FT-IR (KBr) spectrum of compound **4e**.

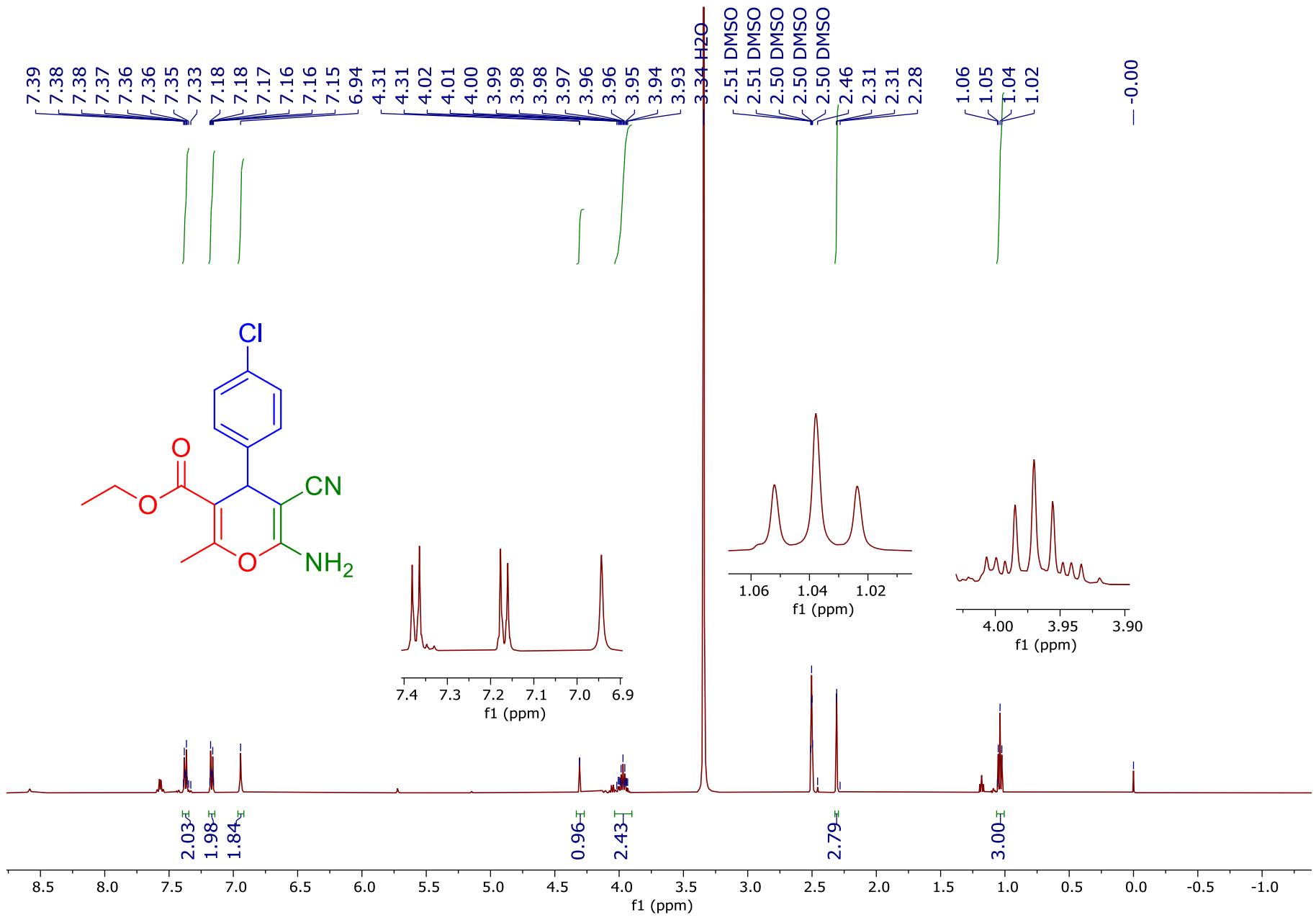

Figure S55. ^{1}H NMR spectrum (500 MHz, DMSO-d_6) of **4e**.

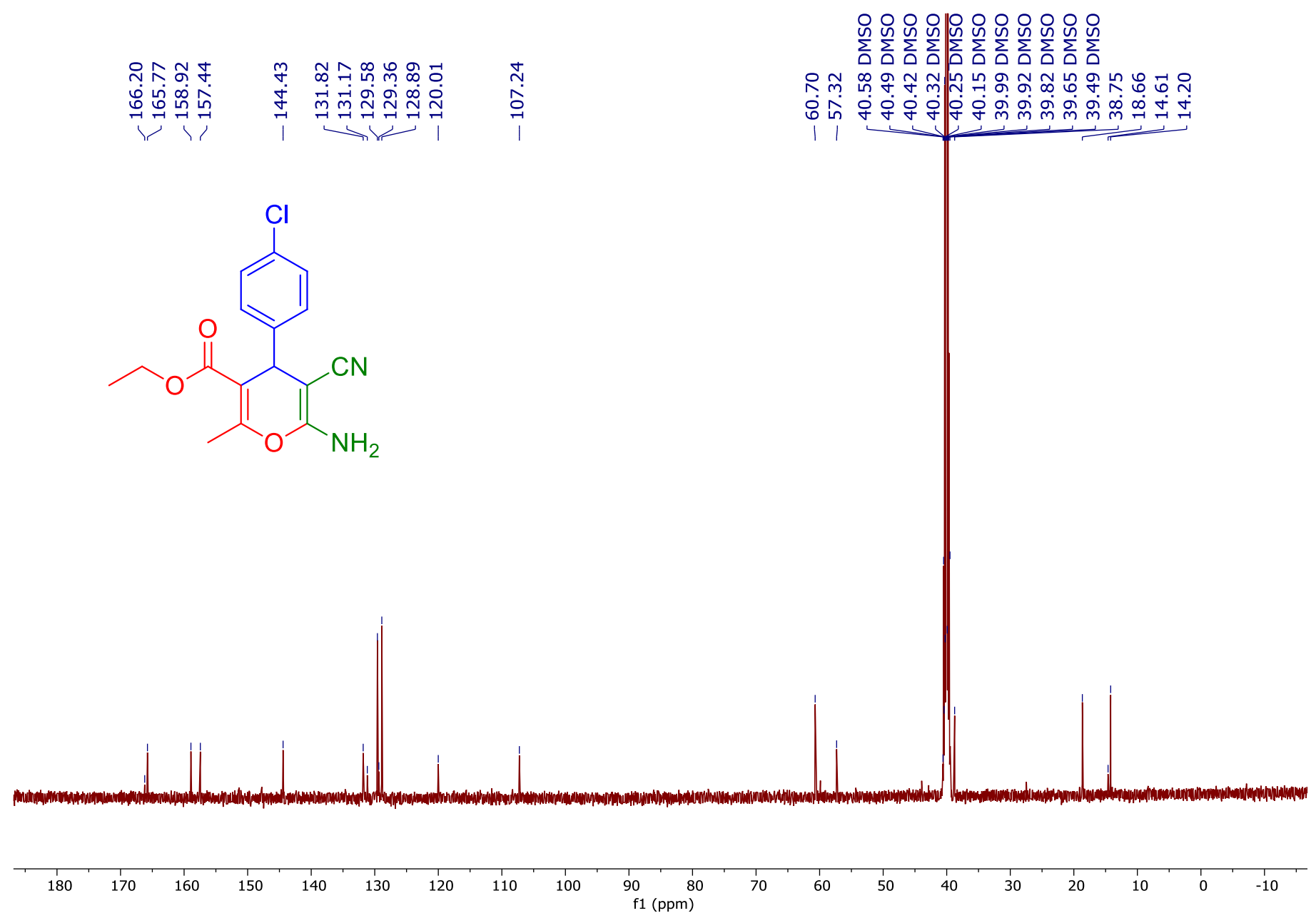

Figure S56. $^{13}C\{^{1}H\}$ NMR spectrum (125 MHz, DMSO-d_6) of **4e**.

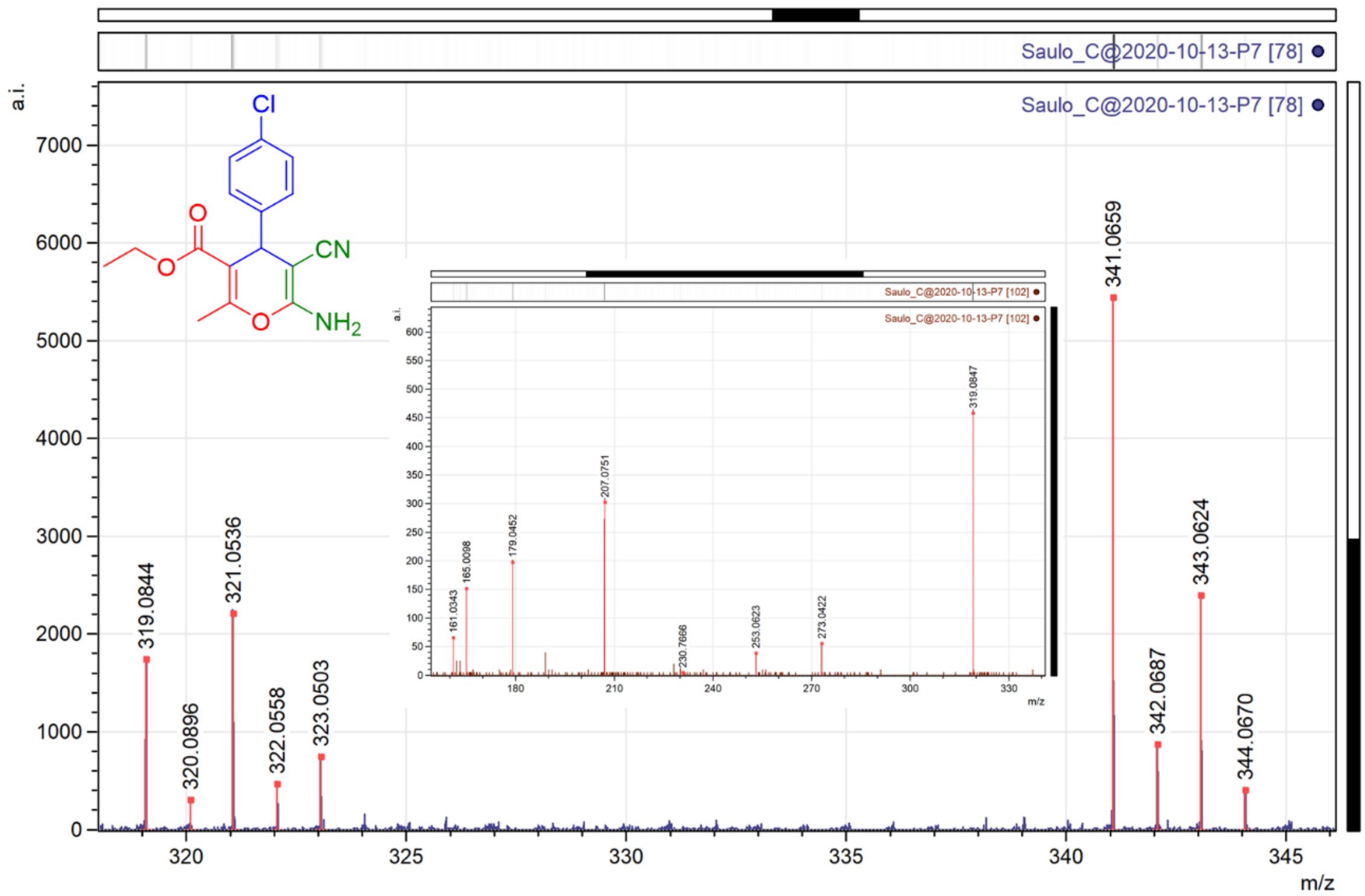

Figure S57. High-resolution ESI(+)-MS of **4e** showing its isotopic pattern. The inset is the ESI(+)-MS/MS from the $[M+H]^+$ isotopologue.

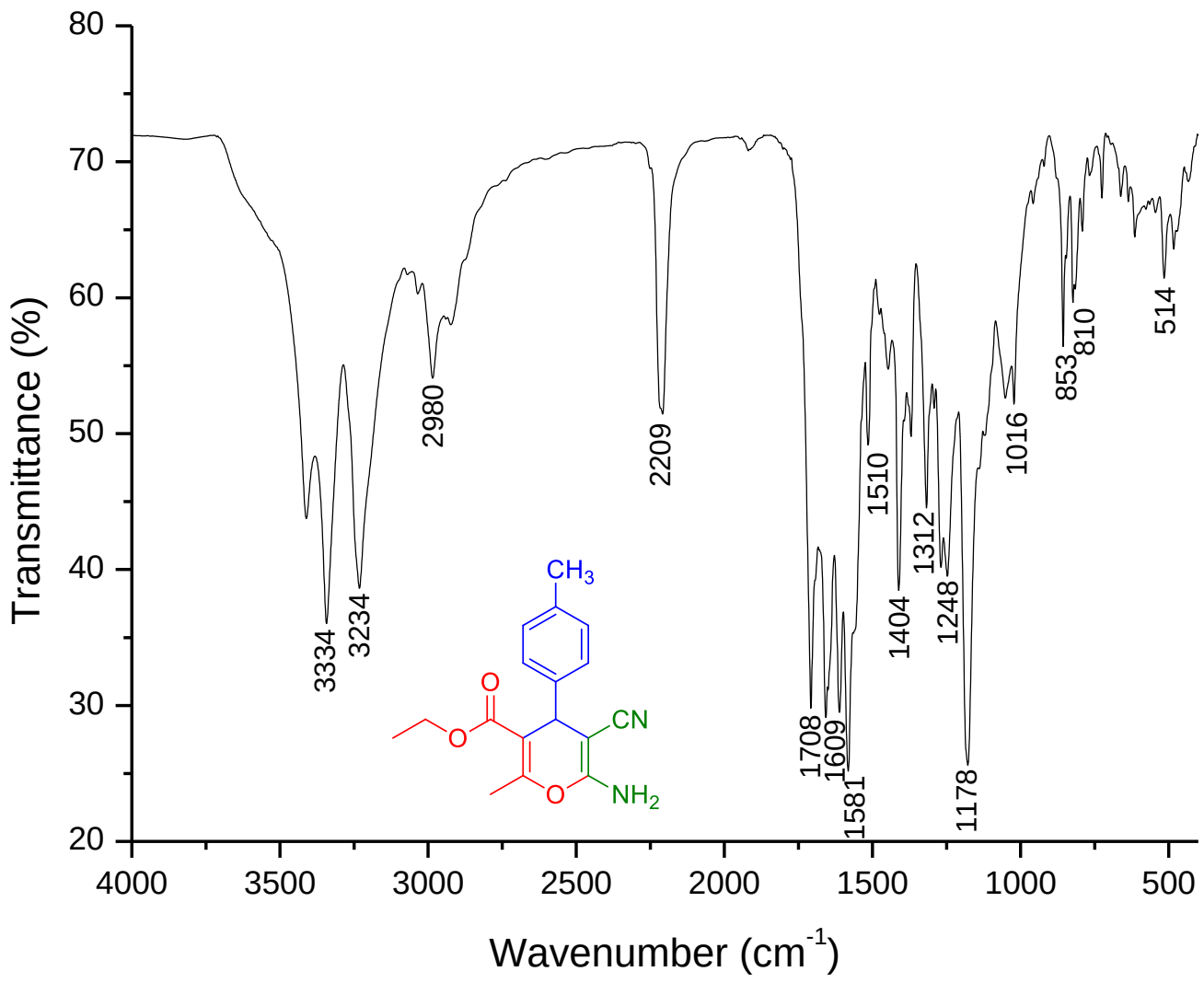

Figure S58. FT-IR (KBr) spectrum of compound **4f**.

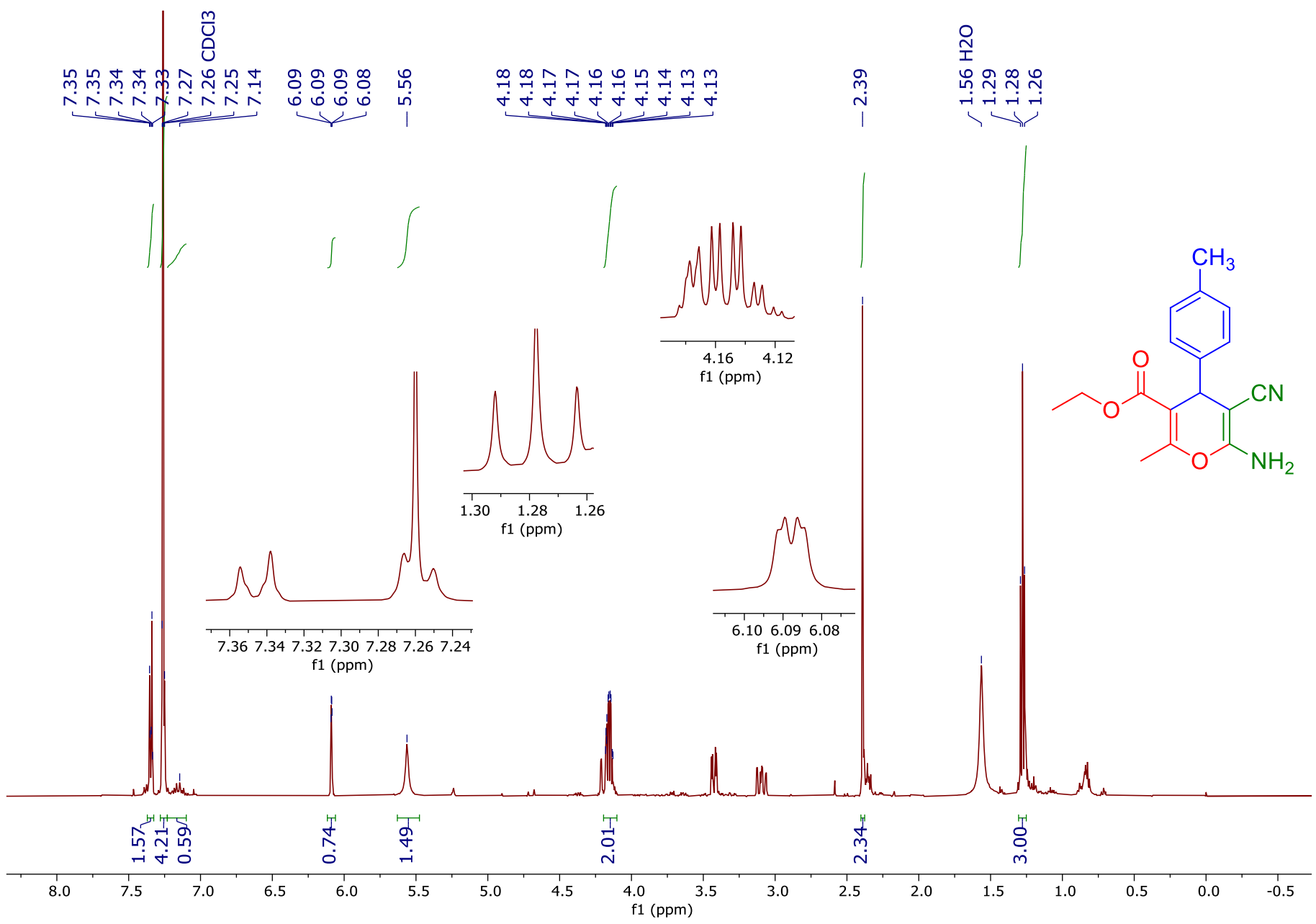

Figure S59. ^{1}H NMR spectrum (500 MHz, $CDCl_3$) of **4f**.

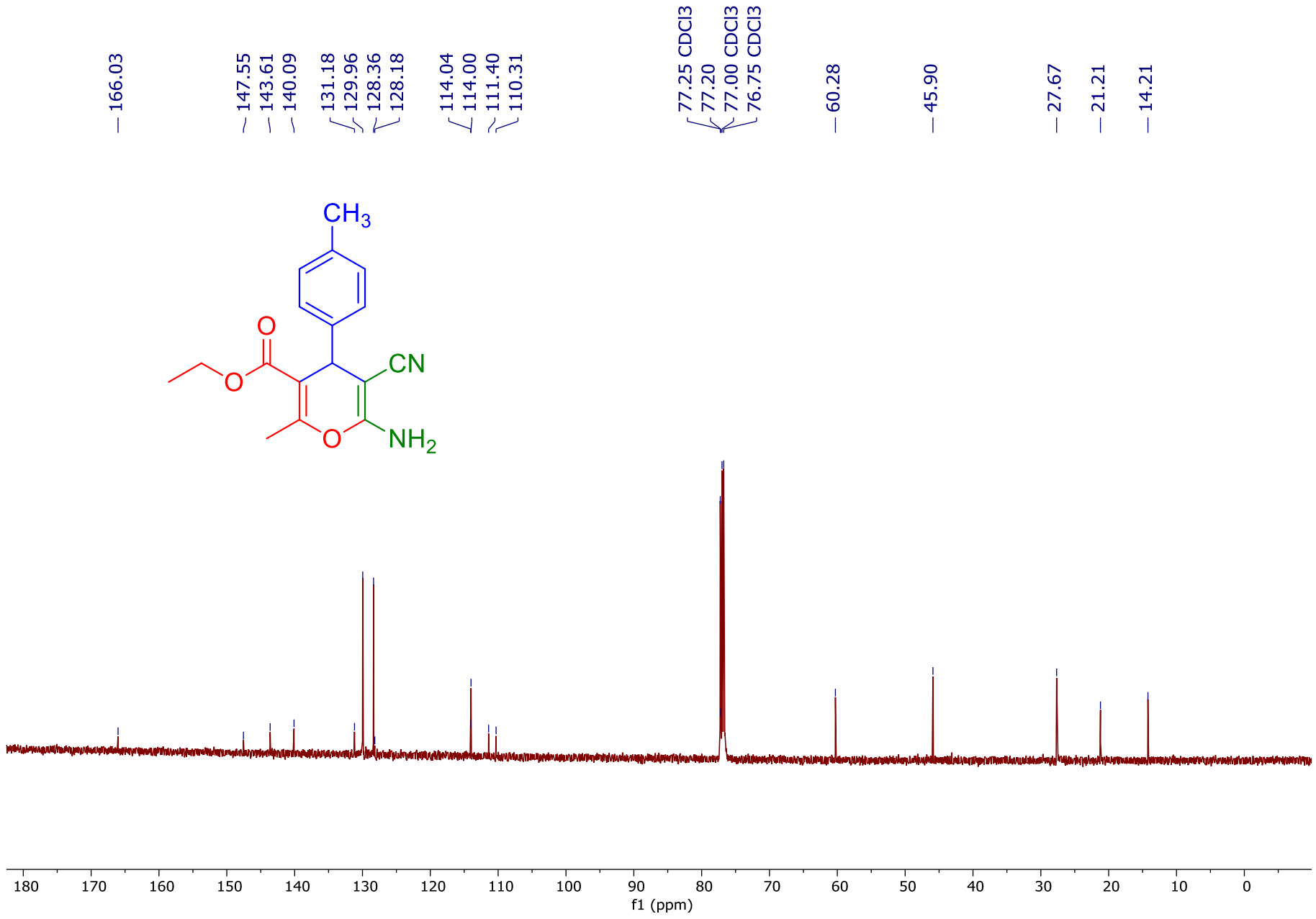

Figure S60. $^{13}C\ \{^{1}H\}$ NMR spectrum (125 MHz, $CDCl_3$) of **4f**.

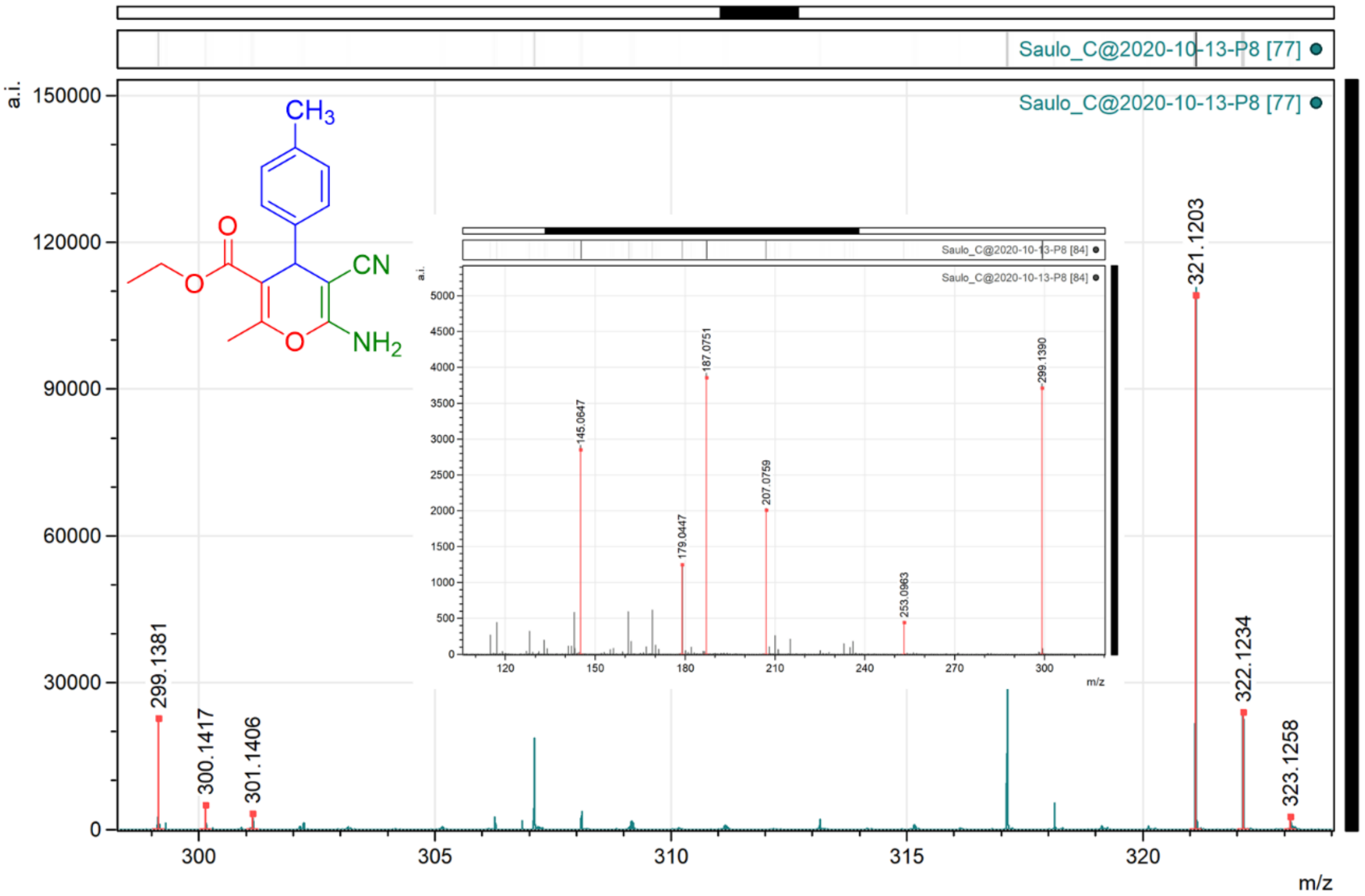

Figure S61. High-resolution ESI(+)-MS of **4f** showing its isotopic pattern. The inset is the ESI(+)-MS/MS from the $[M+H]^+$ isotopologue.

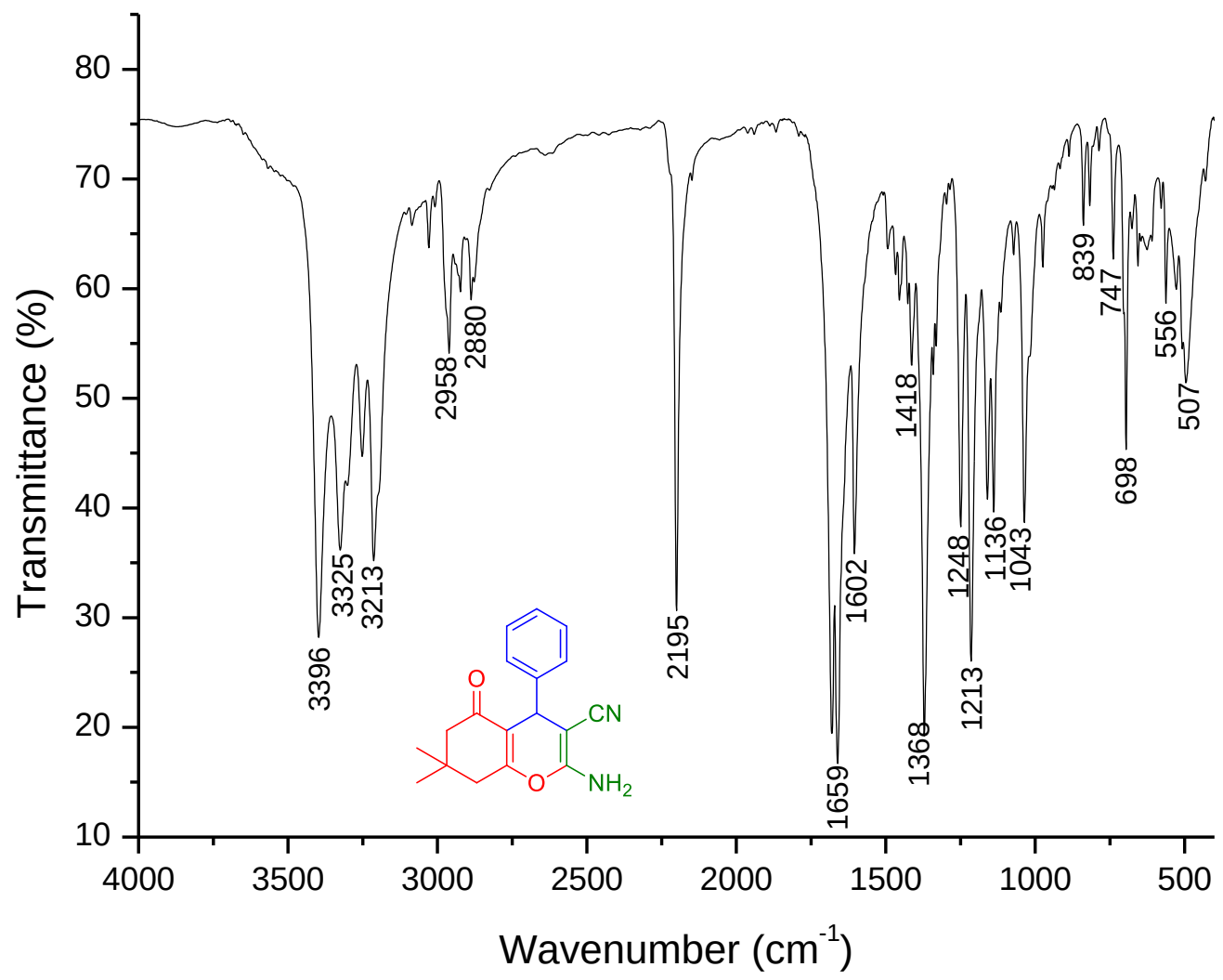

Figure S62. FT-IR (KBr) spectrum of compound **4g**.

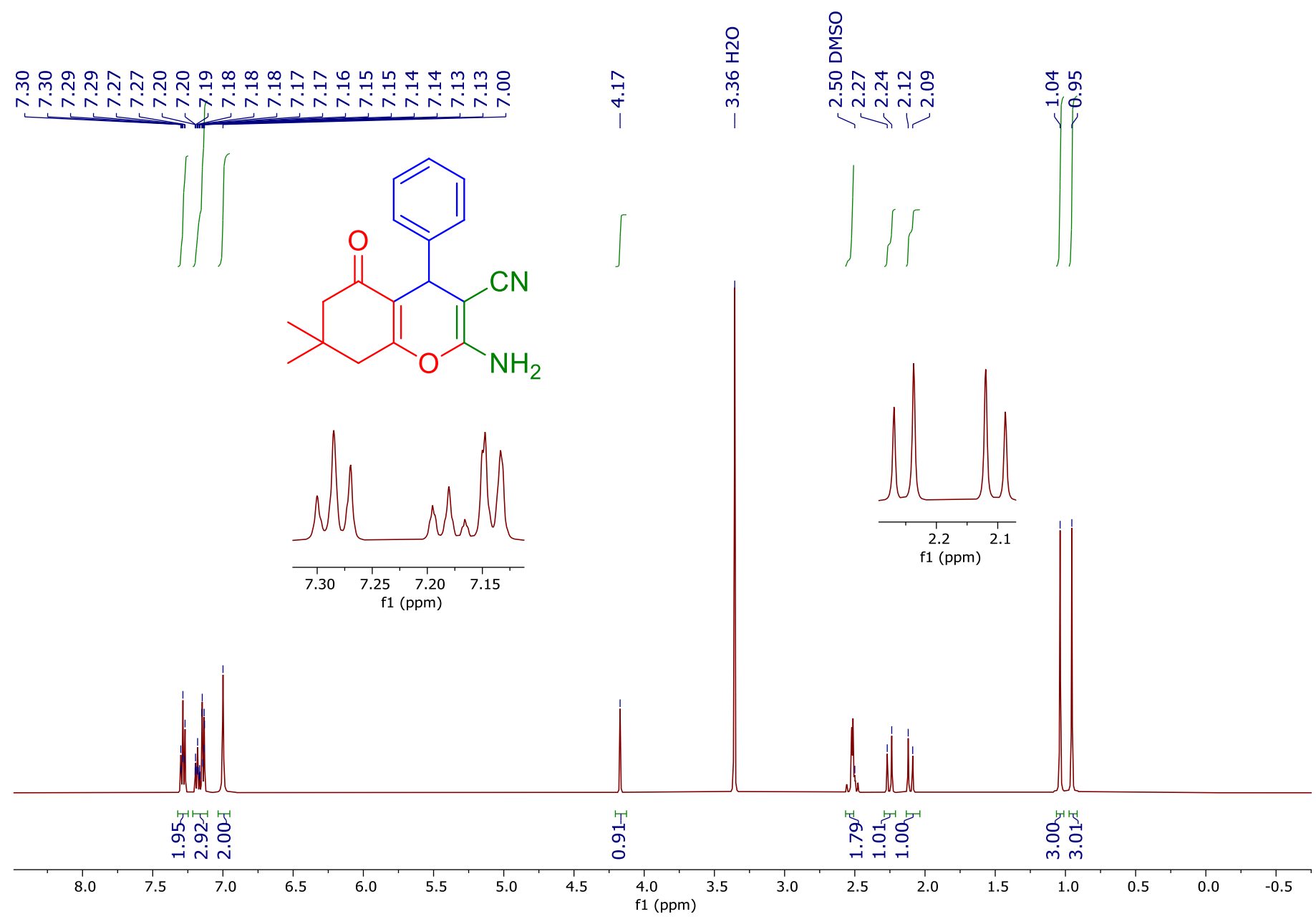

Figure S63. ^{1}H NMR spectrum (500 MHz, DMSO-d_6) of **4g**.

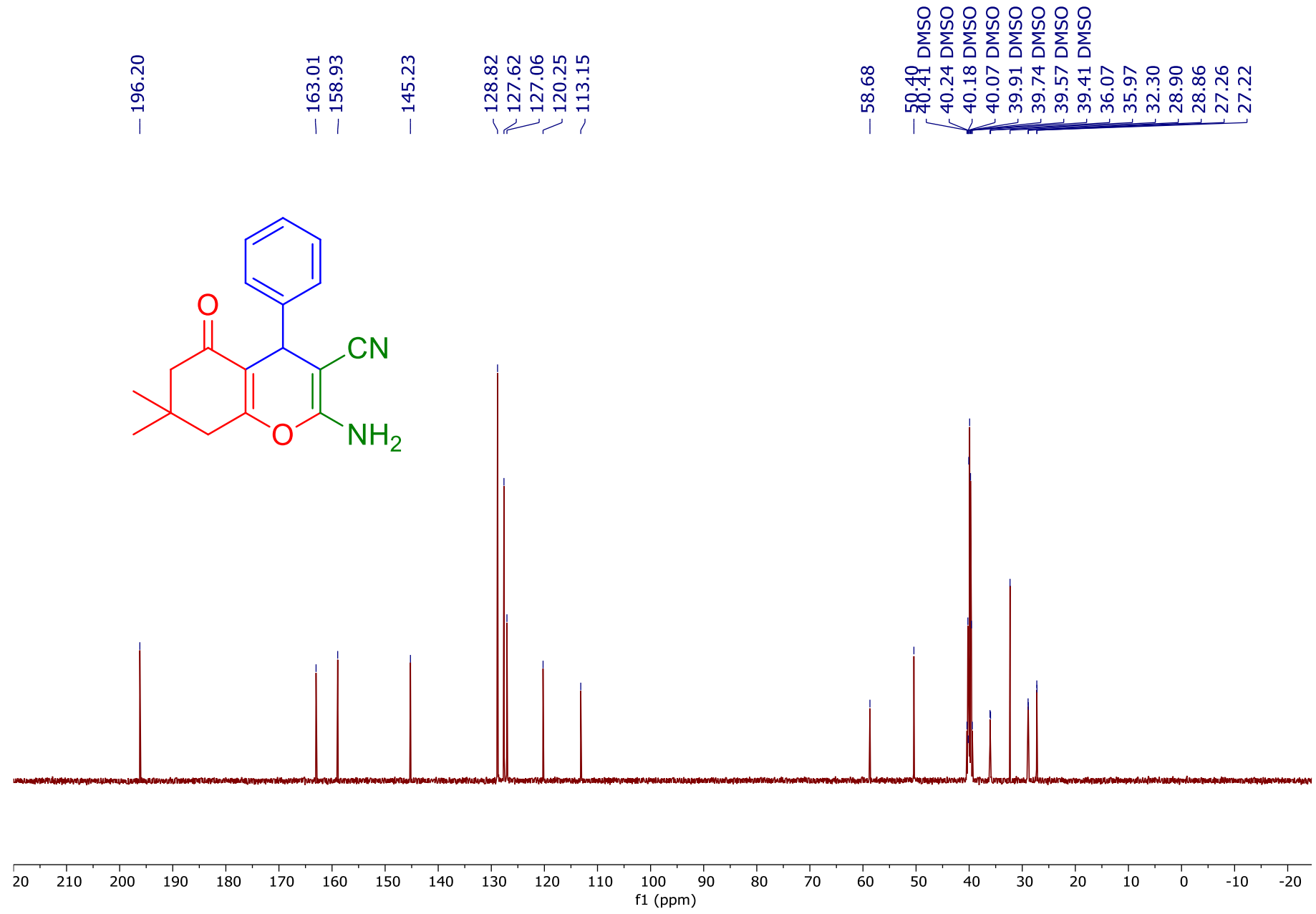

Figure S64. $^{13}C\{^{1}H\}$ NMR spectrum (125 MHz, DMSO-d_6) of **4g**.

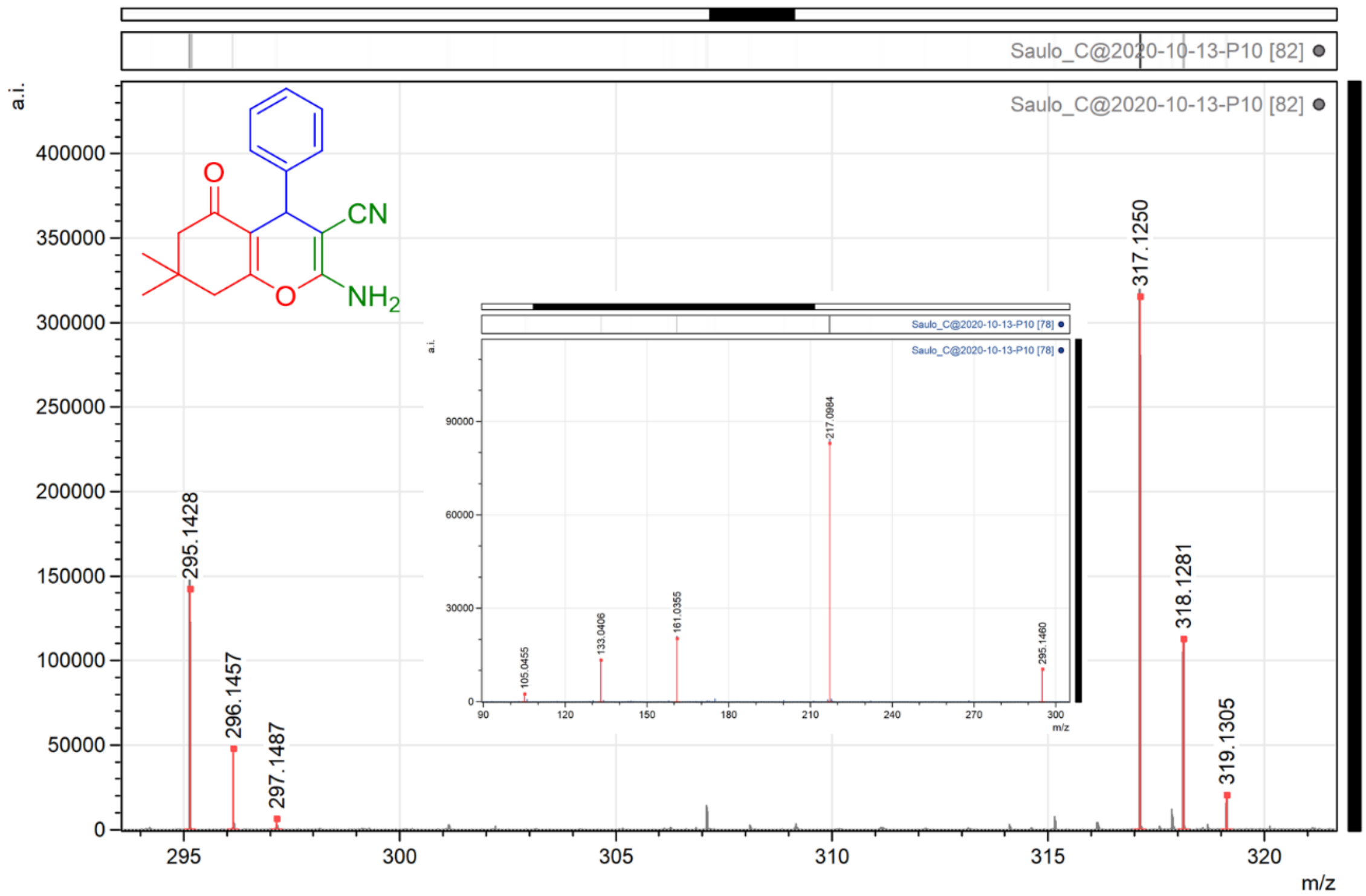

Figure S65. High-resolution ESI(+)-MS of **4g** showing its isotopic pattern. The inset is the ESI(+)-MS/MS from the $[M+H]^+$ isotopologue.

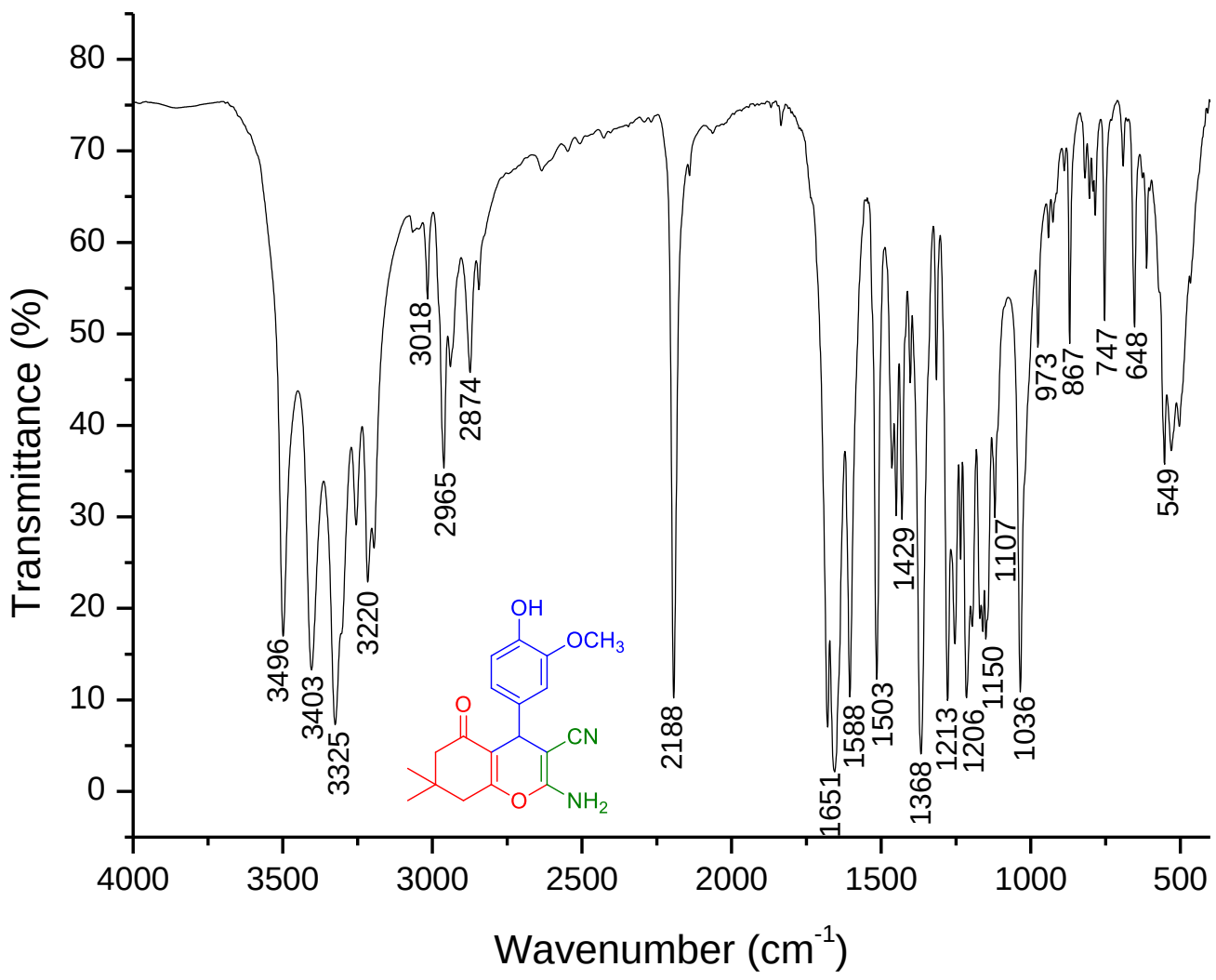

Figure S66. FT-IR (KBr) spectrum of compound **4h**.

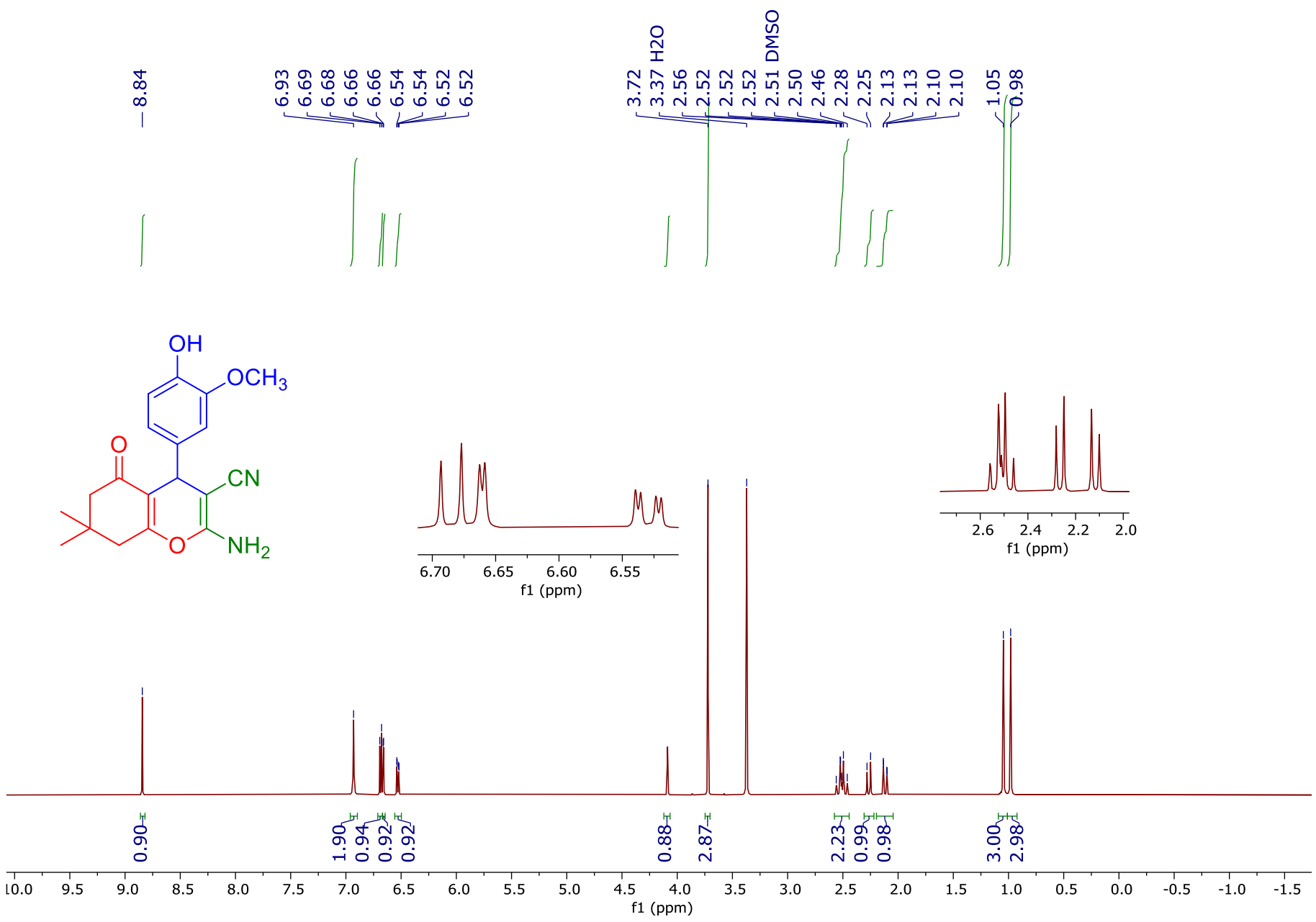

Figure S67. ^{1}H NMR spectrum (500 MHz, DMSO-d$_6$) of **4h**.

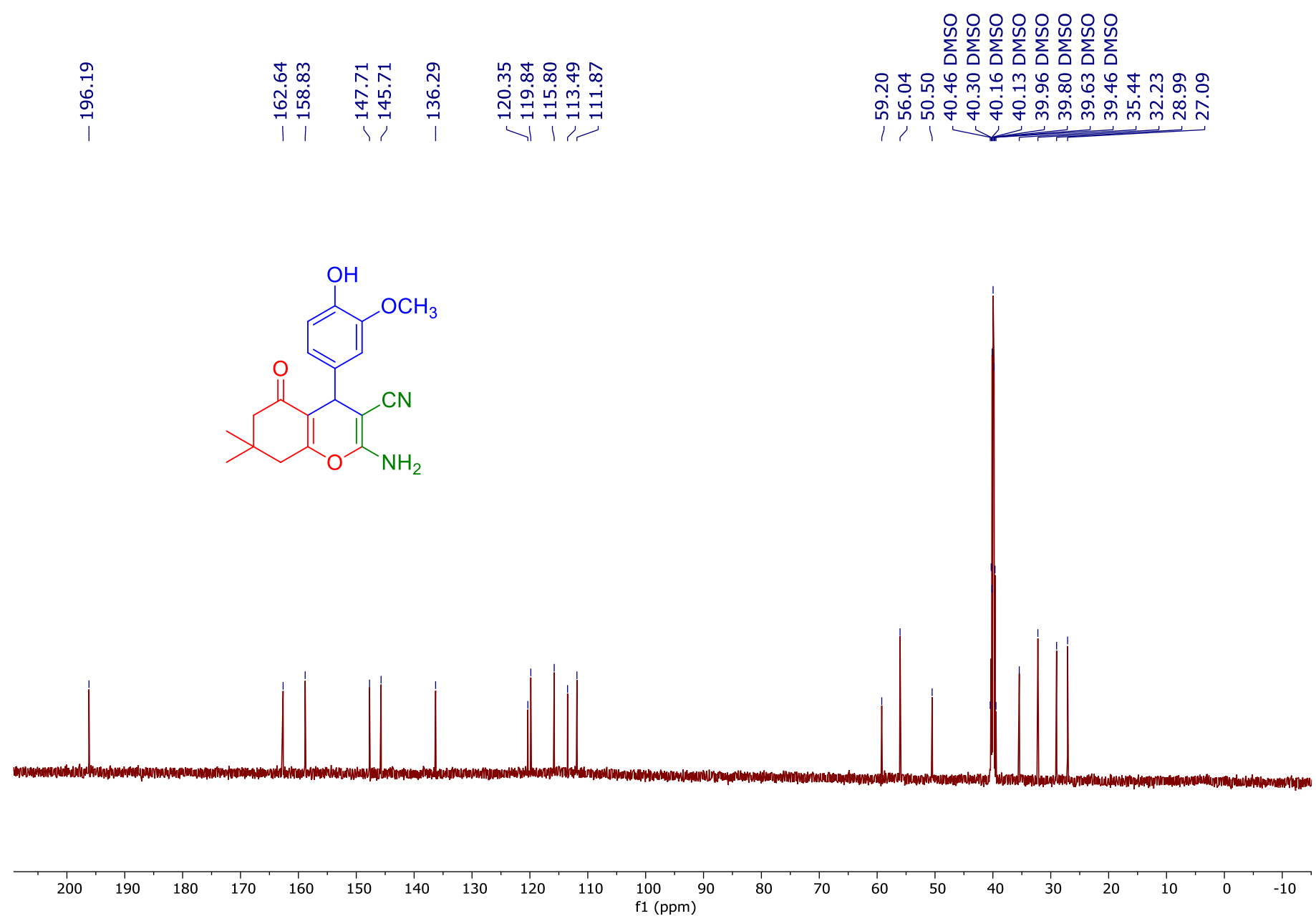

Figure S68. ^{13}C {^{1}H} NMR spectrum (125 MHz, DMSO-d_6) of **4h**. One carbon signal overlaps with the solvent signals.

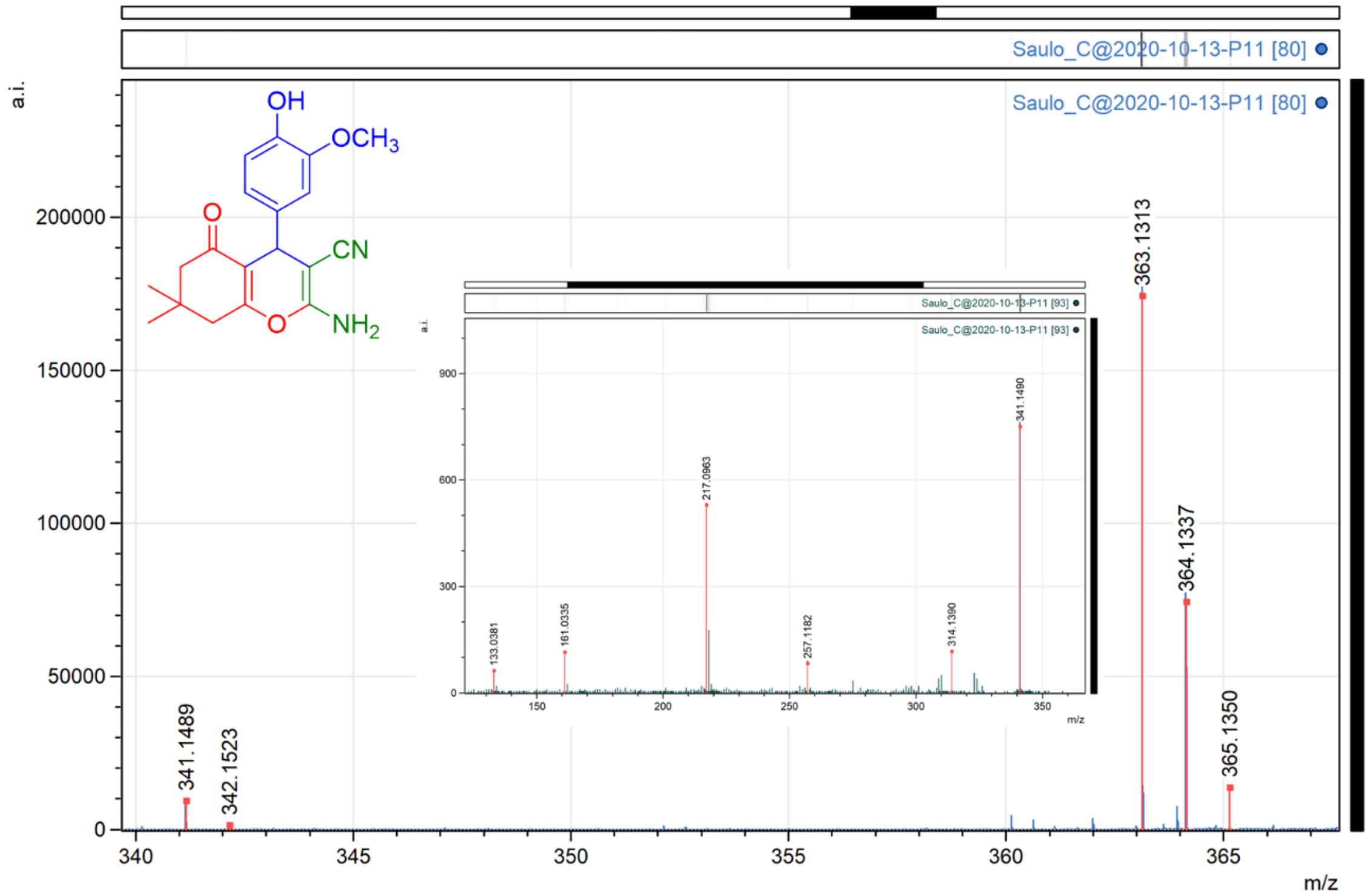

Figure S69. High-resolution ESI(+)-MS of **4h** showing its isotopic pattern. The inset is the ESI(+)-MS/MS from the $[M+H]^+$ isotopologue.

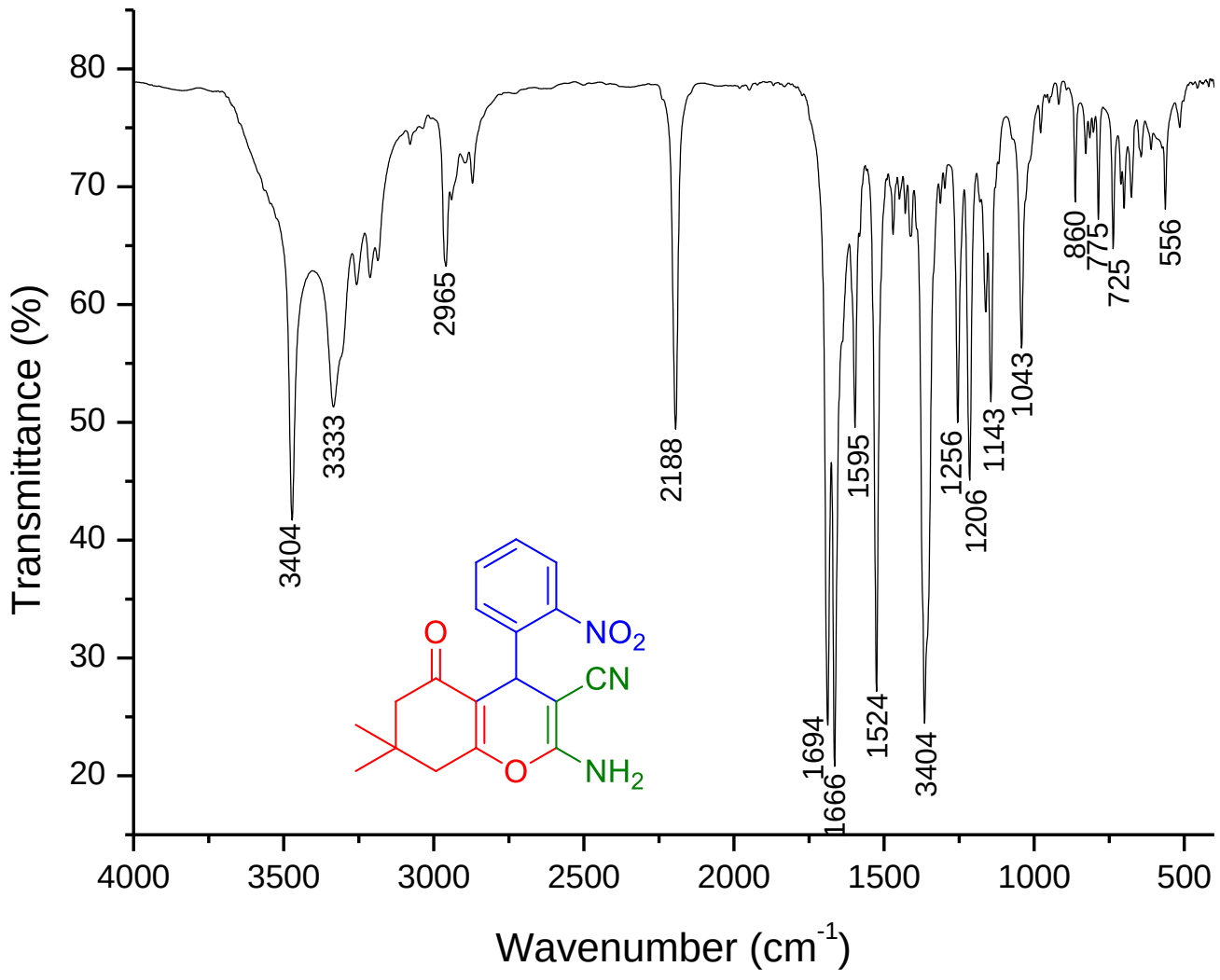

Figure S70. FT-IR (KBr) spectrum of compound **4i**.

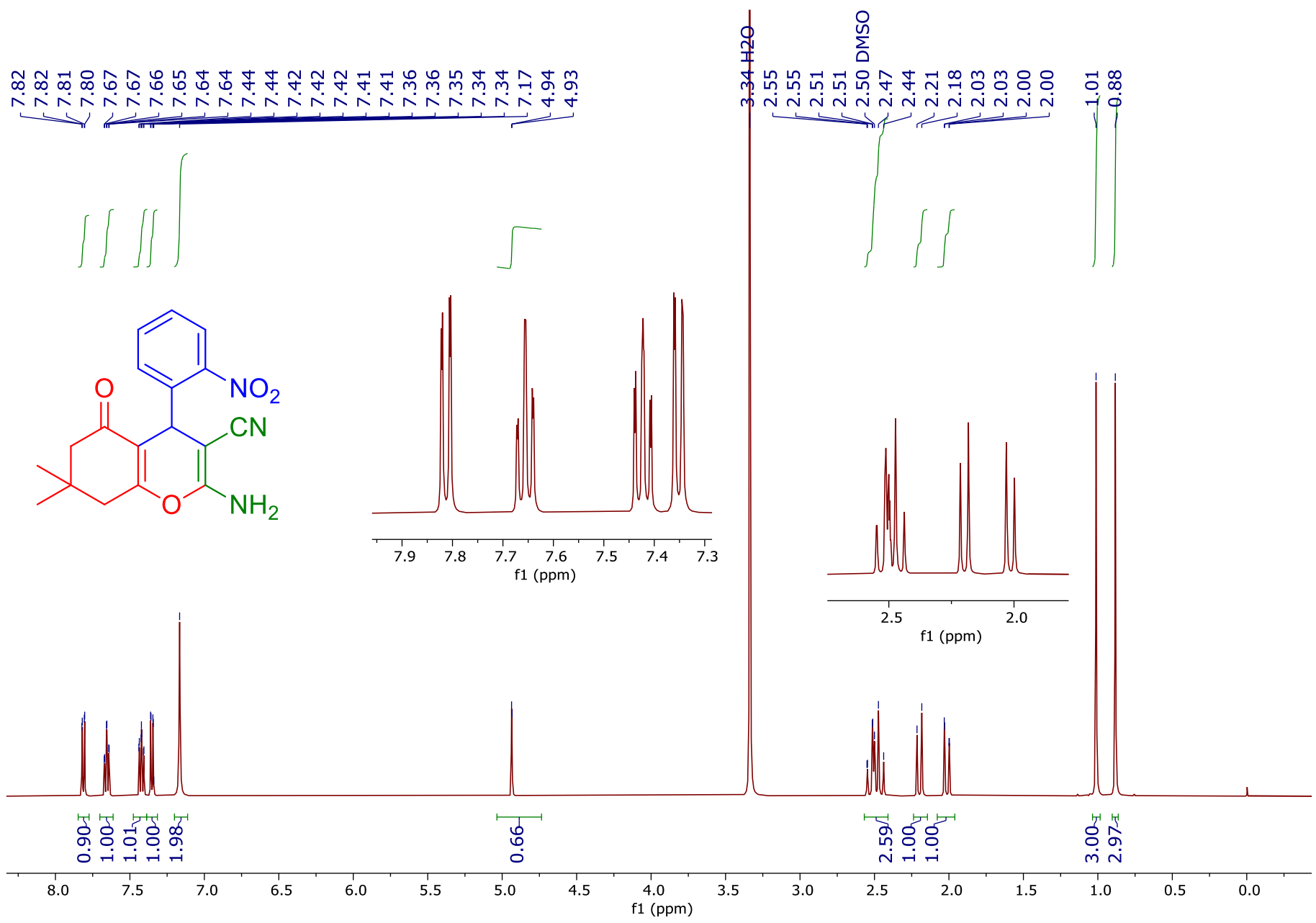

Figure S71. ^{1}H NMR spectrum (500 MHz, DMSO-d_6) of **4i**.

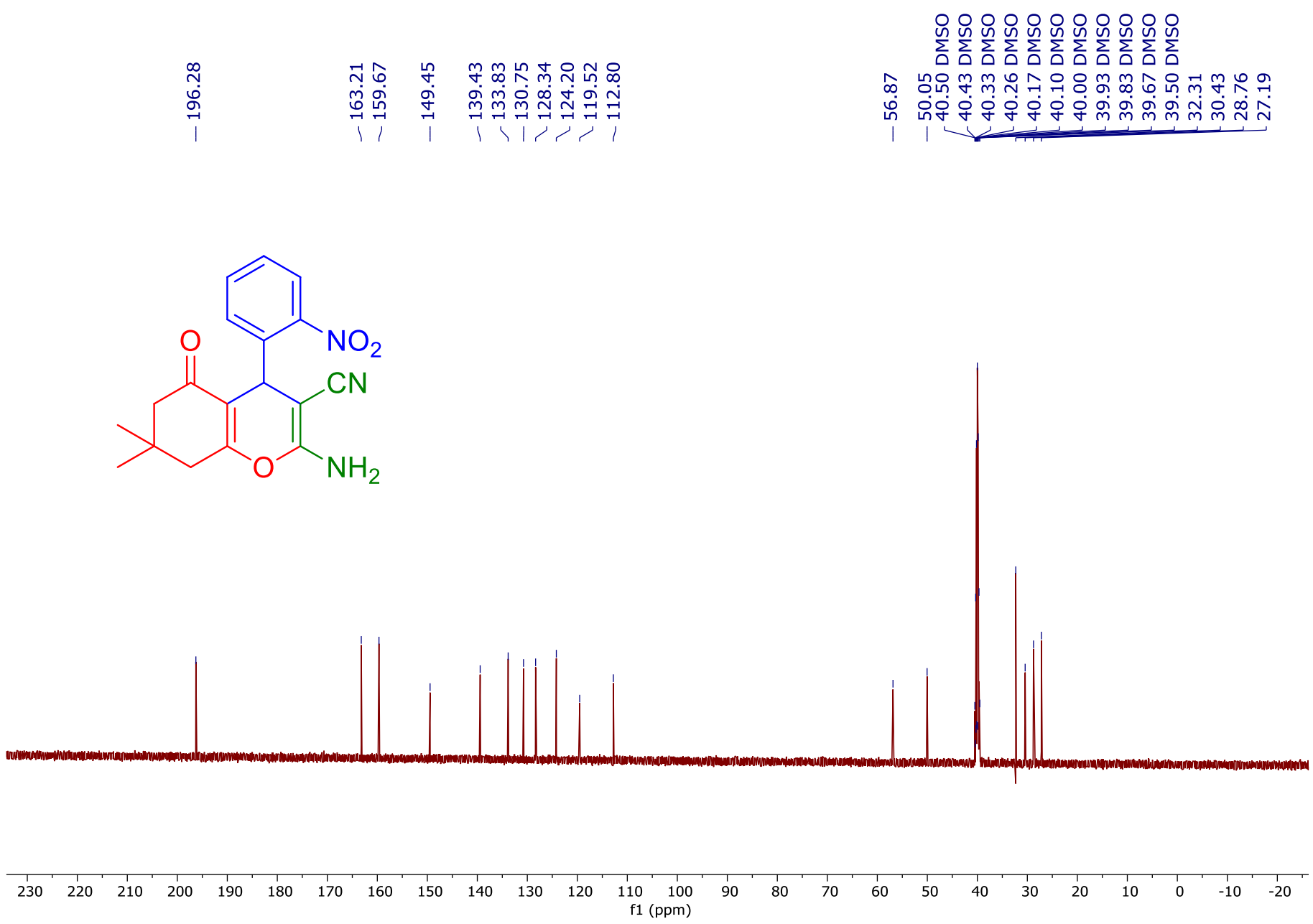

Figure S72. $^{13}C\ \{^1H\}$ NMR spectrum (125 MHz, DMSO-d_6) of **4i**. One carbon signal overlaps with the solvent signals.

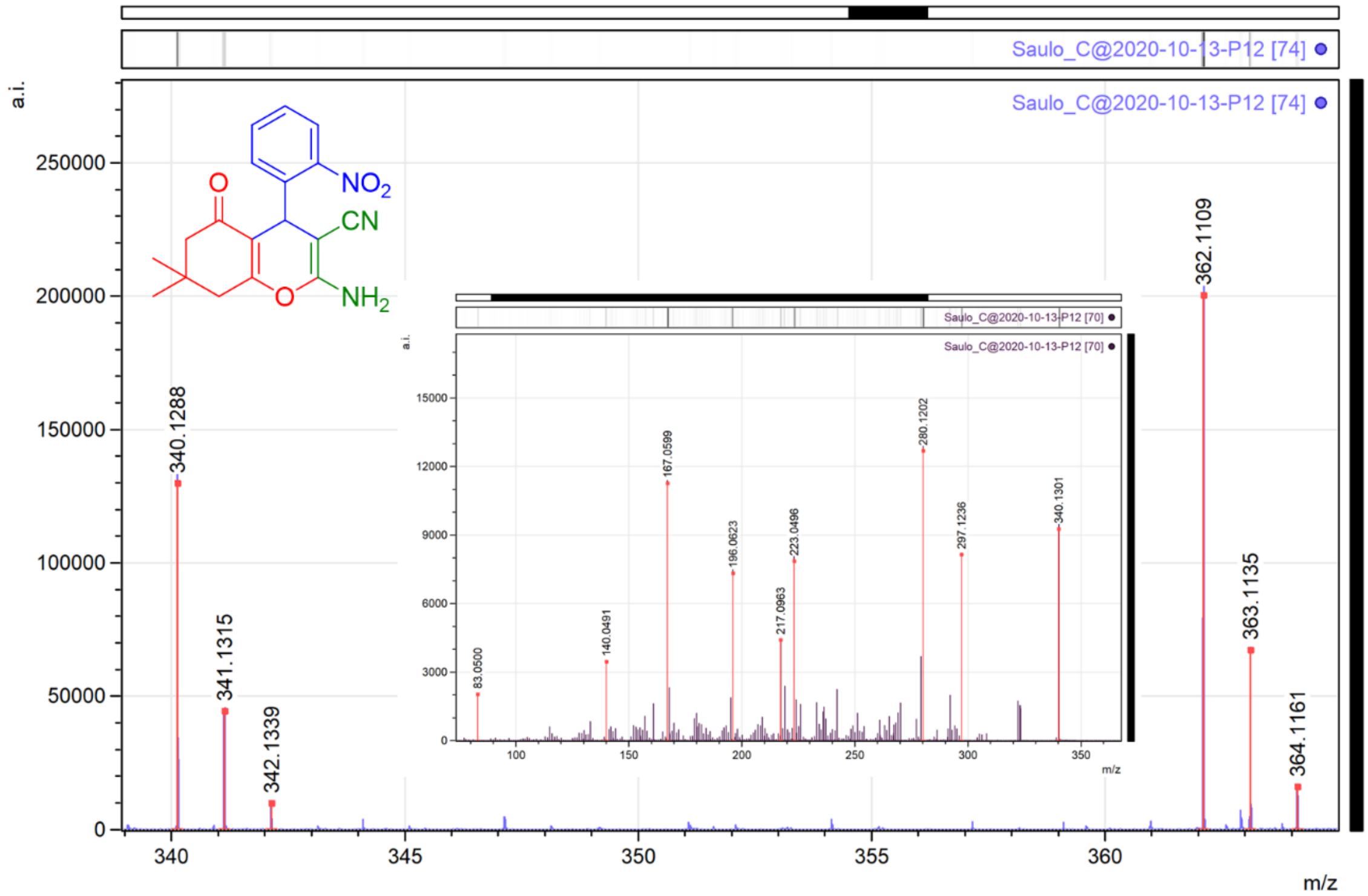

Figure S73. High-resolution ESI(+)-MS of **4i** showing its isotopic pattern. The inset is the ESI(+)-MS/MS from the $[M+H]^+$ isotopologue.

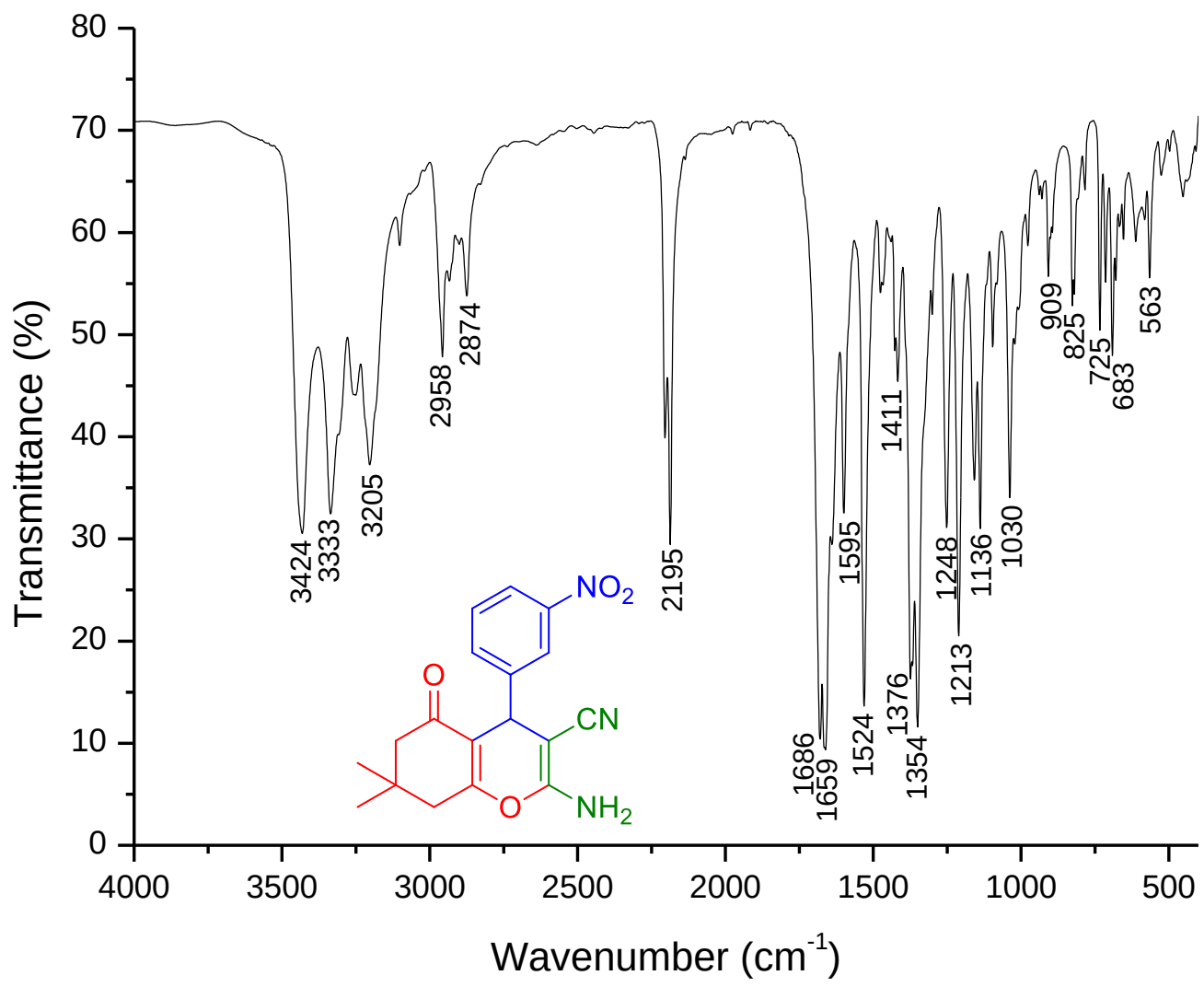

Figure S74. FT-IR (KBr) spectrum of compound **4j**.

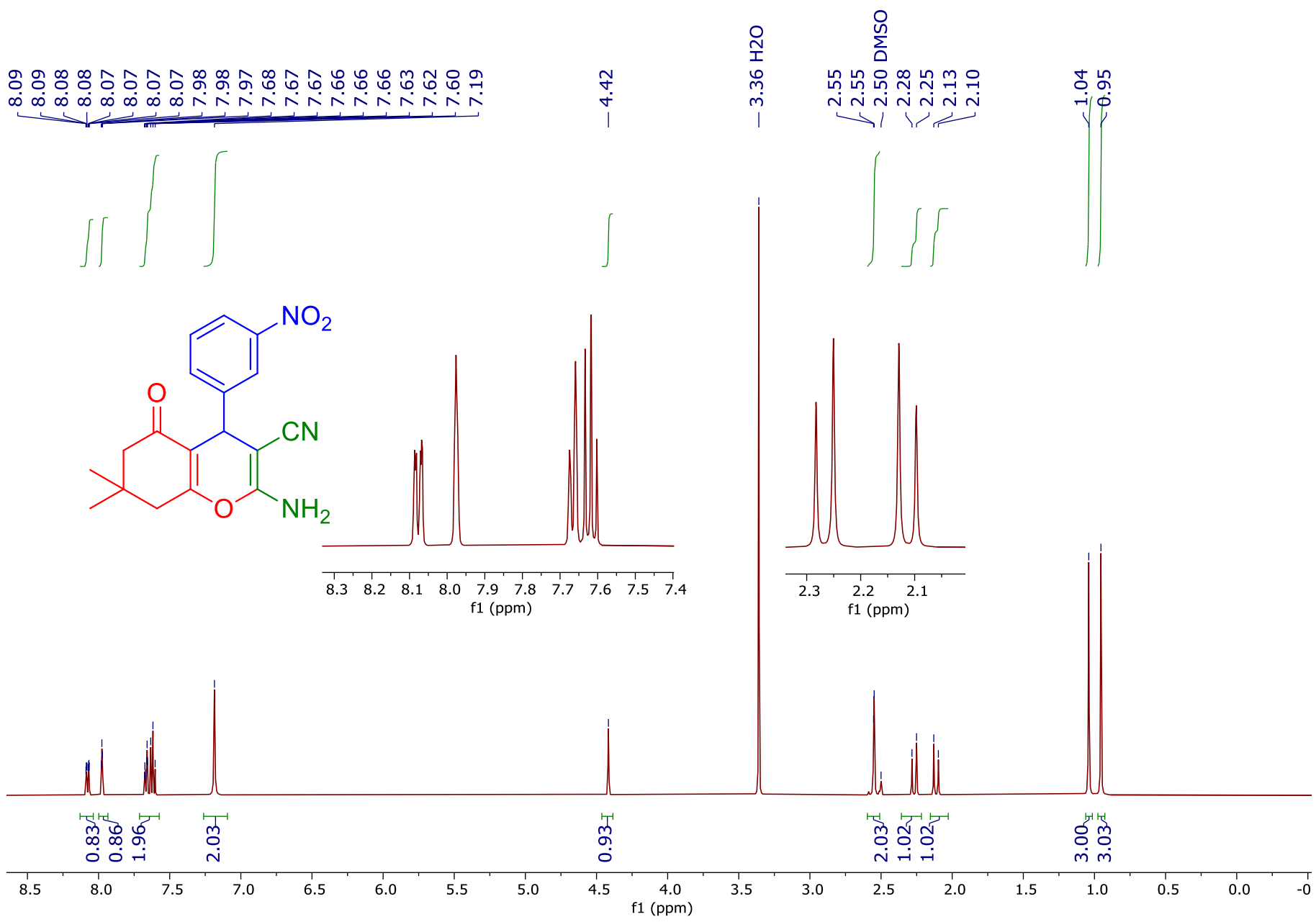

Figure S75. ^{1}H NMR spectrum (500 MHz, DMSO-d_6) of **4j**.

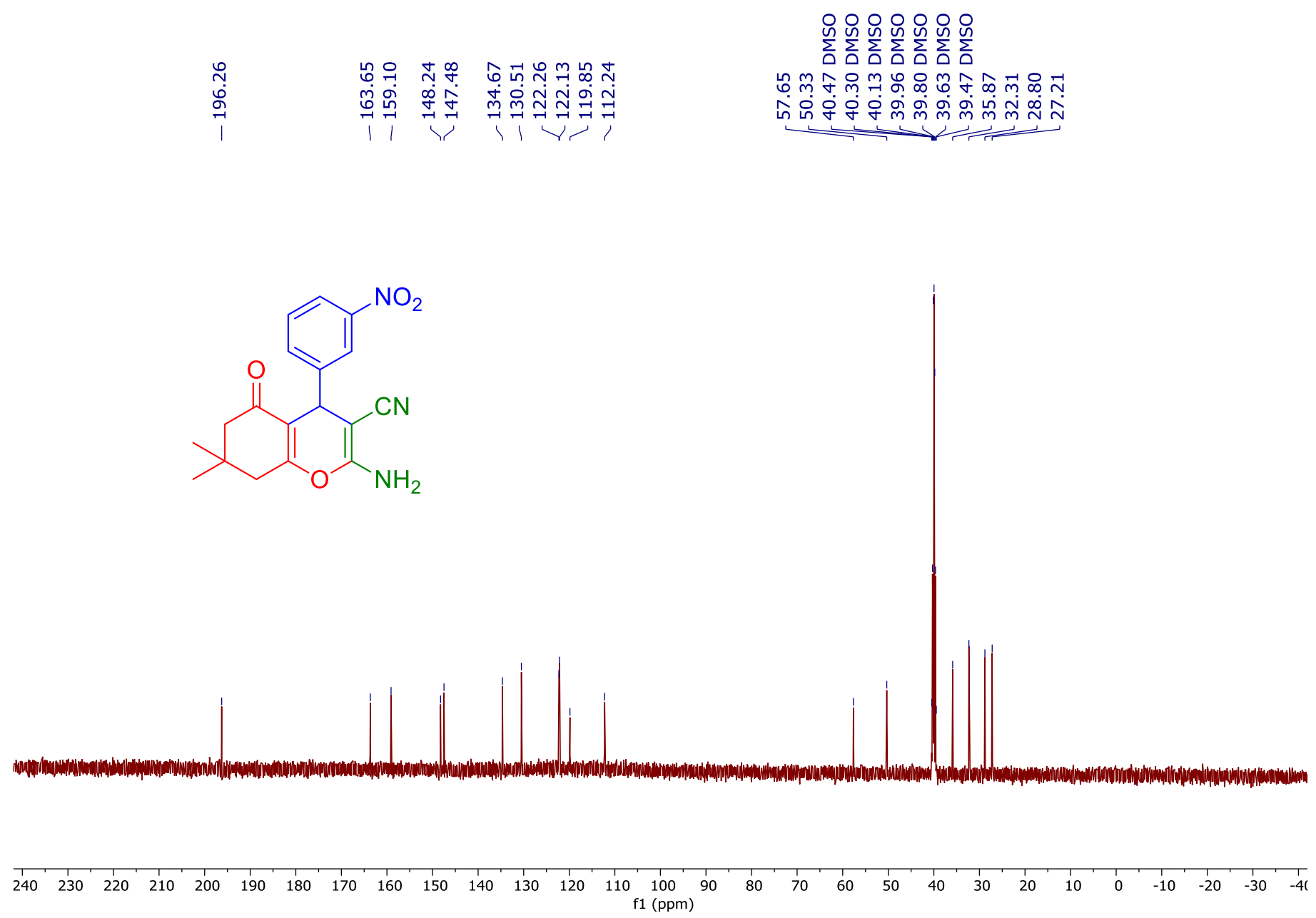

Figure S76. ^{13}C {^1H} NMR spectrum (125 MHz, DMSO-d_6) of **4j**. One carbon signal overlaps with the solvent signals.

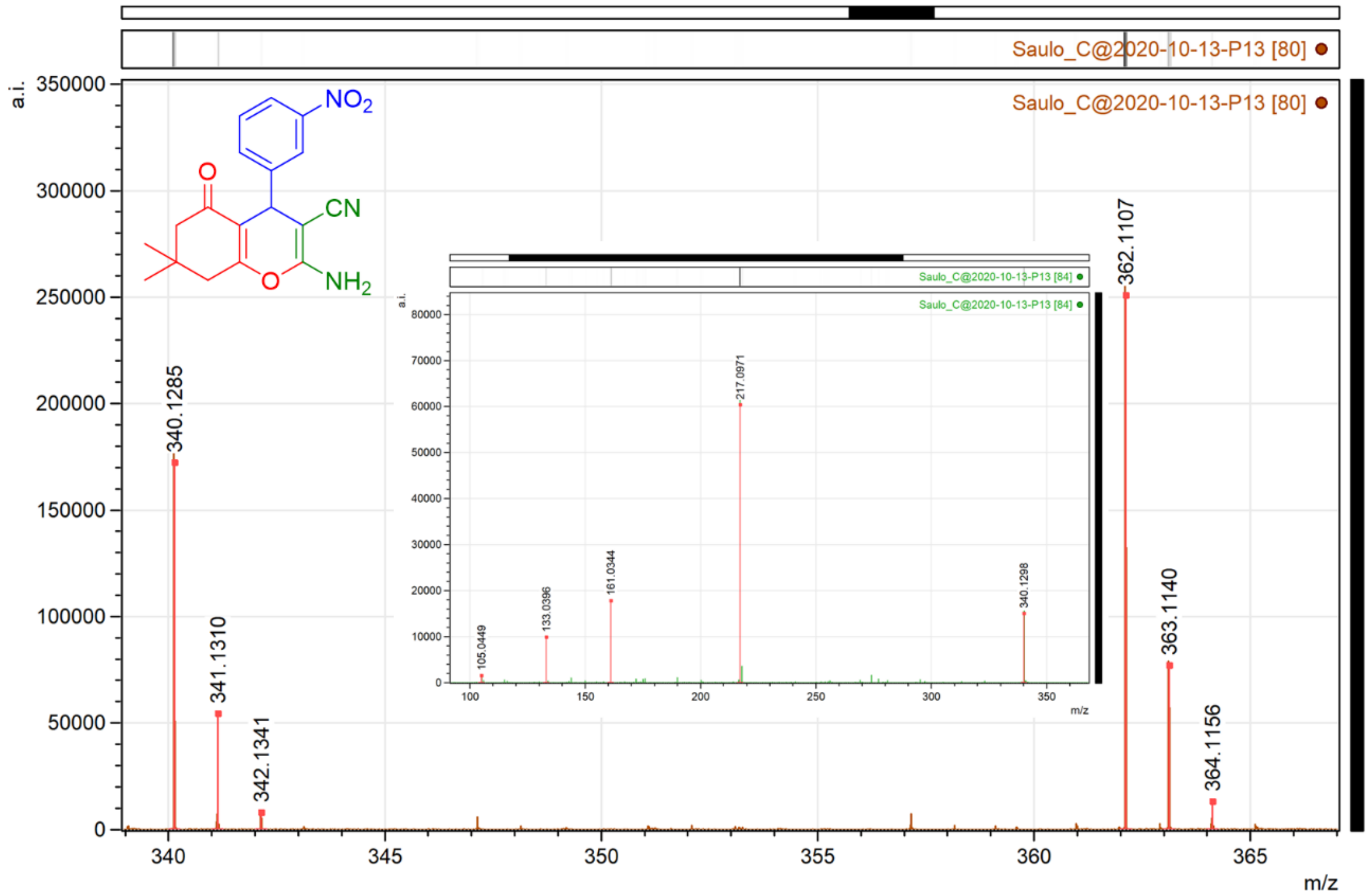

Figure S77. High-resolution ESI(+)-MS of **4j** showing its isotopic pattern. The inset is the ESI(+)-MS/MS from the $[M+H]^+$ isotopologue.

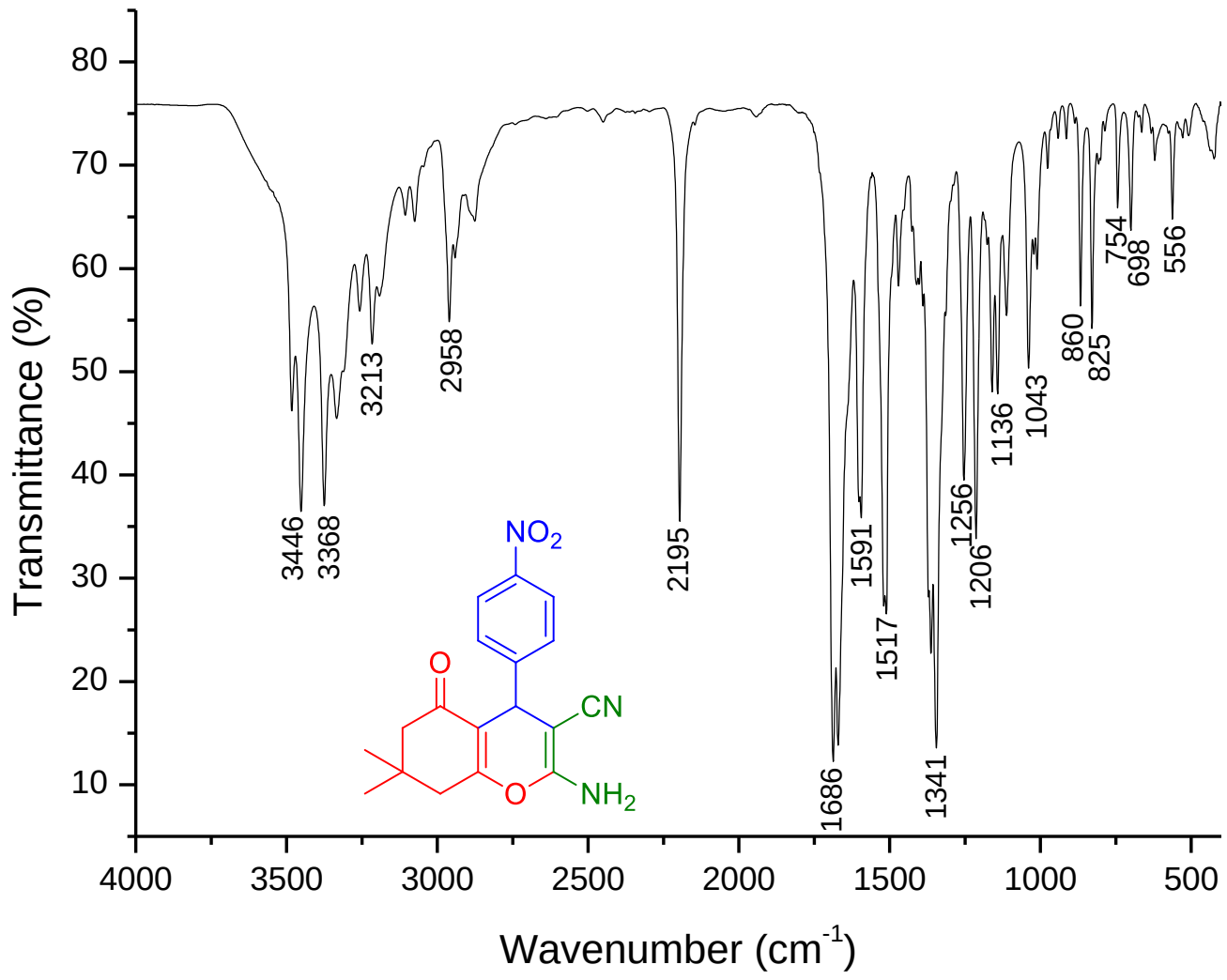

Figure S78. FT-IR (KBr) spectrum of compound **4k**.

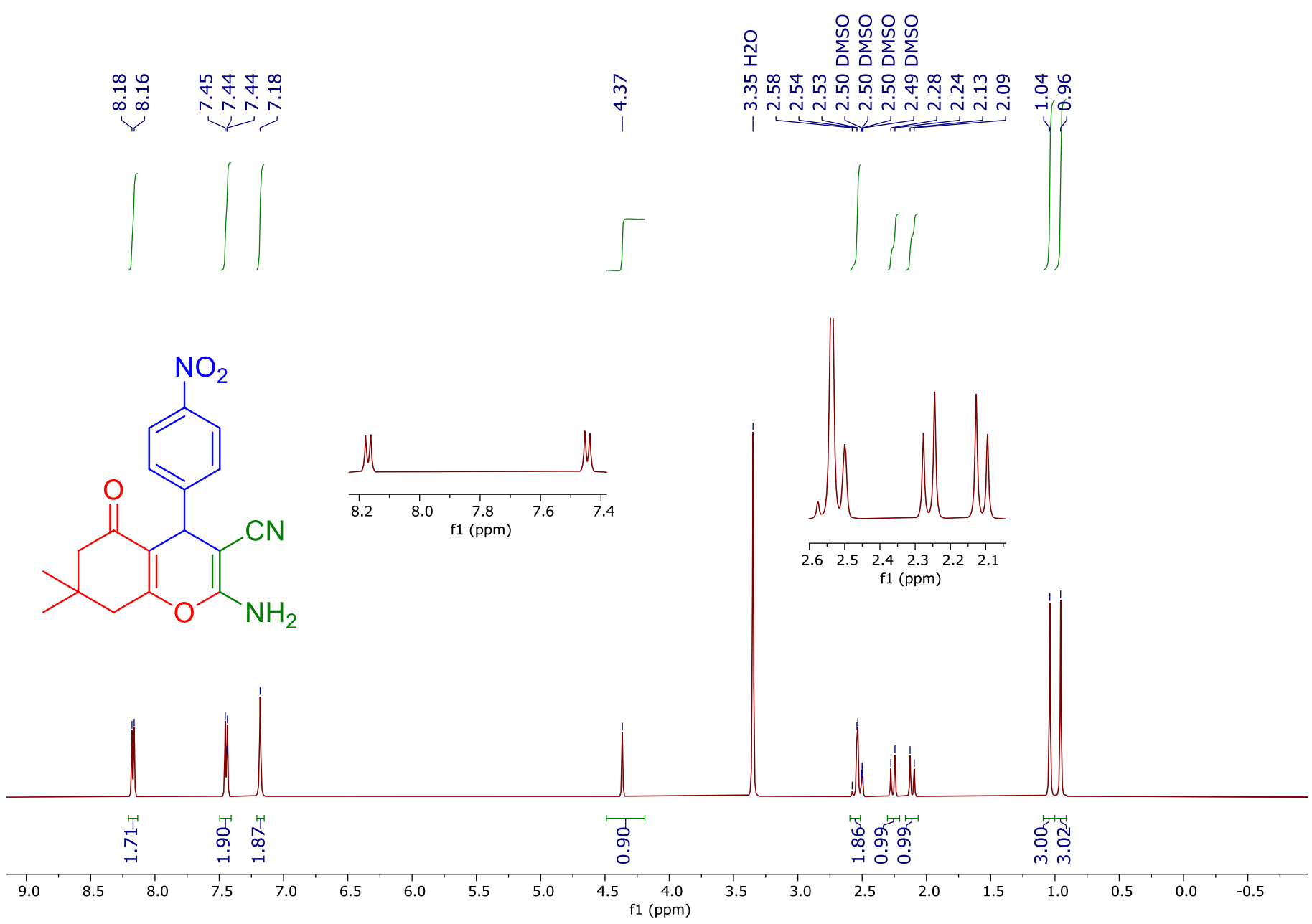

Figure S79. ^{1}H NMR spectrum (500 MHz, DMSO-d_6) of **4k**.

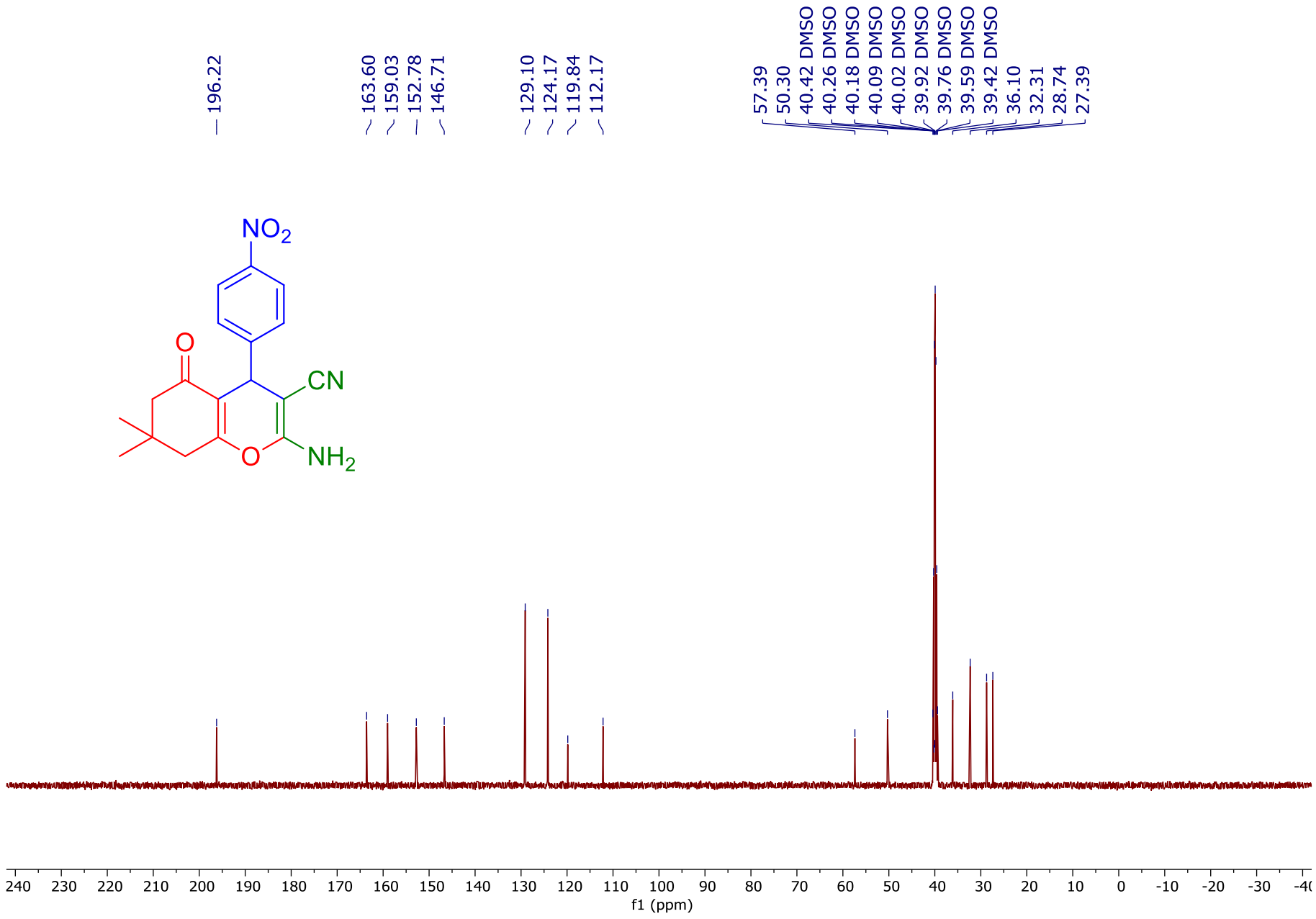

Figure S80. ^{13}C {^{1}H} NMR spectrum (125 MHz, DMSO-d*6*) of **4k**. One carbon signal overlaps with the solvent signals.

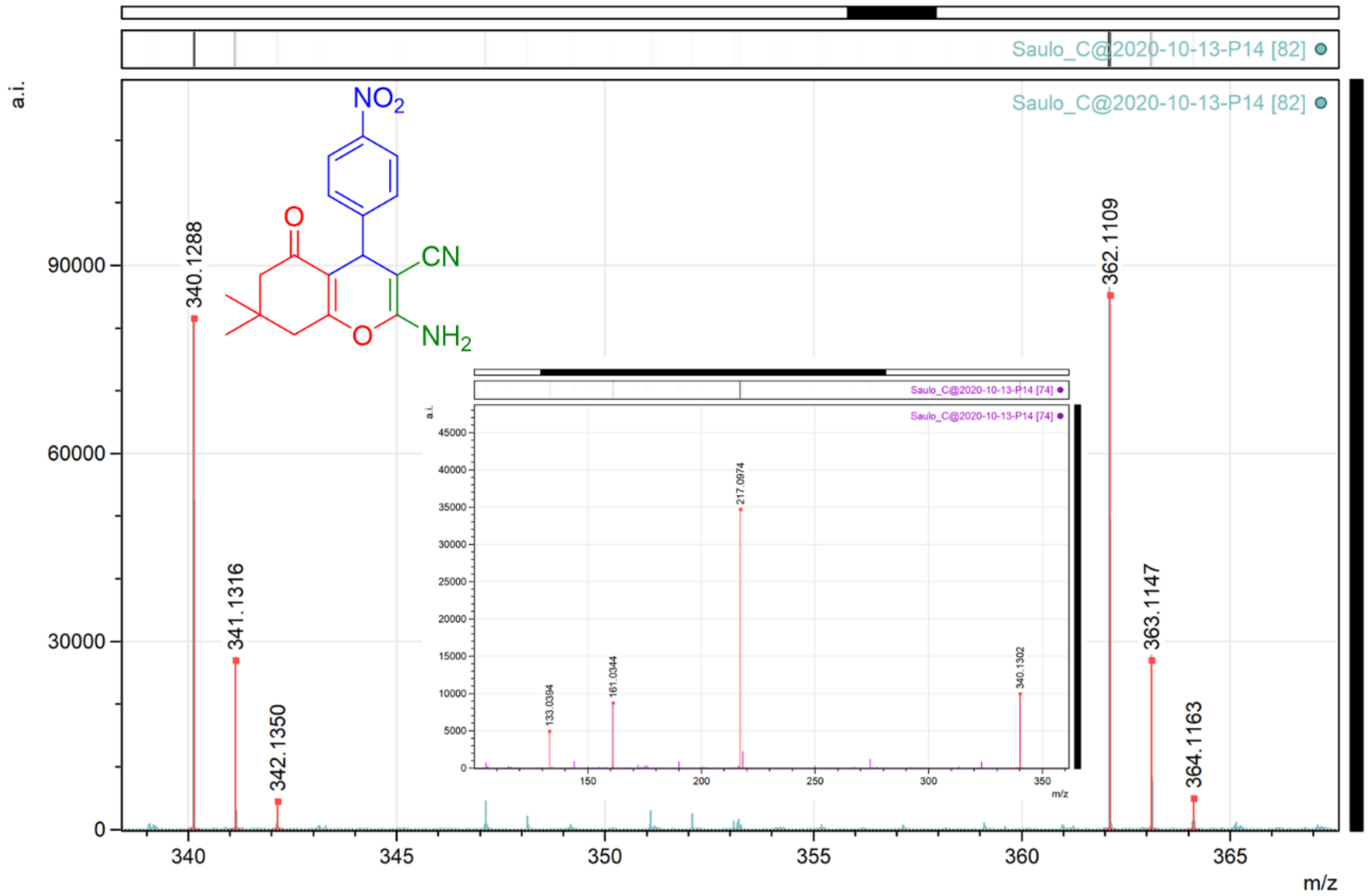

Figure S81. High-resolution ESI(+)-MS of **4k** showing its isotopic pattern. The inset is the ESI(+)-MS/MS from the $[M+H]^+$ isotopologue.

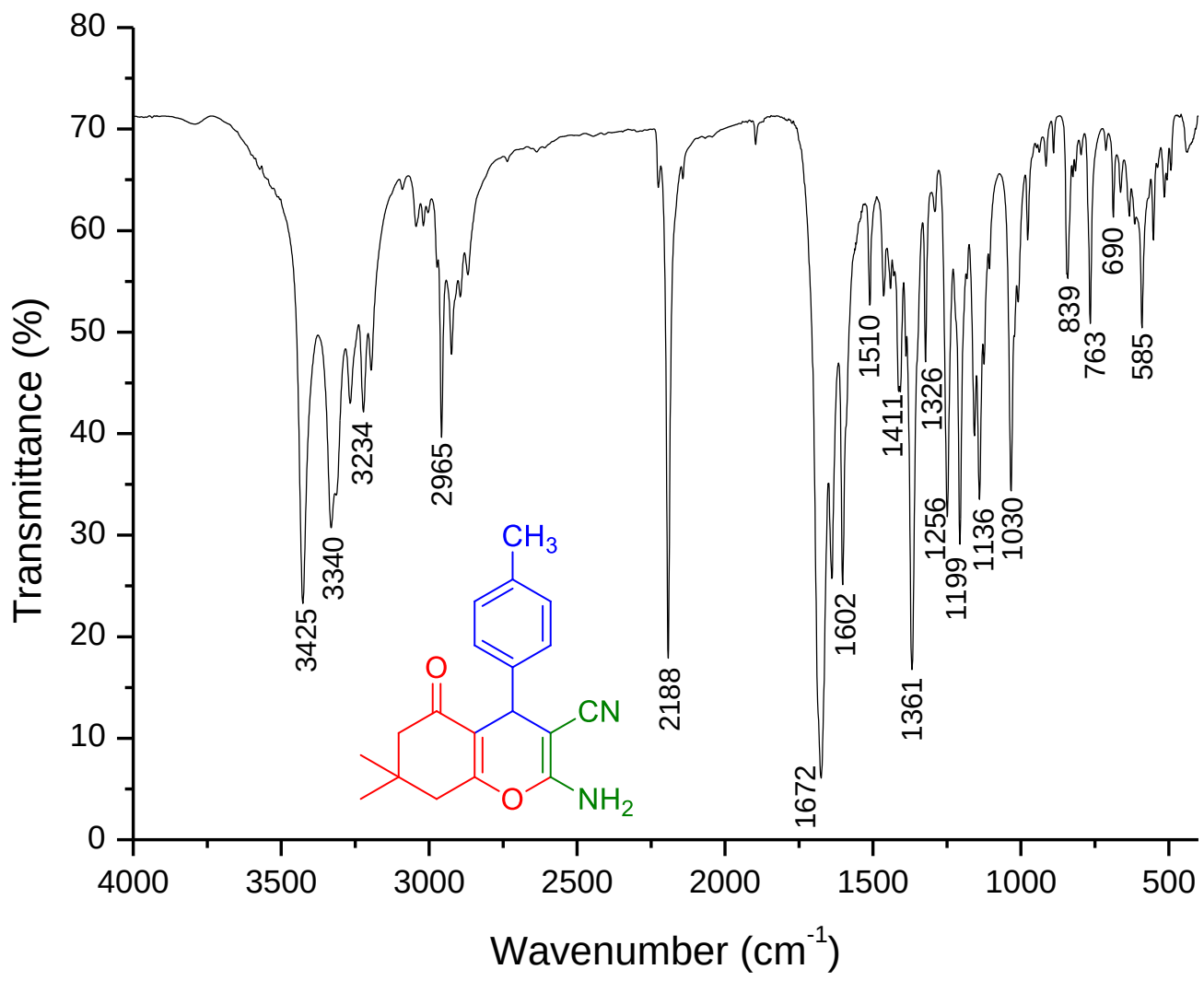

Figure S82. FT-IR (KBr) spectrum of compound **4l**.

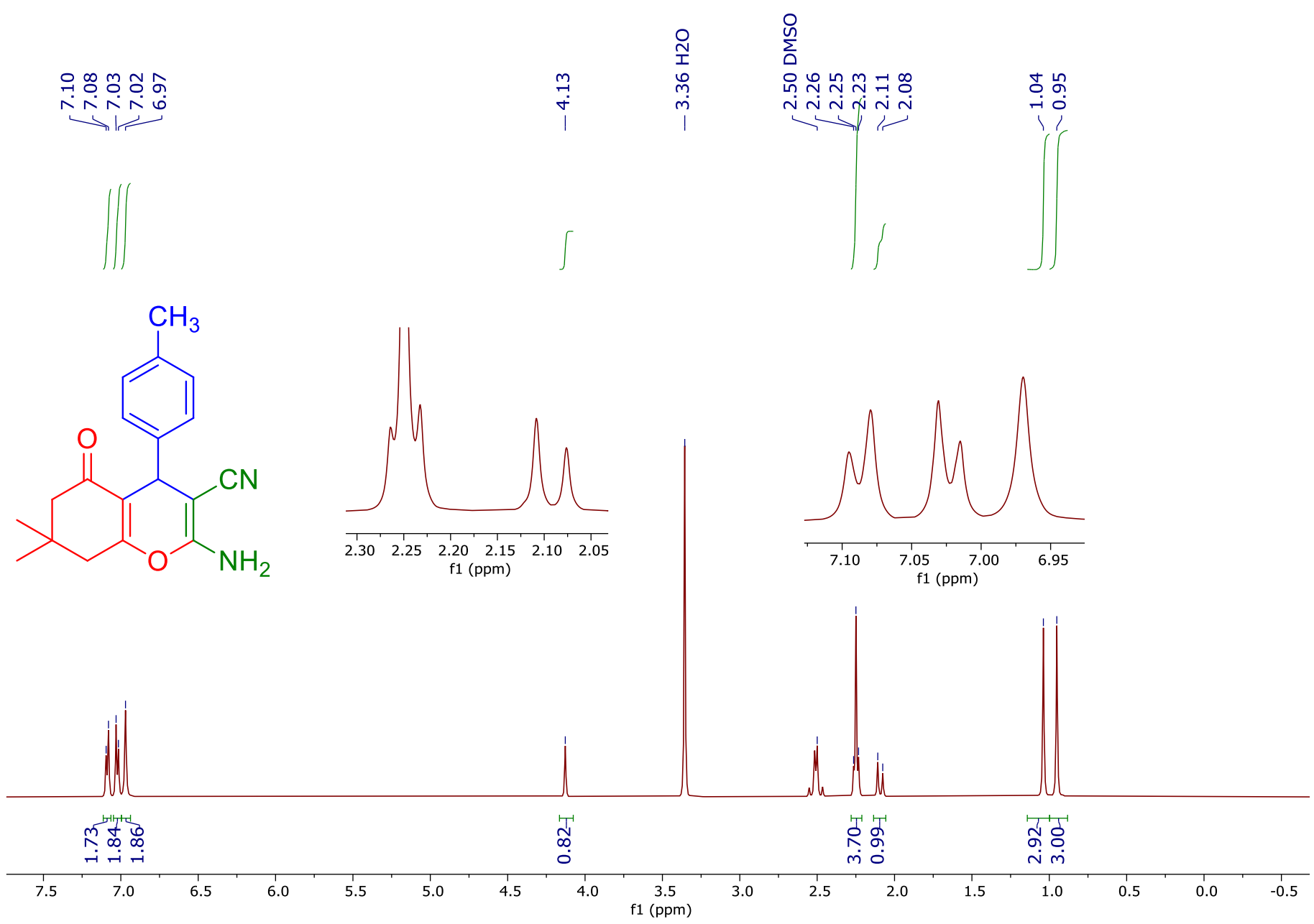

Figure S83. ^{1}H NMR spectrum (500 MHz, DMSO-d_6) of **4l**.

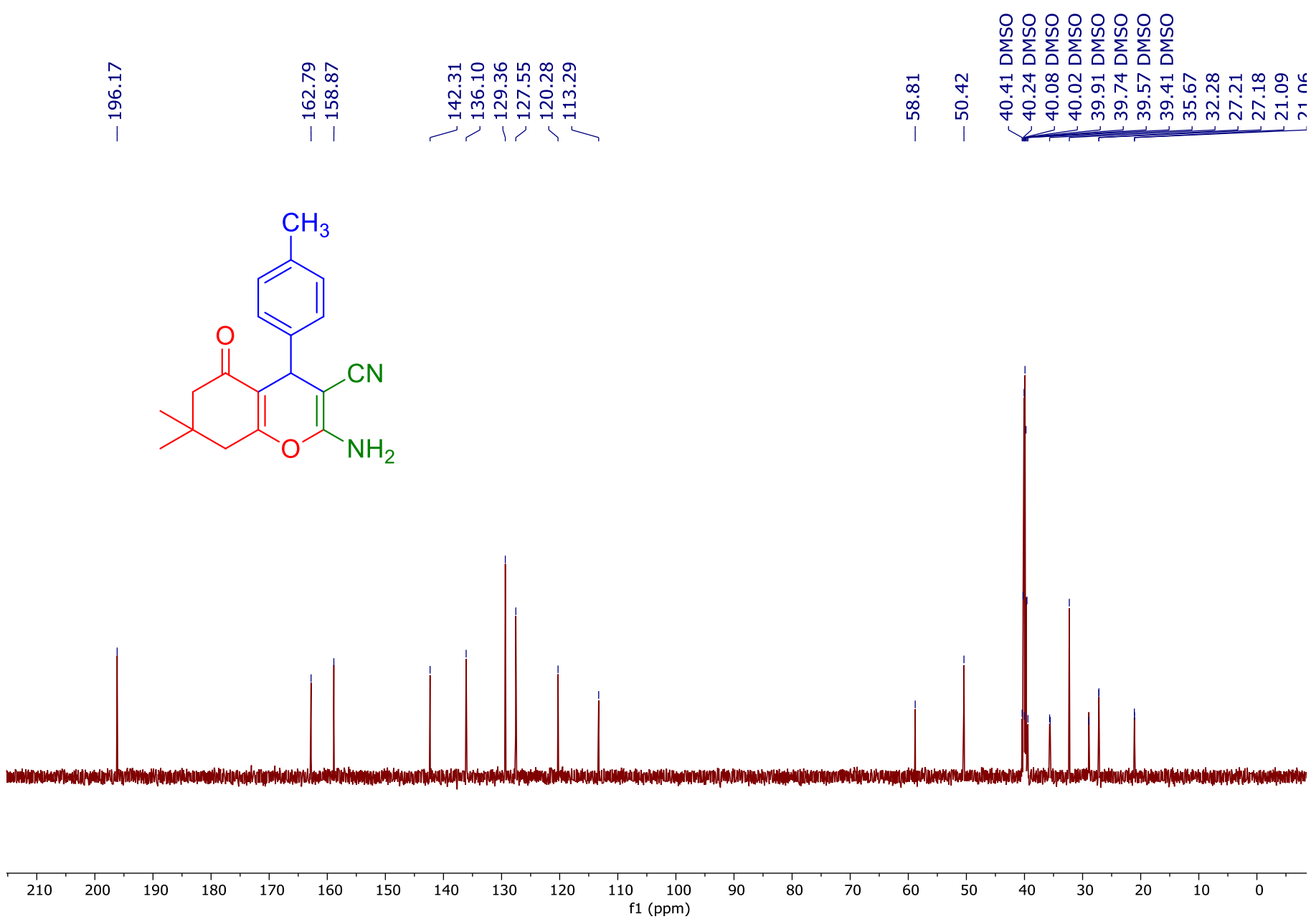

Figure S84. ^{13}C {^{1}H} NMR spectrum (125 MHz, DMSO-d$_6$) of **4l**.

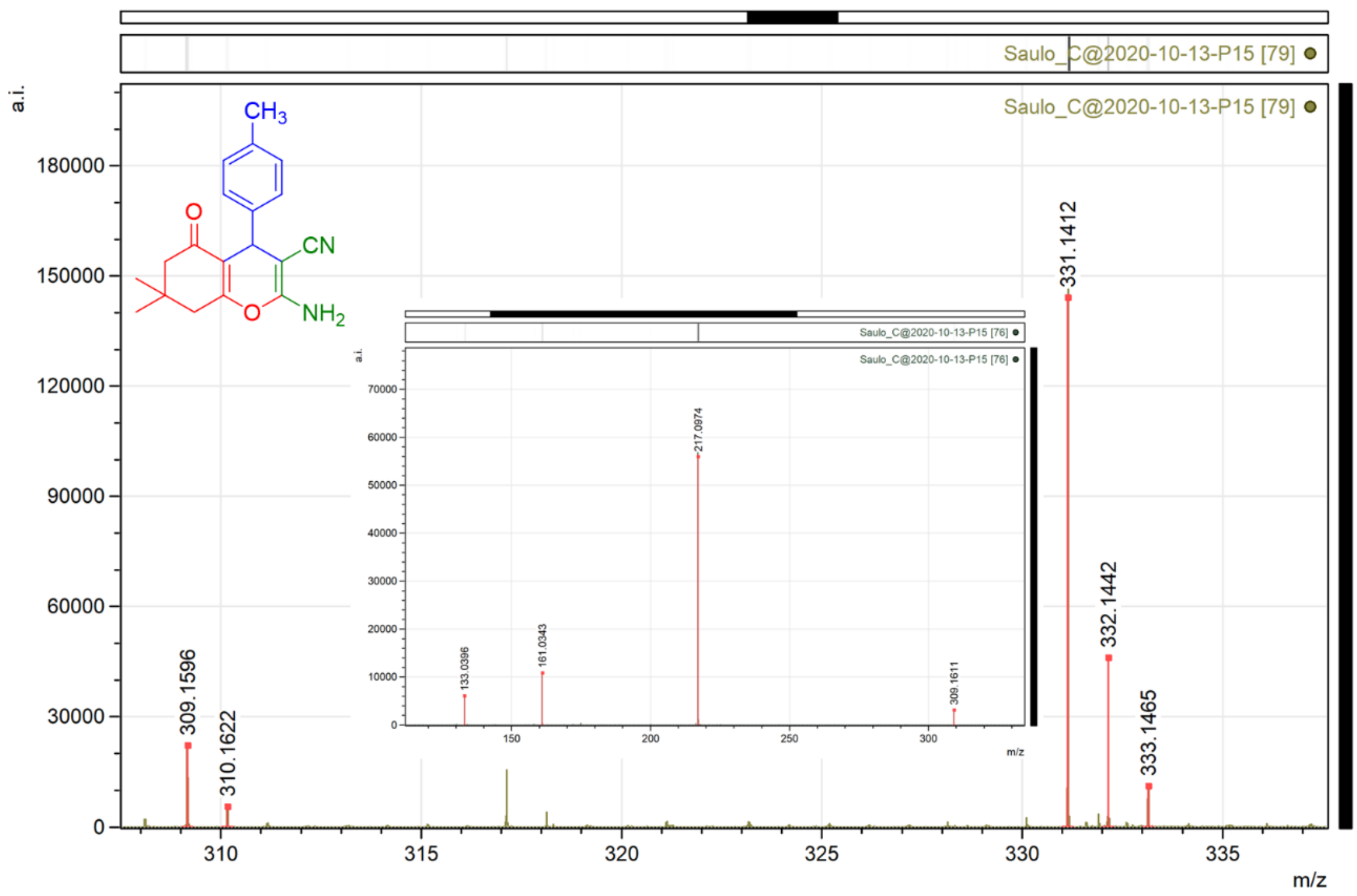

Figure S85. High-resolution ESI(+)-MS of **4l** showing its isotopic pattern. The inset is the ESI(+)-MS/MS from the $[M+H]^+$ isotopologue.

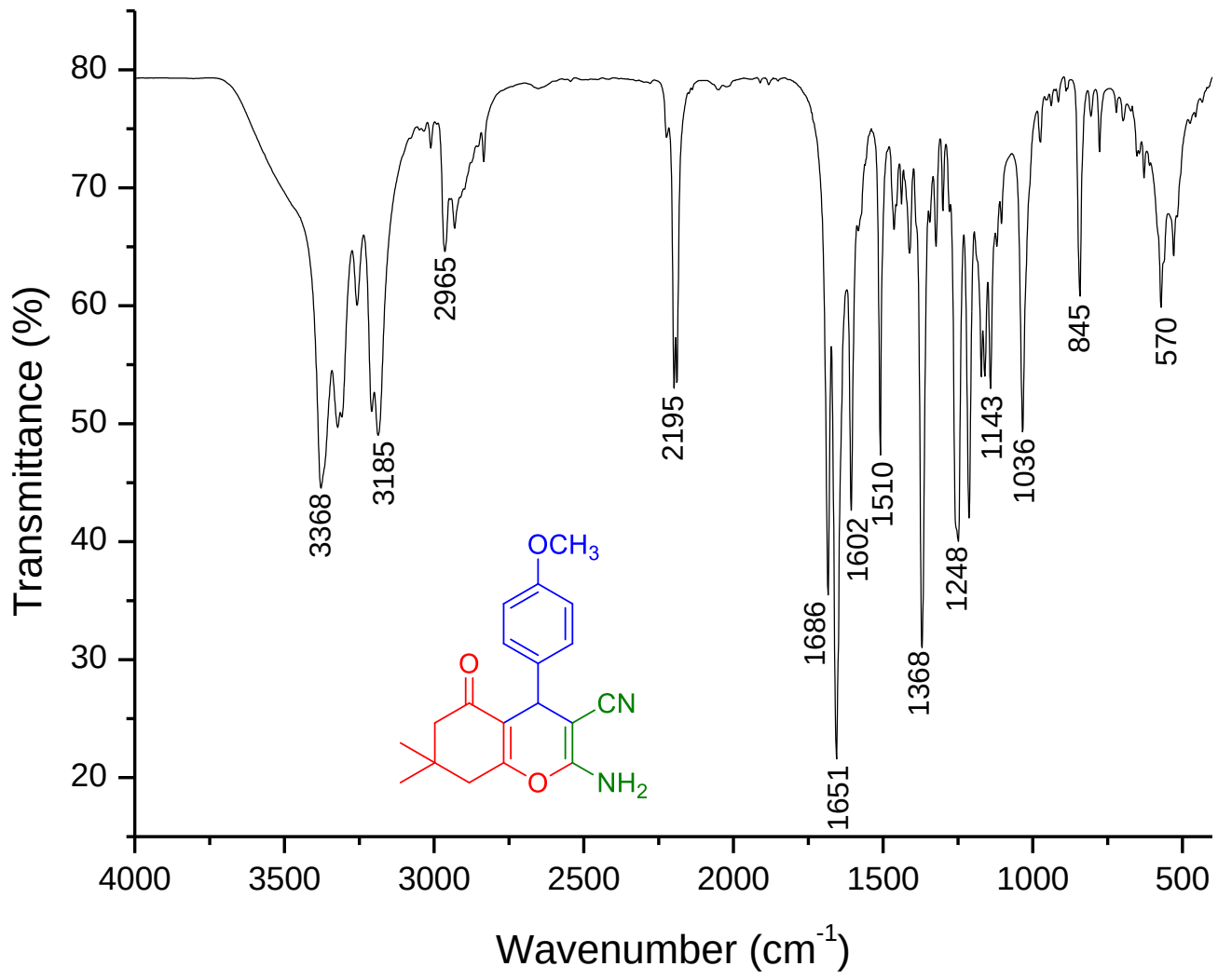

Figure S86. FT-IR (KBr) spectrum of compound **4m**.

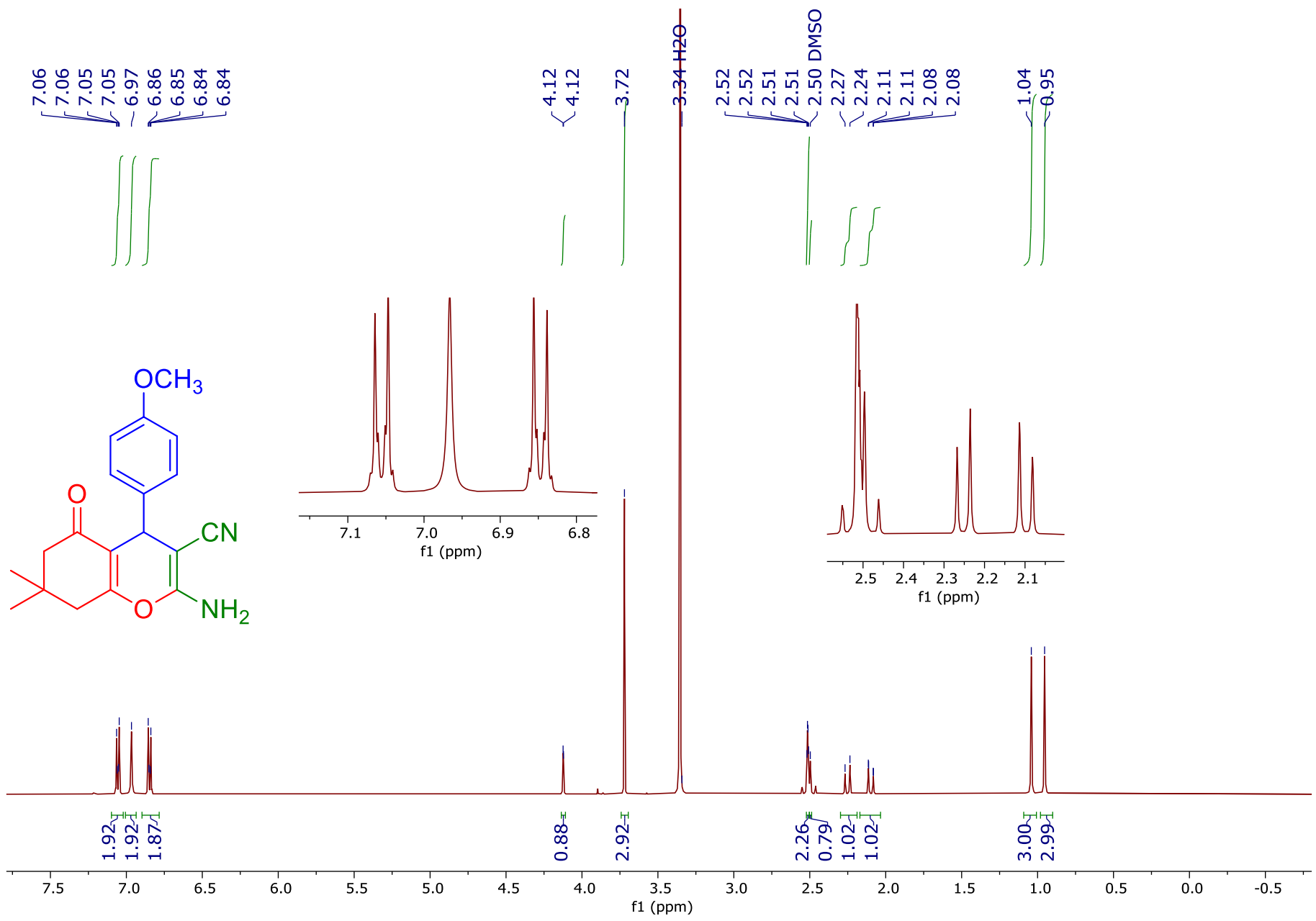

Figure S87. ^{1}H NMR spectrum (500 MHz, DMSO-d_6) of **4m**.

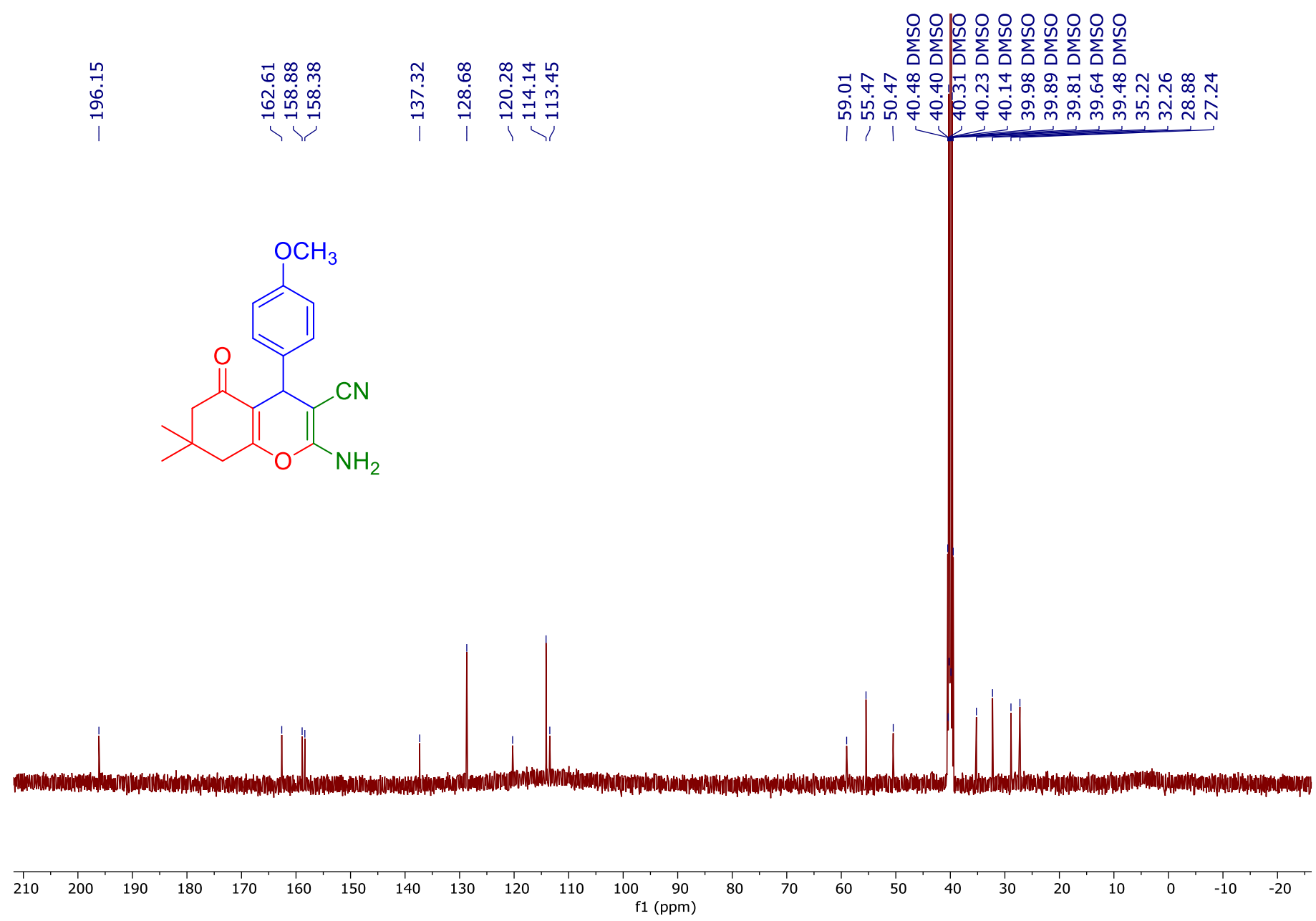

Figure S88. ^{13}C {^1H} NMR spectrum (125 MHz, DMSO-d_6) of **4m**. One carbon signal overlaps with the solvent signals.

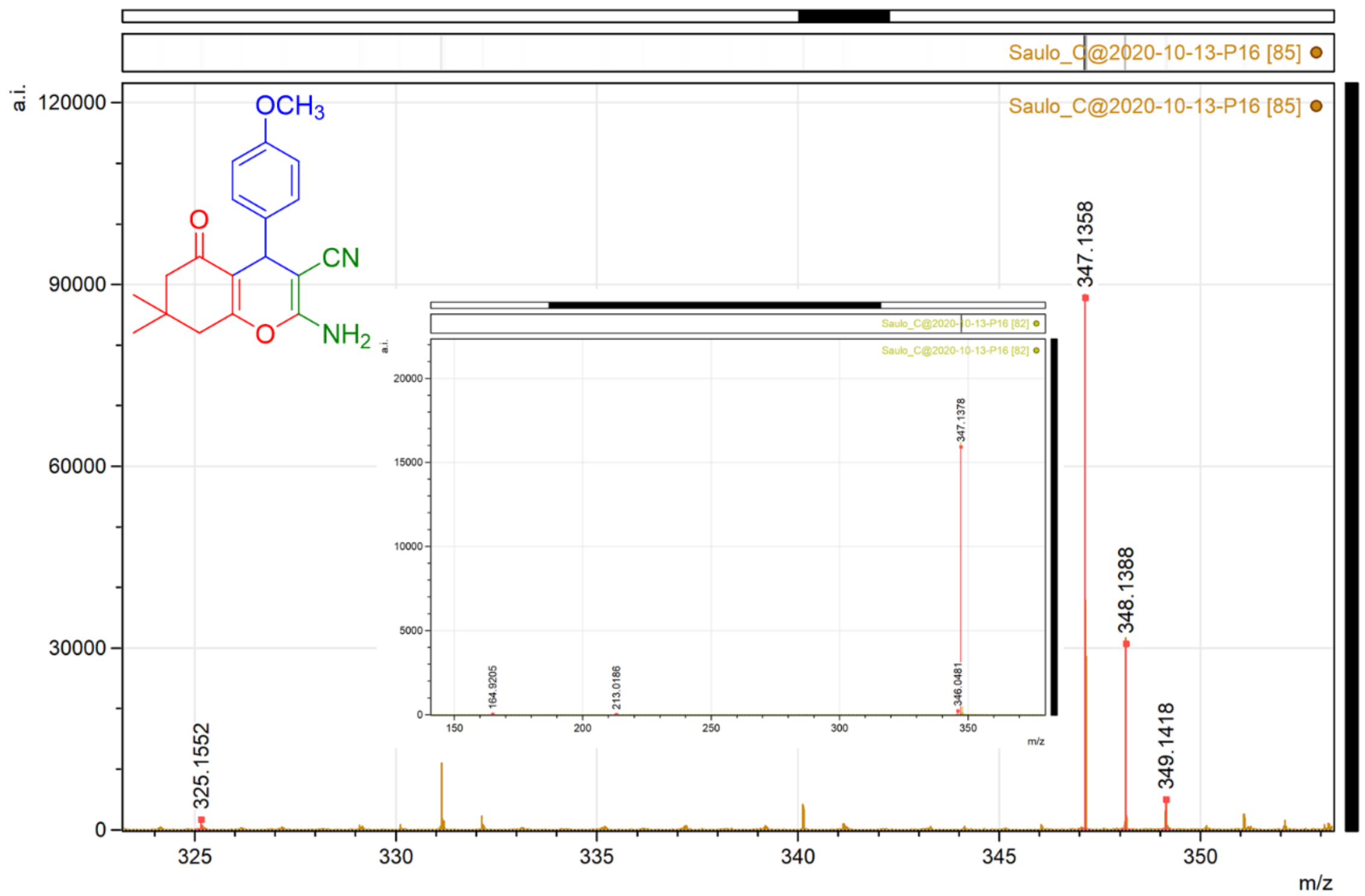

Figure S89. High-resolution ESI(+)-MS of **4m** showing its isotopic pattern. The inset is the ESI(+)-MS/MS from the [M+Na]$^+$ isotopologue.

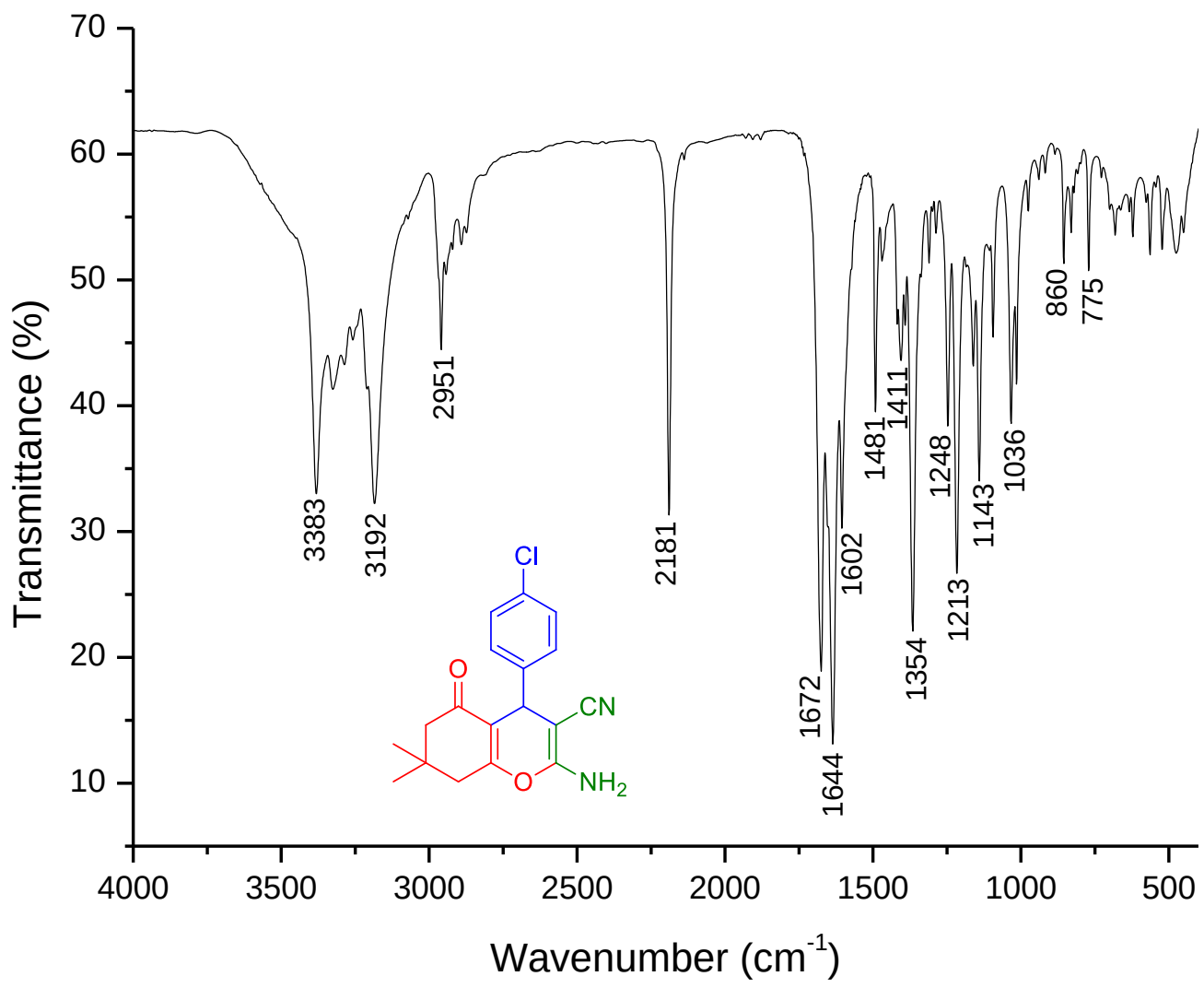

Figure S90. FT-IR (KBr) spectrum of compound **4n**.

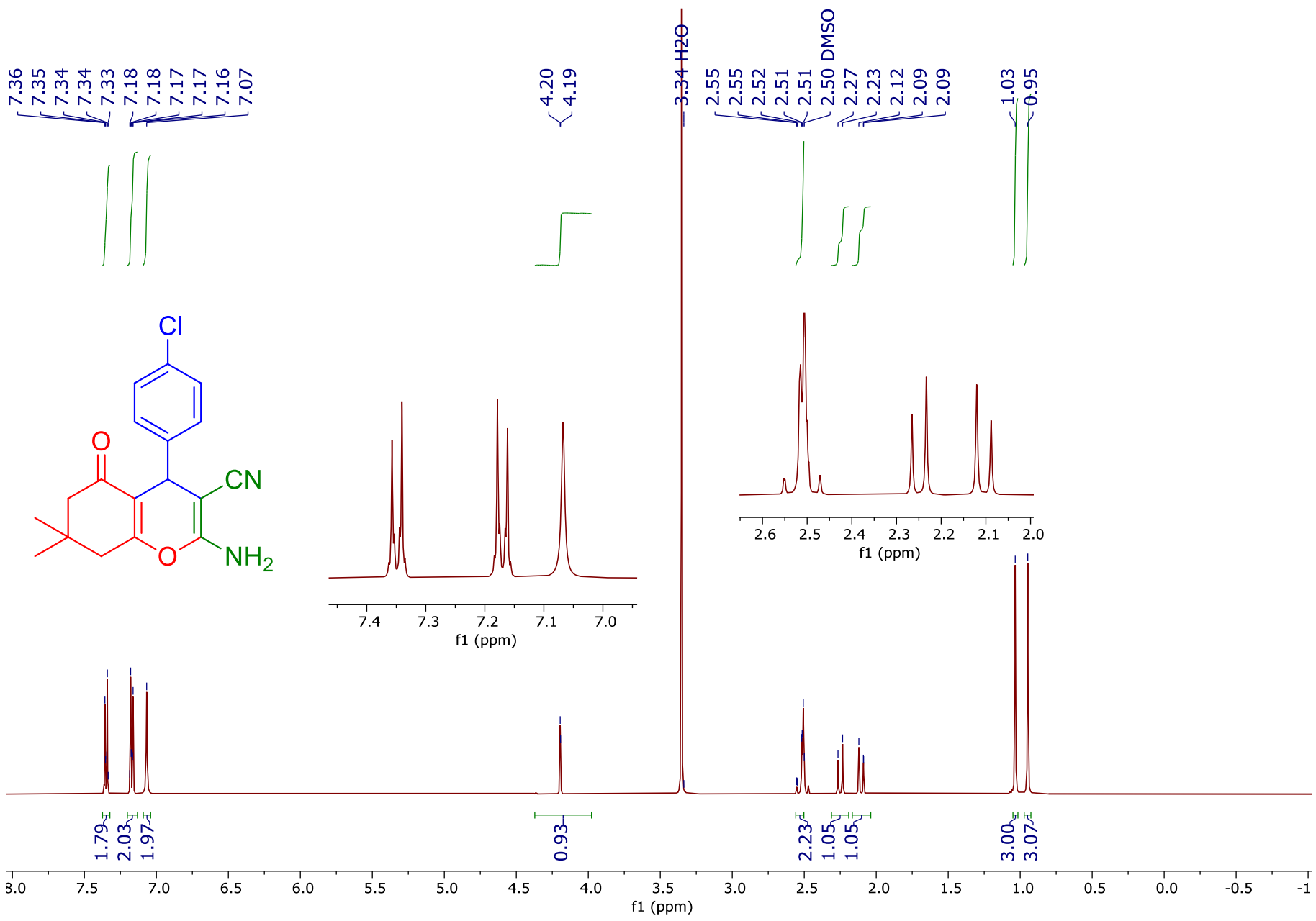

Figure S91. ^{1}H NMR spectrum (500 MHz, DMSO-d_6) of **4n**.

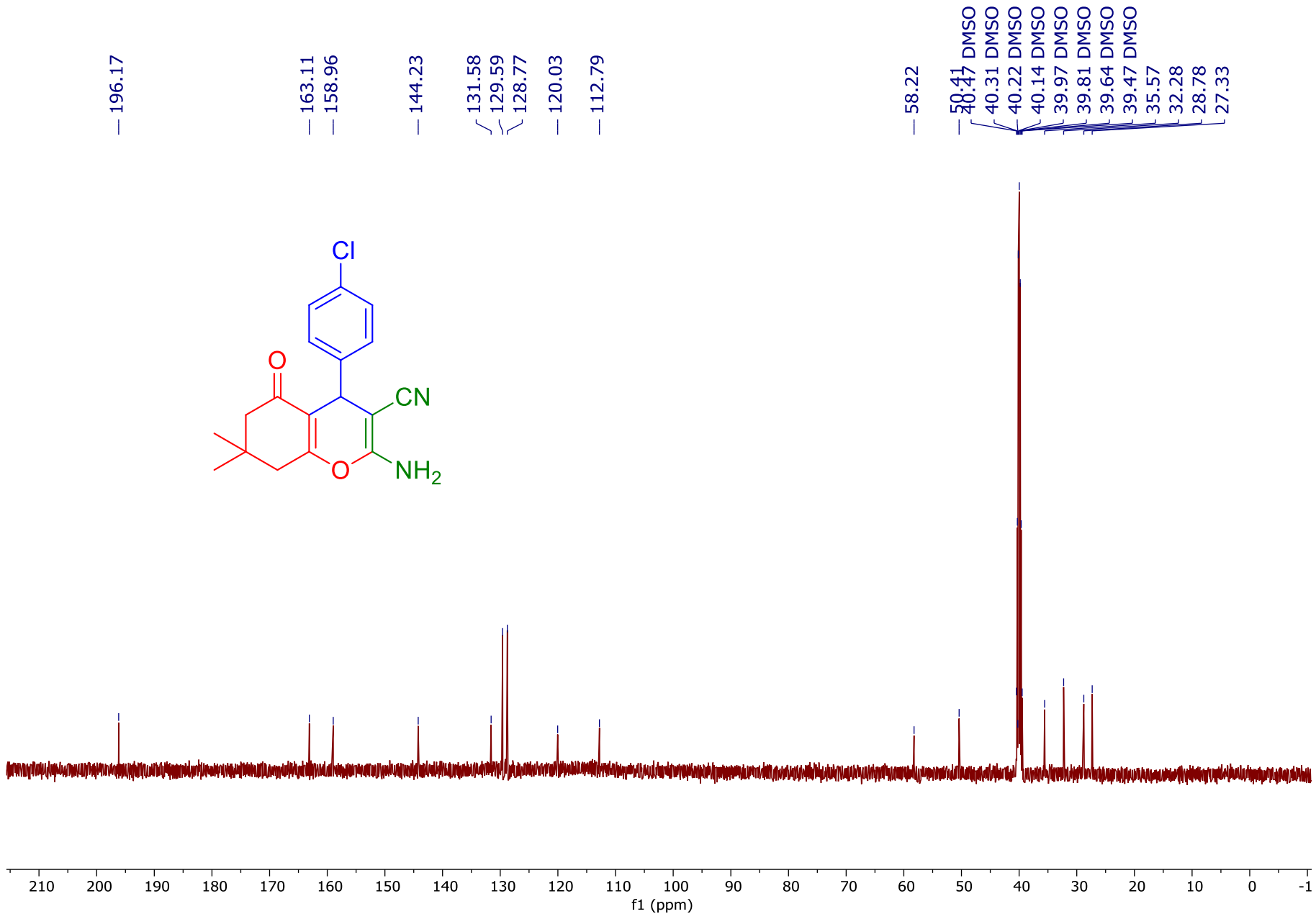

Figure S92. $^{13}C\{^1H\}$ NMR spectrum (125 MHz, DMSO-d_6) of **4n**. One carbon signal overlaps with the solvent signals.

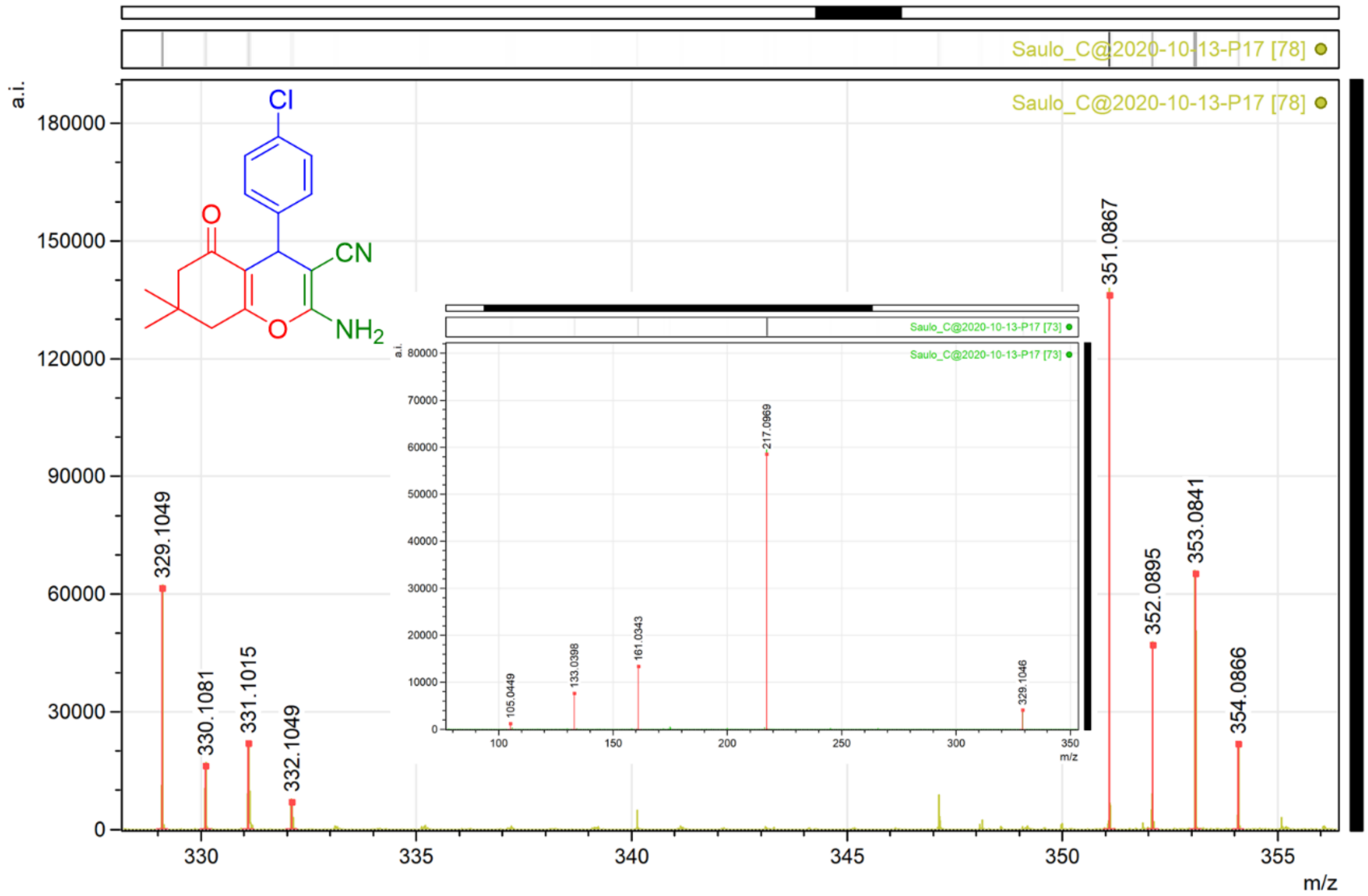

Figure S93. High-resolution ESI(+)-MS of **4n** showing its isotopic pattern. The inset is the ESI(+)-MS/MS from the $[M+H]^+$ isotopologue.

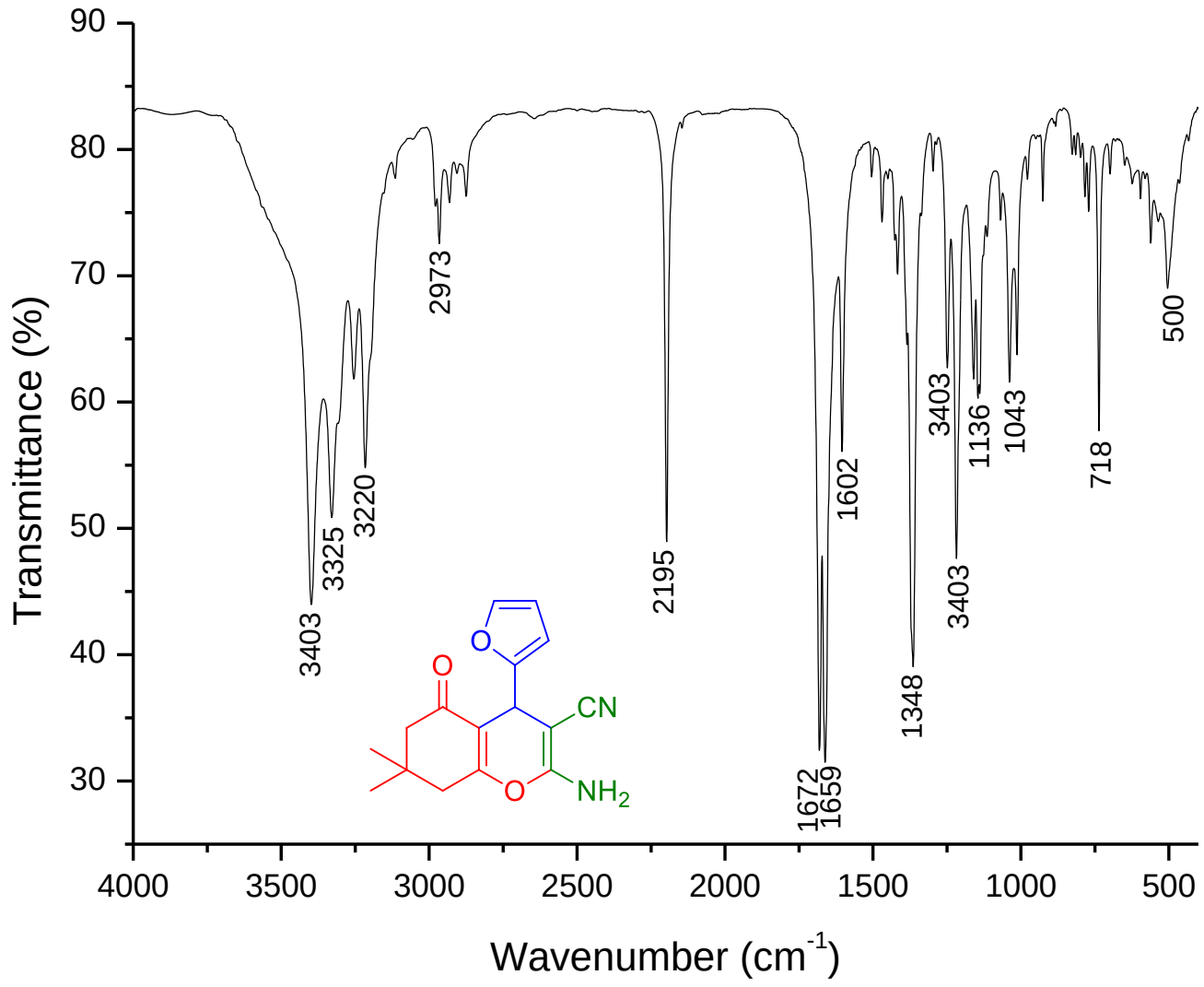

Figure S94. FT-IR (KBr) spectrum of compound **4o**.

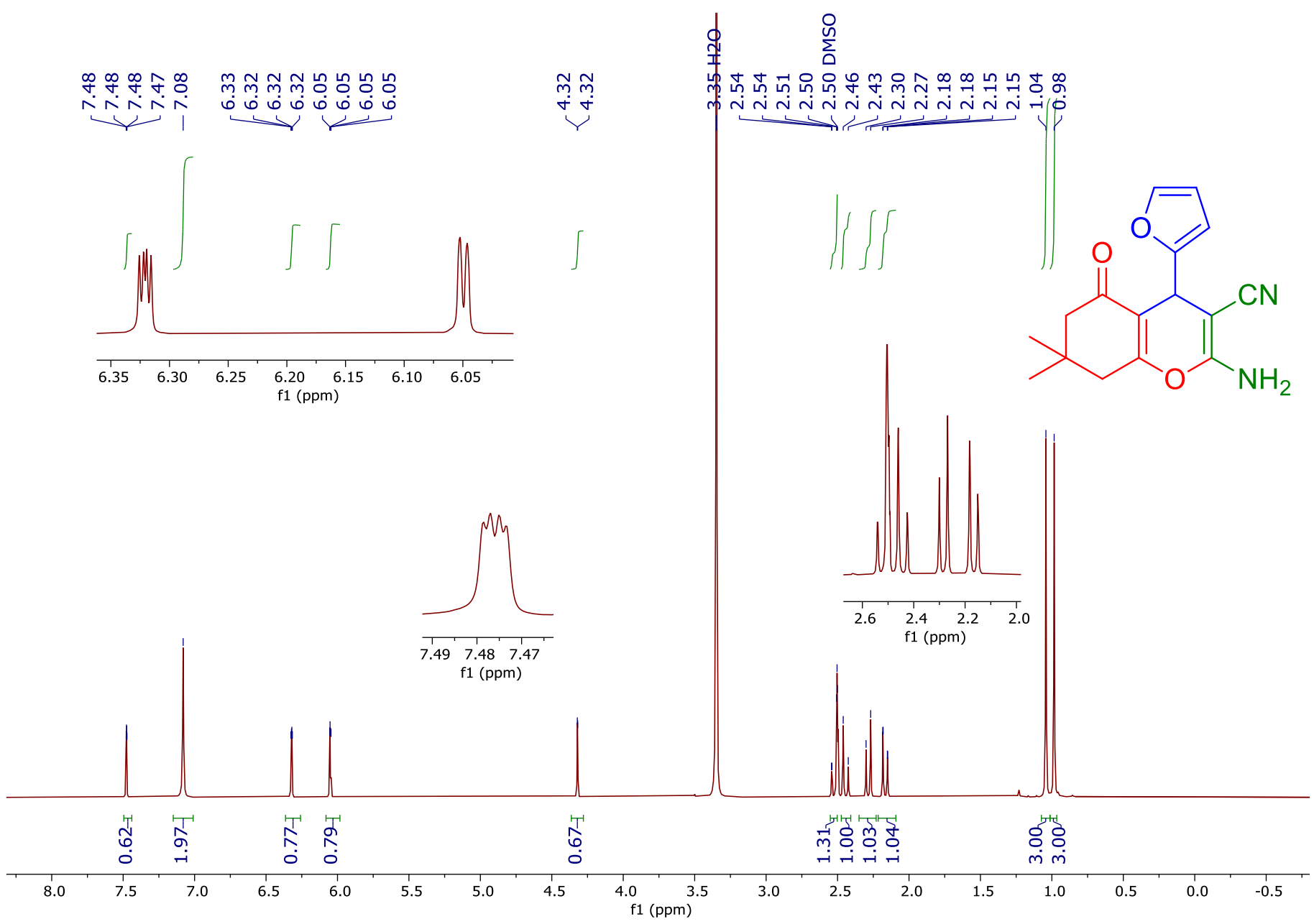

Figure S95. ^{1}H NMR spectrum (500 MHz, DMSO-d$_6$) of **4o**.

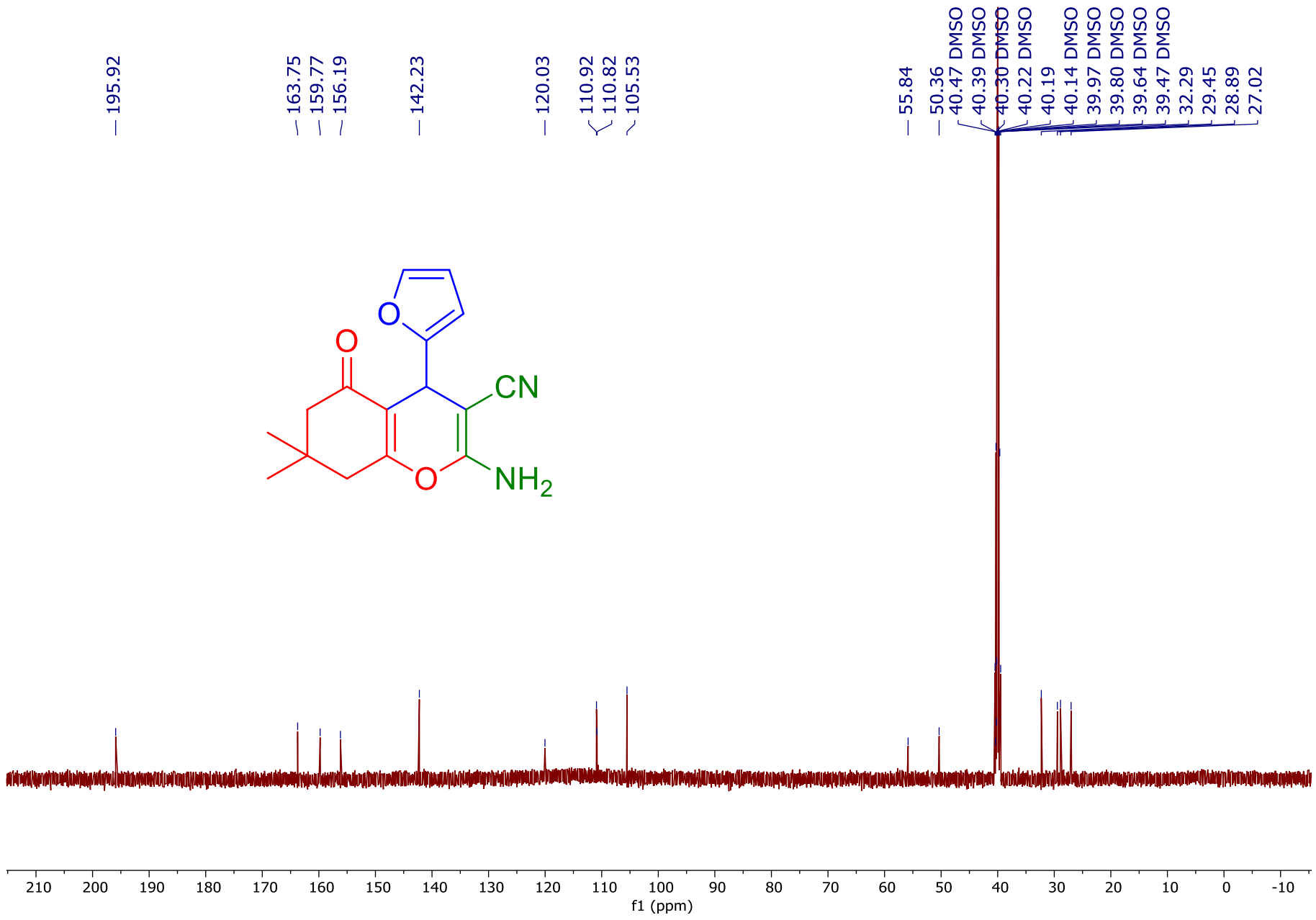

Figure S96. $^{13}C\{^{1}H\}$ NMR spectrum (125 MHz, DMSO-d_6) of **4o**.

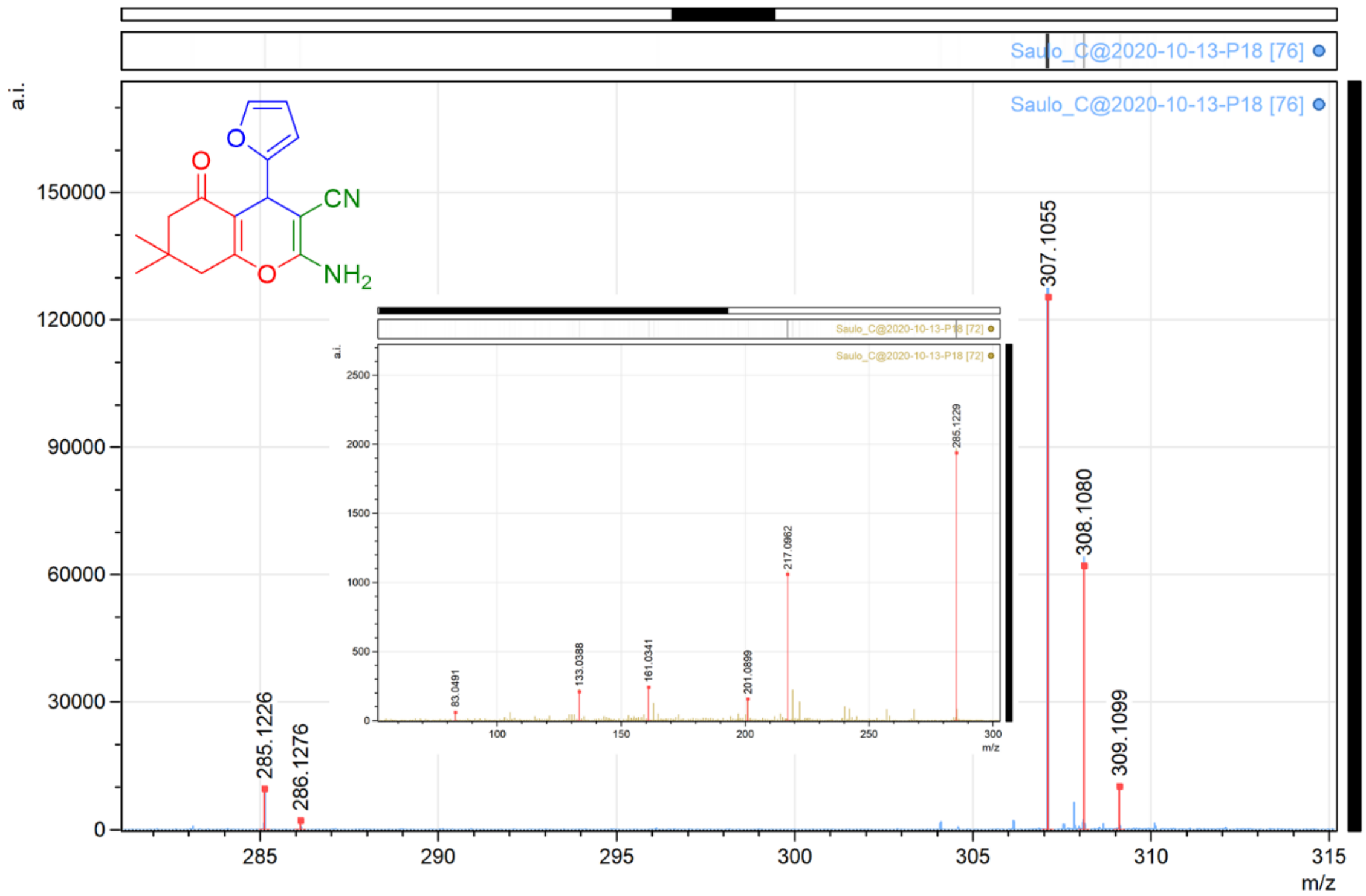

Figure S97. High-resolution ESI(+)-MS of **4o** showing its isotopic pattern. The inset is the ESI(+)-MS/MS from the $[M+H]^+$ isotopologue.

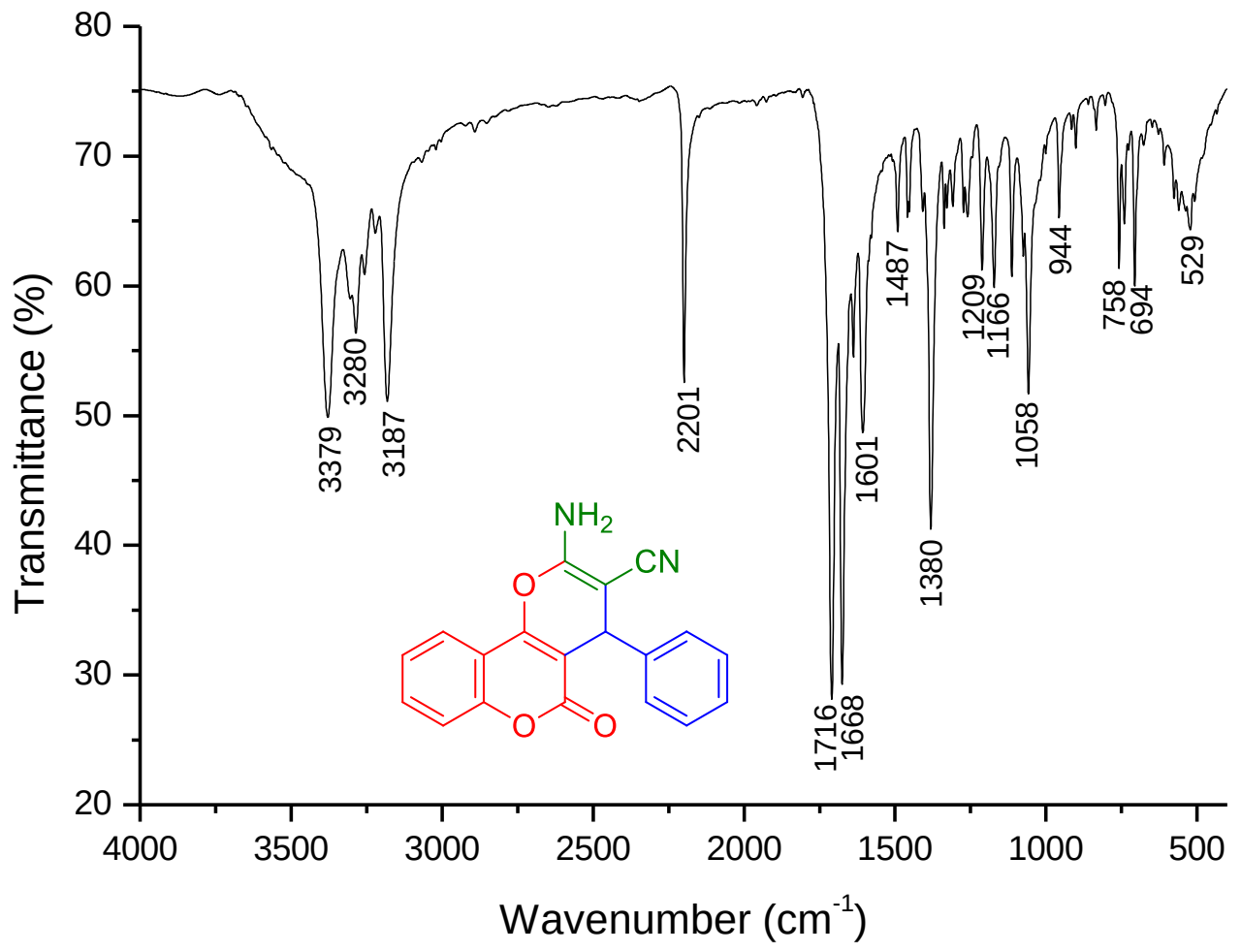

Figure S98. FT-IR (KBr) spectrum of compound **4p**.

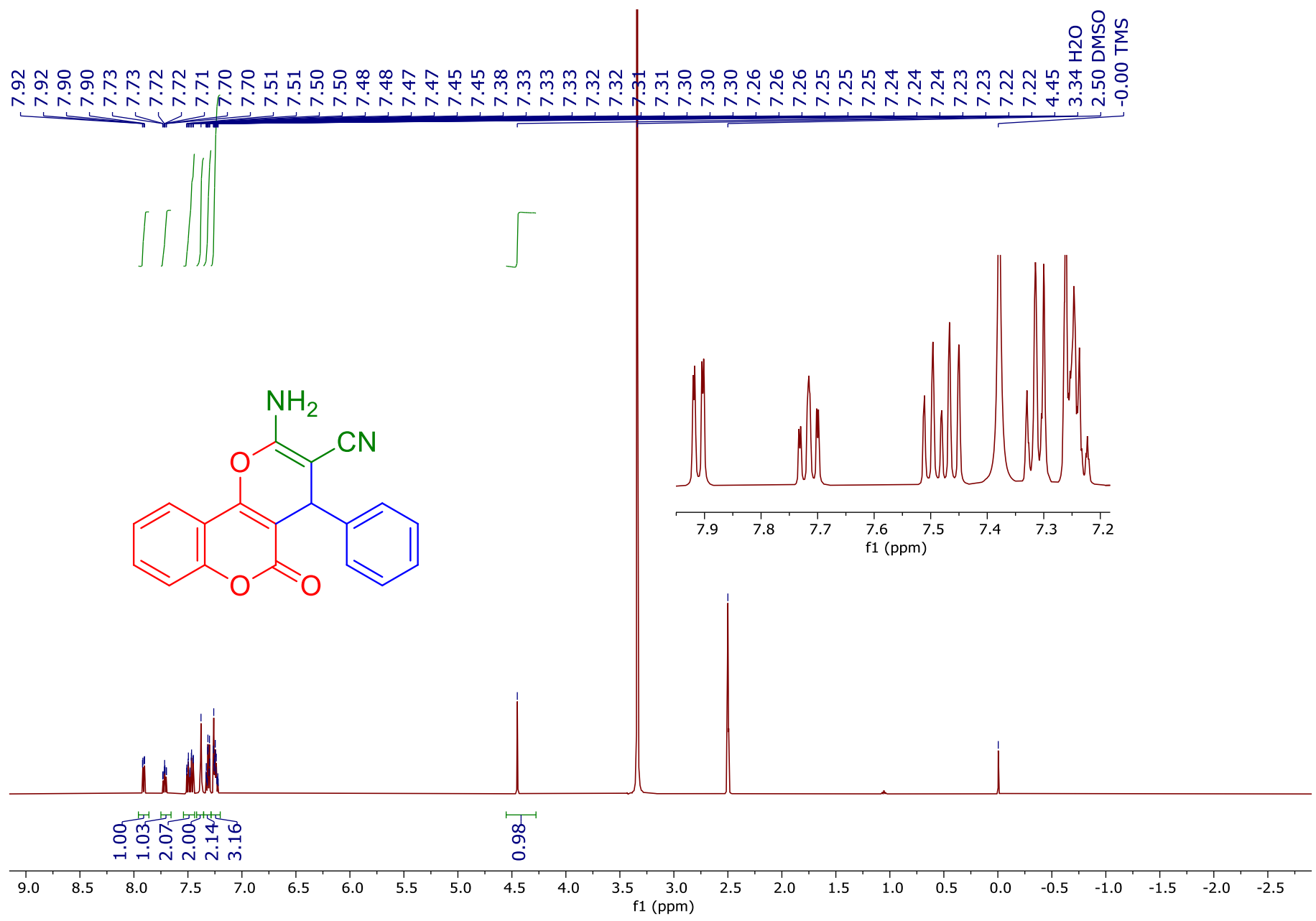

Figure S99. ^{1}H NMR spectrum (500 MHz, DMSO-d$_6$) of **4p**.

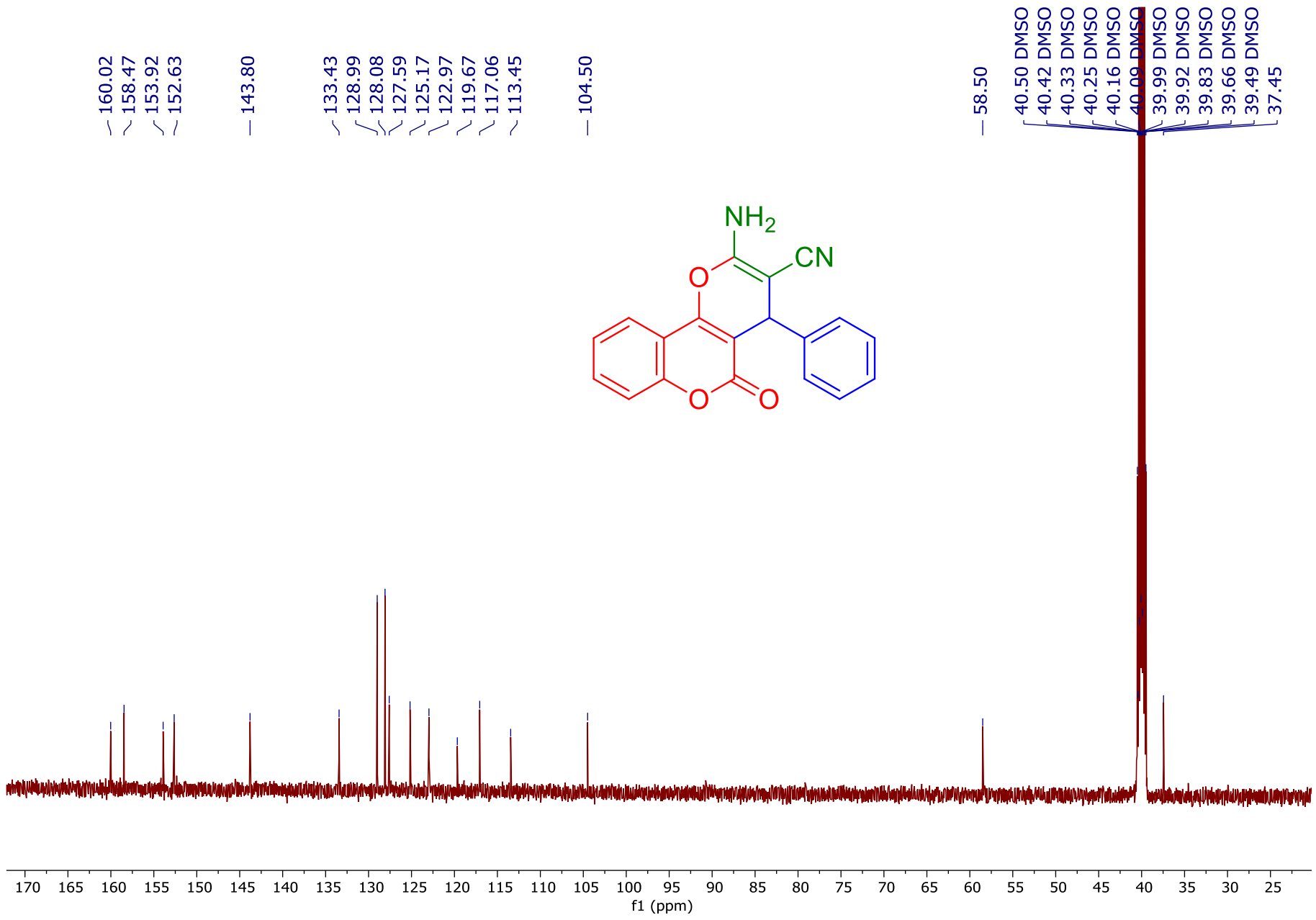

Figure S100. $^{13}C\ \{^{1}H\}$ NMR spectrum (125 MHz, DMSO-d$_6$) of **4p**.

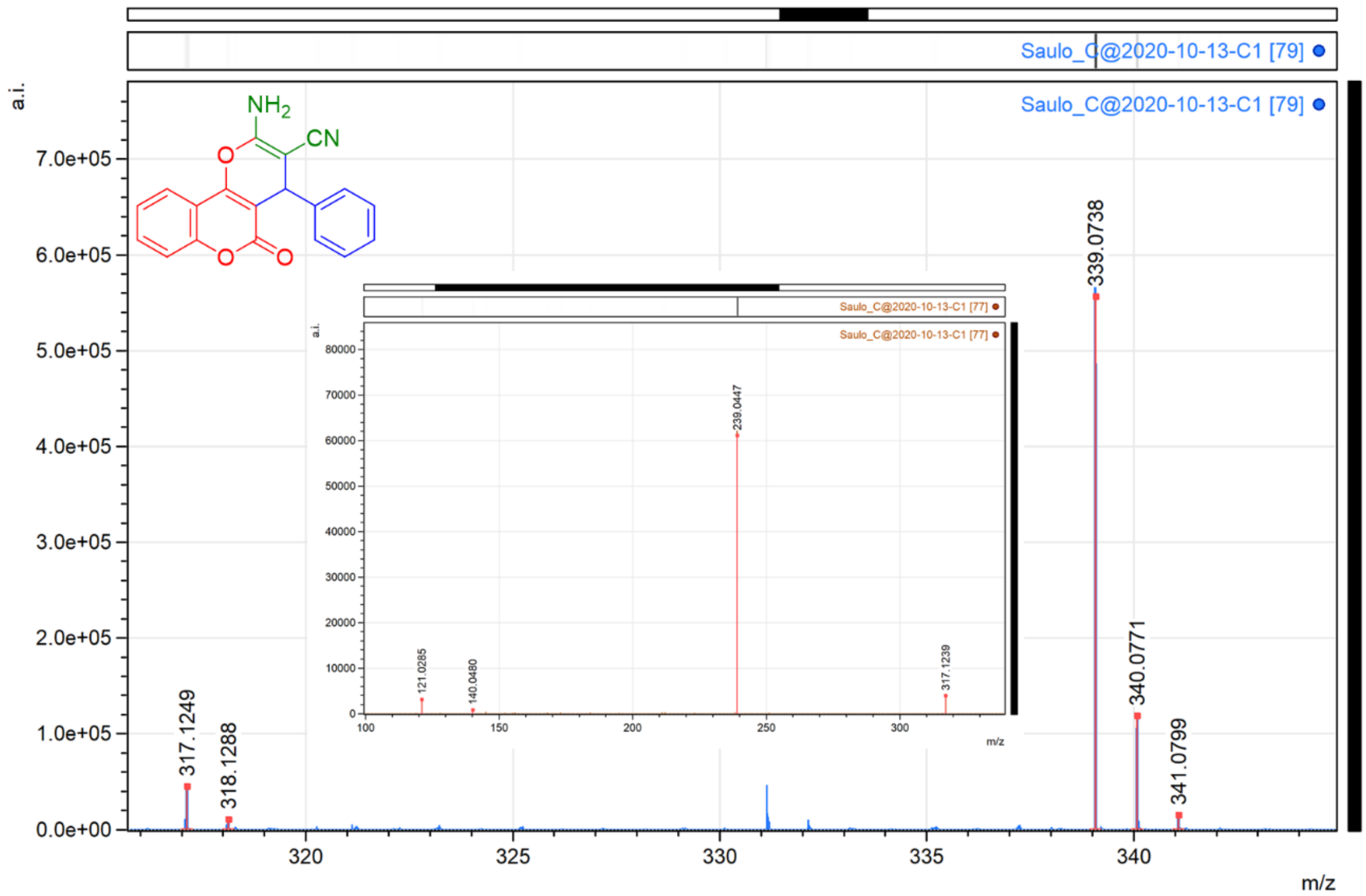

Figure S101. High-resolution ESI(+)-MS of **4p** showing its isotopic pattern. The inset is the ESI(+)-MS/MS from the $[M+H]^+$ isotopologue.

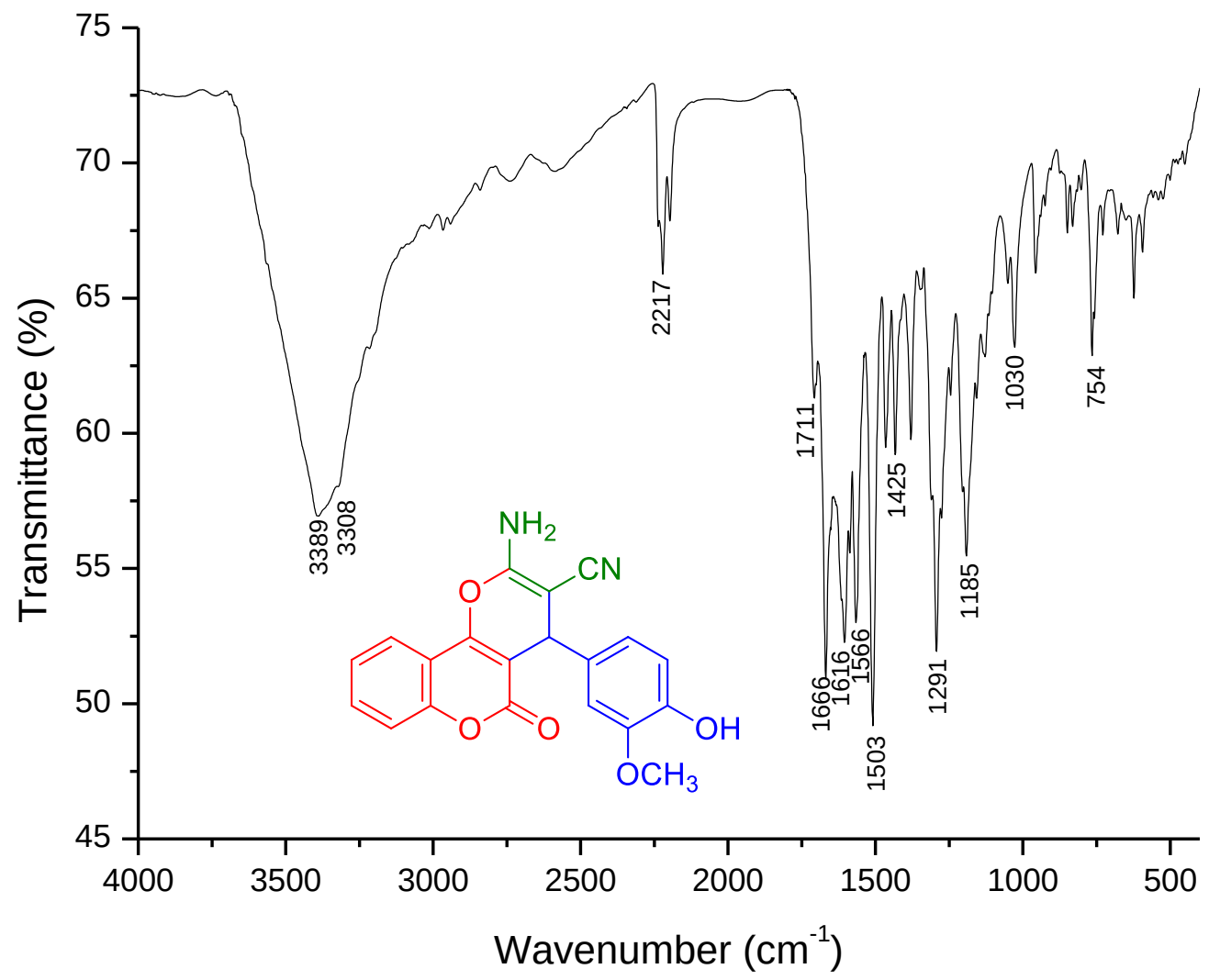

Figure S102. FT-IR (KBr) spectrum of compound **4q**.

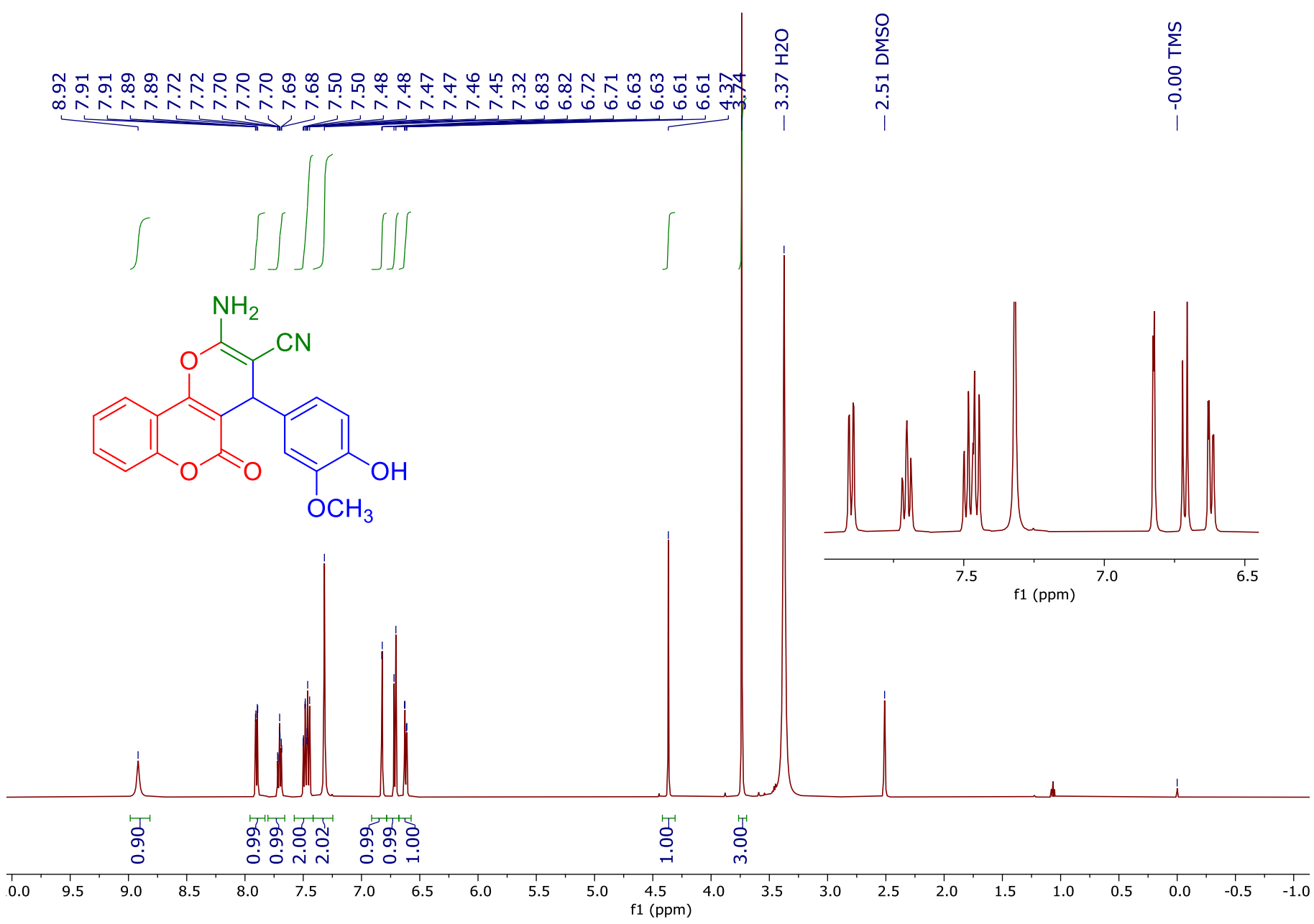

Figure S103. ^{1}H NMR spectrum (500 MHz, DMSO-d*6*) of **4q**.

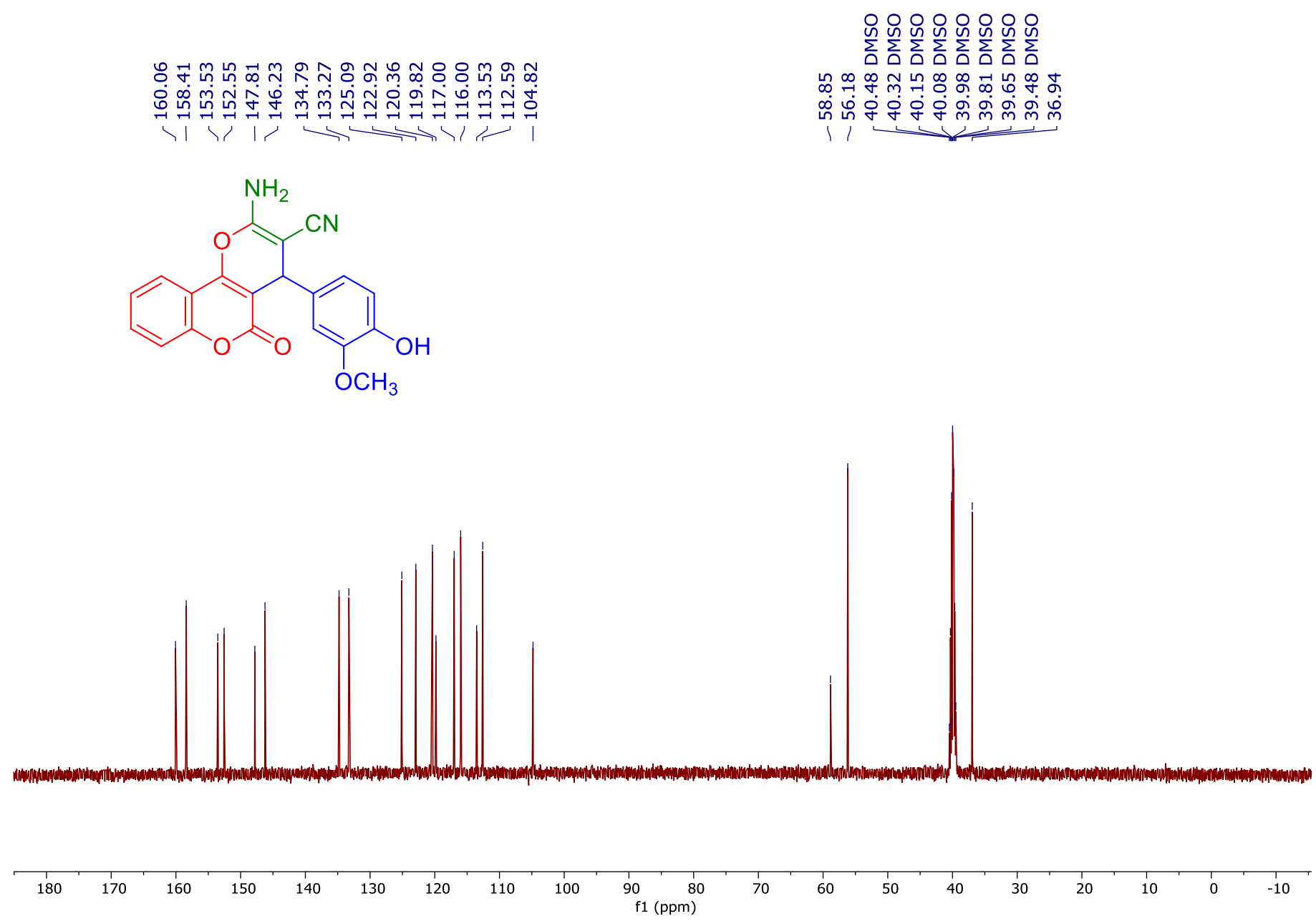

Figure S104. ^{13}C NMR spectrum (125 MHz, DMSO-d$_6$) of **4q**.

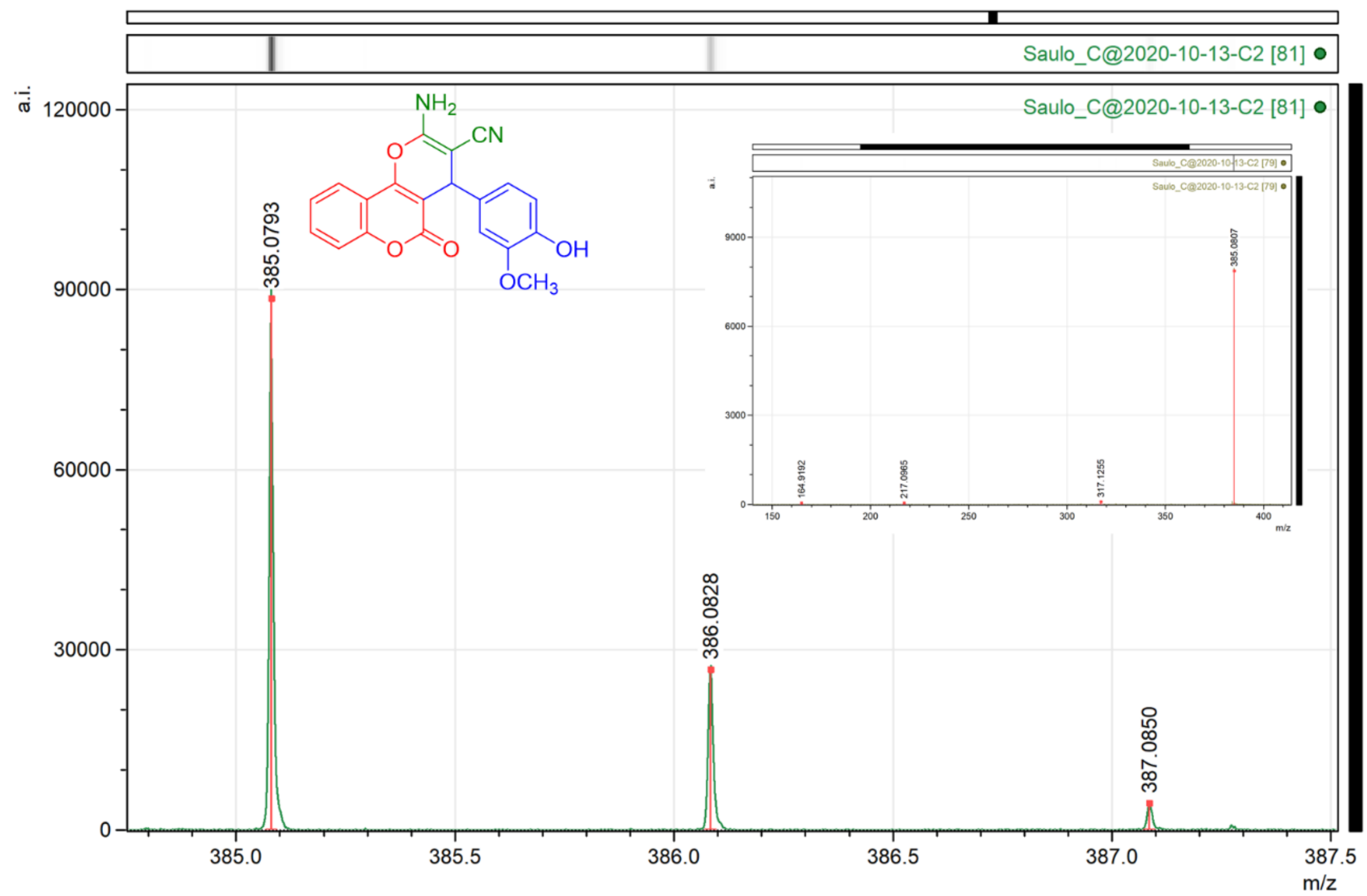

Figure S105. High-resolution ESI(+)-MS of **4q** showing its isotopic pattern. The inset is the ESI(+)-MS/MS from the [M+Na]$^+$ isotopologue.

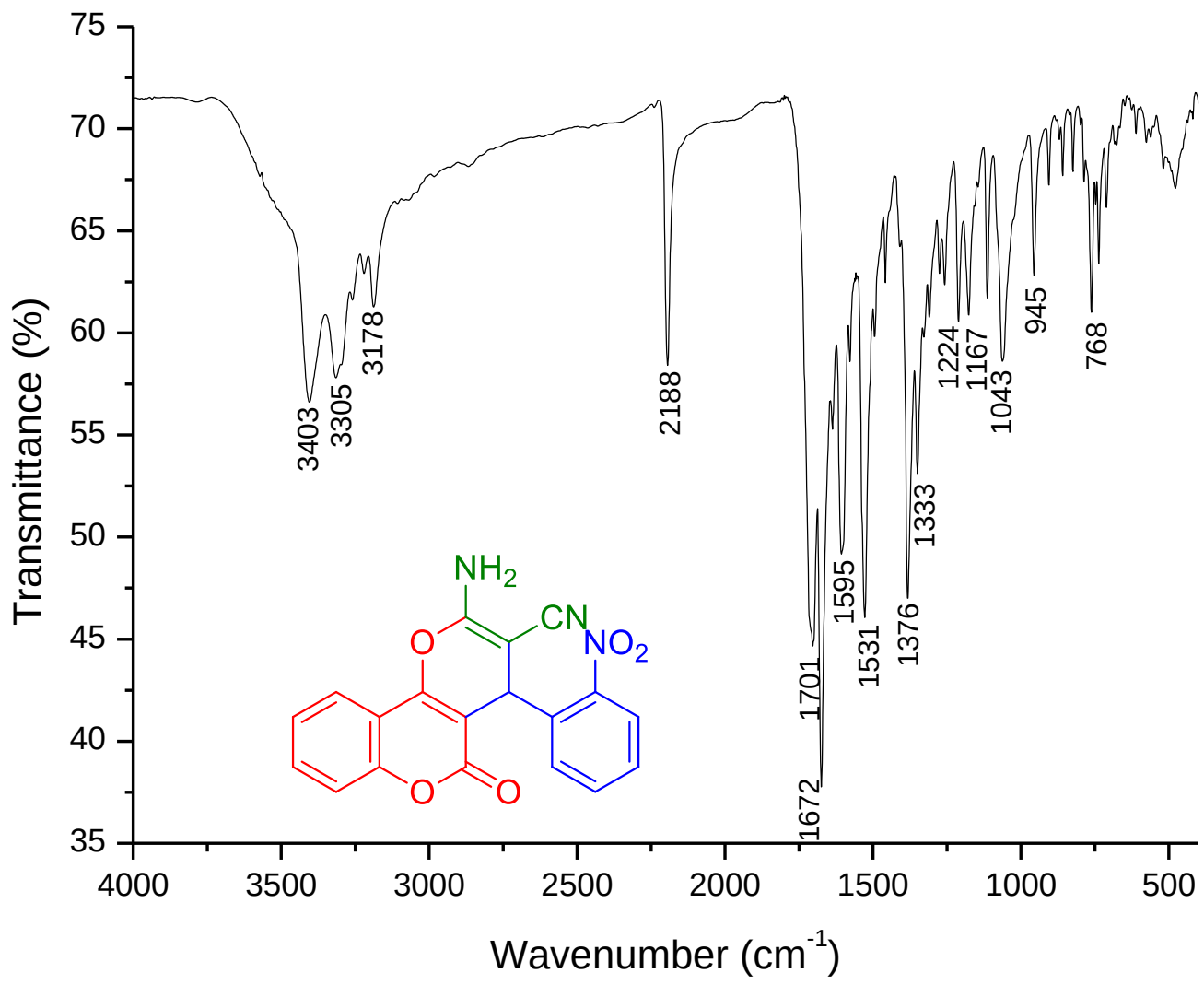

Figure S106. FT-IR (KBr) spectrum of compound **4r**.

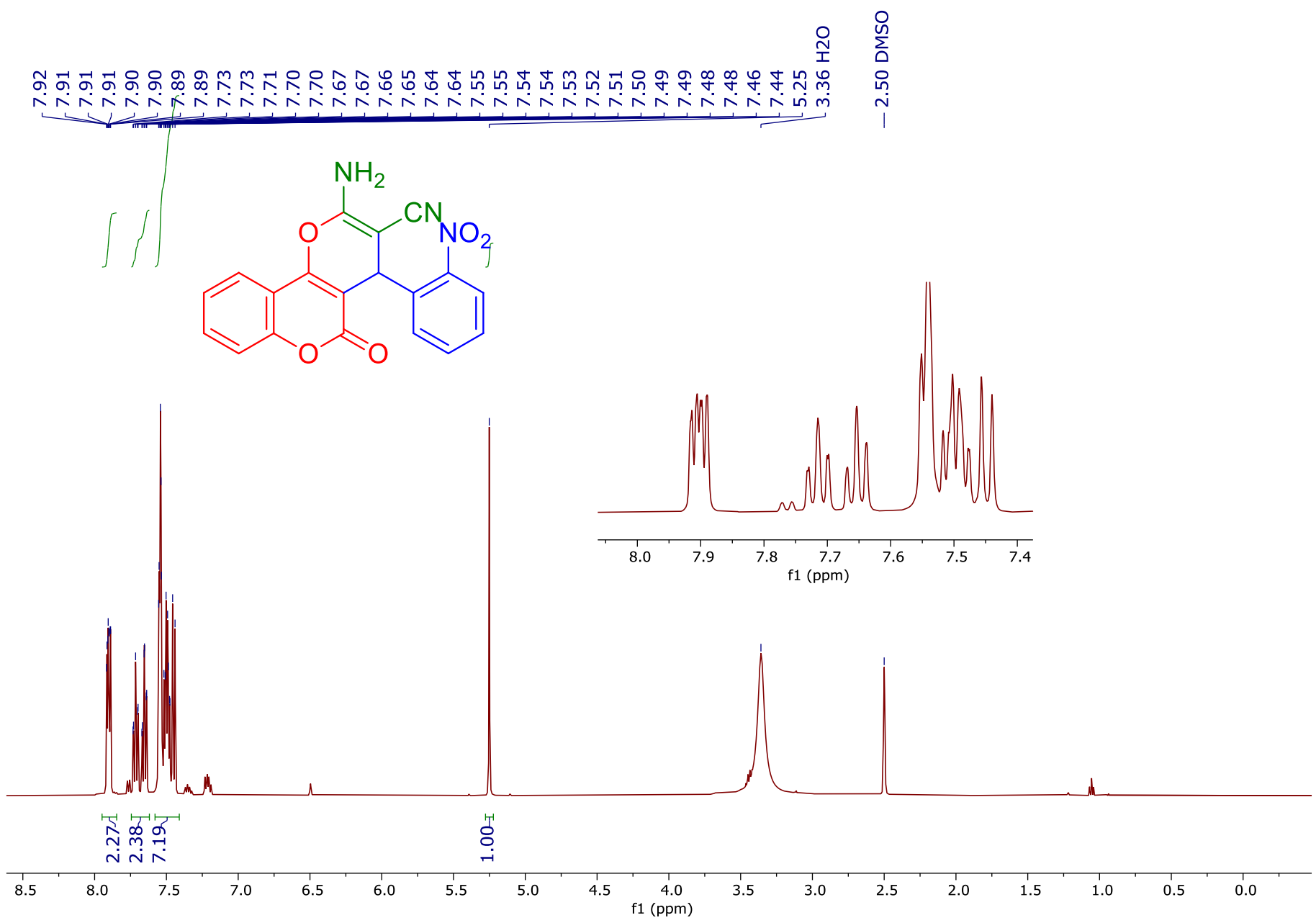

Figure S107. ^{1}H NMR spectrum (500 MHz, DMSO-d_6) of **4r**.

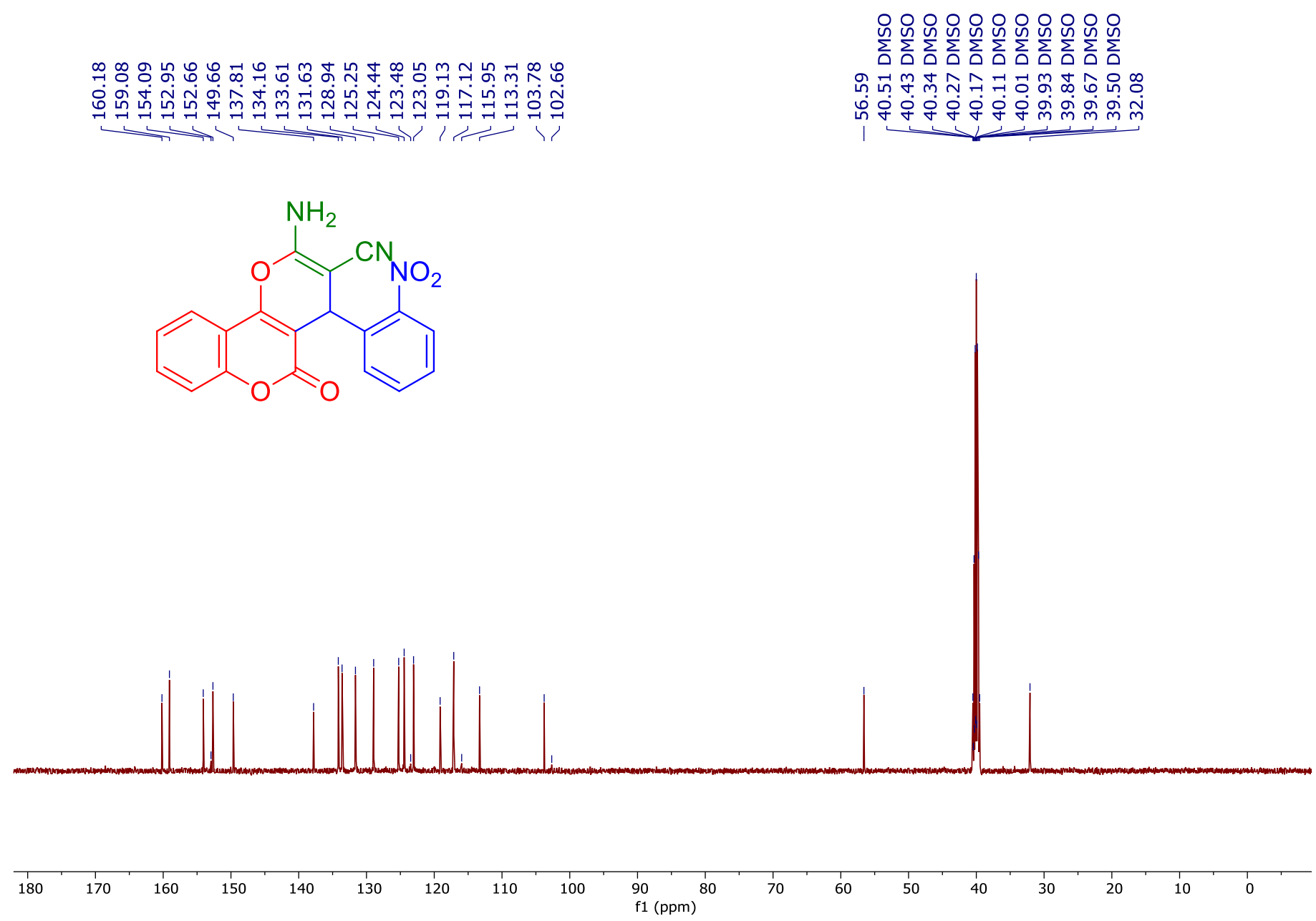

Figure S108. $^{13}C\ \{^{1}H\}$ NMR spectrum (125 MHz, DMSO-d_6) of **4r**.

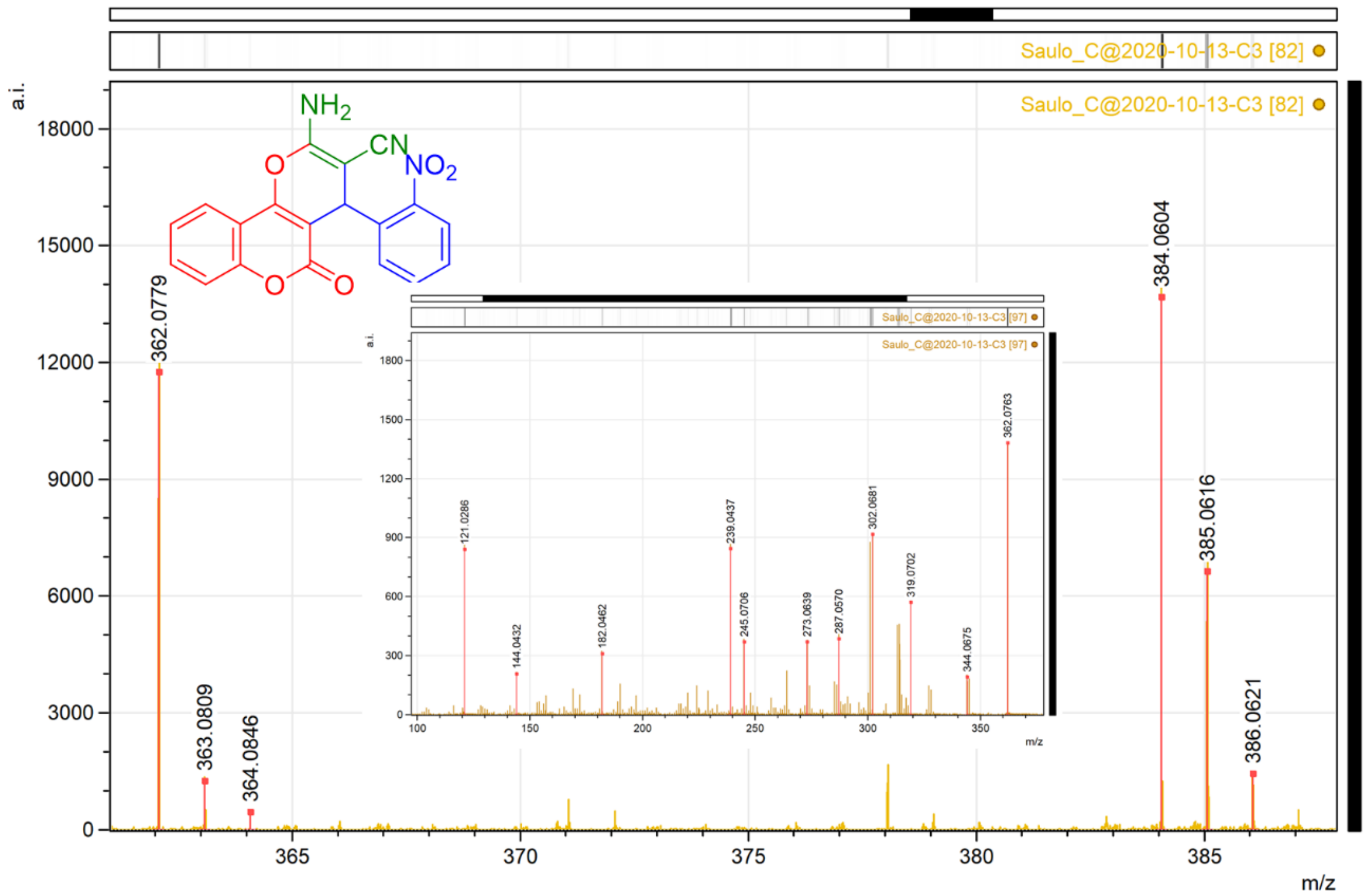

Figure S109. High-resolution ESI(+)-MS of **4r** showing its isotopic pattern. The inset is the ESI(+)-MS/MS from the $[M+H]^+$ isotopologue.

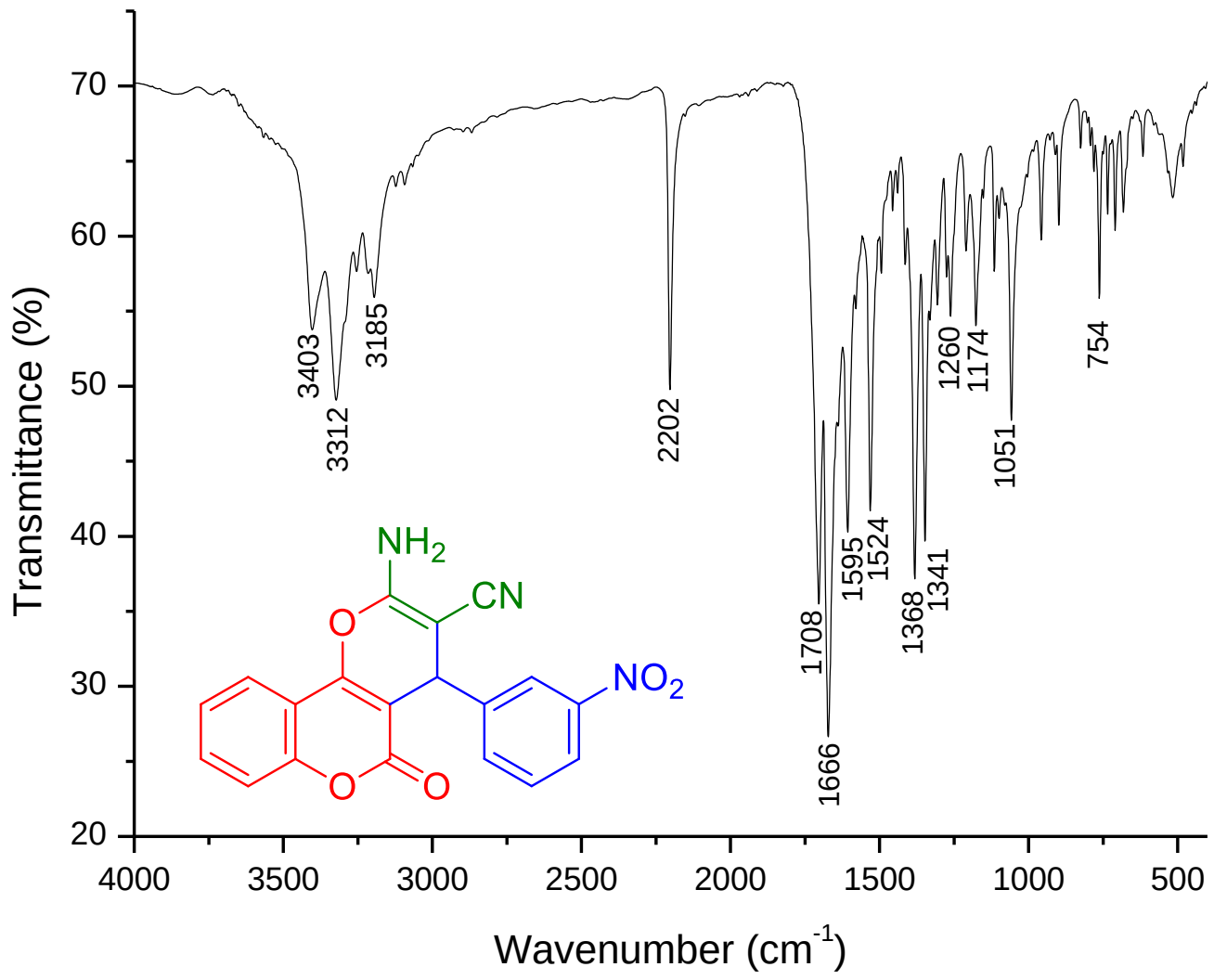

Figure S110. FT-IR (KBr) spectrum of compound **4s**.

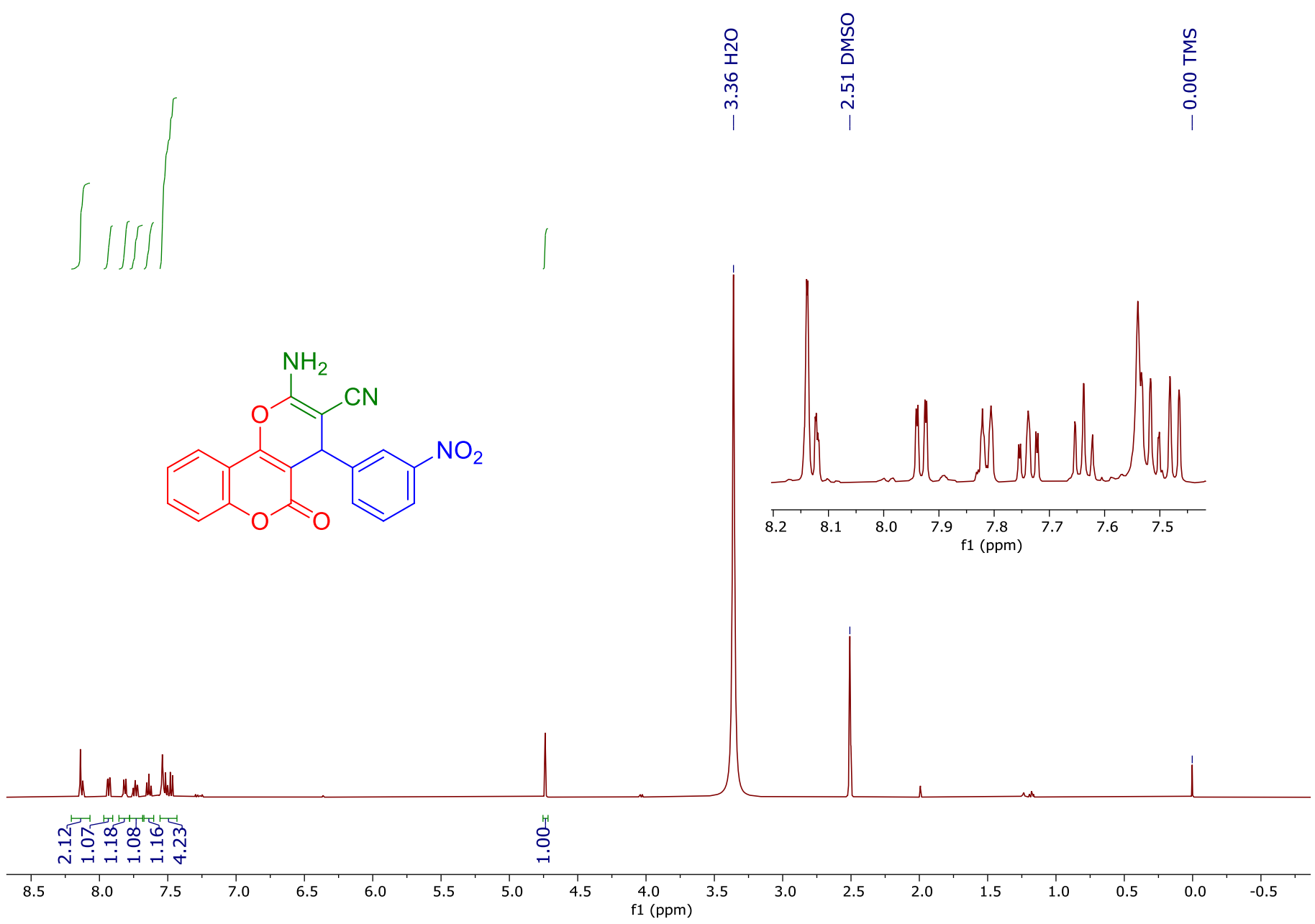

Figure S111. ^{1}H NMR spectrum (500 MHz, DMSO-d_6) of **4s**.

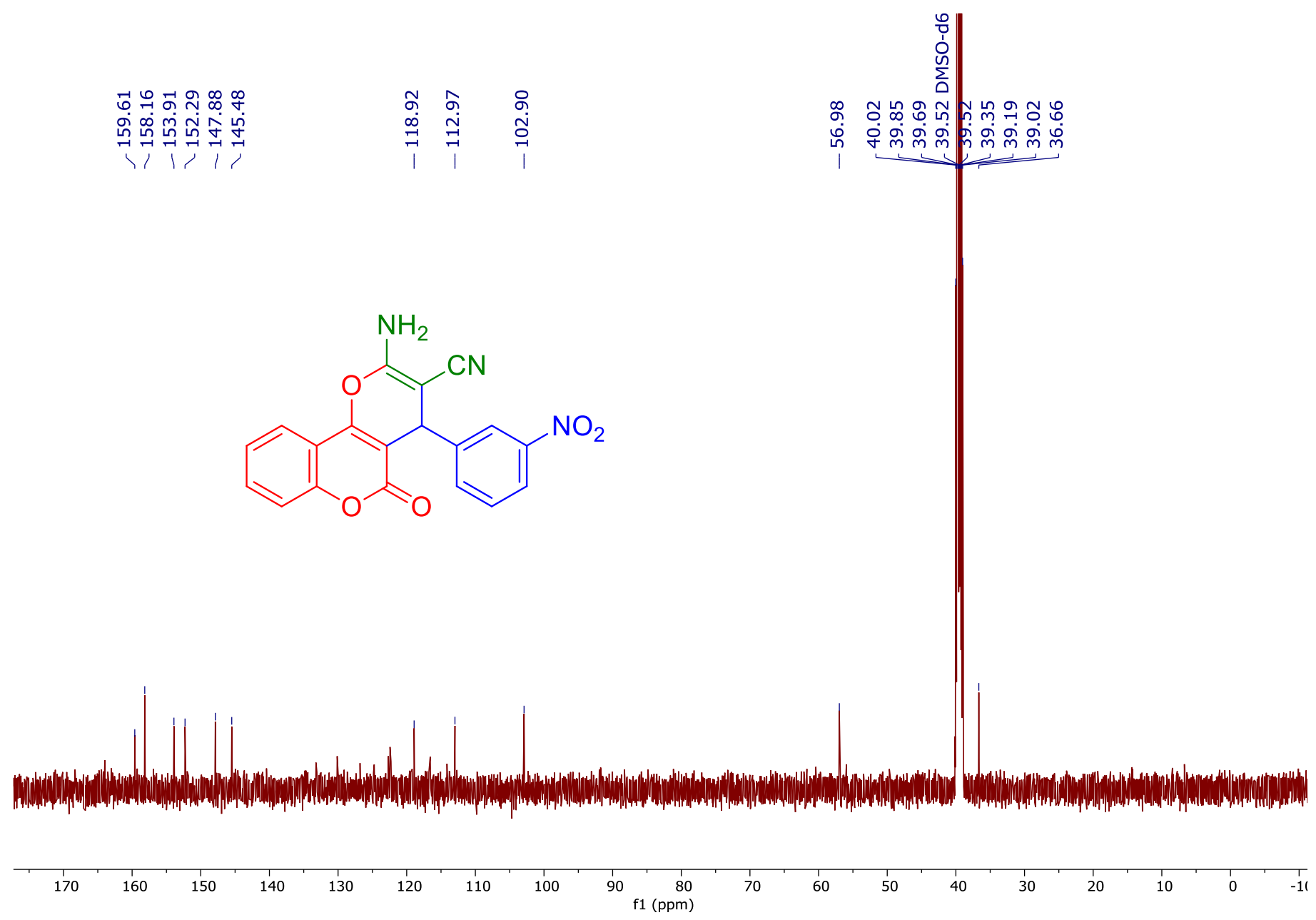

Figure S112. ^{13}C {^{1}H} NMR spectrum (125 MHz, DMSO-d$_6$) of **4s**.

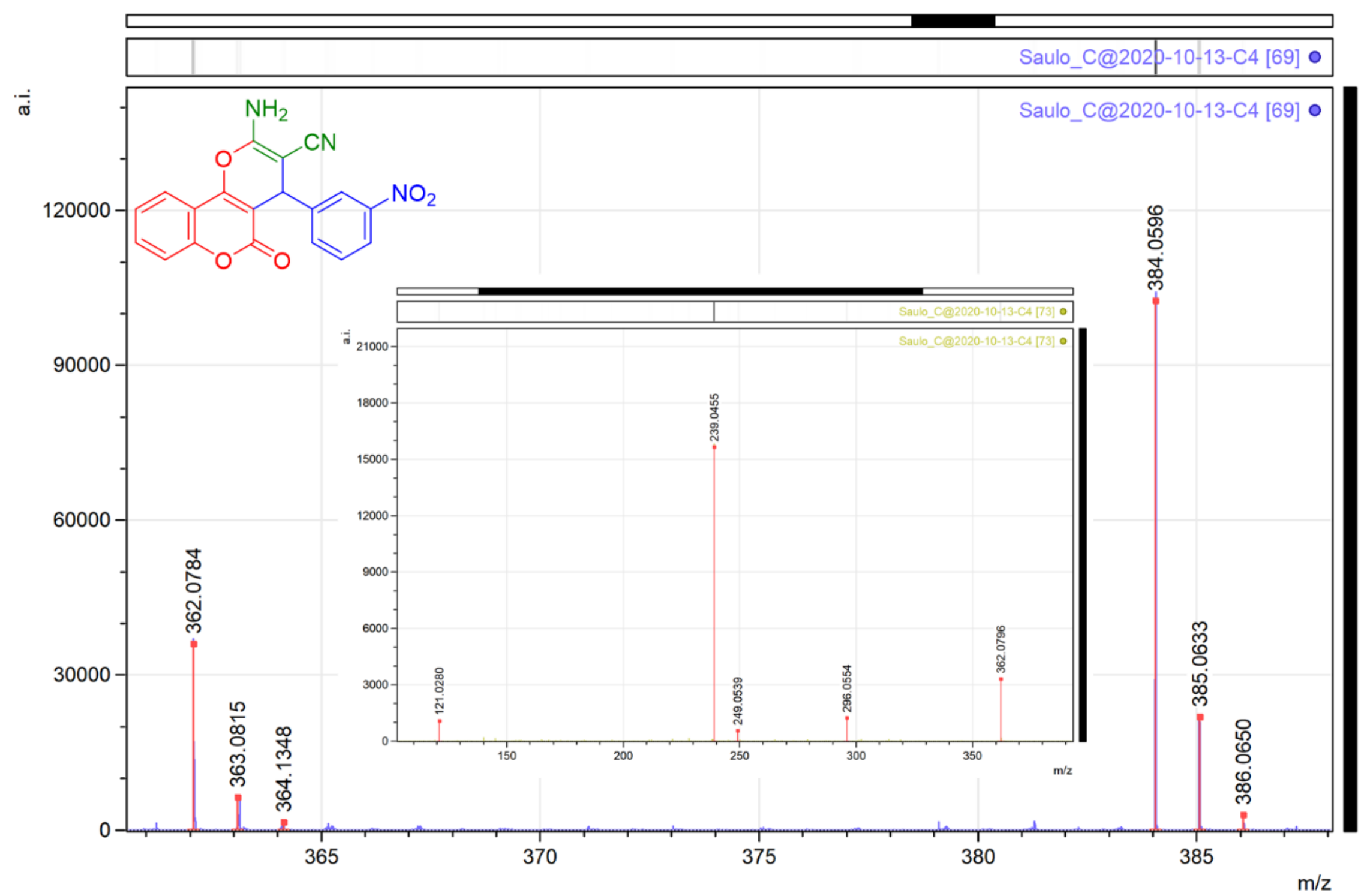

Figure S113. High-resolution ESI(+)-MS of **4s** showing its isotopic pattern. The inset is the ESI(+)-MS/MS from the [M+H]$^{+}$ isotopologue.

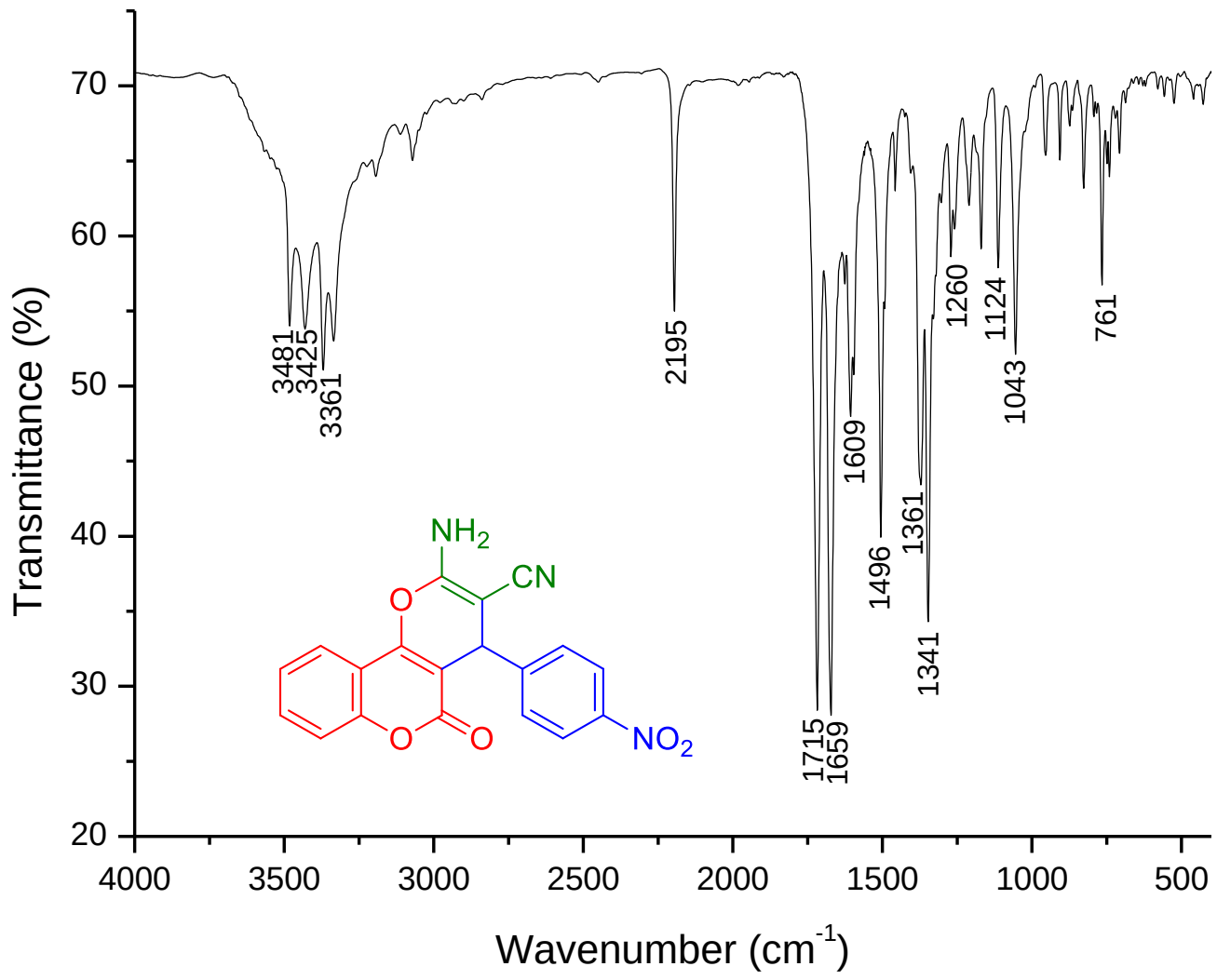

Figure S114. FT-IR (KBr) spectrum of compound **4t**.

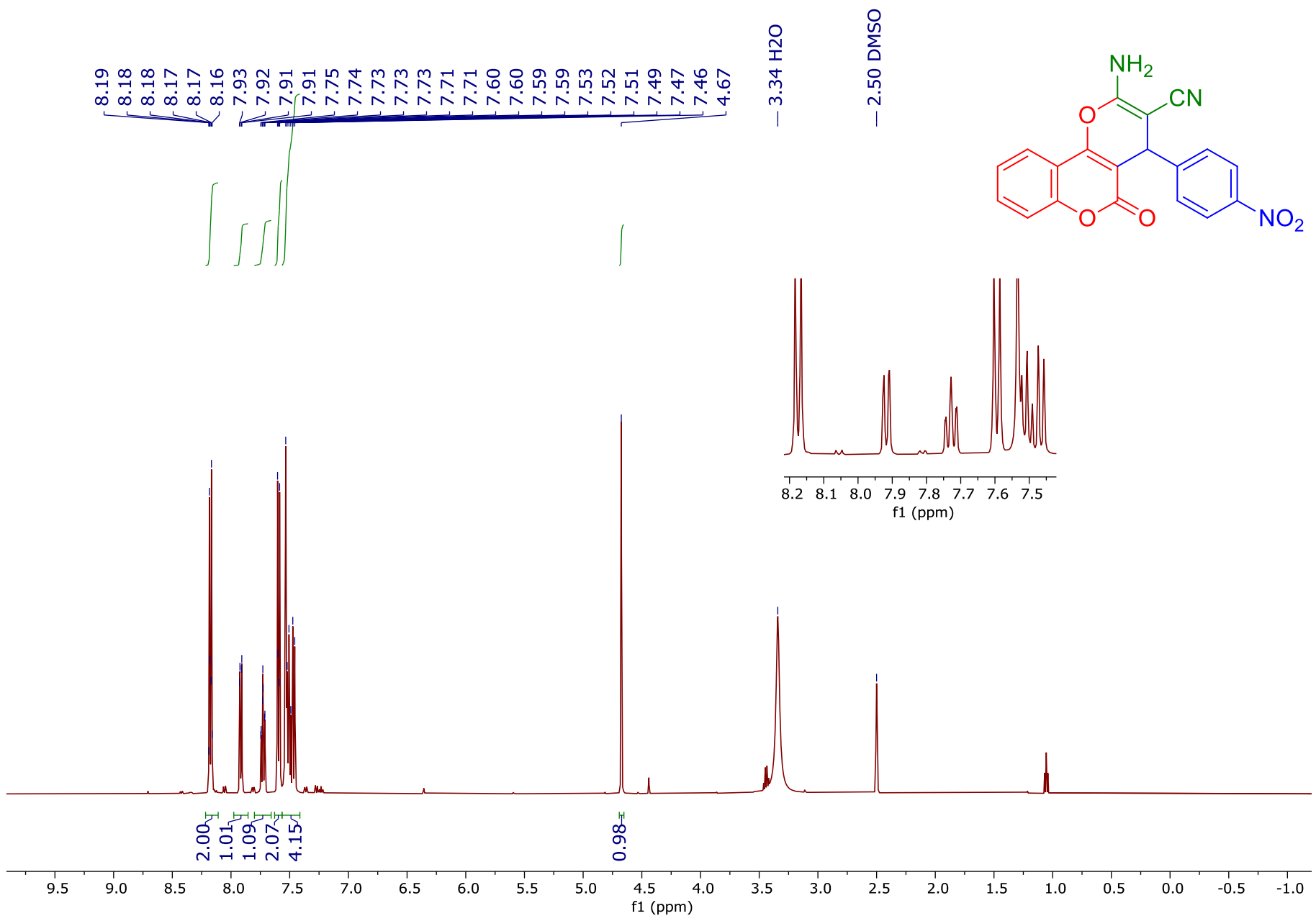

Figure S115. ^{1}H NMR spectrum (500 MHz, DMSO-d_6) of **4t**.

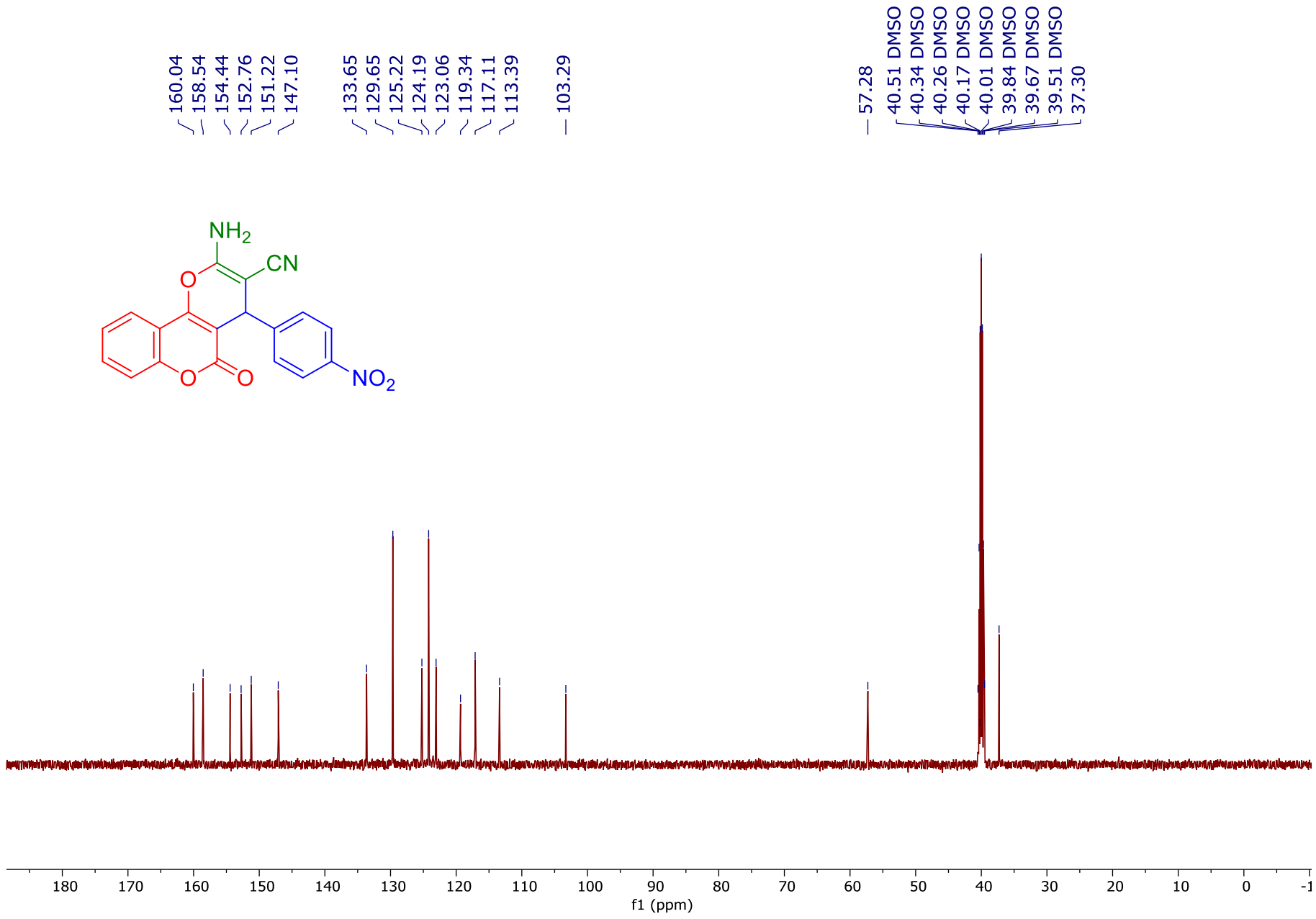

Figure S116. $^{13}C\{^{1}H\}$ NMR spectrum (125 MHz, DMSO-d_6) of **4t**.

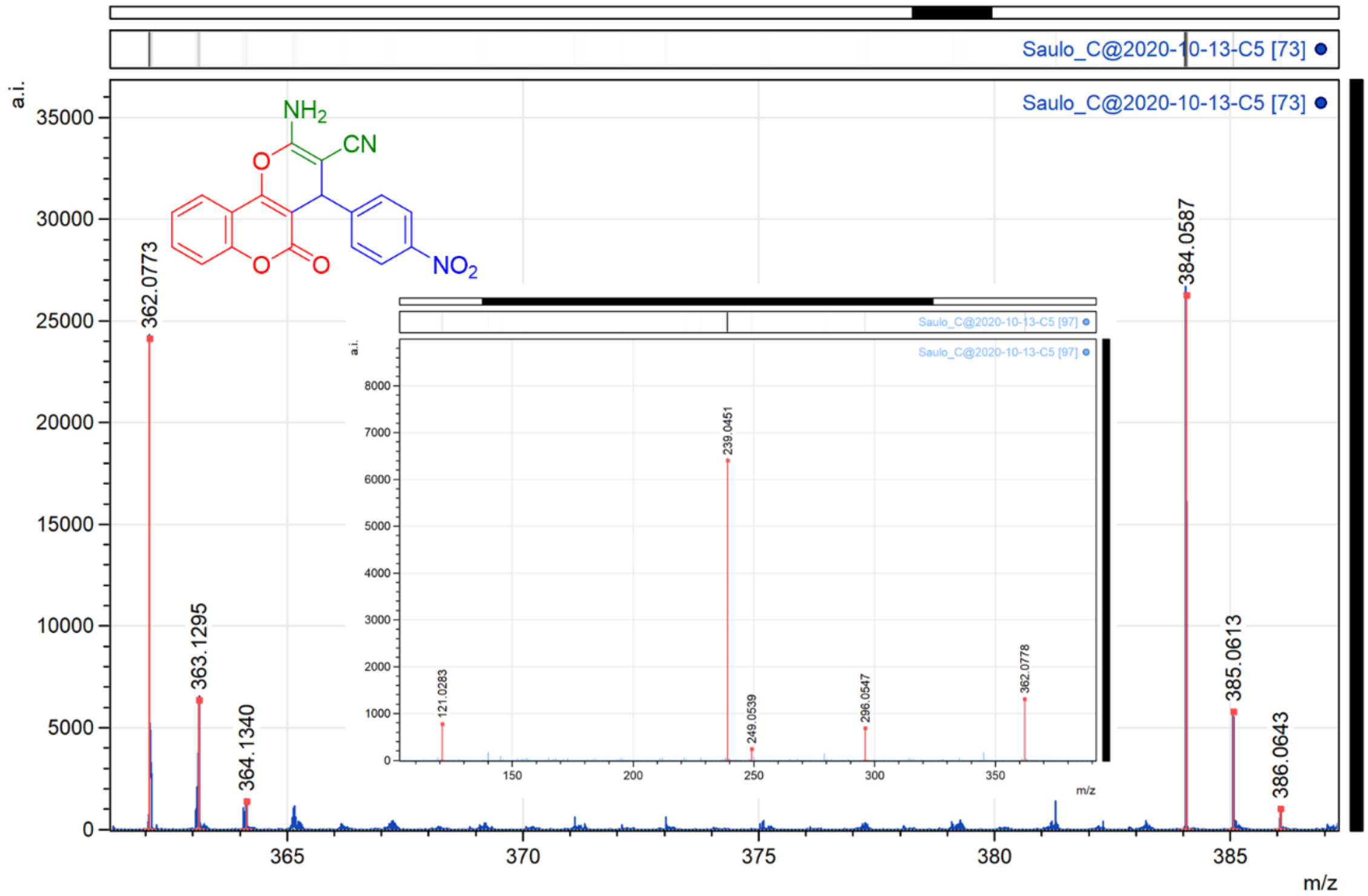

Figure S117. High-resolution ESI(+)-MS of **4t** showing its isotopic pattern. The inset is the ESI(+)-MS/MS from the $[M+H]^+$ isotopologue.

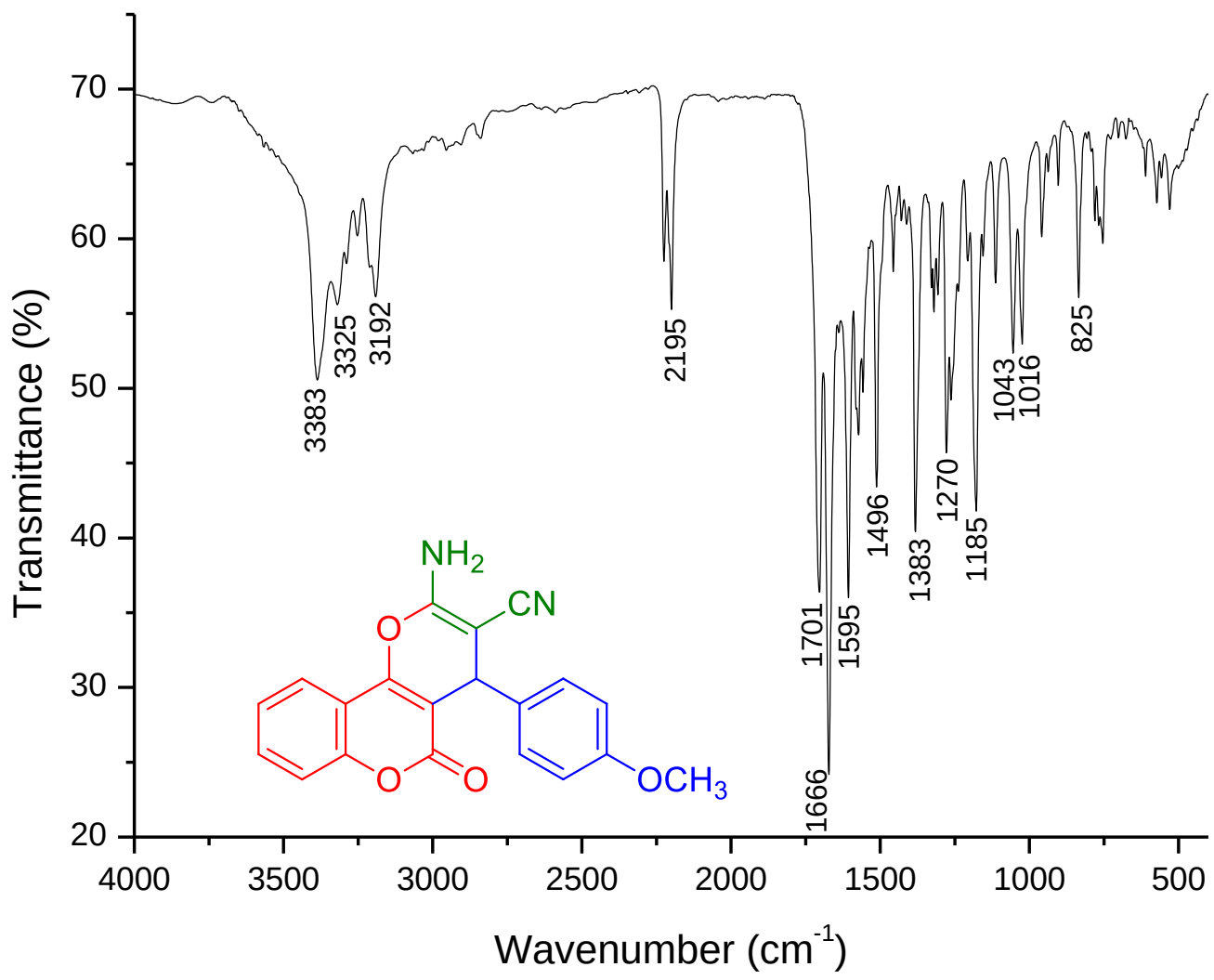

Figure S118. FT-IR (KBr) spectrum of compound **4u**.

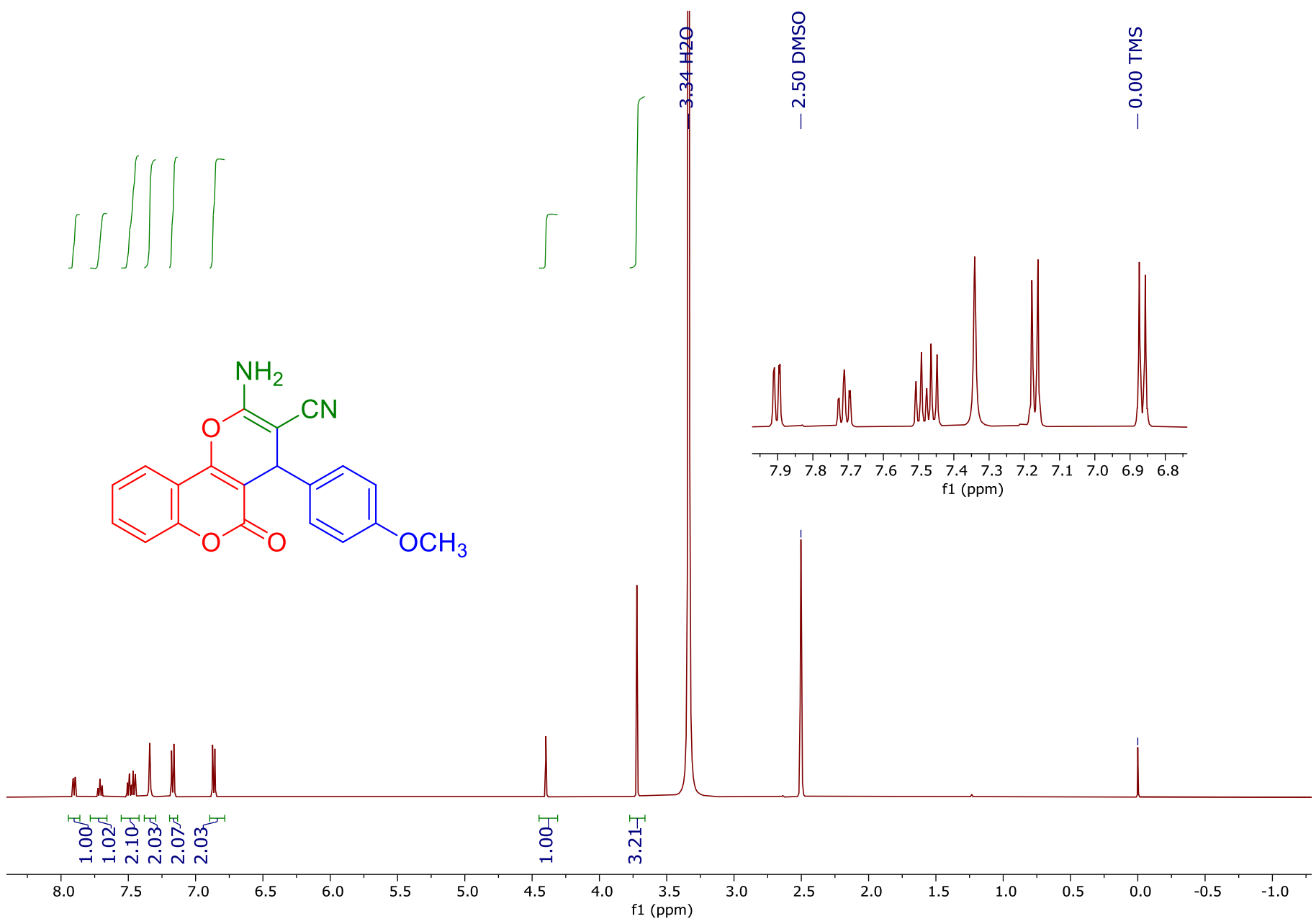

Figure S119. ^{1}H NMR spectrum (500 MHz, DMSO-d_6) of **4u**.

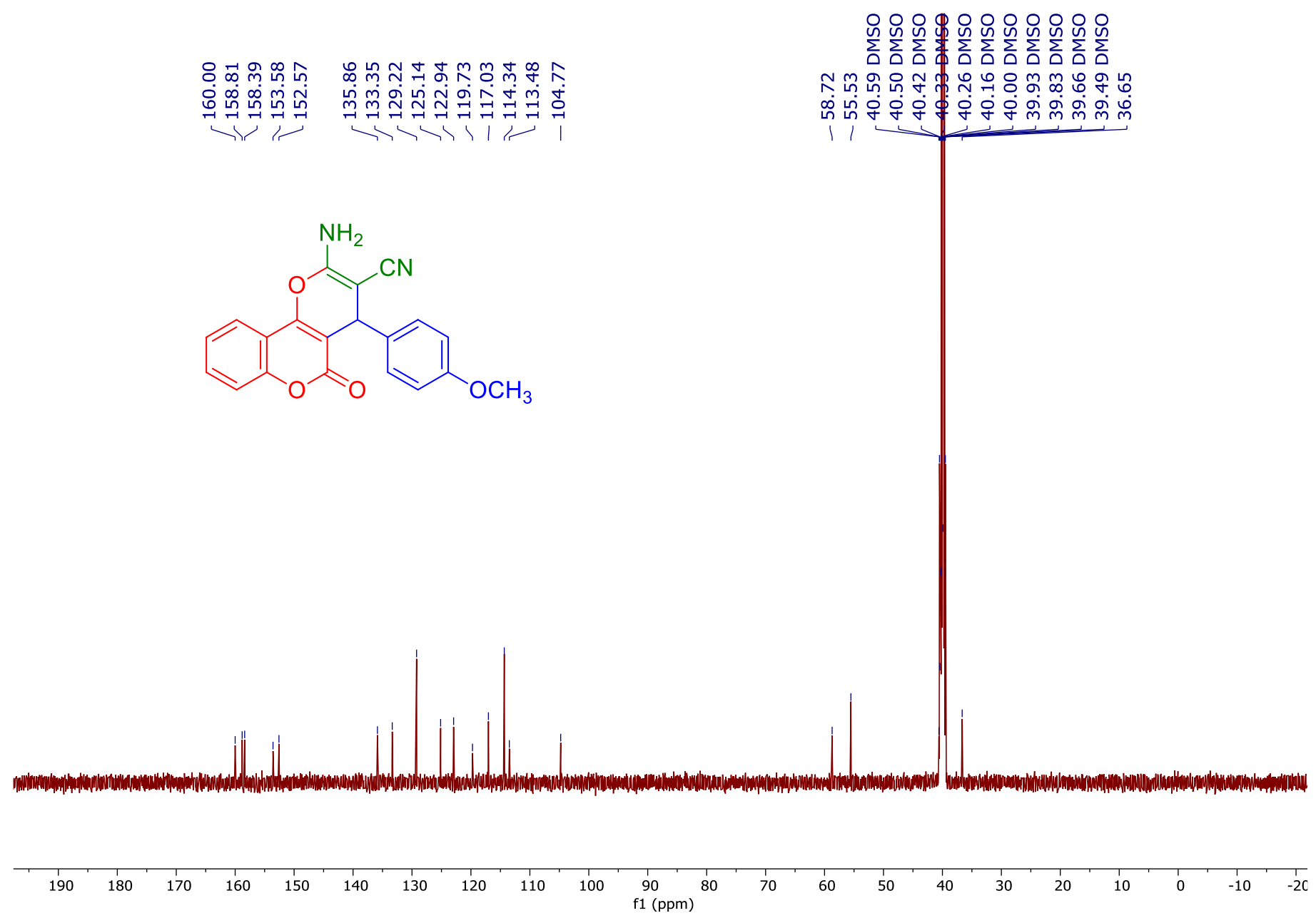

Figure S120. ^{13}C {^{1}H} NMR spectrum (125 MHz, DMSO-d$_6$) of **4u**.

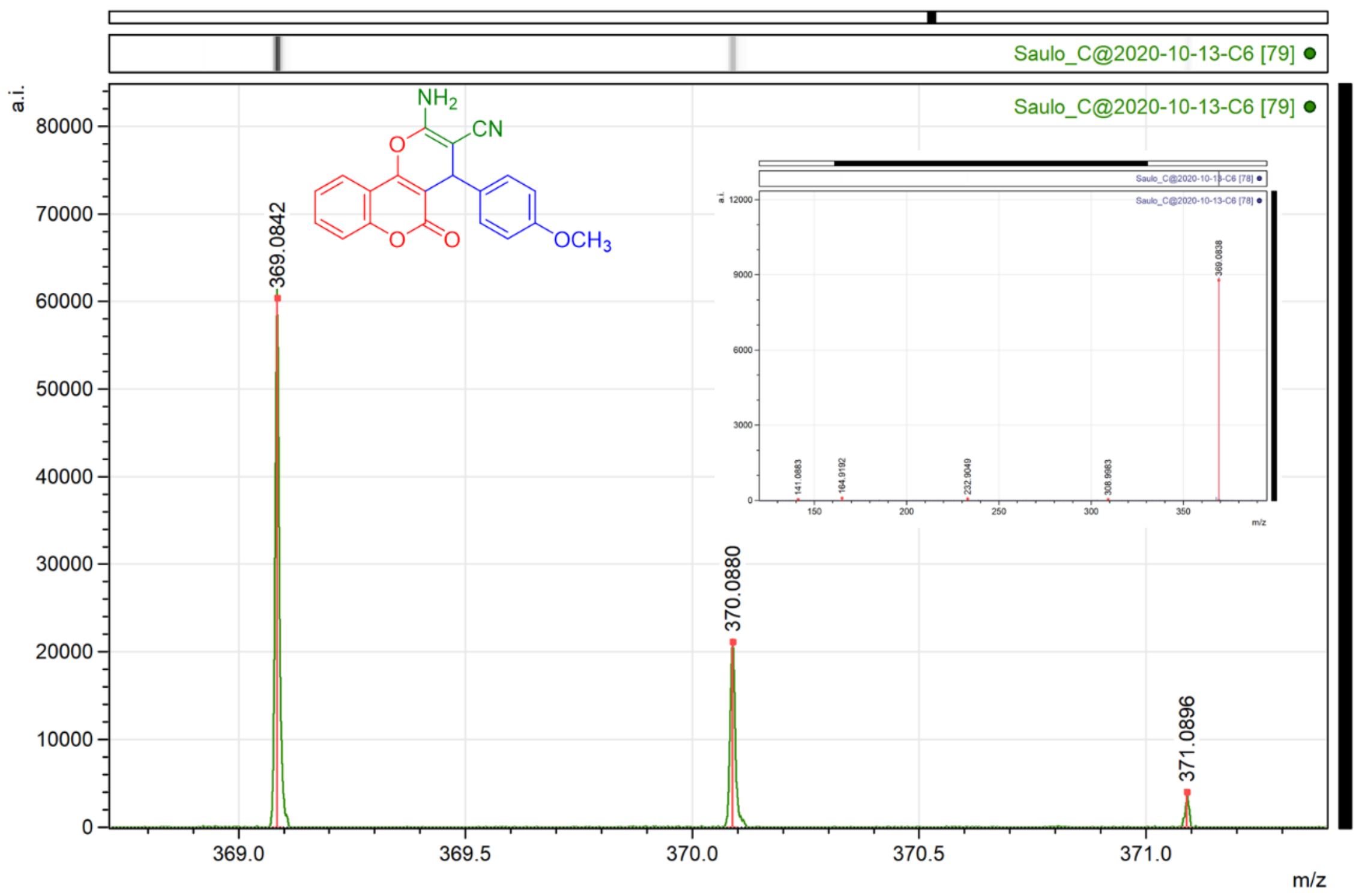

Figure S121. High-resolution ESI(+)-MS of **4u** showing its isotopic pattern. The inset is the ESI(+)-MS/MS from the [M+Na]$^+$ isotopologue.

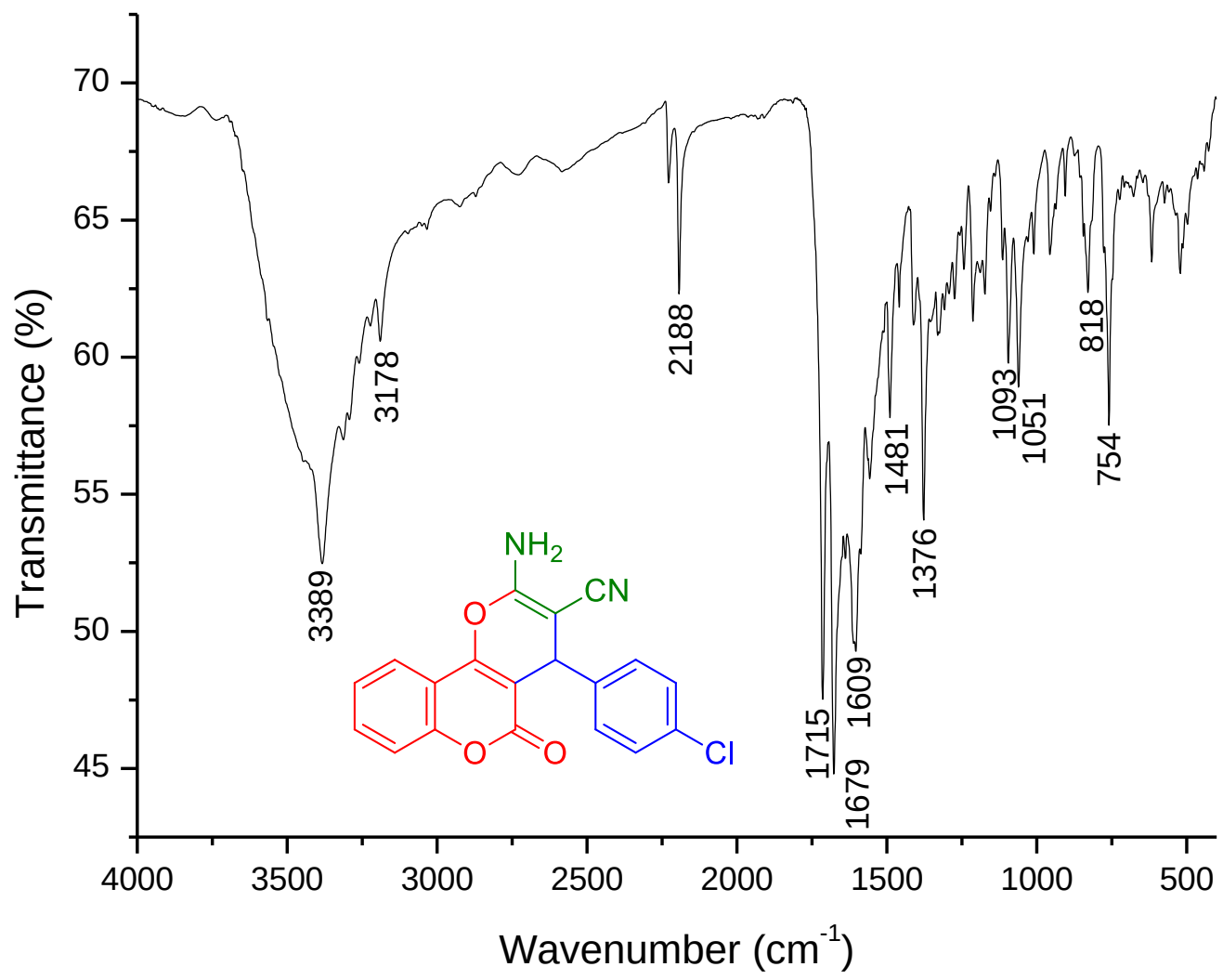

Figure S122. FT-IR (KBr) spectrum of compound **4v**.

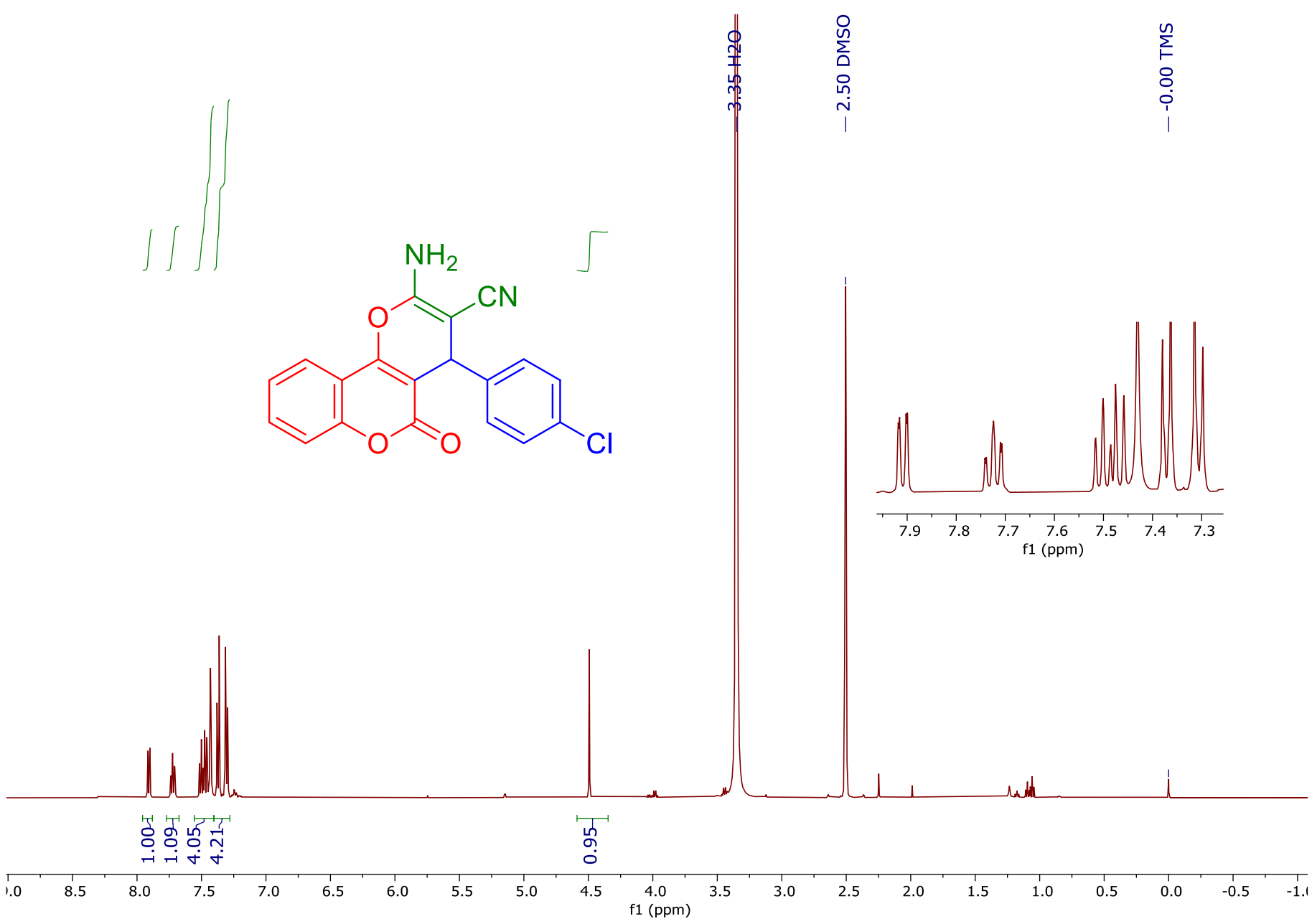

Figure S123. ^{1}H NMR spectrum (500 MHz, DMSO-d_6) of **4v**.

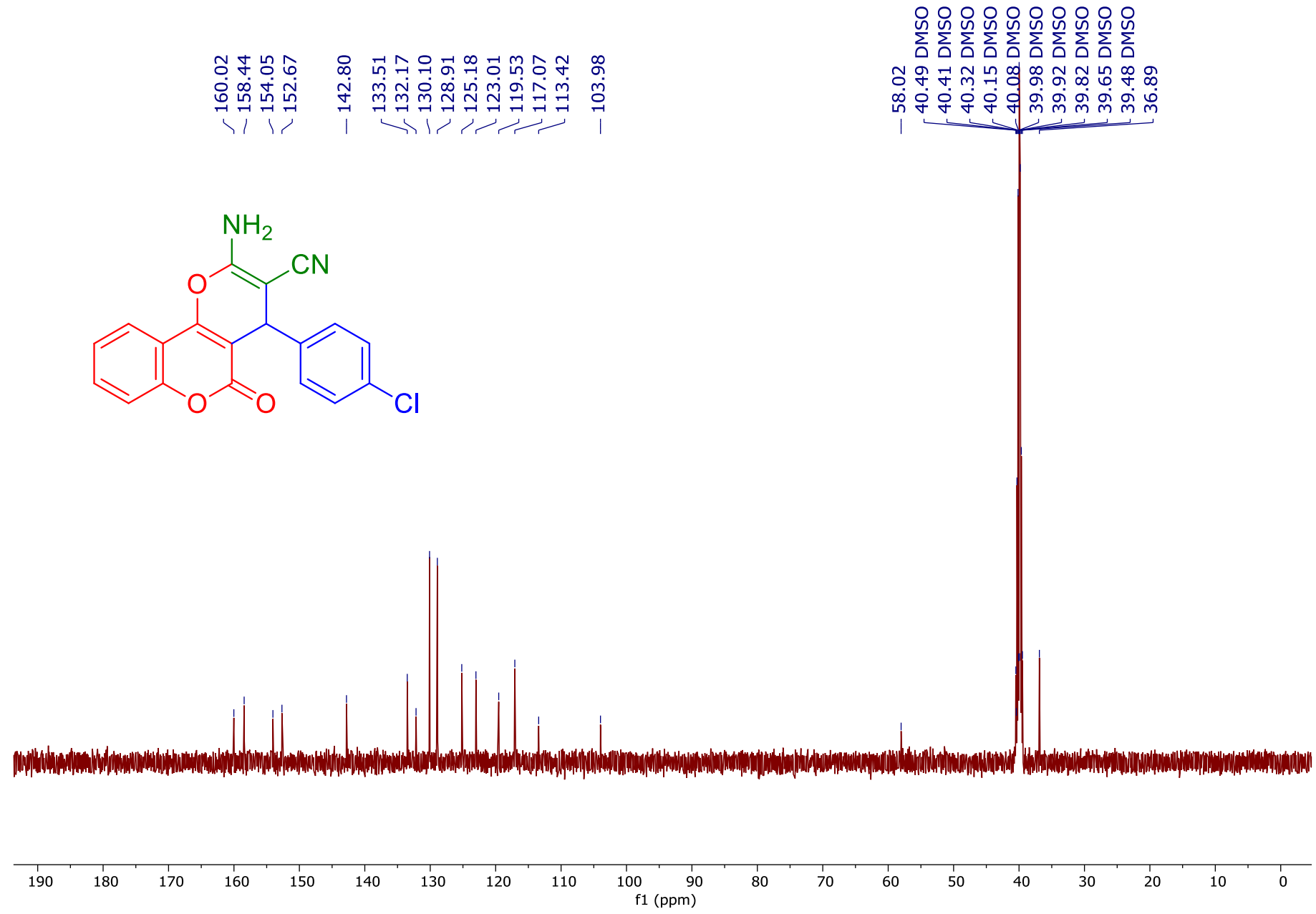

Figure S124. $^{13}C\{^{1}H\}$ NMR spectrum (125 MHz, DMSO-d_6) of **4v**.

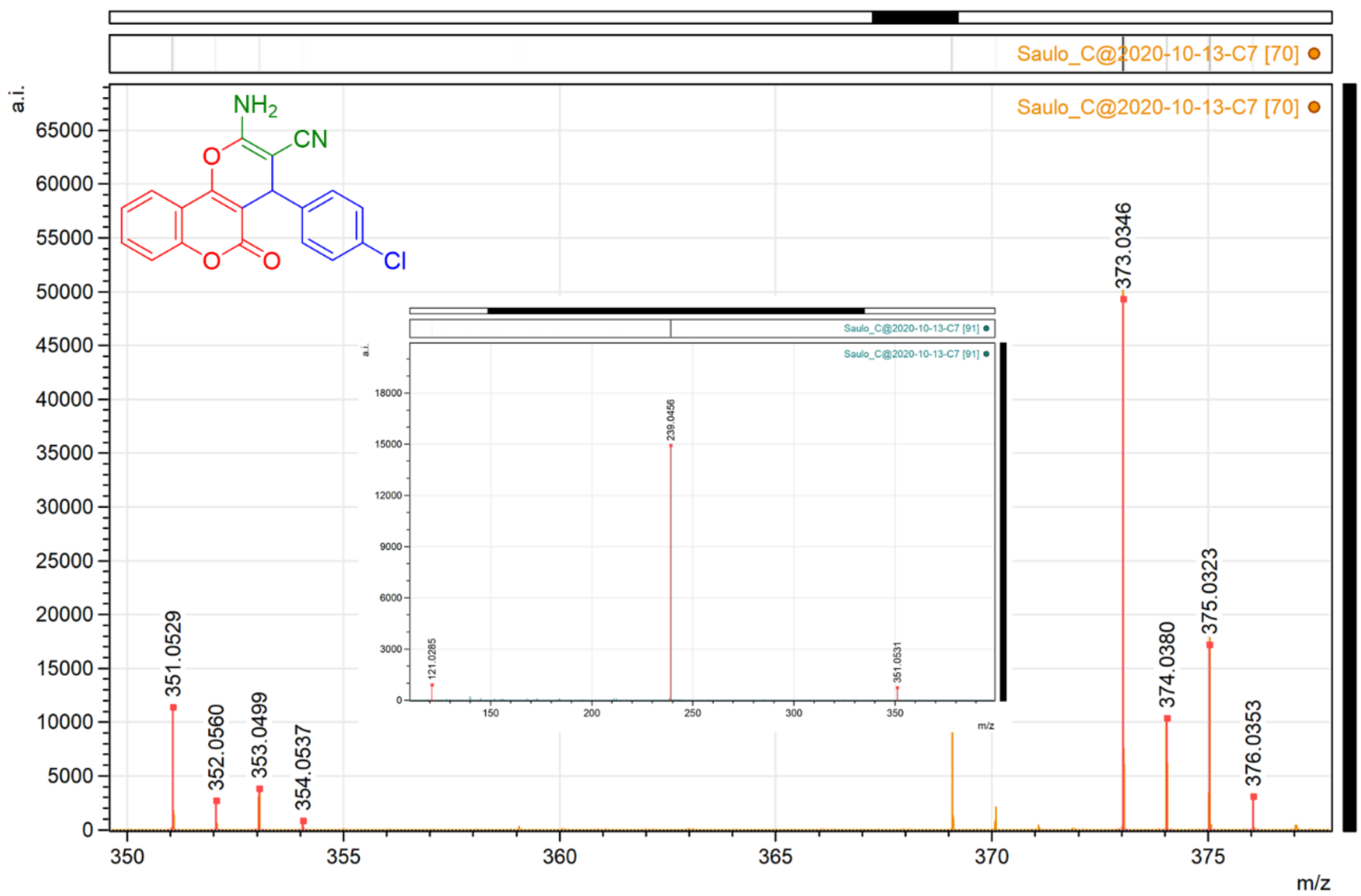

Figure S125. High-resolution ESI(+)-MS of **4v** showing its isotopic pattern. The inset is the ESI(+)-MS/MS from the $[M+H]^+$ isotopologue.

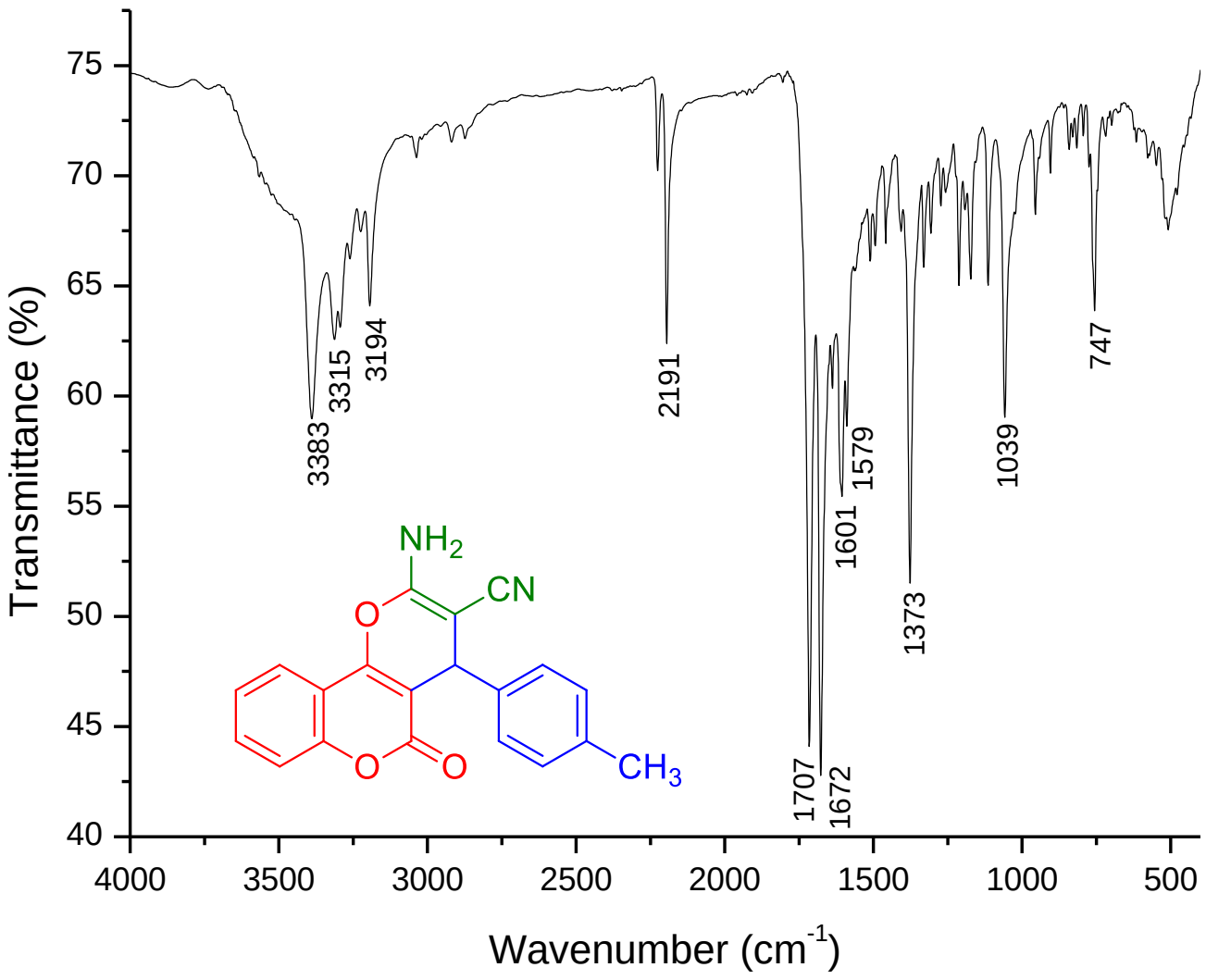

Figure S126. FT-IR (KBr) spectrum of compound **4w**.

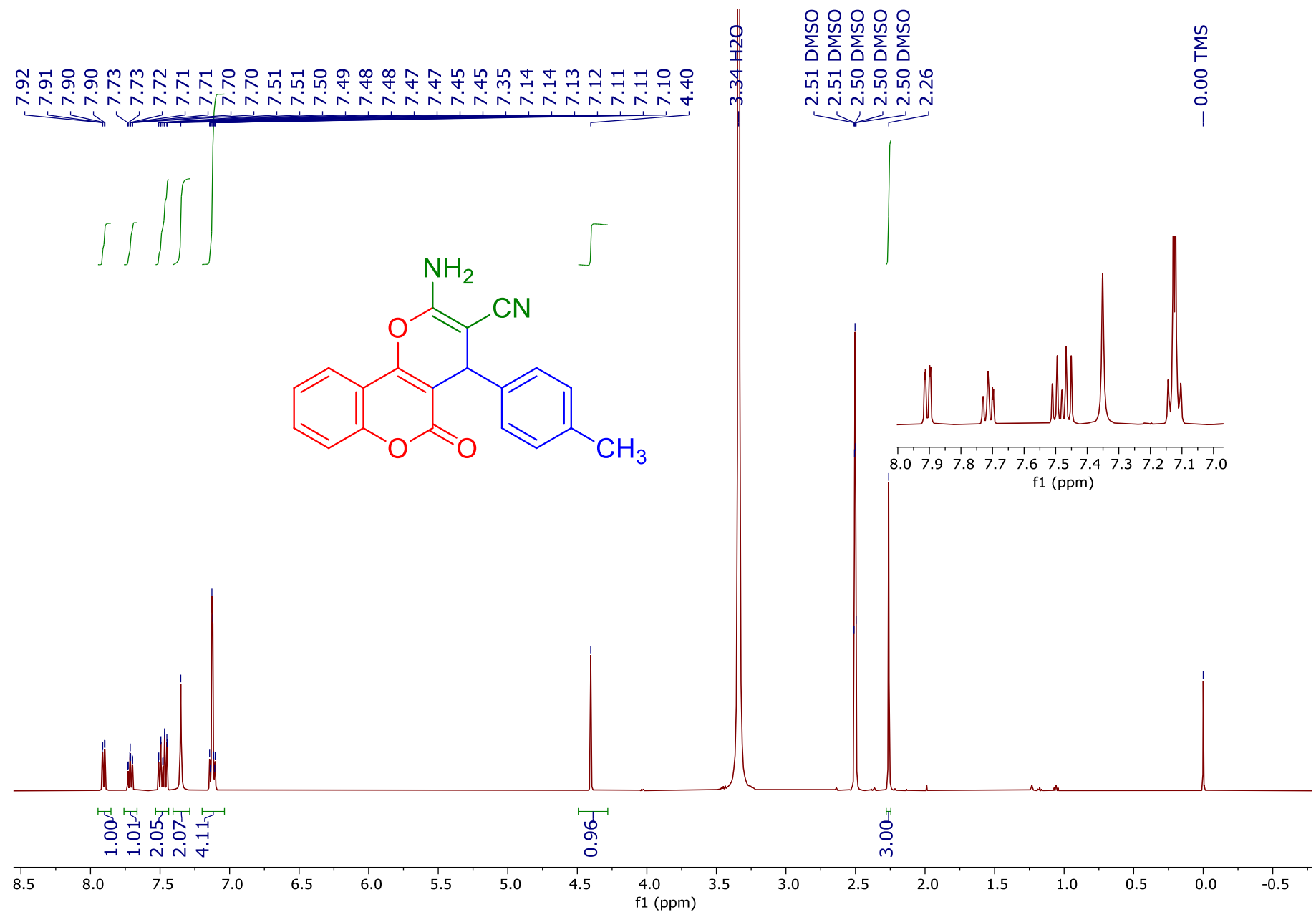

Figure S127. ^{1}H NMR spectrum (500 MHz, DMSO-d_6) of **4w**.

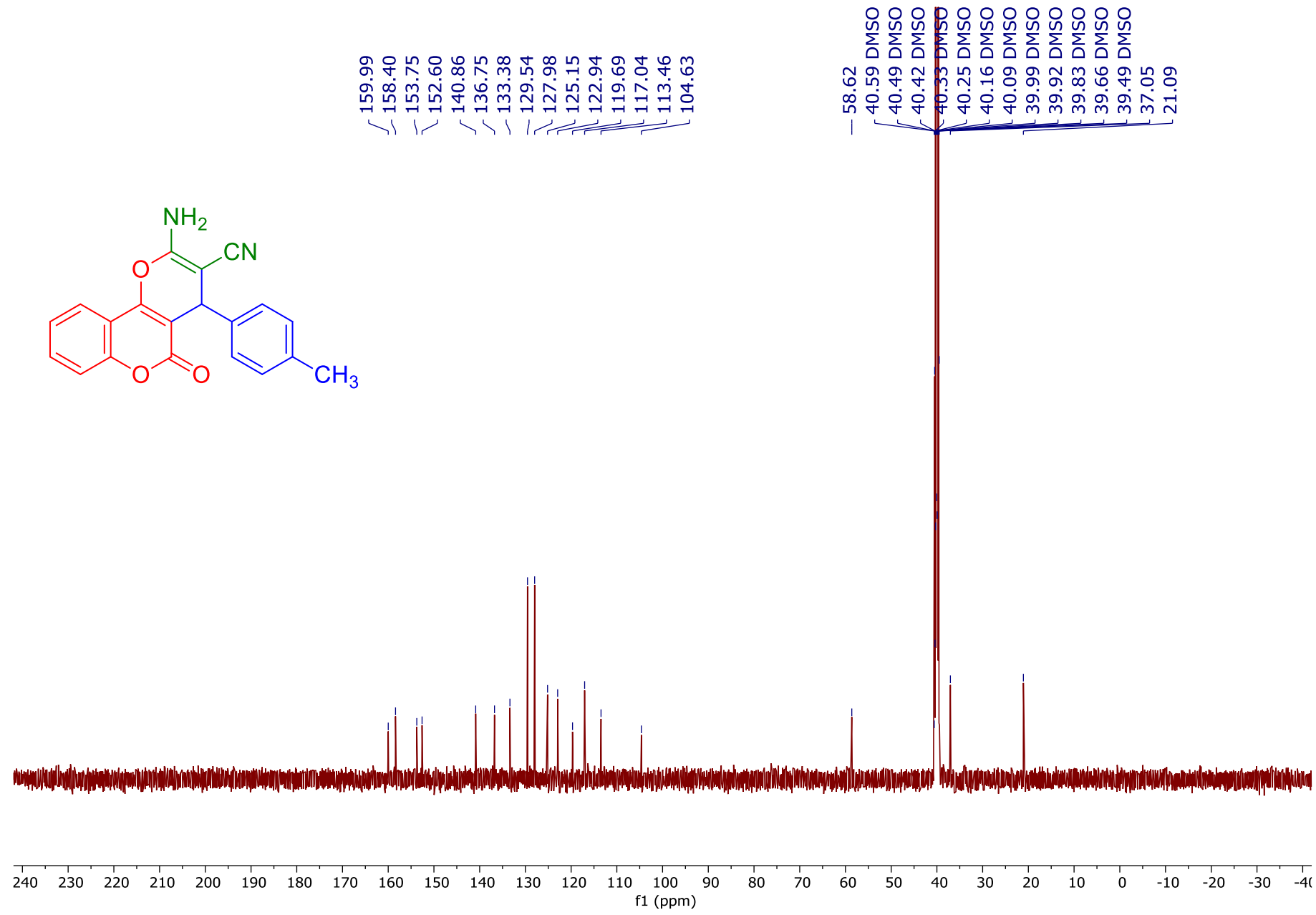

Figure S128. ^{13}C {^{1}H} NMR spectrum (125 MHz, DMSO-d_6) of **4w**.

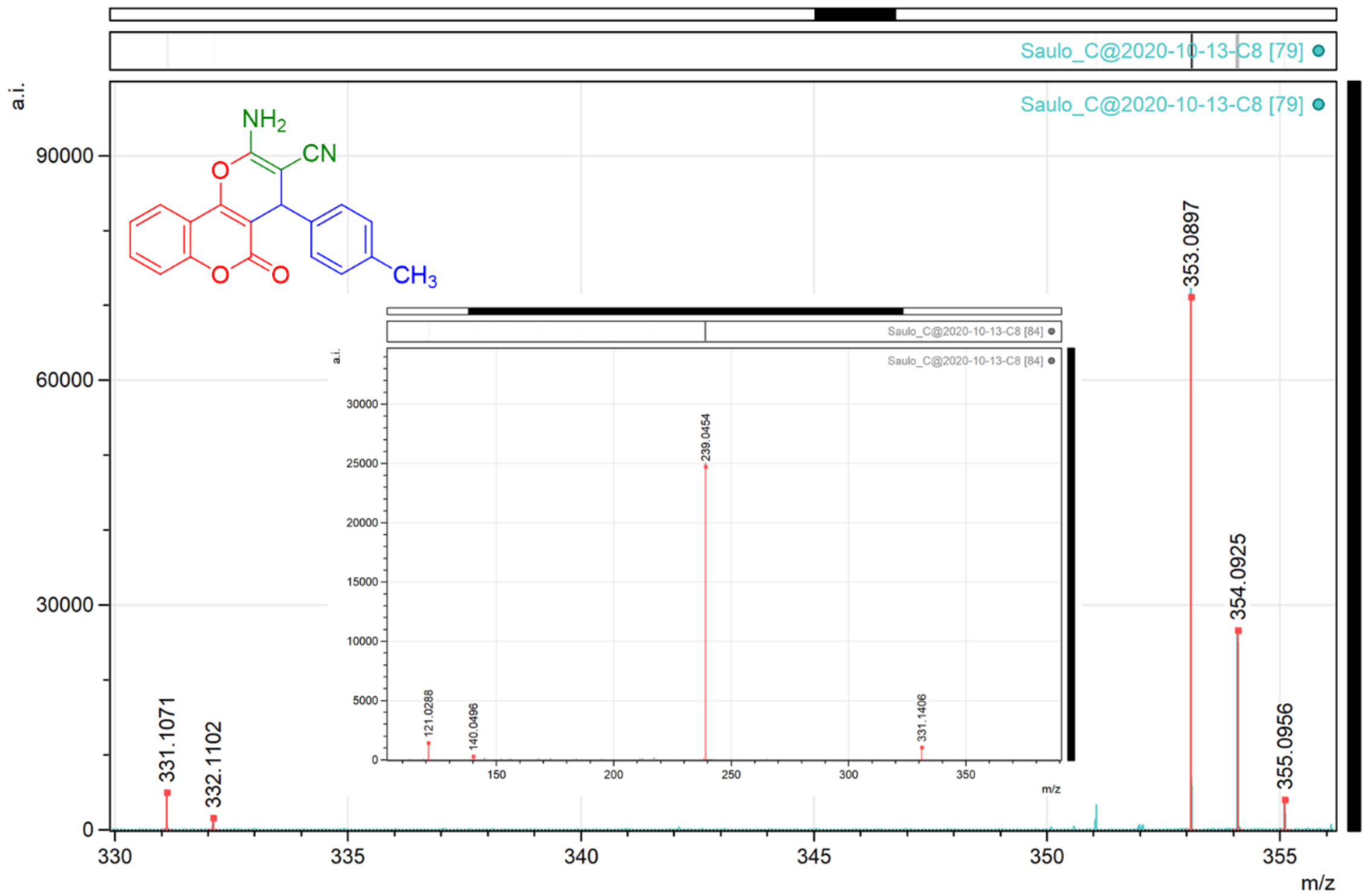

Figure S129. High-resolution ESI(+)-MS of **4w** showing its isotopic pattern. The inset is the ESI(+)-MS/MS from the $[M+H]^+$ isotopologue.

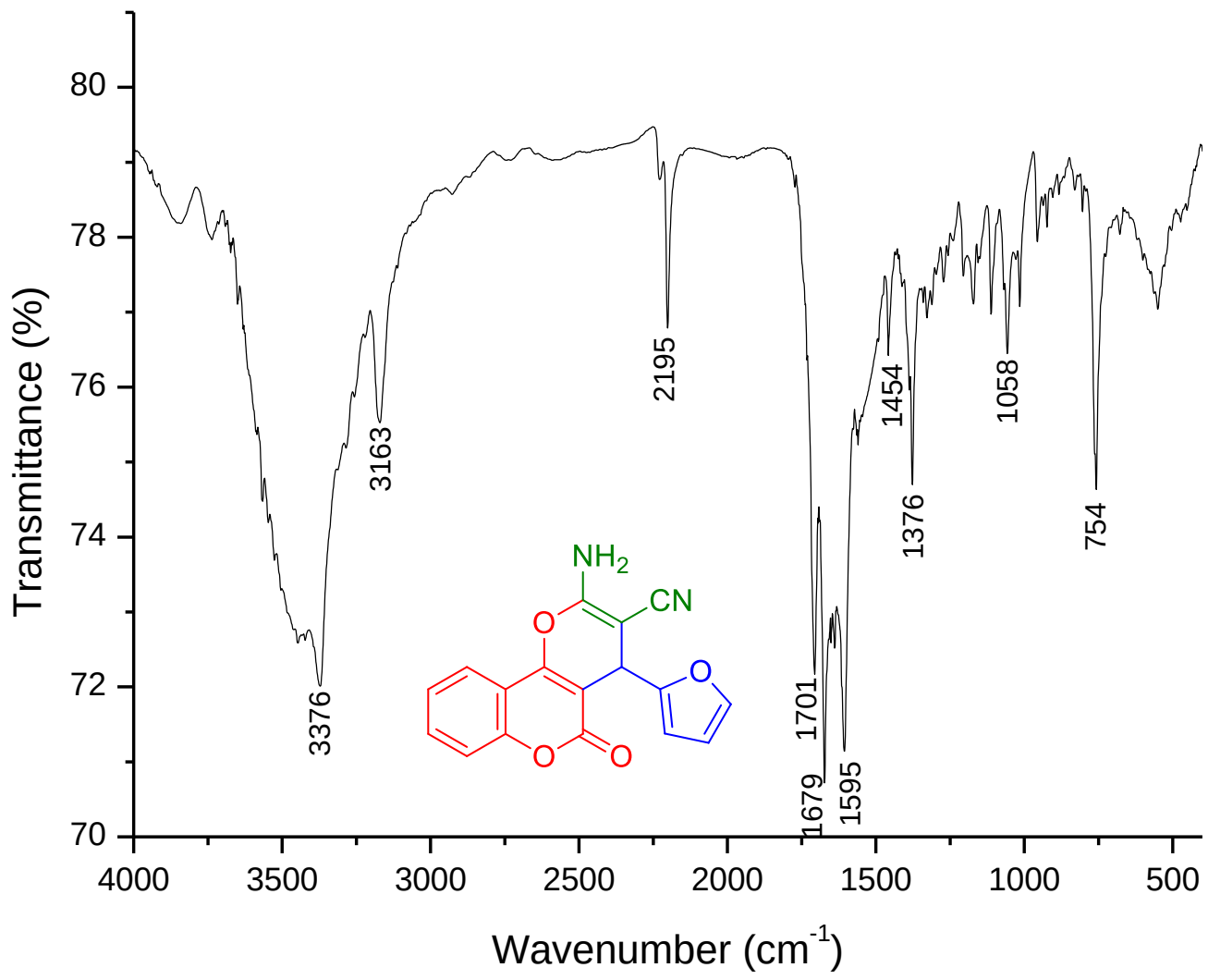

Figure S130. FT-IR (KBr) spectrum of compound **4x**.

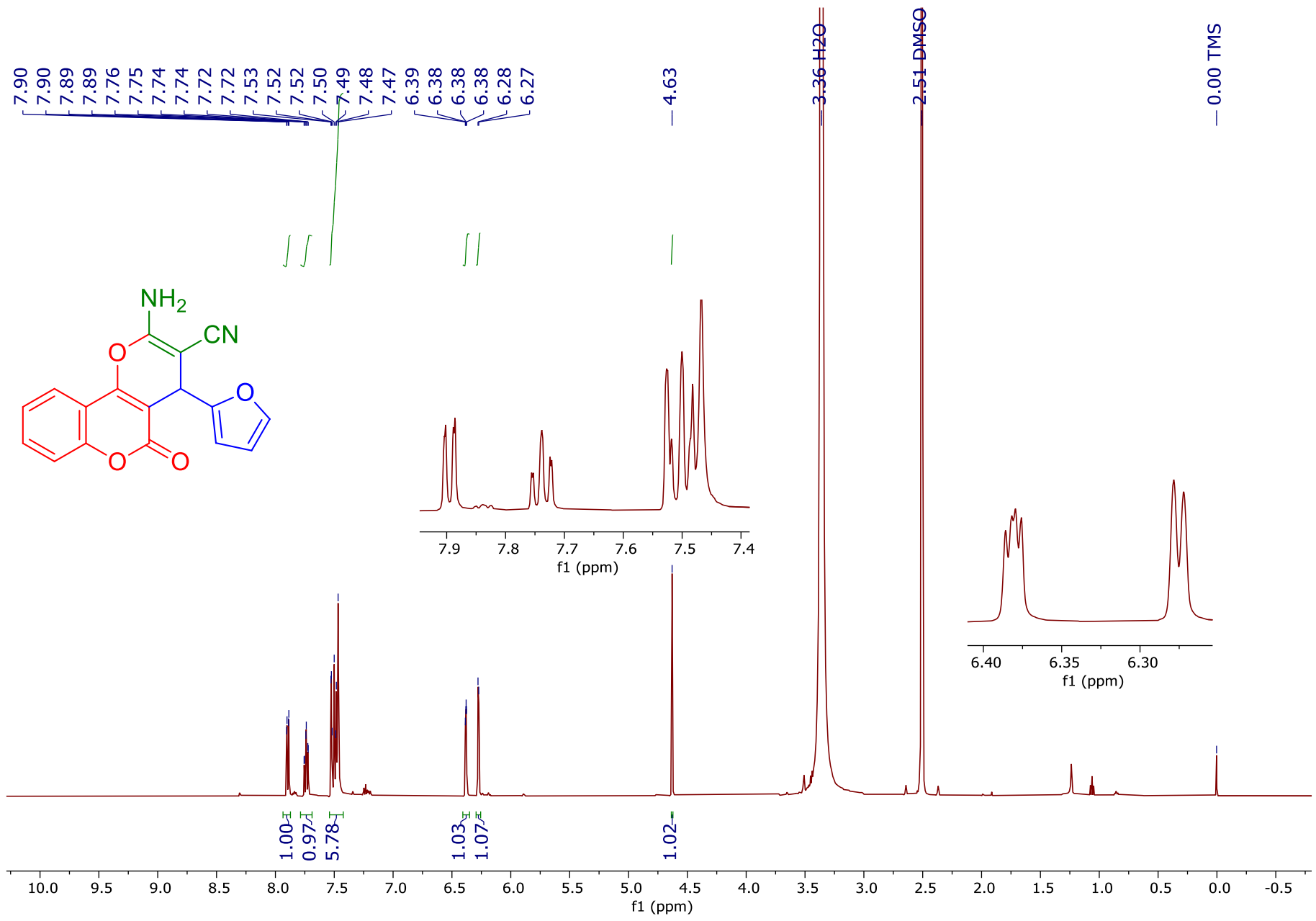

Figure S131. ^{1}H NMR spectrum (500 MHz, DMSO-d_6) of **4x**.

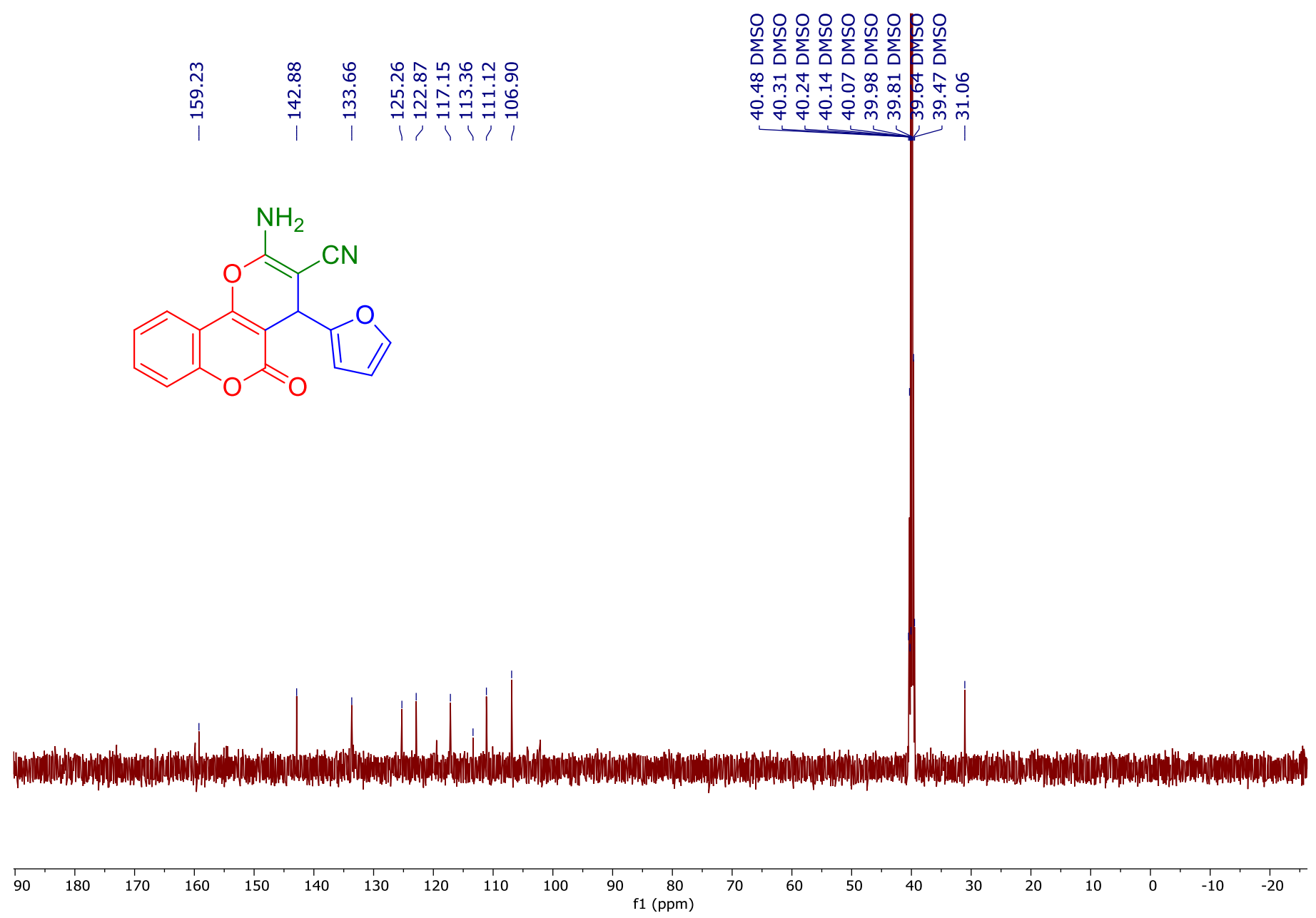

Figure S132. $^{13}C\{^1H\}$ NMR spectrum (125 MHz, DMSO-d_6) of **4x**.

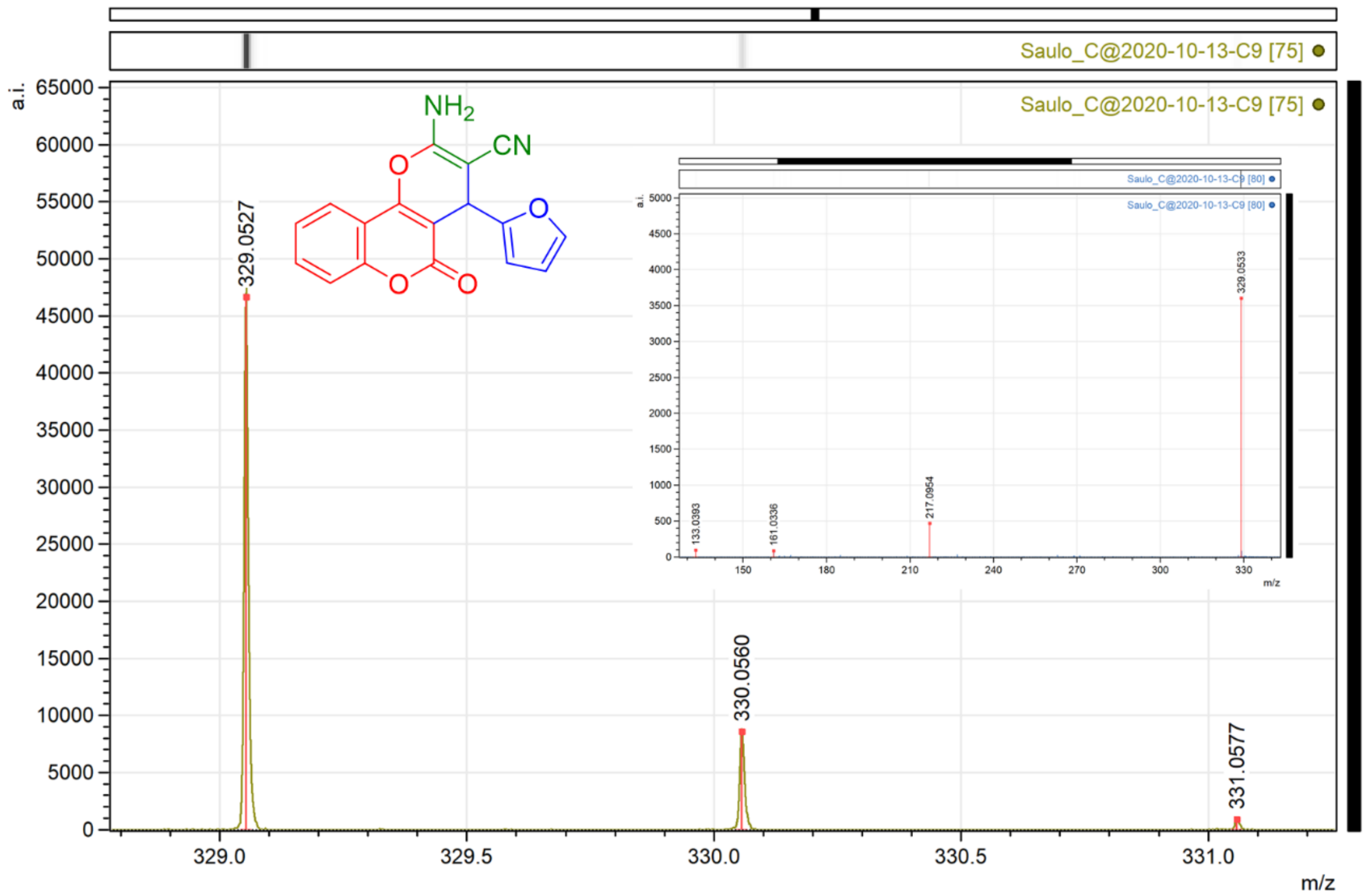

Figure S133. High-resolution ESI(+)-MS of **4x** showing its isotopic pattern. The inset is the ESI(+)-MS/MS from the $[M+Na]^+$ isotopologue.
